# Genome-wide identification of the *GhARF* gene family reveals that *GhARF2* and *GhARF18* are involved in cotton fibre cell initiation

**DOI:** 10.1093/jxb/ery219

**Published:** 2018-06-12

**Authors:** Guanghui Xiao, Peng He, Peng Zhao, Hao Liu, Li Zhang, Chaoyou Pang, Jianing Yu

**Affiliations:** 1 Key Laboratory of the Ministry of Education for Medicinal Plant Resources and Natural Pharmaceutical Chemistry, National Engineering Laboratory for Resource Development of Endangered Crude Drugs in the Northwest of China, College of Life Sciences, Shaanxi Normal University, Xi’an, China; 2 College of Life Sciences, Shaanxi Normal University, Xi’an, China; 3 State Key Laboratory of Cotton Biology, Institute of Cotton Research of Chinese Academy of Agricultural Sciences, Anyang, China

**Keywords:** Auxin, auxin response factor, cotton, gene expression, seed fibre initiation

## Abstract

Auxin signalling plays an essential role in regulating plant development. Auxin response factors (ARFs), which are critical components of auxin signalling, modulate the expression of early auxin-responsive genes by binding to auxin response factor elements (AuxREs). However, there has been no comprehensive characterization of this gene family in cotton. Here, we identified 56 *GhARF* genes in the assembled *Gossypium hirsutum* genome. This gene family was divided into 17 subfamilies, and 44 members of them were distributed across 21 chromosomes. *GhARF6* and *GhARF11* subfamily genes were predominantly expressed in vegetative tissues, whereas *GhARF2* and *GhARF18* subfamily genes were highly expressed during seed fibre cell initiation. *GhARF2-1* and *GhARF18-1* were exclusively expressed in trichomes, organs similar to cotton seed fibre cells, and overexpression of these genes in Arabidopsis enhances trichome initiation. Comparative transcriptome analysis combined with AuxRE prediction revealed 11 transcription factors as potential target genes of *GhARF2* and *GhARF18*. Six of these genes were significantly expressed during seed fibre cell initiation and were bound by GhARF2-1 and GhARF18-1 in yeast one-hybrid assays. Our results suggest that *GhARF2* and *GhARF18* genes may be key regulators of cotton seed fibre initiation by regulating the expression of several transcription factor genes. This study deepens our understanding of auxin-mediated initiation of cotton seed fibre cells and helps us in breeding better cotton varieties in the future.

## Introduction

The phytohormone auxin plays a pivotal role in regulating many distinct aspects of plant development, including lateral root initiation, shoot elongation, embryogenesis, vascular growth, tropic development, and flowering and tissue architecture (Teale *et al.*, 2006; Domagalska and Leyser, 2011). Auxin response factors (ARFs), which are important components of auxin signalling pathways, regulate the expression of auxin-responsive genes by directly binding to the auxin-responsive element (AuxRE, TGTCTC) in their promoter regions (Guilfoyle and Hagen, 2007). A typical ARF protein consists of three conserved domains: an N-terminal B3-type DNA-binding domain (DBD) that recognizes the AuxRE; a variable middle region (MR) that activates or represses the expression of auxin-responsive genes depending on its amino acid composition; and two C-terminal dimerization domains (CTDs) that are involved in the formation of homo- and heterodimers between ARFs and auxin/indole-3-acetic acid (Aux/IAA) proteins (Gray *et al.*, 2001). The Aux/IAA proteins can act as repressors of ARF-mediated transcription by forming multimers with ARFs and recruit the co-repressor TOPLESS (TPL) and its family proteins (TPRs) through a typical EAR motif to prevent activation of auxin-responsive genes by activating ARFs (Leyser, 2018). It has been shown that ubiquitination of Aux/IAA proteins by the transport inhibitor response 1 (TIR1) subunit of the SCF^TIR1/AFB^ ubiquitin ligase accelerates Aux/IAA protein degradation by the 26S proteasome (Kepinski and Leyser, 2005). Aux/IAA proteins bind to ARFs and inhibit their binding to downstream target genes. Thus, when the inhibition of ARFs by Aux/IAA proteins is released, they modulate the expression of downstream genes.

The functions of plant ARF proteins, which are encoded by a large gene family, have been identified by classical genetic approaches. In *Arabidopsis thaliana*, the first identified *ARF* gene was *AtARF1*. Mutations in *AtARF1* were found to enhance the phenotype of the *arf2* mutant, and AtARF1 might act with AtARF2 to control aspects of maturation and senescence (Ellis *et al.*, 2005). Arabidopsis plants with loss of function of *AtARF3* displayed defects in gynoecium development (Nishimura *et al.*, 2005). Mutation of the *AtARF5* gene caused abnormal vascular strand formation and influenced the development of the embryo axis (Hardtke and Berleth, 1998). The *arf7* mutant showed normal responses to auxin in the root, but increased sensitivity to blue light in the hypocotyl in Arabidopsis (Harper *et al.*, 2000). Loss of function of *AtARF8* was found to disrupt auxin homeostasis and affect fruit development and hypocotyl elongation (Goetz *et al.*, 2006). The *AtARF8* gene was reported to act redundantly with *AtARF6*, which is the target gene of miR167, and *arf6/arf8* double mutants had infertile buds and short stamen filaments in Arabidopsis (Nagpal *et al.*, 2005). *AtARF16* is involved in root cap cell differentiation, and gene expression is regulated by miR160 (Wang *et al.*, 2005). The *arf19* single mutant showed normal auxin responses in the hypocotyl, but reduced sensitivity in the root in Arabidopsis. Interestingly, the *arf19/arf7* double mutant displayed strong auxin resistance and impaired hypocotyl and root development (Narise *et al.*, 2010). In a previous study, transgenic rice expressing an *OsARF1* antisense transcript displayed low vigour, stunted growth, sterility, and curled leaves, suggesting that the *OsARF1* gene is pivotal for reproductive and vegetative development in rice (Attia *et al.*, 2009). Another rice ARF gene, *OsARF16*, was found to control iron and phosphate deficiency responses by regulating auxin redistribution in rice (Shen *et al.*, 2013). In tomato, the *SlARF4* gene is involved in flower development and fruit set, growth, and ripening (Sagar *et al.*, 2013; X. Zhang *et al.*, 2015).

To date, ARFs have been widely characterized in several plant species. There are 19 *ARF* genes in sweet orange (*Citrus sinensis*) (S.B. Li *et al.*, 2015), 24 in *Medicago truncatula* (Shen *et al.*, 2015), 47 in banana (*Musa acuminata* L.) (Hu *et al.*, 2015), 25 in the medicinal model plant *Salvia miltiorrhiza* (Xu *et al.*, 2016), 15 in cucumber (*Cucumis sativus*) (Liu and Hu, 2013), 17 in *Eucalyptus grandis* (Yu *et al.*, 2014), and 17 in *Vitis vinifera* (Wan *et al.*, 2014). *ARF* genes have also characterized in several economically important crops; there are 31 members in maize (*Zea mays* L.) (Wang *et al.*, 2012*a*), 25 in rice (*Oryza sativa* L.) (Wang *et al.*, 2007), and 51 in soybean (*Glycine max* L.) (Ha *et al.*, 2013). Cotton is the world’s most important natural textile seed fibre and is also an important oilseed crop. About 27 million metric tons of cotton seed fibre is utilized worldwide per year. Each cotton seed fibre is a single, phenomenally elongated epidermal cell of the cotton seed coat and, in upland cotton (*Gossypium hirsutum*), which accounts for 90% of the world’s cotton production, the seed fibre generally grows up to 30–40 mm in length. The quality and amount of cotton seed fibre depend mainly on two biological processes: seed fibre initiation [occurring about –3 to 3 days post-anthesis (DPA)], which determines the number of seed fibres present on each ovule; and seed fibre elongation, which determines the final length and strength of each seed fibre (Shi *et al.*, 2006). The accumulation of the plant hormone IAA in the epidermis of cotton ovules was found to increase the number of lint seed fibres, resulting in a significant increase in seed fibre yield (Zhang *et al.*, 2011). However, the mechanism linking auxin signalling to seed fibre growth is largely unknown.

In this study, we identified 56 *GhARF* genes in *G. hirsutum* and investigated their evolutionary relationships as well as gene structures to gain insight into the role of auxin signalling in seed fibre growth. Analysis of gene expression patterns revealed that genes in different subfamilies tend to have different expression patterns: *GhARF2* and *GhARF18* subfamily genes were predominantly expressed during cotton seed fibre initiation, whereas *GhARF6* and *GhARF11* genes were highly expressed in vegetative tissues. We selected one gene from the *GhARF2* and *GhARF18* subfamilies to study their function in detail. Ectopic expression of *GhARF2-1* and *GhARF18-1* in Arabidopsis significantly increased trichome initiation. Finally, we identified six potential target genes of *GhARF2-1* and *GhARF18-1*. Our results suggest that *GhARF2* and *GhARF18* subfamily genes may play an important role in regulating cotton seed fibre initiation.

## Materials and methods

### Plant materials

*Gossypium hirsutum* (Xuzhou 142) and the *seed fibreless* (*fl*) mutant, discovered in the same cotton field in China (Zhang and Pan, 1992), were grown in a climate-controlled greenhouse with a 16 h light and 8 h dark cycle at 30 °C as previously reported (Zhang *et al.*, 2017). Fresh cotton seed fibres were excised from bolls at various DPA, and leaves, flowers, stems, and roots were harvested at the indicated time points and then immediately frozen in liquid nitrogen.

### RNA extraction and quantitative real-time (qRT-PCR) analysis

Plant materials frozen in liquid nitrogen were ground to a fine powder with a mortar and pestle using a previously described method (He *et al.*, 2017). Total RNA was prepared using the PureLink™ RNA mini kit (Invitrogen, Lot no. 1687455) as previously described (Liu *et al.*, 2015), and cDNA was reverse-transcribed from 5 μg of total RNA with the Superscript™ first-strand synthesis system (Invitrogen, Carlsbad, CA, USA). In qRT-PCR experiments, each result was obtained from three experiments using independent RNA samples. The reaction parameters were as follows: 95 °C for 5 min, followed by 38 cycles of 95 °C for 15 s and 55 °C for 25 s. Then a melting curve was generated from 65 °C to 95 °C. One-way ANOVA was performed using SigmaStat software. Primers used for qRT-PCR analysis are listed in Supplementary Table S5 at JXB online. The cotton ubiquitin gene *UBQ7* was used as the internal control for each PCR experiment.

### Sequence retrieval and phylogenetic analysis

The cotton and Arabidopsis genome sequences were obtained from the CottonGen website (https://www.cottongen.org) and TAIR 10 (http://www.arabidopsis.org/), respectively (Supplementary Table S6). Other plant genome sequences used in this study were downloaded from Phytozome (https://phytozome.jgi.doe.gov/pz/portal.html). BLASTP with default parameters was used to identify further the ARF proteins with AtARF sequences as the queries based on homology search. The selected cotton ARF proteins were used for further identification of ARFs by searching the cotton database again. All the obtained sequences were sorted as unique sequences, and further protein domain searches were performed using InterProScan (http://www.ebi.ac.uk/Tools/pfa/iprscan). Subsequently, HMMER software with default parameters and conserved ARF domains were used to search for ARF protein sequences. The genomic DNA and cDNA sequences of predicted genes were obtained from the three cotton genomes. Multiple sequence alignment of all identified ARF proteins was performed using ClustalX with default parameters (Thompson *et al.*, 1997). A phylogenetic tree of deduced ARF amino acid or DNA sequences was constructed using the Neighbor–Joining algorithm with default parameters, with 1000 bootstrap replicates in MEGA 5.0 (https://www.megasoftware.net), respectively. The phylogenetic tree was subsequently visualized using TreeView1.6 (http://taxonomy.zoology.gla.ac.uk/rod/treeview.html).

### Gene structure and chromosomal mapping

The Gene Structure Display Server Program (http://gsds.cbi.pku.edu.cn/) was employed to draw the exon–intron structure of *GhARF* genes based on the full-length genome sequence and the corresponding coding sequences obtained as described above. The MEME program was used to identify conserved domains in GhARF proteins (Bailey *et al.*, 2006). Chromosomal position information for all GhARFs was obtained from annotation files downloaded from the CottonGen website. We used Circos to draw the distribution of *GhARF* genes along the chromosomes from the top to the bottom (Zou *et al.*, 2013). The MCScanX software was used to determine and analyse *ARF* gene synteny and collinearity (Wang *et al.*, 2012*b*).

### In vitro ovule culture and treatment with exogenous NAA

Developing cotton seeds were harvested at 1 DPA, soaked in 10% sodium hypochlorite for surface sterilization, and then rinsed eight times in distilled and deionized water. Ovules were excised from bolls, and 30 ovules were placed in a flask containing 20 ml of liquid ovule culture medium. The ovules were cultured at 30 °C in darkness without agitation as previously described (Xiao *et al.*, 2016). Naphthalene acetic acid (NAA; 5 µM) was added to the culture medium for 3, 6, and 24 h. Finally, the cultured ovules were collected and prepared for the qRT-PCR experiment.

### Vector construction, plant transformation, and GUS assay

The full-length *GhARF2-1* and *GhARF18-1* genes were amplified from cDNA by PCR with gene-specific primers listed in Supplementary Table S5. The PCR products were then cloned into the PQG110 vector driven by the constitutive Cauliflower mosaic virus 35S promoter. These constructs were introduced into *Agrobacterium tumefaciens* strain GV3101 and subsequently transformed into Arabidopsis plants (Columbia ecotype) using the floral dip method (Y. Zhang *et al.*, 2015). Transgenic plants were selected on solid half-strength Murashige and Skoog (MS) medium plates containing 50 µg ml^–^1 chloramphenicol. The selected transgenic seedlings were further validated by genomic PCR. Histochemical β-glucuronidase (GUS) assays were performed as previously described (Wang *et al.*, 2004). Samples were stored in 70% ethanol before microscopic observation.

### IAA content analysis

IAA extraction was performed as previously described (Zhang *et al.*, 2011). Cotton ovules harvested at –3, 0, and 3 DPA from the wild type (WT) and the *fl* mutant were immediately frozen and ground into powder in liquid nitrogen. IAA was extracted with 80% (v/v) methanol with 10 ng of [^13^C6]IAA as the internal standard. The supernatant was evaporated under vacuum, dissolved in water, and further purified on Sep-Pak Plus tC18 cartridges (Waters). The eluant was dried and then stored at −20 °C. IAA was analysed according to a previously described method (Gou *et al.*, 2010).

### Scanning electron microscopy

The observation of 0 DPA cotton ovules from the WT and the *fl* mutant was performed using a previously published method (Zhang *et al.*, 2010). Ovule samples were dehydrated for 30 min each in a series of ethanol solutions (30, 50, 70, 90, and 10%), and then sputter-coated with silver using an iron sputter (Hitachi E-1020). The samples were observed using a scanning electron microscope (Hitachi S-3000N).

### Yeast one-hybrid assay

The yeast one-hybrid (Y1H) assay was performed as described (Xu *et al.*, 2017). Briefly, the ORFs of *GhARF2-1* and *GhARF18-1* were each constructed into the pGADT7 vector. The promoter sequences of 11 transcription factors were each inserted into the pHIS2 vector. Yeast strain (Y187) cells were transformed with a pGADT7 prey vector carrying the ORF of *GhARF2-1* or *GhARF18-1* and a constructed pHIS2 bait vector described above. The transformed yeast cell suspension was dropped on SD [DDO for –Trp/–Leu, and TDO for –Trp/–Leu/–His with or without 3-amino-1,2,4-triazole (3-AT)] medium plates and cultured at 30 °C for 5 d.

### Accession number

The transcriptome data we used are available in the NCBI Sequence Read Archive (SRA) under accession number SRA180756.

## Results

### Identification of ARF genes in cotton

To identify *ARF* genes in *G. hirsutum*, we used 22 ARF protein sequences from Arabidopsis as queries in searches against the *G. hirsutum* genome database (F. Li *et al.*, 2015; T. Zhang *et al.*, 2015). After that, the screened GhARFs were used as queries to identify further other possible ARF proteins in *G. hirsutum*. All the candidate GhARF proteins from these step selections were subjected to further selection based on the presence of a conserved ARF domain identified using HMMER software. A total of 56 ARF protein-encoding genes were identified in *G. hirsutum*, including 27 genes originating from the A_t_ subgenome (where ‘t’ stands for tetraploid) and 29 from the D_t_ subgenome (Supplementary Table S1). The same methods were used to identify *ARF* genes in the whole-genome sequences of *Gossypium arboreum* and *Gossypium raimondii* (K. Wang *et al.*, 2012; Paterson *et al.*, 2012; Li *et al.*, 2014). Each of these diploid cotton species contains 35 *ARF* genes. Notably, there are <70 *ARF* genes in allotetraploid *G. hirsutum*, indicating that gene loss occurred after polyploidization.

### Phylogenetic analysis of the *ARF* gene family

**Fig. 1.**
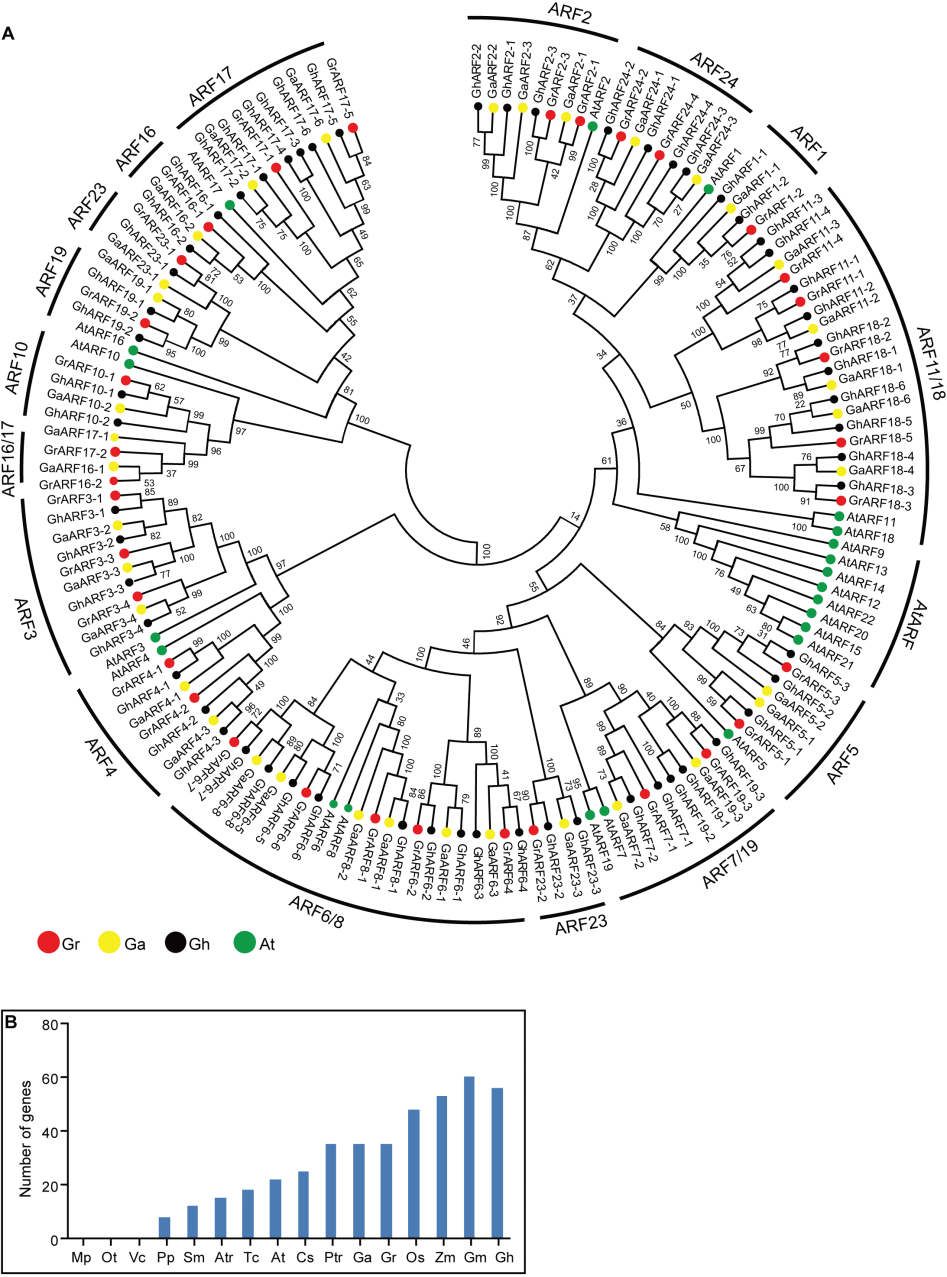
Phylogenetic and evolutionary analysis of the *ARF* gene family in different plant species. (A) An unrooted phylogenetic tree of *ARF* DNA sequences from *Arabidopsis thaliana*, *Gossypium arboreum*, *Gossypium raimondii*, and *Gossypium hirsutum*. The phylogenetic tree was constructed using *ARF* DNA sequences and the Neighbor–Joining (NJ) method in MEGA 5.0 software. (B) Comparisons of *ARF* gene number across a wide range of plant species. Mp, *Micromonas pusilla*; Ot, *Ostreococcus tauri*; Vc, *Volvox carteri*; Pp, *Physcomitrella patens*; Sm, *Selaginella moellemdorffii*; Atr, *Amborella trichopoda*; Tc, *Theobroma cacao*; Cs, *Cucumis sativus*; Ptr, *Populus trichocarpa*; At, *A. thaliana*; Os, *Oryza sativa Japonica*; Gm, *Glycine max*; Zm, *Zea mays*; Ga, *G. arboretum*; Gr, *G. raimondii*; Gh, *G. hirsutum*.

To investigate the evolutionary relationships between *ARF* genes, the ARF DNA or protein sequences from Arabidopsis, *G. arboreum*, *G. raimondii*, and *G. hirsutum* were used to generate an unrooted phylogenetic tree. *ARF* genes were classified into 17 subfamilies, and most of the orthologous genes between the allotetraploid and the corresponding diploids were clustered into the same clade based on phylogenetic analysis with *ARF* DNA sequences (Fig. 1A). According to the phylogenetic tree generated from ARF protein sequences, the protein sequences were divided into 16 subfamilies (Supplementary Fig. S1), which showed a similar result to gene analysis. Subsequently, we explored the evolutionary relationships of *ARF* genes with the phylogenetic tree generated from DNA sequences. Only one *ARF6* and one *ARF18* gene were found in Arabidopsis. However, the *ARF6* and *ARF18* subfamily genes have extensively expanded in *G. hirsutum*, which contained eight and six members, respectively. Interestingly, *ARF9*, *ARF12*, *ARF13*, *ARF14*, *ARF15*, *ARF20*, *ARF21*, and *ARF22* were present in Arabidopsis but were absent from the three cotton species, indicating that these genes may have been acquired in Arabidopsis after divergence from the common ancestor of Arabidopsis and *Gossypium*. We searched for *ARF* genes in 16 species ranging from lower aquatic plants to higher terrestrial plants to determine the origin of the *ARF* gene. Based on this analysis, an *ARF* gene is present in *Physcomitrella patens*, but not in the algae *Micromonas pusilla*, *Ostreococcus tauri*, and *Volvox carteri* (Fig. 1B), suggesting that the *ARF* gene might have originated in moss. Notably, the number of *ARF* genes has dramatically increased in important economic crops.

### Gene structure, conserved motif, and chromosomal location analysis

**Fig. 2.**
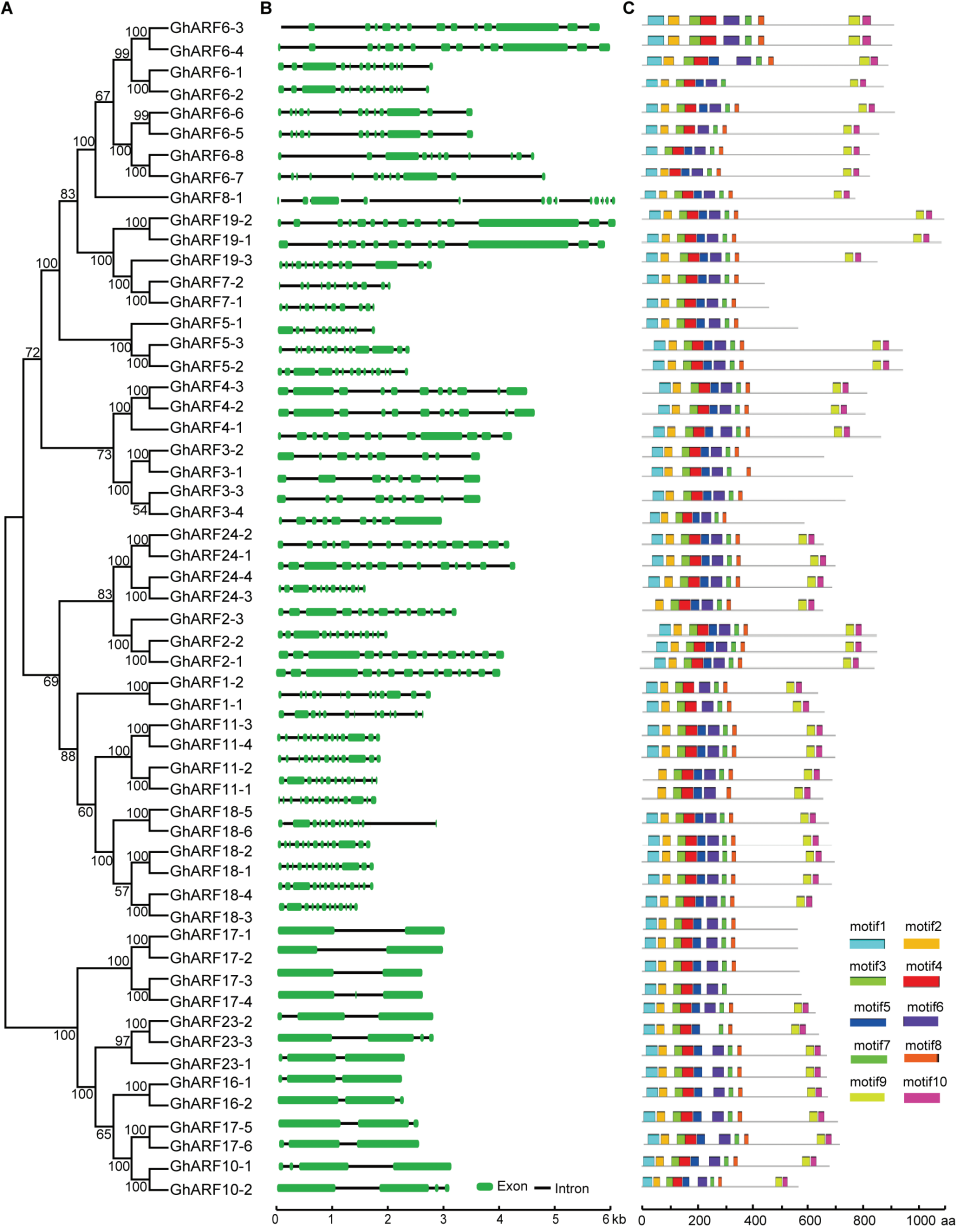
Phylogenetic, gene structure, and motif analyses of *GhARF* genes. (A) Phylogenetic relationships between GhARFs. The phylogenetic tree (left panel) was constructed with MEGA 5.0 using the Neighbor–Joining (NJ) method with 1000 bootstrap replicates. (B) Gene structure analysis of *GhARF* genes. Gene structure maps were drawn with the Gene Structure Display Server 2.0 (Hu *et al.*, 2015). The scale bar is shown at the bottom. (C) Motif analysis of *GhARF* genes. All motifs were identified by MEME software (http://meme-suite.org/). Lengths of each motif are shown proportionally.

To better understand the evolutionary relationships between different members of the *GhARF* gene family, we constructed a separate unrooted phylogenetic tree with *GhARF* DNA sequences (Fig. 2A) and performed a comparative analysis of intron–exon structure. We found that *GhARF* gene length varied significantly, with *GhARF18-1* having the longest (6.1 kb) and *GhARF18-4* having the shortest (1.4 kb) genomic sequence. A highly rich distribution of introns was found in the *GhARF* gene regions. In general, *GhARF* genes possessed at least two exons and could be divided into two categories based on exon number (Fig. 2B). Forty-three genes had >10 exons, and 13 genes had 2–4 exons. Notably, closely related genes had more similar arrangements of exons and introns, indicating that exon–intron structure and the phylogenetic relationship between *GhARF* genes are highly correlated. Conserved motifs in the GhARF proteins were identified using MEME software. In total, 10 conserved motifs, named motif 1 to motif 10, were identified in GhARFs, and the number of conserved motifs in each GhARF varied from 7 to 10 (Fig. 2C). Most members had motifs 1, 2, 3, 4, 5, 6, 7, and 8, indicating that they are highly conserved among GhARFs.

**Fig. 3.**
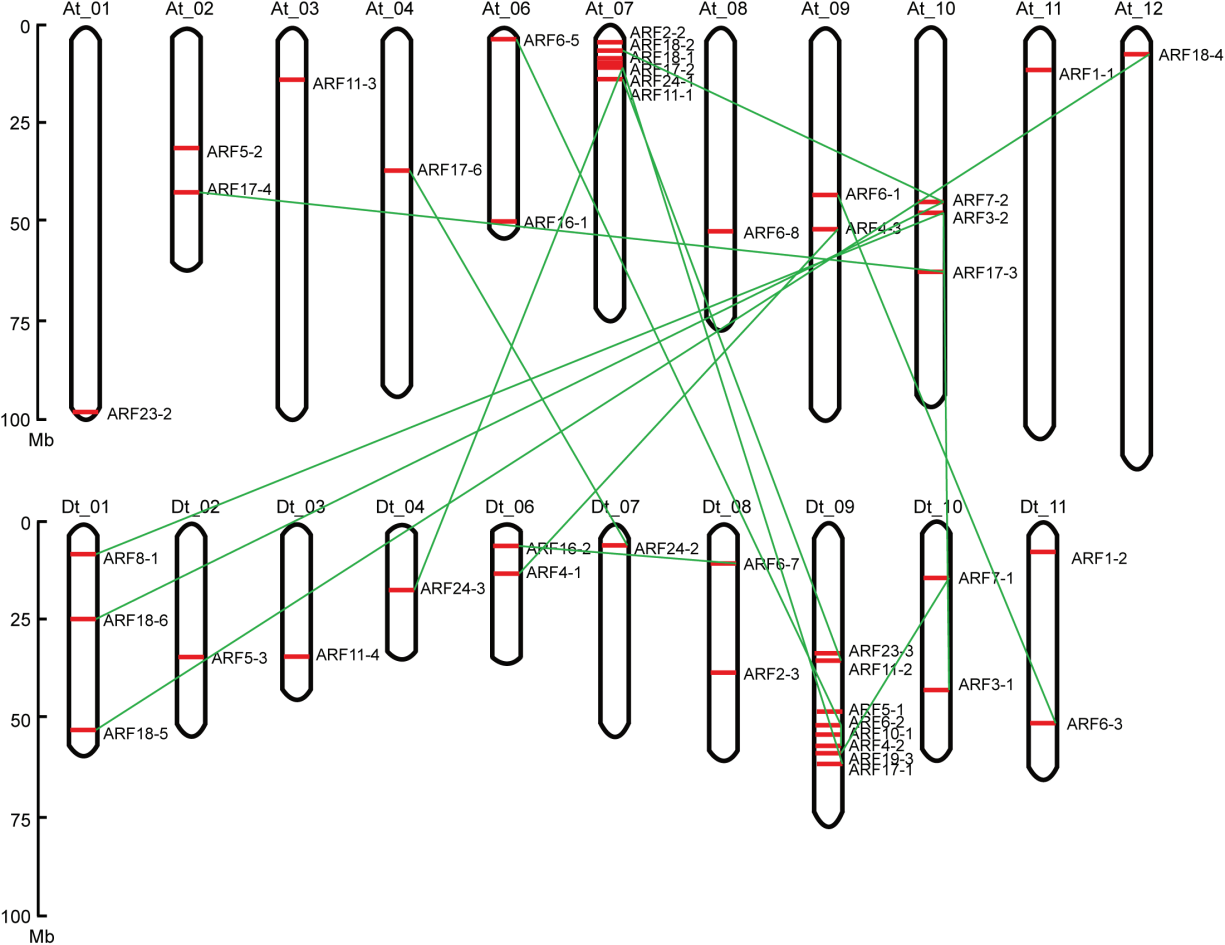
Chromosomal distribution and gene duplication of *GhARF* genes. The genome visualization tool CIRCOS was used to illustrate the chromosomal distribution of *GhARF* genes. The chromosome number is shown above each chromosome. The chromosomal location of each *GhARF* is shown from the top to the bottom of the corresponding chromosome. Duplicated gene pairs are linked by lines. The scale bar beside the chromosome indicates the length in megabases (Mb).

Using the complete *G. hirsutum* genome sequence (F. Li *et al.*, 2015; T. Zhang *et al.*, 2015), we investigated the chromosomal locations of *GhARF* genes. A total of 44 genes were distributed throughout the 21 chromosomes comprising 11 located on the A_t_ subgenome and 10 located on the D_t_ subgenome (Fig. 3). The highest numbers of *GhARF* genes were located on chromosome 7 from the A_t_ subgenome (six *GhARF* genes) and chromosome 9 from the D_t_ subgenome (eight *GhARF* genes). The other *GhARF* genes were found to be located in the scaffolds (Supplementary Table S2). We further investigated the evolutionary history of *GhARF* genes by conducting genome synteny analysis. Based on syntentic relationships, there are 15 pairs of putative paralogous *GhARF* genes. Forty-nine *GhARF* genes were produced by whole-genome duplication (WGD) and seven were produced by singleton duplication (Supplementary Table S3). The high proportion of *GhARF* genes derived from WGD indicates that these duplication events played a crucial role in *GhARF* gene expansion in the *G. hirsutum* genome.

### *GhARF2* and *GhARF18* genes are expressed in ovules during fibre cell initiation

**Fig. 4.**
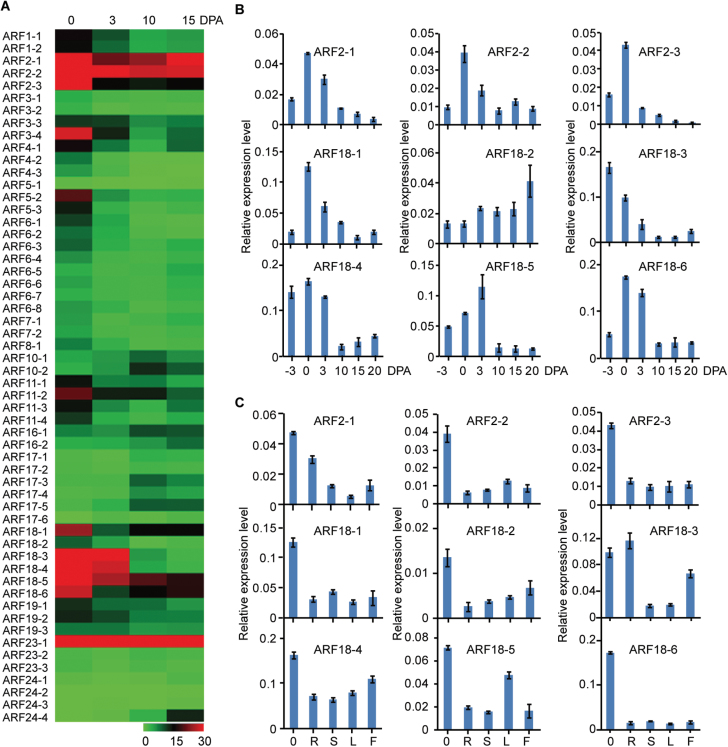
Analysis of *GhARF* expression patterns during seed development and seed fibre initiation. (A) Heat map of the expression level of 56 *GhARF* genes during different stages of seed fibre growth based on RNA-seq data. (B) Expression profiling of seed and seed fibre initiation-specific *GhARF2* and *GhARF18* subfamily genes during seed and seed fibre development. Gene expression data were obtained by quantitative real-time PCR with three independent replicates. (C) Expression profiling of *GhARF2* and *GhARF18* subfamily genes in various tissues. 0, R, S, L, and F represent ovules at 0 DPA, root, stem, leaf and flower, respectively. Gene expression data were obtained by quantitative real-time PCR with three independent replicates.

To determine which *GhARF* genes potentially function in seed fibre development, the expression patterns of individual members were investigated using transcriptome data for different stages of *G. hirsutum* seed fibre and ovule development. The majority of *GhARF* genes from the same subfamily had similar expression patterns. Notably, all members of the *GhARF2* and *GhARF18* subfamilies, except for *GhARF18-2*, were highly expressed in the 0 DPA ovules (Fig. 4A). In addition, *GhARF3-4*, *GhARF11-2*, and *GhARF23-1* genes were dramatically expressed in 0 DPA ovules. These results suggest that these genes may function in cotton seed fibre cell initiation. The similar expression patterns of these genes suggest that they may have redundant functions, especially given that within each subfamily genes share a high degree of protein sequence identity. *GhARF2-1* shares 98% identity with *GhARF2-2* and 84% identity with *GhARF2-3*, and there is 98% identity between *GhARF18-1* and *GhARF18-2*, 93% identity between *GhARF18-3* and *GhARF18-4*, and 99% identity between *GhARF18-5* and *GhARF18-6*. Genes from the *GhARF2* subfamily were highly expressed in 0–15 DPA seed fibre cells, indicating that these genes play an important role in both seed fibre cell initiation and cell elongation. The transcript levels of *GhARF2* and *GhARF18* in seed fibre cells and vegetative tissues were analysed using qRT-PCR. We found that all *GhARF2* and *GhARF18* genes, except for *GhARF18-2*, were predominantly expressed in –3, 0, or 3 DPA cells which corresponds to seed fibre initiation (Fig. 4B, C). *GhARF18-3* was highly expressed in both roots and seed fibre cells at the initiation stage (Fig. 4C).

**Fig. 5.**
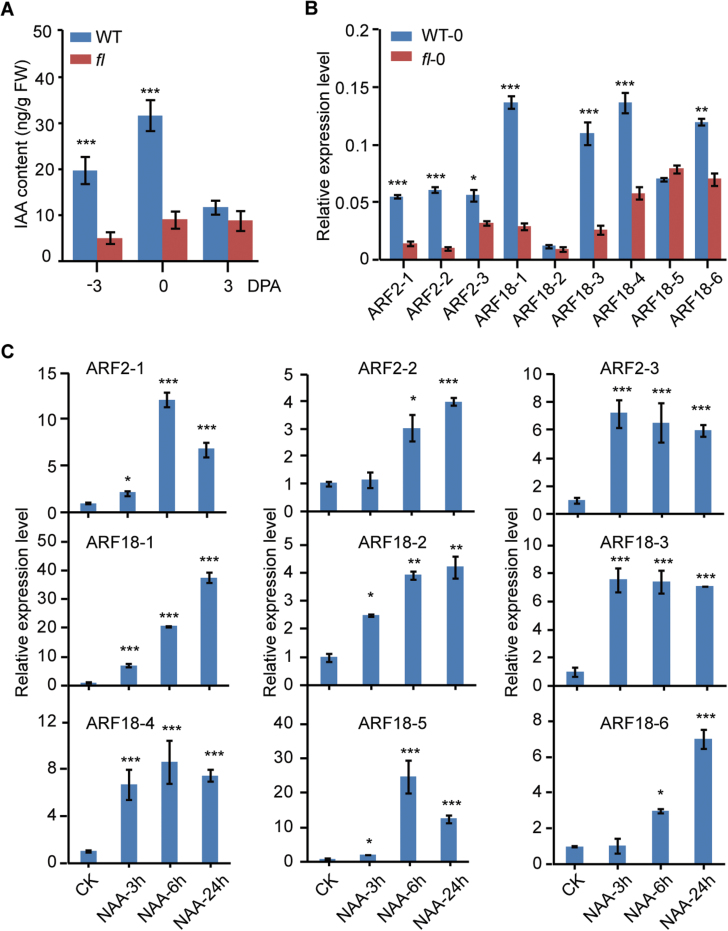
*GhARF2* and *GhARF18* genes are responsive to auxin in fibre cell initiation. (A) Endogenous IAA levels in wild-type and *fl* mutant ovules during seed fibre initiation. The bar represents the SD for three samples. (B) The expression level of *GhARF2* and *GhARF18* subfamily genes in wild-type and *fl* mutant ovules at 0 DPA. (C) Exogenous NAA promotes the transcription of *GhARF2* and *GhARF18*. Relative expression levels of each gene were determined after normalizing to the expression level in CK ovules, which was set to 1.0. Statistical signiﬁcance was determined using one-way ANOVA combined with Tukey’s test. *P<0.05; **P<0.01; ***P<0.001.

To assess whether auxin plays an important role in seed fibre initiation, we quantified IAA accumulation and determined the expression levels of the *GhARF2* and *GhARF18* genes in the WT and the *fl* mutant. Large numbers of initiated seed fibre cells were observed in WT ovules, whereas no seed fibre cell initiation and differentiation was observed on the surface of *fl* ovules at 0 DPA (Supplementary Fig. 2). We further analysed the free IAA content in –3, 0, and 3 DPA ovules by LC and MS. A significant increase in IAA level was found in WT ovules relative to *fl* ovules, especially in –3 and 0 DPA ovules (Fig. 5A). On average, IAA levels in WT ovules were 298% of the level in *fl* ovules. The expression level of all *GhARF2* and *GhARF18* subfamily members, except for *GhARF18-2* and *GhARF18-5*, was significantly higher in WT ovules than in *fl* ovules (Fig. 5B). The transcript levels of *GhARF2* and *GhARF18* genes increased significantly after the application of 5 µM NAA for 6 h (Fig. 5C).

Taken together, our results suggest that *GhARF2* and *GhARF18* genes may play important roles in cotton seed development and seed fibre cell initiation. In addition, auxin may enhance the expression of *GhARF2* and *GhARF18* genes to promote seed fibre cell initiation.

### Overexpression of *GhARF2-1* and *GhARF18-1* stimulates trichome initiation in Arabidopsis

**Fig. 6.**
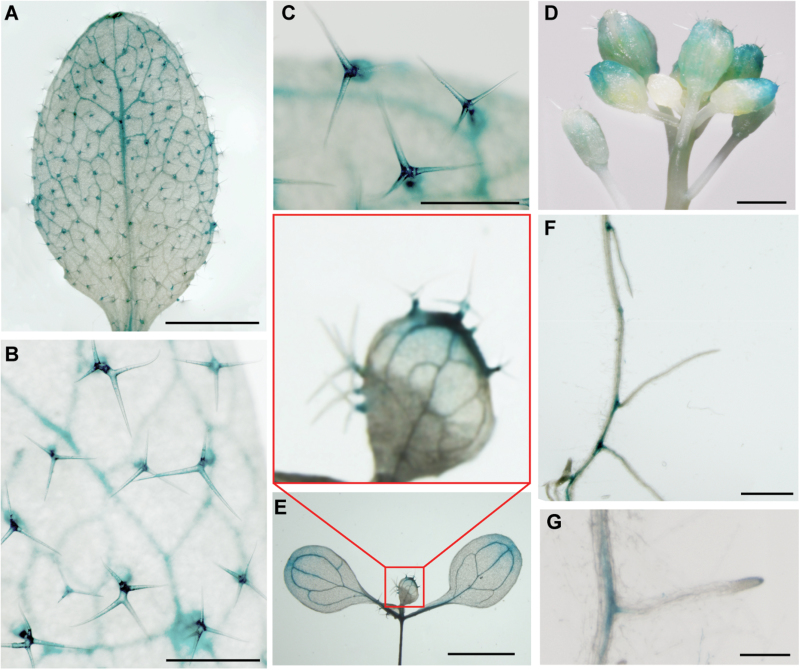
GUS staining assay of the *GhARF2-1* and *GhARF18-1* gene promoters. (A–D) GUS staining of 14-day-old Arabidopsis seedlings expressing *P*_*GhARF2-1*_*:GUS*. GUS staining was observed in the base of the trichome, leaf veins, and sepals of flowers. Scale bars=2.5 mm in (A), 0.5 mm in (B), 0.25 mm in (C), and 1 mm in (D). (E–G) GUS staining of 7-day-old Arabidopsis seedlings expressing *P*_*GhARF18-1*_*:GUS*. GUS staining was observed in the base of the trichome (E) and emerging lateral roots (F,G). Insert in (E) shows a magnified leaf. Scale bars=5 mm in (E), 1 cm in (F), and 2.5 mm in (G).

Trichomes in Arabidopsis are organs similar to cotton seed fibres (Zhang *et al.*, 2018). Based on the analysis of RNA expression patterns, *GhARF2* and *GhARF18* genes are mainly expressed during seed development and seed fibre cell initiation. To investigate further the possible expression region of *GhARF2* and *GhARF18* genes, we selected *GhARF2-1* and *GhARF18-1* as examples and detected GUS activities in *P*_*GhARF2-1*_*:GUS* and *P*_*GhARF18-1*_*:GUS* Arabidopsis seedlings. *GhARF2-1* promoter-driven GUS expression was mainly observed in the base of trichomes in the leaf epidermis, leaf veins, and sepals of flowers (Fig. 6A–D), and *GhARF18-1* promoter-driven GUS expression was present exclusively in the base of trichomes in the leaf epidermis and emerging lateral roots (Fig. 6E–G).

**Fig. 7.**
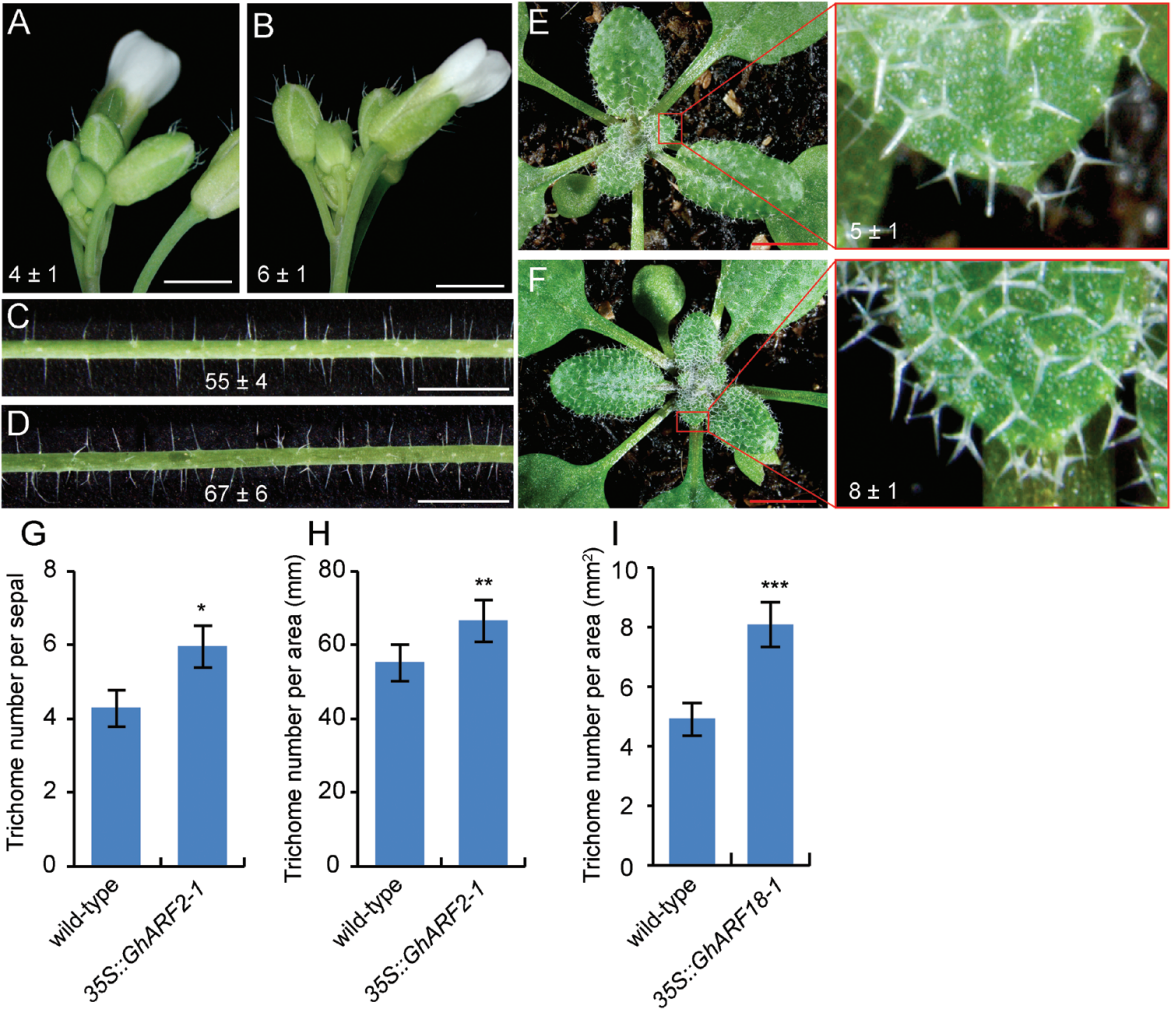
Phenotypes of *35S:GhARF2-1* and *35S:GhARF18-1* transgenic Arabidopsis plants. (A) Trichome initiation on the sepals of wild-type flowers. (B) Trichome initiation on the sepals of *35S:GhARF2-1* flowers. (C) Trichomes on the main inflorescence stems of wild-type plants. (D) Trichomes on the main inflorescence stems of *35S:GhARF2-1* transgenic plants. (E, F) Comparison of the densities of trichomes in the sepal (E) and the main inflorescence stems (F) from the wild-type and *35S:GhARF2-1* transgenic plants. Each experiment was performed in three biological replicates, and the error bars represent the mean ±SE. *P<0.05; **P<0.01. (G) Trichomes on the leaves of wild-type plants. (H) Trichomes on leaves of *35S:GhARF18-1* plants. (I) Comparison of the densities of trichomes in the leaf from the wild-type and *35S:GhARF18-1* transgenic plants. Each experiment was performed in three biological replicates, and the error bars represent the mean ±SE. ***P<0.001. Scale bars=2.0 mm.

To explore further the functions of *GhARF2* and *GhARF18*, we generated two transgenic Arabidopsis overexpression lines with *GhARF2-1* or *GhARF18-1* expression driven by the constitutive 35S promoter, and observed the trichomes, which are similar to cotton seed fibres. Compared with WT plants, the transgenic plants overexpressing *GhARF2-1* showed an increase in the number of trichomes on the sepals of flowers (Fig. 7A, B) and main inflorescence stems (Fig. 7C, D). The density of trichomes per area in the *35S:GhARF2-1* transgenic lines increased to 141% and 121% of the level in the sepals of flowers and main inflorescence stems of WT plants, respectively (Fig. 7E, F). Moreover, *35S:GhARF18-1* transgenic plants had more trichomes on the leaves than WT plants (Fig. 7G, H). The number of trichomes in the *35S:GhARF18-1* lines increased to 164% of the level in the leaves of WT plants (Fig. 7I). These results indicate that there was some influence of these cotton genes on trichome initiation in the Arabidopsis transgenic plants.

### Identification of potential transcription factors regulated by *GhARF*

**Fig. 8.**
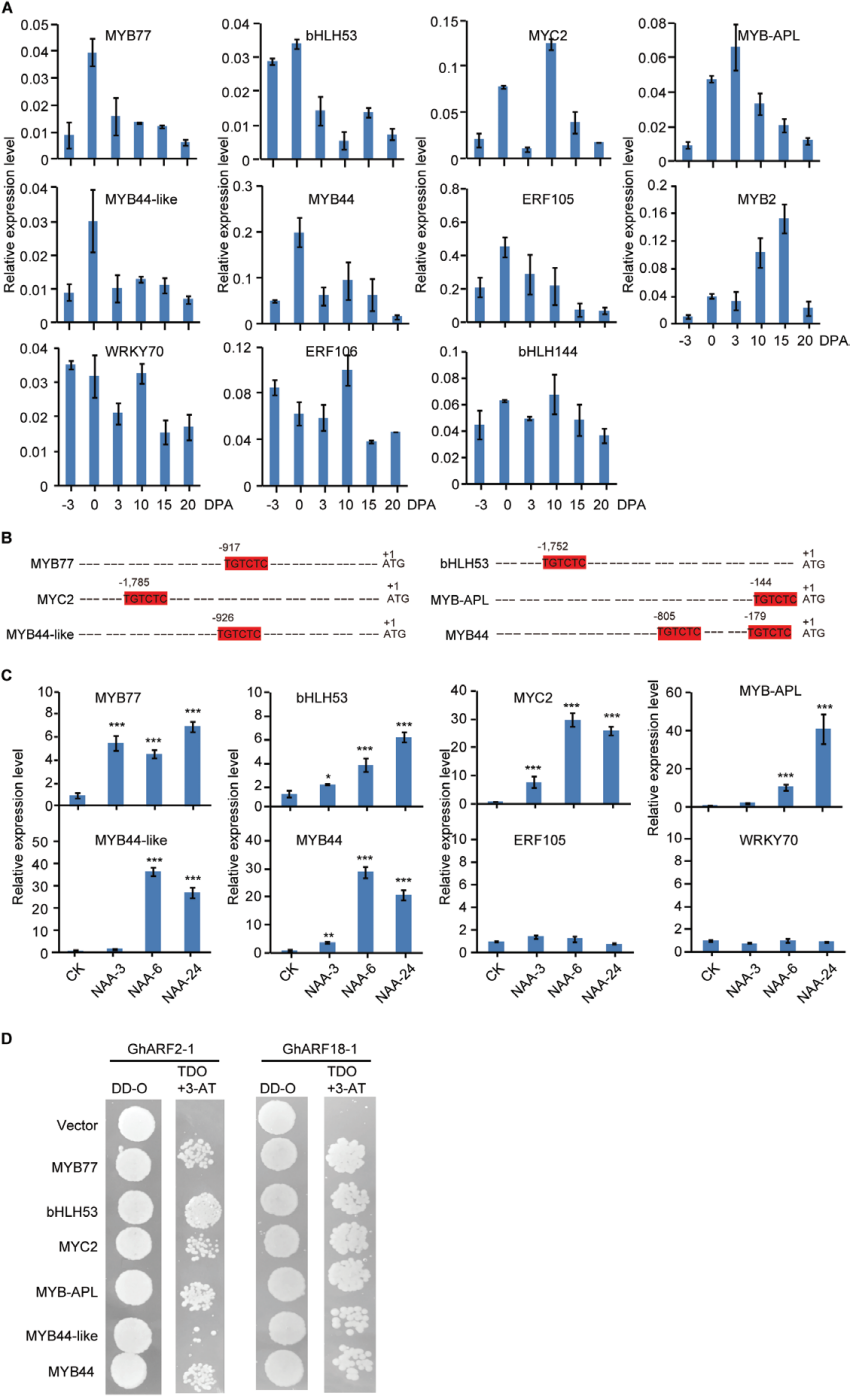
Validation and expression analysis of target transcription factor genes. (A) Expression profiling of 11 target transcription factors during different stages of seed fibre growth. Target genes up-regulated in 0 DPA ovules and containing the auxin-responsive element (AuxRE) in their promoter regions were selected. Gene expression data were obtained by quantitative real-time PCR with three replicates, with each replicate performed using independent materials. (B) Promoter sequence analysis of six transcription factor genes selected based on high expression levels during seed fibre initiation shown in (A). (C) NAA significantly induced the transcription of the six transcription factor genes. RNA samples were prepared from three independent cultures of ovules treated with 5 µM NAA for the indicated time. Relative expression levels of each gene were determined by normalizing to the expression level in CK ovules, which was set to 1.0. Statistical signiﬁcance was determined using one-way ANOVA combined with Tukey’s test. *P<0.05; **P<0.01; ***P< 0.001. (D) Yeast one-hybrid assay. DD-O, yeast medium lacking leucine and tryptophan. TDO, yeast medium lacking leucine, tryptophan, and histidine. 3-AT, 3-amino-1,2,4-triazole.

ARFs, which are essential components of auxin signalling pathways, bind specifically to the AuxRE to regulate the expression of target genes (Cho *et al.*, 2014). In order to identify the potential transcription factors directly regulated by *GhARF*, we screened for transcription factors that were both highly expressed during the seed fibre initial stage and had at least one AuxRE in their promoter. First, we calculated the gene expression level in 0 DPA and 10 DPA samples. We found 3624 genes whose expression level at 0 DPA was over twice that at 10 DPA. We regarded these genes as high expression genes in 0 DPA. Secondly, we investigated the genes which contained at least one AuxRE in their promoter. As a result, we found 21 386 genes in which AuxRE elements were present in their promoter region. Finally, we picked up 11 transcription factors which belong to high expression genes in 0 DPA and contain an AuxRE in their promoter (Supplementary Fig. S3; Supplementary Table S4). qRT-PCR analysis confirmed that six genes, *MYC2*, *MYB44*, *MYB44-like*, *MYB*-*APL*, *MYB77*, and *bHLH53*, were highly expressed in ovules at the initiation stage (Fig. 8A). *MYB2* expression peaked at 15 DPA, when transcript levels were ~15-fold higher than at 0 DPA. There was no obvious difference in *ERF105*, *WRKY70*, *ERF106*, and *bHLH144* expression in seed fibre cells at different developmental stages. The promoter regions of each of the six transcription factor genes preferentially expressed during seed fibre initiation were found to contain at least one AuxRE (Fig. 8B). The other transcription factor genes were found to contain one AuxRE in their promoter regions, except for that of *ERF106* gene, which contained two AuxREs in the promoter regions (Supplementary Fig. S4). When ovules were incubated for several hours in ovule culture medium containing 5 µM NAA, an analogue of auxin, the transcripts of these genes increased significantly (Fig. 8C). For *MYC2*, *MYB44*, and *MYB44-like*, a >20-fold increase in transcript level was observed after 6 h treatment with NAA. An ~40-fold increase was observed for *MYB*-*APL* after NAA treatment for 24 h. NAA also significantly induced the expression of *MYB77* and *bHLH53*. In contrast, application of NAA did not enhance the expression of *ERF105* and *WRKY70*, which do not have an AuxRE in their promoters. Consistent with this, GhARF2-1 and GhARF18-1 could bind to the promoters of these genes in Y1H assays using target gene promoters as baits and GhARF2-1 or GhARF18-1 as prey (Fig. 8D). Interestingly, GhARF2-1 could bind to the *MYB2* promoter region and GhARF18-1 could bind to the *WRKY70*, *ERF106*, and *bHLH144* promoter regions (Supplementary Fig. S5). Taking into consideration the gene expression pattern during cotton seed fibre initiation, we suggest that GhARF2 and GhARF18 may regulate the expression of *MYC2*, *MYB44*, *MYB44-like*, *MYB*-*APL*, *MYB77*, and *bHLH53* via binding to the AuxRE element in their promoters during cotton seed fibre initiation.

## Discussion

During the past few years, the whole-genome sequences of three cotton species have been completed (K. Wang *et al.*, 2012; Paterson *et al.*, 2012; F. Li *et al.*, 2014, 2015; T. Zhang *et al.*, 2015), which has greatly promoted the development of cotton science. Cotton seed fibres are single-celled trichomes that differentiate from the ovule epidermis (Basra and Malik, 1984). The development of cotton seed fibres is divided into four well-defined and overlapping stages, namely initiation, elongation, secondary cell wall thickening, and seed fibre maturation (Yoo and Wendel, 2013). Phytohormones, such as auxin, gibberellins, and ethylene, play important roles in seed fibre development (Beasley, 1973; Zhang *et al.*, 2011; Shi *et al.*, 2006). Application of gibberellin (GA_3_) to the flower buds in the field was found to increase the seed fibre number per ovule greatly (Seagull and Gialvalis, 2004). GhHOX3, a homeodomain transcription factor, interacts with GhSLR1, a cotton DELLA protein, to promote seed fibre cell elongation (Shan *et al.*, 2014). Exogenous application of IAA, the most important natural auxin, to an *in vitro* ovule culture system significantly increased seed fibre initiation and the number of seed fibre units (Gialvalis and Seagull, 2001). Ectopic expression of the auxin biosynthetic gene *iaaM* increased IAA levels and the number of lint seed fibres (Zhang *et al.*, 2011). ARFs, key components of auxin signalling pathways, can directly bind to downstream target gene promoters by recognizing TGTCTC AuxREs and regulate gene expression (Cho *et al.*, 2014).

In this work, we simultaneously identified *ARF* genes in three representative cotton species, upland cotton *G. hirsutum* and its two diploid ancestors, *G. arboreum* and *G. raimondii*. We identified 56 *ARF* genes in *G. hirsutum*, and 35 *ARF* genes each in *G. arboreum* and *G. raimondii*. These results indicate that *ARF* gene loss occurred in *G. hirsutum*, which is consistent with the higher rate of gene loss in allotetraploid cotton than in both diploid species (F. Li *et al.*, 2015; T. Zhang *et al.*, 2015). *ARF6* and *ARF18* subfamily genes have extensively expanded in *Gossypium*, indicating that genes in these two subfamilies may contribute to better agronomic traits in cotton. The role of these genes in agronomically important stages of development is supported by the fact that *ARF18* genes are expressed during seed fibre cell initiation. Some *ARF* genes were only found in Arabidopsis, but lost in *Gossypium*, suggesting that gene loss has occurred since Arabidosis and *Gossypium* diverged from their common ancestor. Most *GhARF* genes possess >10 exons, whereas four *GhARF* genes each have no more than four exons (Fig. 2B). This indicates that *ARF* family genes may be regulated by two different mechanisms. There is increasing evidence that introns play important roles in alternative splicing and generation of non-coding RNAs (Rearick *et al.*, 2011). A similar phenomenon was observed for Arabidopsis *ARF* genes, suggesting that these regulatory mechanisms are relatively conserved in plants.

The expression patterns of *ARF* genes provided insight into their potential functions. Transcription profiling showed that the *GhARF2* and *GhARF18* subfamily genes were highly expressed in 0 DPA ovules (Fig. 4). In Arabidopsis, the *arf2* loss-of-function mutant displayed many defects including enlarged rosette leaves, reduced fertility, delayed senescence, hypocotyl elongation defects, enlarged seeds, and enlarged cotyledons (Ellis *et al.*, 2005). The functions of the *ARF18* and *ARF11* genes are still unknown, and loss of function of *ARF18* results in no obvious phenotype in Arabidopsis. ARF6 may act redundantly with ARF8 to control stamen elongation and flower maturation in tomato (Liu *et al.*, 2014). MacMillan and his colleagues found that two auxin-responsive-related genes was seed fibre specifically expressed; one of these has moderate expression at 7 DPA and the highest transcript level at 15 DPA and the other was exclusively expressed at 15 DPA, indicating that auxin-responsive-related genes might be involved in secondary cell wall synthesis at the eed fibre elongation (MacMillan *et al.*, 2017). Our results indicate that *GhARF2* and *GhARF18* genes play important roles in cotton seed fibre initiation; the evidence is as follows. The transcript level of most *GhARF2* and *GhARF18* genes was significantly higher in WT ovules than in *fl* ovules (Fig. 4E). GUS activity driven by the *GhARF20-1* and *GhARF18-1* promoters was present exclusively in trichomes (Fig. 6), and ectopic expression of *GhARF2-1* and *GhARF18-1* in Arabidopsis resulted in a considerable increase in the number of trichomes (Fig. 7).

As central repressors in the auxin pathway, Aux/IAA proteins can interact with the co-repressor TPL to prevent activation of auxin-responsive genes by activating ARFs (Leyser, 2018). Overexpression of Arabidopsis transcription factors RAV1 and RAV2, which could physically interact with TPL, significantly increased the number of longer fibres in cotton (Mittal *et al.*, 2015). In addition, TPL was reported to mediate brassinosteroid-induced transcriptional repression through interaction with BZR1 (Oh *et al.*, 2014). Brassinosteroid played an important role in seed fibre development, and application of brassinosteroid *in vitro* significantly promoted cotton seed fibre cell elongation (Zhou *et al.*, 2015). We presumed that TPL may mediate the crosstalk between auxin and brassinosteroid via ARF and BZR1 during cotton seed fibre developemt.

In Arabidopsis, trichome formation is modulated by transcription factors, including members of the bHLH, MYB, and MYC families (Schiefelbein *et al.*, 2014). MYB and homeodomain-leucine zipper (GhHOX3) transcription factors have been reported to be involved in cotton seed fibre development (Shan *et al.*, 2014). We found that bHLH53, MYC2, and four MYB transcription factors are potential target genes of GhARF2 and GhARF18 (Fig. 8). Some of these transcription factors have already been shown to regulate the development of seed fibre cells or other types of epidermal cells. GhMYC2 has previously been shown to interact with GhJAZ2 to regulate seed fibre initiation in cotton (Hu *et al.*, 2016), and AtMYC2 is a positive regulator of lateral root formation in Arabidopsis (Raya-González *et al.*, 2017). *apl* mutant seedlings display a short, determinate root with only occasional lateral branches (Abe *et al.*, 2015). It has been reported that GhMYB25 regulates early seed fibre and trichome development, and that GhMYB109 and GaMYB2 are required for cotton seed fibre development (Wang *et al.*, 2004; Pu *et al.*, 2008; Machado *et al.*, 2009). In Arabidopsis, MYB77, which interacts with ARF7, regulates the expression of some auxin-responsive genes and is required for lateral root development (Zhao *et al.*, 2014).

In summary, our data have revealed the potentially important role of *GhARF2* and *GhARF18* subfamily genes in cotton seed fibre cell initiation. Auxin may activate several transcription factors via GhARF2 and GhARF18 proteins to regulate cotton seed fibre cell initiation. In order to understand the molecular mechanisms of seed fibre initiation regulated by auxin, further work will be aimed at characterizing the function of the genes directly targeted by *GhARF2* and *GhARF18*.

## Supplementary data

Supplementary data are available at *JXB* online.

**Fig. S1.** Phylogenetic and evolutionary analysis of the *ARF* gene family in different plant species.

**Fig. S2.** Scanning electron microscopy images of the surface of wild-type and *fl* mutant ovules at 0 DPA.

**Fig. S3.** Venn diagram analysis of potential target transcription factor genes of GhARF. **Fig. S4.** Promoter sequence analysis of five transcription factor genes.

**Fig. S5.** Yeast one-hybrid assay.

**Table S1.** Analysis of *G. hirsutum ARF* gene family and its orthologues in AA and DD cotton genomes.

**Table S2.** Location analysis of 12 *GhARF* genes out of the chromosome.

**Table S3.** Analysis of duplication events in *G. hirsutum ARF* genes located in chromosomes.

**Table S4.** Transcription factor genes up-regulated in 0 DPA ovules and containing an ARF-binding site in their promoter.

**Table S5.** A list of primers used in this study.

**Table S6.** *ARF* gene sequences from the three cottons used in this study.

Supplementary Figures and Tables
