# Supplementary material for: Genome-wide identification of the GhARF gene family reveals that GhARF2 and GhARF18 are involved in cotton fibre cell initiation
Source: J Exp Bot. 2018 Jun 12;69(18):4323–37. doi: 10.1093/jxb/ery219 (PMC6093391; doi:10.1093/jxb/ery219)
Supplement: Supplementary Figures and Tables [file ery219_suppl_supplementary_figures_and_tables.pdf]

Figure S1. Phylogenetic and evolutionary analysis of the *ARF* gene family in different plant species. An unrooted phylogenetic tree of ARF protein sequences from *Arabidopsis thaliana*, *Gossypium arboreum*, *Gossypium raimondii*, and *Gossypium hirsutum*. The phylogenetic tree was constructed using ARF protein sequences and the neighbor-joining (NJ) method in MEGA 5.0 software.

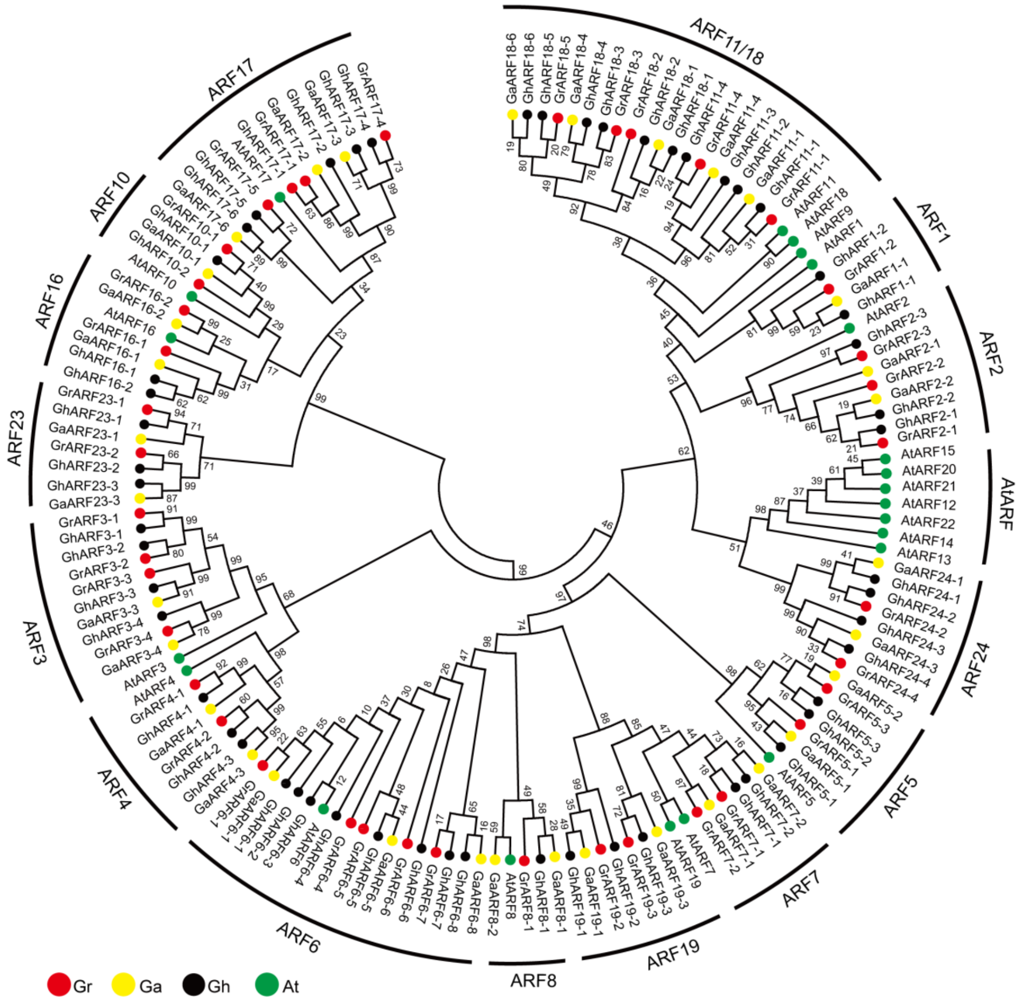

Figure S2. Scanning electron microscopy images of the surface of wild-type and *fl* mutant ovules at 0 DPA.

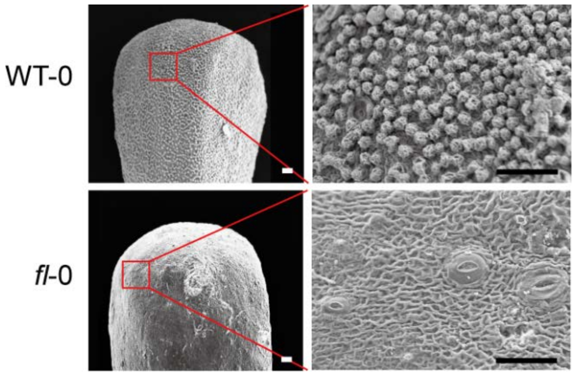

Figure S3. Venn diagram analysis of potential target transcription factor genes of GhARF. For each venn diagram, the circle on the left indicates the number of genes highly expressed in the indicated tissue, and the circle on the right indicates all the *G. hirsutum* genes containing the auxin responsive element (AuxRE) in their promoters. The overlap between the two circles indicates the potential target transcription factor genes of GhARF.

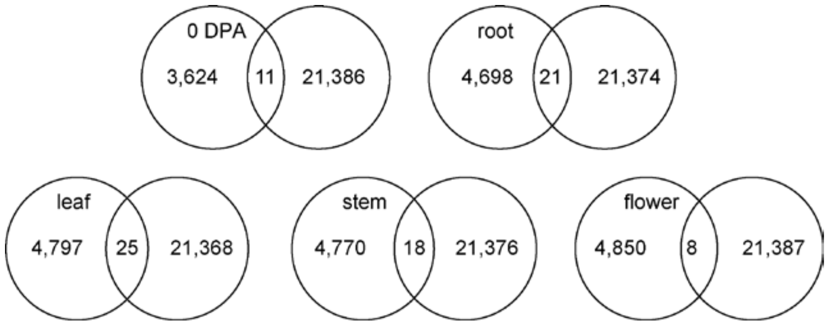

Figure S4. Promoter sequence analysis of five transcription factor genes .

TGTCTC, ARF binding sites.

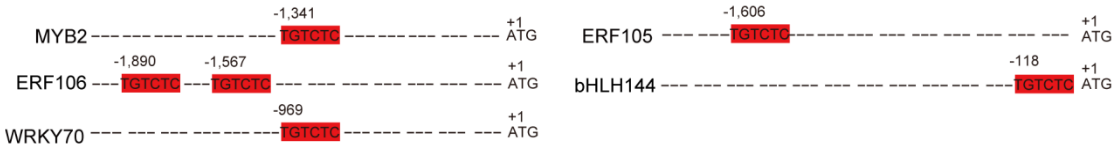

Figure S5. Yeast one-hybrid assay. DDO, yeast medium lacking leucine and tryptophan. TDO, yeast medium lacking leucine, tryptophan and histidine. 3-AT, 3-amino-1,2,4-triazole.

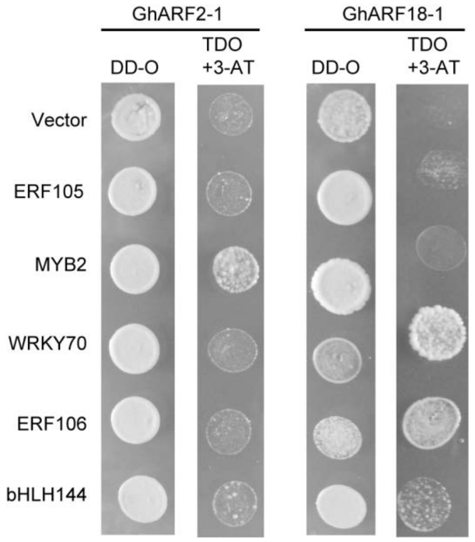

Table S1. Analysis of *G. hirsutum ARF* gene family and its orthologues in AA and DD cotton genomes

Gene ID	Length of amino acid	Gene name	Orthologous	Subgenome	Diploid cotton
CotAD_56239	650	ARF1-1	Cotton_A_31395	At	*G. arboreum*
CotAD_58764	860	ARF2-2	Cotton_A_01955	At	*G. arboreum*
CotAD_72124	684	ARF3-2	Cotton_A_40208	At	*G. arboreum*
CotAD_64060	738	ARF3-3	Cotton_A_11311	At	*G. arboreum*
CotAD_12422	815	ARF4-3	Cotton_A_11048	At	*G. arboreum*
CotAD_13433	563	ARF5-1	Cotton_A_16048	At	*G. arboreum*
CotAD_63258	932	ARF5-2	Cotton_A_27669	At	*G. arboreum*
CotAD_27033	896	ARF6-1	Cotton_A_26156	At	*G. arboreum*
CotAD_40344	908	ARF6-3	Cotton_A_02993	At	*G. arboreum*
CotAD_26127	876	ARF6-5	Cotton_A_14740	At	*G. arboreum*
CotAD_67703	840	ARF6-8	Cotton_A_35443	At	*G. arboreum*
CotAD_57703	432	ARF7-2	Cotton_A_38575	At	*G. arboreum*
CotAD_67896	796	ARF8-1	Cotton_A_16760	At	*G. arboreum*
CotAD_09605	696	ARF10-2	Cotton_A_07064	At	*G. arboreum*
CotAD_02558	680	ARF11-2	Cotton_A_18937	At	*G. arboreum*
CotAD_70430	695	ARF11-3	Cotton_A_36154	At	*G. arboreum*
CotAD_66018	709	ARF16-2	Cotton_A_24047	At	*G. arboreum*
CotAD_31393	579	ARF17-2	Cotton_A_16138	At	*G. arboreum*
CotAD_57862	577	ARF17-3	Cotton_A_18446	At	*G. arboreum*
CotAD_04601	652	ARF17-6	Cotton_A_10518	At	*G. arboreum*
CotAD_57415	692	ARF18-1	Cotton_A_14407	At	*G. arboreum*
CotAD_51593	614	ARF18-4	Cotton_A_25871	At	*G. arboreum*
CotAD_42645	694	ARF18-6	Cotton_A_31049	At	*G. arboreum*
CotAD_29901	1097	ARF19-1	Cotton_A_06701	At	*G. arboreum*
CotAD_08070	675	ARF23-3	Cotton_A_06107	At	*G. arboreum*
CotAD_26342	706	ARF24-1	Cotton_A_08273	At	*G. arboreum*
CotAD_22697	652	ARF24-3	Cotton_A_22543	At	*G. arboreum*
CotAD_15593	655	ARF1-2	Cotton_D_gene_10008576	Dt	*G. raimondii*
CotAD_76410	863	ARF2-1	Cotton_D_gene_10022860	Dt	*G. raimondii*
CotAD_43230	841	ARF2-3	Cotton_D_gene_10003843	Dt	*G. raimondii*
CotAD_65781	780	ARF3-1	Cotton_D_gene_10040816	Dt	*G. raimondii*
CotAD_00650	686	ARF3-4	Cotton_D_gene_10031398	Dt	*G. raimondii*
CotAD_13694	870	ARF4-1	Cotton_D_gene_10015825	Dt	*G. raimondii*
CotAD_54309	812	ARF4-2	Cotton_D_gene_10019010	Dt	*G. raimondii*
CotAD_74926	932	ARF5-3	Cotton_D_gene_10009104	Dt	*G. raimondii*
CotAD_09789	893	ARF6-2	Cotton_D_gene_10031674	Dt	*G. raimondii*
CotAD_49470	908	ARF6-4	Cotton_D_gene_10031573	Dt	*G. raimondii*
CotAD_65552	915	ARF6-6	Cotton_D_gene_10027306	Dt	*G. raimondii*
CotAD_48506	831	ARF6-7	Cotton_D_gene_10018893	Dt	*G. raimondii*
CotAD_06531	461	ARF7-1	Cotton_D_gene_10039023	Dt	*G. raimondii*

CotAD_01672	696	ARF10-1	Cotton_D_gene_10032280	Dt	*G. raimondii*
CotAD_14047	642	ARF11-1	Cotton_D_gene_10017685	Dt	*G. raimondii*
CotAD_27462	695	ARF11-4	Cotton_D_gene_10031002	Dt	*G. raimondii*
CotAD_44201	709	ARF16-1	Cotton_D_gene_10006397	Dt	*G. raimondii*
CotAD_60840	579	ARF17-1	Cotton_D_gene_10002224	Dt	*G. raimondii*
CotAD_42076	585	ARF17-4	Cotton_D_gene_10010678	Dt	*G. raimondii*
CotAD_35101	640	ARF17-5	Cotton_D_gene_10025306	Dt	*G. raimondii*
CotAD_16962	696	ARF18-2	Cotton_D_gene_10035704	Dt	*G. raimondii*
CotAD_76319	436	ARF18-3	Cotton_D_gene_10015890	Dt	*G. raimondii*
CotAD_29654	689	ARF18-5	Cotton_D_gene_10028503	Dt	*G. raimondii*
CotAD_08111	1103	ARF19-2	Cotton_D_gene_10016759	Dt	*G. raimondii*
CotAD_00347	856	ARF19-3	Cotton_D_gene_10037311	Dt	*G. raimondii*
CotAD_76501	686	ARF23-1	Cotton_D_gene_10019554	Dt	*G. raimondii*
CotAD_61329	675	ARF23-2	Cotton_D_gene_10008456	Dt	*G. raimondii*
CotAD_55285	668	ARF24-2	Cotton_D_gene_10035920	Dt	*G. raimondii*
CotAD_72164	684	ARF24-4	Cotton_D_gene_10005088	Dt	*G. raimondii*

Table S2. Location analysis of 12 *GhARF* genes out of the chromosome.

Gene ID	Gene name	Location
CotAD_64060	GhARF3-3	scaffold3586.1
CotAD_09605	GhARF10-2	scaffold288.1
CotAD_29901	GhARF19-1	scaffold586.1
CotAD_76410	GhARF2-1	scaffold7718.1
CotAD_00650	GhARF3-4	scaffold26.1
CotAD_49470	GhARF6-4	scaffold1889.1
CotAD_65552	GhARF6-6	scaffold2334.1
CotAD_35101	GhARF17-5	scaffold1451.1
CotAD_76319	GhARF18-3	scaffold12846.1
CotAD_08111	GhARF19-2	scaffold280.1
CotAD_76501	GhARF23-1	scaffold5427.1
CotAD_72164	GhARF24-4	scaffold7572.1

Table S3. Analysis of duplication events in *G. hirsutum ARF* genes located in chromosomes.

Gene ID	Gene name	Duplication type
CotAD_56239	ARF1-1	WGD/ segmental
CotAD_15593	ARF1-2	WGD/ segmental
CotAD_58764	ARF2-2	WGD/ segmental
CotAD_43230	ARF2-3	WGD/ segmental
CotAD_65781	ARF3-1	WGD/ segmental
CotAD_72124	ARF3-2	WGD/ segmental
CotAD_13694	ARF4-1	WGD/ segmental
CotAD_54309	ARF4-2	WGD/ segmental
CotAD_12422	ARF4-3	WGD/ segmental
CotAD_13433	ARF5-1	WGD/ segmental
CotAD_63258	ARF5-2	WGD/ segmental
CotAD_74926	ARF5-3	WGD/ segmental
CotAD_27033	ARF6-1	WGD/ segmental
CotAD_09789	ARF6-2	WGD/ segmental
CotAD_40344	ARF6-3	WGD/ segmental
CotAD_49470	ARF6-4	WGD/ segmental
CotAD_26127	ARF6-5	WGD/ segmental
CotAD_48506	ARF6-7	WGD/ segmental
CotAD_67703	ARF6-8	WGD/ segmental
CotAD_06531	ARF7-1	WGD/ segmental
CotAD_57703	ARF7-2	WGD/ segmental
CotAD_67896	ARF8-1	WGD/ segmental
CotAD_01672	ARF10-1	WGD/ segmental
CotAD_09605	ARF10-2	WGD/ segmental
CotAD_14047	ARF11-1	WGD/ segmental
CotAD_02558	ARF11-2	WGD/ segmental
CotAD_70430	ARF11-3	WGD/ segmental
CotAD_27462	ARF11-4	WGD/ segmental
CotAD_44201	ARF16-1	WGD/ segmental
CotAD_66018	ARF16-2	WGD/ segmental
CotAD_60840	ARF17-1	WGD/ segmental
CotAD_31393	ARF17-2	WGD/ segmental
CotAD_57862	ARF17-3	WGD/ segmental
CotAD_42076	ARF17-4	WGD/ segmental
CotAD_35101	ARF17-5	WGD/ segmental
CotAD_04601	ARF17-6	WGD/ segmental
CotAD_57415	ARF18-1	WGD/ segmental
CotAD_16962	ARF18-2	WGD/ segmental
CotAD_51593	ARF18-4	WGD/ segmental
CotAD_29654	ARF18-5	WGD/ segmental

CotAD_42645	ARF18-6	WGD/ segmental
CotAD_29901	ARF19-1	WGD/ segmental
CotAD_08111	ARF19-2	WGD/ segmental
CotAD_00347	ARF19-3	WGD/ segmental
CotAD_08070	ARF23-3	WGD/ segmental
CotAD_26342	ARF24-1	WGD/ segmental
CotAD_55285	ARF24-2	WGD/ segmental
CotAD_22697	ARF24-3	WGD/ segmental
CotAD_72164	ARF24-4	WGD/ segmental
CotAD_76410	ARF2-1	singleton
CotAD_64060	ARF3-3	singleton
CotAD_00650	ARF3-4	singleton
CotAD_65552	ARF6-6	singleton
CotAD_76319	ARF18-3	singleton
CotAD_76501	ARF23-1	singleton
CotAD_61329	ARF23-2	singleton

WGD, whole gemome duplication

Table S4. Transcription factor genes upregulated in 0 DPA ovules and containing ARF binding site in their promoter.

Gene ID	Annotation
CotAD_10537	ethylene-responsive transcription factor ERF105
CotAD_22795	ethylene-responsive transcription factor ERF106
CotAD_26577	MYB-APL transcription factor
CotAD_26900	MYB2 transcription factor
CotAD_19591	MYB44 transcription factor
CotAD_56556	MYB44-like transcription factor
CotAD_65011	MYB77 transcription factor
CotAD_62517	bHLH53-like transcription factor
CotAD_25671	bHLH144-like transcription factor
CotAD_16771	WRKY70 transcription factor
CotAD_36462	MYC2-like transcription factor

Table S5. A list of primers used in this study.

Gene ID	Sense primer sequence	Antisense primer sequence
QRT-PCR analysis		
ARF2-1	CGCCTCCCAGATGGCAGAGGA	ATGGCACAAAATTCCTGCCAAGG
ARF2-2	GGGGACTGCTCTTGGAAGGTCT	TTTGAACCCAACGACCCTGG
ARF2-3	GCCTCATACACGAGAGATTCAA	AATTCCAGCCAGGGATCATCTCC
ARF18-1	CGTACCAAGGTACATATGCAAGG	ACAACCGTCCCTTCACACTCC
ARF18-2	GCAAGTTGCATCGGAGACATC	GATGCTGGAAATTTGCATCTTGT
ARF18-3	CCACTTCTTCTATGAGAACTCG	CTAAAGACGAGGCTTGAAATTTGC
ARF18-4	GTTTGGCCTATGAGACAGAATG	CATATCACCTTCATTGTCAGT
ARF18-5	GGAACAAACGAACACAACGGTTG	CTTGCAAAAATCCATCCATGG
ARF18-6	GCAGTTATCCCCACTGACAAG	TACCACTGGGGCGAAGCTCTCC
ARF6-1	GAGCCATCTCTTATTTGGTGT	CTCTGGAGACTGCAAGAATCC
ARF6-2	TGCAGGCACTGATTTTTCAG	CAACAAATACAAGCTGCCAGC
ARF6-3	TCGGTTAATCCAGCAATGACAC	TTGATACACCAGACACTGTTCACG
ARF6-4	CTCAGGAAAATGTGGGCCAATC	AGAGTTTAGAAGCTCCAGGCC
ARF6-5	GTCGGGGTCCTTTGGGCGCTC	TGCCATTGCTGCTGGTGGTGG
ARF6-6	AATGATTCAATGTCCCTTCCG	GAATAGAAGTAGCAGGATTCTG
ARF11-1	CTGTTTCCTGAAGCAAGAGAAG	AATATTCTTCTTACCATGTTGC
ARF11-2	CTGTTTCCTGAAGCAAGAGAAG	GTCCCTTCACCTTCGATAGAAGC
ARF11-3	TTCAGGATTGTCAAACCCAC	GGTCATCGCCTACAAGCATC
ARF11-4	CAGGATTGTCAAACCCACAAGTG	CCACTTATTCCTAGGGCGAAGC
ERF105	TGCTTTTAAGCTACGTGGAAG	CCCTTCATATCTTCACTATC
ERF106	GGCTCGGAACTTTCGAAACCG	CTCCCGTCGTCGTTACGTTT
MYB-APL	GAATGATGGTTACCAGGTTAC	TTTGTTCTCTAAAGCTGCAGGAA
MYB2	CAGGTGGTCTTTGATTGCTG	TCTTCCATCTGGACAGTTTCC
MYB44	AATCCTGTCGGCTCCGATGGTGC	TCCCCAACCGGCAAGCACTTACG
MYB44-like	GCCCATCCCGAGGACCGGCGGA	TCTCCTCTCGAAACGCCACCG
MYB77	ATCCCGAGGACCGGCGGAGTC	TCTCCTCCCGAAACGCCGCC
bHLH53	GAGTTCATATTTCGACTGGC	TCGTGAAAGTTTCATATGGG
bHLH144	CAGAGGTTTGTCCCAAAAATTTCG	GGATTTCAGAGTAGAAGAAC
WRKY70	GGACAAAAGCAGATCCTTAATTC	ATTTCAGTGCTTGTATACACG
MYC2	GGGAGAAAACCGGCTAACGG	TCTAATTGGCTCTGCATCTCC
UBQ7	GGCATTCCACCTGACCAACAA	CCGCATTAGGGCACTCTTTTC
Vector construction		
ARF2-1	CCGGAGCTCATGACTACGTCGGA GATATCG	CGCGGATCCTTACCCTGAACAATT CTTTGC
ARF18-1	CCGGAGCTCATGGCTCATTTAGAA GGTA	CGCGGATCCTCAAACAGACCTATG CTC

Table S6. *ARF* gene sequences from the three cottons used in this study.

>GhARF1-1
ATGGCTTTTCCGGCATCAGAAATTCCATCTGCTGAACAACAAGCAGATGATCCTTTATATC
GTGAACTATGGCATGCCTGTGCTGGACCTCTTGTCACACTTCCTCGTGTTGGGGAGCGT
GTTTATTACTTCCCACAAGGTCACATGGAACAACTTGAGGCATCAATGCATCAAGGGTTAG
AACACCAAATGCCTTCATTCGATCTGCCATCTAAAATACTTTGCAAAGTGGCTTCTGTTCA
GCGTAAGGCTGAACCTGATACAGATGAAGTTTATGCCCAAATAACCCTGGTACCTGAAGTA
GATCAAAGTGAGGTTATGAGCCCAGATGATCCACTTCAAGAACCTGAAAGGTGCATAGTC
CATTCATTTTGCAAGACTCTTACTGCTTCCGACACAAGCACCCATGGTGGATTCTCAGTTT
TGCGCCGGCATGCAGATGATTGTCTGCCCCCGCTGGACATGACACAGCAGCCACCATGG
CAGGAACTGATTGCAACTGATCTGCATGGAAATGAATGGCATTTTCGGCATATTTTTCGAG
GACAACCTAGGCGCCACTTGCTCACTACTGGGGGTGCTAATGGGGAGCTGCGTGTTGGA
GTGAGGAGGCTTATGAGACAACAGGCAAATATGCCTTCTTCTGTTATATCTAGTCATAGCAT
GCATCTTGGGGTGCTTGCCACTGCATCTCATGCCCTTTCTACGCGAAGTATGTTTTCCATC
TTCTACAAGCCCAGAACAAGTTTGTCTGAGTTCATAGTGAGTGTAAACAAATTTCTTGAAG
CTCAAAGCCATAAGCTATCAGTTGGGATGAGGTTCAAAATGAGATTTGAGGGTGAAGAGA
TACCTGAAAGAAGATTCAGTGGCACAATTGTTGGTGTTGAGGATAATAAATCATCTGCATG
GGCTGATTCTGAGTGGAGATCTCTCAAGGTTCAATGGGATGAACCTTCATCCATCATACGT
CCTGATAGGGTGTCCCCATGGGAATTAGAGCCTCTTGTTGCAACTAGTAACTCTTCCATCT
CACAACCTGCACAAAGGAACAAGCGGGCTAGGCCACCCGTTCTACCTTCACCATCTTCA
GATCTTTCTTCACTTGGTATATGGAAATCACCAGTTGAATCTCCTTTCTCGTATTGTGATGC
ACAACGTGGGCACCCATCACCTAAATTATCCTCCGCTGCAAAGCCTAATTCTGTTGGCTTT
AATAGGAATAGCTCCCTGGCTGCAGTTTCTAGCAGCTCAATGTATTGGCCTAACCGAGTTG
AGAATGTTACAGAATCTGTTGCACCAGTTGTGAACAAAGAATCTAGTGAAAGAAAGCAGG
GAACTGGGAATGGCTGCAGACTTTTTGGTATTCAGTTACTTGACAAAATAAACATGGAAGA
AAATTCACCTTTGGCTACAATTTCTGGGACTGGTGTGGATGACCAGCCACTTCATTCACTA
GATGCTAATTCTGACCAGCAATCTGATCCATCAAATCTTAATCAGTCTGATCTTCCTTCTATA
AGTTGTGAACCTGAGAAGTGCCTGAGATCTCCTCAGGAGTCACAGAGCAAGCAAATTCG
GAGCTGCACAAAGGTTCACATGCAAGGTATGGCAGTTGGAAGGGCTGTTGATTTGACAC
GATTCGACTGTTATGAGGATTTGCTAAAGAAGCTGGAATACATGTTTGACATTAAAGGTCA
GCTTTGTGGATCAACAAAGAATTGGCAAGTTGTCTATACCGATGATGAAGATGACATGATG
ATGGTTGGGGATGATCCCTGGAATGAGTTTTGCAGCATGGTGAGGAAAATTTTTATCTATA
CATCAGAGGAAGTCCGGAAGTTATCACCTAAGATAAAACTTCCGGTGAATGATGATGATGA
TGATAGCAAAGCAACCAAGGCTGGGGTTGACACAGTTATCAACCCTGAAGATCGTTCATC
AGGAACATAG

>GhARF1-2
ATGGCTTTTCCGGCATCAGATATTCCATCTGCTGAACAACAAGCAGATGATCCTTTATATCG
TGAACTATGGCATGCCTGTGCTGGACCTCTTGTCACACTTCCTCGTGTTGGGGAGCGTG
TTTATTACTTCCCACAAGGTCACATGGAACAACTTGAGGCGTCAATGCATCAAGGGTTAGA
ACACCAAATGCCTTCATTCGATCTGCCATCTAAAATACTTTGCAAAGTGGCTTCTGTTCAG
CGTAAGGCTGAACCTGATACAGATGAAGTTTATGCCCAAATAACTCTGGTACCTGAAGTAG
ATCAAAGTGAGGTTATGAGCCCAGATGATCCACTTCAAGAACCTGAAAGGTGCATAGTCC
ATTCATTTTGCAAGACTCTTACTGCTTCCGACACAAGCACCCATGGTGGATTCTCAGTTTT
GCGCCGGCATGCAGATGATTGTCTGCCCCCGCTGGACATGACACAGCAGCCACCATGGC
AGGAACTGATTGCAACTGATCTGCATGGAAATGAGTGGCATTTTCGGCATATTTTTCGAGG
ACAACCTAGGCGCCACTTGCTCACTACTGGGGGTGCTAATGGGGATCTGCGTGTTGGAG
TGAGGAGGCTTATGAGACAACAGGCAAATATGCCTTCTTCTGTTATATCTAGTCATAGCATG
CATCTTGGGGTGCTTGCCACTGCATCTCATGCCCTCTCTACGCGAAGTATGTTTTCCATCT
TCTACAAGCCCAGAACAAGTTTGTCTGAGTTCATAGTGAGTGTAAACAAATATCTTGAAGC
TCAAAGCCATAAGCTATCAGTTGGGATGAGGTTCAAAATGAGATTTGAGGGTGAAGAGGT
ACCTGAAAGAAGATTCAGTGGCACAATTGTTGGTGTTGAGGCTGATAAATCATCTGCATG
GGCTGATTCTGAGTGGAGATCTCTCAAGGTTCAATGGGATGAACCTTCATCCATCATACGT
CCTGATAGGGTGTCCCCATGGGAATTAGAGCCTCTTGTTGCAACTAGTAACTCTTCCATCT
CACAACCTGCACAAAGGAACAAGCGGGCTAGGCCACCCGTTCTACCTTCACCATCTTCA
GATCTTTCTTCACTTGGTATATGGAAATCACCAGTTGAATCTCCTTTCTCGTATTGTGATGC
ACAACGTGGGCAACCGTCACCTAAATTATCCTCCACTGCAAAGCCTAATTCTGTTGGCTTT
AGTGGGAATAGCTCCCTGGCTGCAGTTTCTAGCAGCTCTAAGTATTGGCCTAACCGAGTT
GAGAATGTTACAGAATCTGTTGCACCAGTTGTGAACAAAGAATCTAGTGAAAGAAAGCAG

GGAACTGGGAATGGCTGCAGACTTTTTGGTATTCAGTTACTTGACAATATAAACATGGAAG
AAAATTCACCTTTGGCTACAATTTCTGGGACTGGTGTTAATGACCAGCCACTTCATTCACT
AGATGCTAACTCTGACCAGCAGTCTGATCCATCAAATCTTAATCAGTCTGATCTTCCTTCTA
TAAGTTGTGAACCTGAGAAGTGCCTGAGATCTCCTCAGGAGTCACAGAGCAAGCAAATTC
GGAGCTGCACAAAGGTTCACATGCAAGGTATGGCAGTTGGAAGGGCTGTTGATTTGACA
CGATTTGACTGTTATGAGGATTTGCTAAAGAAGCTGGAATACATGTTTGACATTAAAGGTC
AGCTTTGTGGATCAACAAAGAATTGGCAGGTTGTCTACACCGATGATGAAGATGACATGAT
GATGGTTGGGGATGATCCCTGGAATGAGTTTTGCAGCATGGTGAGGAAAATTTTTATCTAT
ACGTCAGAGGAAGTCCGGAAGTTATCACCTAAGATAAAACTTCCAGTGAATGATGATGATG
ATGATGATAGCAAAGCAACCAAGGCTGGGGTTGACACAGTTATCAACCCTGAAGATCGTT
CATCAATCGTTGGTCAAGGGTGCTAA
>GhARF2-1
ATGACTACGTCGGAGATATCGATAAAAGGAAATTGTGTCAACGGAAGAGGAGATAGTTTTT
CTTCCGGTTATACCGAGCCACGAGATACTAGGAACGCCATGGAAGGGCAGAACGGTCATT
CCGCTCGTACAGCTGCCGTCAGAGAAACCGTAGACCCCGAAAGGGCGCTGTATACGGAG
CTATGGCATGCATGTGCTGGACCTCTGGTGACGGTCCCTCGCGAATTAGAGCGCGTGTT
CTACTTTCCTCAAGGTCACATAGAACAGGTTGAGGCGTCTACTCATCAGGTATCAGACCA
GCAGATGCCGGTGTATGACCTTCCACCAAAGATCCTTTGTCGTGTGATTAACGTACAACTA
AAGGCTGAACTGGATACTGATGAGGTTTTTGCTCAAGTGACTTTGCTTCCTGAACATAATC
AAGATGAGAACATGGTGGACAAGGAGCCTCCCATTCTTGAACCCCCTCGGTTCCAAGTG
CATTCGTTTTGCAAAACCCTGACTGCTTCAGATACGAGTACCCATGGTGGATTTTCAGTGC
TCAGGCGGCATGCCGATGAATGTCTTCCACCACTGGATATGTCGCTGCAACCTCCAACAC
AGGAGCTGGTTTCTAAGGATTTGCATGGAAATGAGTGGCGATTCCGGCATATCTTCAGGG
GTCAGCCACGAAGACACTTGCTTCAAAGCGGTTGGAGTGTTTTTGTTAGCTCCAAGAAG
CTTGTTGCTGGGGATGCATTTATATTTTTAAGAGGCGAGAATGGAGAATTATGCGTTGGTG
TACGGCGAGCATTGAGACAACAGGGCAATGTTCCTTCATCGGTTATATCAAGTCATAGCAT
GCATCTTGGTGTGCTAGCGACAGCATGGCATGCCTACACTACCAGAACCATATTCACTGT
GTATTACAAACCCAGGACAAGTCCAGCTGAGTTCATTGTTCCATTTAATCAGTACATGGAG
TCGGTAAAGAACAATTACTCAATAGGGATGAGGTTCAAAATGAGATTTGAAGGTGAAGAAG
CTCCTGAACAGAGGTTTACTGGAACAATAGTTGGAATCGAAGATGCTGATCCAAAAAGGT
GGCAGGGTTCCAAATGGAGATGCCTGAAGGTGCGATGGGATGAAACGTCTACAATACCT
CGTCCAGAGAGAGTTTCTCCTTGGAAAATTGAACATGCTTTGTCTCCTCCTGCCCTTAATC
CCCTTCCAATGCCCCGGCCAAAAAGGCCTCGAACTAATGCTGTATCTTCATCCCCTGATT
CCTCTGTACTTAGTAGGGAAGGTTCTTCCAAAGTTACTGTAGACCCTTTGCCGGCCAGTT
CATTTTCAAGGGTCTTGCAAGGTCAAGAATTCTCGACCTTGAGAGGCACATTTGCTGAGA
GTAATGATTCTGAAACTGCTGATAGGTCAGTGATGTGGCCACCTTCAATAGATGATGAGAA
GATTGATGTAGCTCATGGTGAAAGAAAATTTGGGTCAGAGAATTGGATGCCCTCTAGGAG
GCATGAACCAACTTACACAGATTTGCTCTCAGGTTTTGGGTCGAATGCTGATACATCGCG
CGGATATTATCCTTCCTTTGTTGATCAAACTTCAGTAGCTGGTAATTCGGGGAAAAAACAAT
TACTAGGTCAAGAAGGGAAGCTTGGCTCTTGGTCCCTCCTGCCATCTGGTCTCTCACTCA
AGTTGTCTGACAGTAGTACAGACCCTCCTTTGCAAGGTTCTGATGTGCCTTGTCAGGCGC
GGGGAAATGGTAGATTTAGTGGTTTTGGTGACTACCCTATACTTGAAGGTCGTAGGATTGA
ATGCTCACGTGGTAATTGGTTGATGCCTCCCCCAACCACTTCTTGTTATGATAATTCAATCC
AGTCAAGAGATTTAATGCCGAAAACATCATTGGCTCAAGAGCATAAGAATGGAAAATCTAG
AGAAGGAAACTGCAAGCTCTTTGGTATTCCTCTCATAAGTGCTTCTAGCGCATCAGAGCC
TGCAGTCTCTCATATTAGTGCTTTCGCCAAGCCTGTAGGACATATGCAAGCTGCATTGCAC
CAGGTTCATGCACTTGAATCTGATAAAAGGTCTGAAAATTCAAACGCCTCCCAGATGGCA
GAGGATGTTTCTGCTTTTAATGAGCAGGAGAAAATAGTGAAGCTGGGTCAGCCCCATGCA
CGGGAGTTTCAAAGCAAACTGTCTACTGCTTCAACTAGGAGTTGTACTAAGGTTCTCATG
CAGGGGACTGCTCTTGGAAGGTCTGTGGACCTTACCAAGTTCAACAACTATGATGAGTTG
ATCGCTGAATTGGATCAATTATTTGAGTTTGGAGGTGAATTAATGGCCCCTCAAAAGAACT
GGCTTGTTGTTTATACTGATGATGAGGGTGATATGATGCTTGTTGGCGATGATCCTTGGCA
GGAATTTTGTGCCATGGTCCGCAAGATTGGTATCTACACTAGGGAAGAGGTCCAGAAGAT
GAAGCCAGGGTCGTTGGGTTCAAAGTTTGAGGACATTCCAGTTCCCACAGAAGGTACAG
TTGCAAAAGAAGTGAACTGTCCATCAGCATCTAGTGCAAAGAATTGTTCAGGGTAA

>GhARF2-2
ATGACTACGTCGGAGATATCGATAAAAGGAAATTGTGTCAACGGAAGAGGAGATAGTTTTT
CTTCCGGTTATACTGAGCCACGAGATACTAGGAACACCATGGAAGGGCAGAACGGTCATT
CCGCTCGTACAGCTGCCGTCAGAGAAACCGTAGACCCCGAAAGGGCGCTGTATACGGAG

CTATGGCATGCATGTGCTGGACCTCTGGTGACGGTCCCTCGCGAAGGAGAGCGCGTGTT
CTACTTTCCTCAAGGTCACATAGAACAGGTTGAGGCGTCTACTCATCAGGTATCAGAACA
GCAGATGCCGGTGTATGACCTTCCACCAAAGATCCTTTGTCGTGTGATTAACGTACAATTA
AAGGCTGAACCGGATACTGATGAGGTTTTTGCTCAAGTGACTTTGCTTCCTGAACATACTC
AAGATGAGAACATGGTGGACAAGGAGCCTCCCATTCTTGAACCCCCACGGTTCCAAGTG
CATTCGTTTTGCAAAACCCTGACTGCTTCAGATACGAGTACCCATGGTGGATTTTCAGTGC
TCAGGCGGCATGCTGATGAATGTCTTCCACCACTGGATATGTCGCTGCAACCTCCAACAC
AGGAGCTGGTTTCTAAGGATTTGCATGGAAATGAGTGGCGATTCCGGCATATCTTCAGGG
GTCAGCCACGAAGACACTTGCTTCAAAGCGGTTGGAGTGTTTTTGTTAGCTCCAAGAAG
CTTGTTGCTGGGGATGCATTTATATTTTTAAGAGGCGAGAATGGAGAATTGCGCGTTGGTG
TACGGCGAGCATTGAGACAGCAGGGCAACGTTCCTTCATCGGTTATATCAAGTCATAGCA
TGCATCTTGGTGTGCTAGCGACAGCATGGCATGCCTACACTACCAGAACCATATTCACTGT
GTATTACAAACCCAGGACAAGTCCAGCTGAGTTCATTGTTCCATTTAATCAGTACATGGAG
TCTGTAAAGAACAATTACTCAATAGGGATGAGGTTCAAAATGAGATTTGAAGGTGAAGAAG
CTCCTGAACAGAGGTTTACTGGAACAATAGTTGGAATCGAAGATGCTGATCCAAAGAGGT
GGCAGGGTTCCAAATGGAGATGCCTGAAGGTGCGATGGGATGAAACGTCTACAATACCT
CGTCCCGAGAGAGTTTCTCCTTGGAAAATTGAACATGCTTTGTCTCCTCCTGCACTTAATC
CCCTTCCAATGCCCCGGCCAAAAAGGCCTCGAACTAATGCTGTATCTTCATCCCCTGATT
CCTCTGTACTTACTAGGGAAGGTTCTTCCAAAGTTACTGTAGACCCTTTGCCGGCCAGTT
CATTTTCAAGGGTCTTGCCAGGTCAAGAATTCTCGACCTTGAGAGGCACATTTGCTGAGA
GTAATGATTCTGACACTGCTGATAGGTCAGTGATGTGGCCACCTTCAATAGATGATGAGAA
GGTTGATGTAGCTCATGGTGAAAGAAAATTTGGGTCAGAGAATTGGATGCCCTCTAGGAG
GCATGAACCAACTTACACAGATTTGCTCTCAGGTTTTGGGTCGAATGCTGATACATCACGT
GGATATTATCCGTCCTTTGTTGATCAAACTTCAGTAGCTGGTAATTCGGGGAAAAAACAATT
ACTAGATCAAGAAGGGAAGCTTGGCGCTTGGTCCCTCCTGCCATCTGGTCTCTCACTCAA
GTTGTCTGACAGTAGTACAGACCCTCCTTTGCAAGGTTCTGATGTGCCTTATCAGGCACG
GGGAAATGGTAGATTTTGTGGTTTTGGTGACTACCCTATACTTGAAGGTCGTAGGATTGAA
TGCACACGTGGTAATTGGTTGATGCCTCCCCCATCCACTTCTTGTTATGATAATTCAGTCC
ATTCAAGAGATTTAATGCCGAAAACATCATTGGTTCAAGAACATAAGAATGGAAAATCTAGA
GAAGGAAACTGCAAGCTCTTTGGTATTCCTCTCATAAGTATCTCTAGCGCTTCAGGGCCT
GCAGTCTCCCATATTAGTGCTTTCGCCAAGCCTGTAGGACATATGCAAGCTGCATTGCAC
CAAATTCATGCACTTAAATCTGATAAAAGGTCTGAAAATTCAAACGCCTCCCAGATGGCAG
AGGATGTTTCTGCTTTTAATGAGCAGGAGAAAATGGGTCAGCCCCATGCACGGGAGTTTC
AAAGCAAACCGTCTACTGCTTCAACTAGGAGTTGTACTAAGGTTCTTATGCAGGGGACTG
CTCTTGGAAGGTCTGTGGACCTTACTAAGTTCAACAACTATGATGAGTTGATCGCTGAATT
GGATCAATTATTTGAGTTTGGAGGTGAATTAATGGCCCCTCAAAAGAACTGGCTTGTTGTT
TATACTGATGATGAGGGTGATATGATGCTTGTTGGCGATGATCCTTGGCAGGAATTTTGTG
CCATGGTCCGCAAGATTGGTATCTACACTAGGGAAGAGGTCCAGAAGATGAAGCCAGGG
TCGTTGGGTTCAAAGTTTGAGGACATTCCAGTTCCCACAGAAGGTACAGTTGCAAAAGAA
GTGAACTGTCCATCAGCATCTAGTGCAAAGAATTGTTCAGGGTAA

>GhARF2-3
ATGAAAGGAAATTGTGTTAACGTAAGAGGAGGAGAAAGCTGTTCTTCTGGTTACAGTAAG
TCAATGGATGCTAGGAAGACCATGGAAGGGCAAAATGGTCATTCCACTCATCAAGCTGCC
ATCAGAGACCCTGAAACAGCGCTGTATACGGAGCTATGGCATGCATGTGCGGGACCGCT
GGTGACCGTCCCTCGCGAAGGAGAGAGCGTGTTCTACTTTCCTCAAGGTCACATAGAAC
AGGTTGAGGCGTCTACTAATCAGGTTCCAGACCAGCATATGCCAGTGTATAATCTTCCATC
CAAGATCCTTTGTCGGGTGATTAACGTTCAACTAAAGGCTGAACCAGATACCGATGAGGT
TTTTGCACAAGTGACTCTACTTCCTGAACCTACTCAGCAAGATGAGAATACCGTGGACAA
GGAGTCTCCCTTTCCTCAACCACCAAGGTTCCATGTTCATTCCTTTTGCAAGATCTTGACT
GCCTCAGATACAAGCACCCATGGTGGGTTTTCCGTTCTCAGGCGGCATGCGGATGAATG
TCTTCCACCATTGGACATGTCACGGCAACCTCCAACCCAGGAGTTGGTTGCTAAGGATTT
GCATGGAAATGAGTGGCGATTCCGACATATCTTCAGAGGTCAACCACGAAGGCACTTGCT
TCAAAGCGGCTGGAGTGTATTTGTTAGCTCCAAGAGGCTTGTTGCAGGGGATGCGTTTAT
TTTTTTAAGAGGTGAGAATGGAGAATTGCGTGTTGGTGTACGACGTGCAATGAGGCAGGA
GGGCAATGTTCCTTCATCAGTAATATCAAGTCACAGCATGCATCTTGGTGTGCTTGCAACA
GCATGGCATGCCTTCACAACAAAAACCATTTTCACGGTGTATTACAAACCTAGGACAAGTC
CGGCTGAGTTTATTGTTCCATTTGGTCAGTACATGGAGTCATTAAAGAATAATTACTCAATA
GGTATGAGGTTTAAAATGAGGTTTGAAGGGGAAGAAGCTCCTGAACAGAGGTTTACTGGA
ACAGTAGTCGGGATTGAGGATGCTGATCCCAAAAGGTGGCAGGATTCCAAGTGGAGATG

TCTCAAAGTGCGATGGGATGAGACGTCTTCCATACCTCGTCCGGAGAGAGTTTCTCCTTG
GAAAATTGAACCTCCTTTAGCTCCTCCTGCCTTAAATCCCCTACCAATGCCCAGGACCAAA
AGGCCTCGATCCCATGCAGTACCTTCATCCCCTGATTCCTCTGTGCTGACTAGGGAAGGT
TCATCCAAAGTTACTATAGACCCTTCGTCAGCTAGTGGGTTGTCAAGGGTTTTGCAAGGC
CAAGAATTCTCGACCTTGAGAAGCAACTTTGCTGAGAGTATTGAGTCGGACATTGCTGAA
AAGTCAGTGATGTGGCCTCCTTCTGTAGATGATAACAACAATGATGTGGTTTCTGCTTCAA
GAAGATTTGCATCAGAGAATTGGATGTCCTCTGGGAGGCATGAGCGACCATGCACAGATC
TGCTCACGGGGTTTGGGTCAAATGCTGAGTCCTTGCATGGATACTGTTCATCCTTGGTTG
ATCAAACTTTAGTGGCTGGTAATTCAACGAGAAAACAATCACTACATCAAGAAGGAAAGCT
TGGCTCTTGGTCCCTCATGCCCTCTGGTCAGTCACTTAAGTTGGCAGACACCAATGCAAA
GTCTCCACTGCAAGGTTCTGATGTGCCTTATCAAATGCGAGGAAATGGTAGATGTAGTGG
TTTTGATGACTATCCTATACTTCAAGGTCATAGGATTGAACACTCGCATGGAAACTGGTTGA
TGCCTCCCCCCACTTCATCTCATCATGAAAGCAATCCAGTCCAATCAAGAGATTTAATGCC
CAAAACATCATTTGTACAAGGGCATGAGAATGGAAAATATAGAGAAGGAAATTGTAAACTC
TTTGGTATTCCTCTCATTAGGAATTCTGTTGCATCAGAGCCGACAGTCTCTCATATGCAAG
CTGCATCCCGCCAAGTTCTTGAATTTGAGTCTGCTCAAAATTCTGAAAAGTTGAAGGCCTT
GCAATTGGCAGAGGTGGAGGACCTGTCTAATTATAACGAGCAGGAGAAACAGCCTCATAC
ACGAGAGATTCAAAGCAAACCTTCAAGTGCCTCAACTAGGAGTTGTACCAAGGTTCACAA
GCAGGGGATTGCTCTTGGTAGGTCGGTGGACCTTACTAAGTTCAATGACTATGATGAACT
GATTGCTGAGTTGGATGGATTATTTGAATTTAAAGGCGAGTTAATGGCCCCTAAGAAGAAT
TGGCTCGTTGTTTATACTGATGATGAGGGTGATATGATGCTTGTTGGAGATGATCCCTGGC
TGGAATTTTGTGCCATGGTTCGCAAGATTGGTATCTACACTAGGGAGGAGGTCCAGAAAA
TGAAGCCAGGATCATTGAGTTCAAAGGGTGAGGACAATTCAGTTTCCACAGGAGGCGTA
GATGGAAAGGAAATGATGTGTCCATCAGCATCTAGTGCAGAGAATTGTTAG

>GhARF3-1
ATGGGGGGATTAATCGATCTGAACTCTACGGAAGACGATGAAACTCCATTATCTGGTTCTT
TGTCTCCATCTTCATCTTCAGCTTCAGTGTTAAGTGCCCCTGGTTCTGGTTCTTCTGTTTG
TTTAGAACTTTGGCATGCGTGTGCTGGTCCCCTTATATCTTTGCCAAAGAGAGGAAATGTA
GTGGTGTACTTCCCTCAAGGCCACTTGGAACAAGTTTCCGATTTTTCCGGTGTAGCTGCA
GCTTATGATCTCCCTTCCCACGTGTTTTGTCGGGTTGTTGATGTCAAGCTCCATGCTGAG
GGTGCCACAGATGAGGTTTACGCCCAAGTTTCATTGGTTCCTGAAACTGAGCAATCTGAG
CAAAAGTTGGAGGGAGGGAAGACTGAGGCAGATGGGGAAGAGGAGGATGCTGAAACCA
ATGTCAAGTCAACCACGCCCCATATGTTCTGCAAGACACTAACCGCTTCCGATACCAGCA
CGCATGGTGGCTTCTCTGTTCCTCGTCGAGCTGCCGAGGACTGCTTTCCTCCCTTGGAC
TATAATCAGCAAAGGCCCTCACAAGAGCTTGTTGCAAAAGACCTGCATGGTTCTGAATGG
AGATTTCGACACATCTATAGGGGTCAACCACGGAGACATTTGCTGACTTCCGGATGGAGT
GCTTTTGTAAATAAGAAGAAGCTTGTCTCTGGAGATGCGGTGCTTTTTCTGAGGGGTGAA
GATGGAGAACTGAGGCTTGGAGTCCGAAGAGCTGCTCAAATTAAAGATGGCTCTTCTTTT
CCATCTTCTTGCAGCCAGCAGTTGAATTGCAGCAATTTTGCAGATGTGGTTCATGCTGTTT
CTATGAAAAGTGTATTCAGCATTTACTACAATCCAAGGGCCAGTTCATCGGACTTCGTAATA
CCCGTGCATAAATTCTGGAAGTGTCTTGATCCCTCATTTTCTATTGGAATGAGGTTCAAAAT
GCAATTTGAAGCTGAAGATGCAGCAGAAAGAAGGTACAACCTTATTGAATCTCCAACTCTT
GAGTTCAAATTATTTGATTTTCAGCCTAAAGAGGAGAGCAAAGACCTACTCTACCATGTGA
TGAAGGCCTTAATTGACTTTTCTTGCAGACACTCAGGAGTAATAACTGGAATTAGTGATATA
AATCCTGTTCGATGGCCTGGTTCAAAATGGAGATGTTTGATGGTAAGGTGGGATGATATTG
ATGCCAACAGGCATGGTAGGGTTTCTCCCTGGGAAATTGAGCCATCTGGTTCAGTTTCCG
GTTCTAACAGCTTGATCTCTCCTGGTTCAAAAAGGAACCGAGTTGGATTTCCTTCAGGAA
ATTCTGAATTTATGGTTCCTGATGGAATTAGAGCATCAGACTTTGGGGAGTCTTTGTGGTC
CCAGGTATTGCAAGTTCAAGAAAATCTGGGTTTTAACACTCTTTATGATGGTTCTGATAGTC
TGAATATGCATTGGTCTGAAATAAGGCGTTGCATTCCTGGTTCTATTGGTTCTGATTTTTCT
GCAATAGGAAATATTGGTAGAGGCTCACTGGTGAGTCCTGATATTTCCCGTAATAATGTAG
GCTTTGGGGAATCTTTCCGATTCCGTAAGGTCTTGCAAGGTCAAGAAATTTTTGTGTCCC
CTCCATATAGAAACGGTTCAACTGCAGATGAAAATGAAGAAAATGACGCTTTTGGTCTCCC
TGATGTTGGTCAGTTGTCGGGAACTAGAAGTGGATGGTCTTCCTTGATGCAGAGGTATAAT
ACTCATAGTCGTACACGACCATTTGCACCATCTACACAAACGTCCTCACCATCTTCAGTGT
TAACGTTCCTACAAGTGAGCAATCCAATTCTGAATTTCAGTCCTATCTATAATTCTAACAAC
CAAAAAAGGAAACAGGGAGTTAACAAACAGAGTTCTTTTCATGCACCTGAAATATATGAGG
GAAAGCTATTTCCATCTTCAGCCAGTGAATATGATTCCCGTGCGAGGGATCTTGGAAGCT
CAGATTTATTCGGTCATTCTATTTGTTCTGTTCAACCTGGTGATGCTTCACCTCTAGCAGCT

CAACCAGTATTCAGGACTAGTCAAGAATTAGATTCCTCCTGTAAAAGTAGCTGCAGACTTT
TCGGTTTCTCCTTGACTGAGGGAAGACATGATGCTAGCAAGGAAGAAGACGTGGTACAA
GCAACCTCATCATTGGCCCCTGGAGCGTTTTTACCTTGTGTTAGGGAAGAGTTTCACCCA
AAGCCTTCGTCGGTGACGGACACAGTTGGAAGCAATTATACTGAAGTAAGCAATCTCTAT
GCTGTCAGAGATATGGTTTTAGATATTGCGTTGTAG

>GhARF3-2
ATGGGGGGATTAATCGATCTGAACTCTACGGAAGACGATGAAACGCCATTATCTGGTTCTT
TGTCTCCATCTTCATCTTCAGCTTCCGTGTTAAGTGCCCCTGGTTCTGGTTCTTCTGTTTG
TTTAGAACTTTGGCATGCGTGTGCGGGTCCACTTATATCTTTGCCAAAGAGAGGAAATGTA
GTGGTGTACTTCCCTCAAGGCCACTTGGAACAAGTTTCCGATTTTTCCGGTGTAGCTGCA
GCTTATGATCTCCCTCCCCACGTGTTTTGTCTGGTTGTTGATGTCAAGCTCCATGCTGAG
GGTGCCACAGATGAGGTTTACGCCCAAGTTTCATTGGTTCCTGAAACGGAGCAATCTGAG
CAGAAGTTGGAGGGAGGGAAGACTGAGGCAGATGGTGAAGAGGAGGATACTGAAACCA
ATATCAAGTCAACCACGCCCCATATGTTCTGCAAGACACTAACCGCTTCCGATACCAGCAC
GCATGGTGGCTTCTCTGTTCCTCGTCTAGCTGCCGAGGACTGCTTTCCTCCCTTGGACTA
TAATCAGCAAAGGCCCTCACAAGAGCTTGTTGCGAAAGACCTGCATGGTTCTGAATGGAG
ATTTCGACACATCTATAGGGGTCAACCACGGAGACATTTGCTGACTTCCGGATGGAGTGC
ATTTGTAAATAAGAAGAAGCTTGTCTCTGGAGATGCGGTGCTTTTTCTGAGGGGTGAAGG
TGGAGAACTGAGGCTTGGAATCCGAAGAGCTGCTCAAATTAAAGATGGCTCTTCTTTTCC
ATCATCTTGCAGCCAGCAGTTGAATTGCAGCAATTTTGCAGATGTGGTTCATGCTATTTCTA
TGAAAAGTGTATTCAGCATTTACTACAATCCAAGGGCCAGTTCATCGGACTTCATAATACCC
GTGCATAAATTCTGGAAGTGTCTTGATCCCTCATTTTCTATTGGAATGAGGTTCAAAATGCA
ATTTGAAGCTGAAGATGCAGCAGAAAGAAGACACTCAGGAGTAATAACTGGAATTAGTGAT
ATAAATCCTGTTCAATGGCCTGGTTCAAAATGGAGATGCCTGATGGTAAGGTGGGATGATA
TTGATGCCAACAGGCATAGTAGGGTTTCTCCCTGGGAAATTGAGCCATCTGGTTCAGTTT
CCGGTTCTAACAGCTTGATCTCTCCTGGTTCAAAAAGGAACCGAGTTGGATTTCCTTCAG
GAAATTCTGAATTTATGGTTCCTGGAAATATTGGTAGAGACTCACTGGTGAGTCCTGATATT
TCCCGTAAAAGTGTAGGCTTTGGGGAATCTTTCCGATTCCATAAGGTCTTGCAAGGTCAA
GAAATTTTTGTGTCCCCTCCATATAGAAATGGTTCAACTGCAGATGAAACTCAAGAAAATG
ACGCTTTTGGTCTTGCTGATGTTGGTCAGCTGTCGGGAACTAGAAGTGGATGGTCTTCCT
TGATGCAGAGGTATAATACTCATAGTCGTACACGACCATCTGCACCATCTACACAAACGTC
CTCACCATCTTCAGTGTTAACGTTCCTACAAGTGAGCAATCCAATTCTGAATTTCAGTCCTA
TTTATAATTCTAATAACCAAAAAAGGGAACAGGGAGTTAACAAACAAAGTTCTTTTCATGCA
CCTGAAATATACAAGGGAAAGCTATTTCCATCTTCATCCAGTGAACATGATTCCCGTGCGA
GGGATCTTGGAAGCACAGATTTATTCGGTCATTCTATTGGTTCTGTTCAACTTGGTTTTGC
TCCACCTCTAGCAGCTCAACCAGTATTCAGGACTAGTCAAGAATTAGATTCCTCCTGTAAA
AGTAGCTGCAGACTTTTCGGTTTCTCCTTGACTGAGGGAAGACATGATGCTAGCAAGGAA
GAAGACGTGGTACAAGCAACCTCATCATTGGCGCCTGGAGCGATTTTACCTTGTGTTAGG
GAAGAGTTTCACCCAAAGCCTTCGTCGGTGACGGACACAGTTGGAAGCAATTATACTGAA
GTAAGCAATCTCTATGCTGTCAGAGATATGGTTTTAGATATTGCATTGTAG

>GhARF3-3
ATGGGGGGTTTAATCGATCTGAACACAACGGAAGACGAGGAAACGCCGTCATATGGTTCT
TTATCCCCTTCTTCATCTTCAGCTTCCGTGTTAAGTGCTTCTGGCTCTGCTTCAAGTTCTC
CCGTTTGTTTAGAGCTCTGGCATGCGTGTGCCGGTCCACTTATATCTTTGCCCAAGAGAG
GAAGTGTAGTGGTTTACTTCCCTCAGGGCCACTTGGAACAAGTGTCCGATTTTTCTGGCG
TAGCTCCAGCATATGATCTCCCTCCTCACGTGTTTTGTCGGGTTGTTGATGTCAAGCTCCA
TGCTGAGGGTGCCACAGATGAGGTTTACGCTCAAGTTTCACTTGTTCCTGAAAATGAGCA
AATTGAGCAGAAGTTGAAAGAAGGGAACATAGAAGTAGATGGTGAAGAGGATGCCGAAG
CAGATATCAAGTCCACCACGCCCCACATGTTCTGCAAGACTCTTACGGCTTCTGATACCA
GTACACATGGAGGCTTCTCTGTTCCTCGTCGAGCTGCTGAGGACTGCTTCCCTCCCTTG
GATTATAATCAGCAGCGGCCCTCCCAAGAGCTTGTTGCTAAAGACCTGCATGGCCTGGAA
TGGAGATTTCGACACATCTATAGGGGACAACCACGGAGGCATTTGCTTACAACTGGATGG
AGTGCTTTTGTAAATAAGAAGAAGCTTGTCTCTGGAGACGCTGTGCTTTTTCTTAGGGGT
GAGAATGGAGAACTGAGGCTGGGAATCCGAAGAGCTGCTCACATTAAAAATGGCACTTCT
TTTCATTCTTTGTGCACCCAGCAGTTGAACCGCAGCAATTTCGCAGATGTGGTCCATGCTA
TATCTATGAAAAGTGTGTTCAGCATTTACTACAATCCAAGGGCCAGTTCATCAGAGTTCATA
ATACCAGTGCATAAGTTCTGGAAGAGTCTTGATCATTCTTTTTCTGTTGGAATGAGATTTAA

GATGCGGTTTGAATCTGAAGATGCAGCAGAAGGAAGATACACAGGAGTTGTAACCGGAAT
TAGTGAGATGGATCCTGTTAGATGGTCTGGTTCAAAATGGAGATGCCTGCTGGTAAGGTG
GGATGATATTGAAACCAATAGGCATATTAGGGTCTCTCCCTGGGAAATTGAACCATCCAGT
TCAATATCTAGTTCTAACAGCTTGCTCTCTCCTGTTTCTAAAAGGAACAGGGTTGGACTGC
CTTCAGGGAAACCTGAATTTATGGTTCCTGAAGGAATTGGAGCATCAGACTTTGGGGAAT
CTTTGCGGTTCCAGAAGGTCTTGCAAGGTCAAGAAATTTTGGGTTTTAACACTCATAATGA
TGGTGCTAATGGTCAGATAATGCACCGGTCTGATATAAGTCGGCGCTTTCCTTGCTCTAAT
GGTTCTGGTATTGCTGCTATACGAAATATTGGTAGAGACACATTGGTGAATCCTGATATTTC
TTATAAGGGTGTAGGCTTTGAGGAATCTTTCAGATTCCAAAAGGTCTTGCAAGGTCAAGAA
ACTTTTGTAAGCCCTCCATGTAGAAGAGGTCCAACTGTAGATGACACTCGAGAAAGTGAC
AGTCCTGGTGCCCCTGATGTTGGTCAGTTGTCGGGAACTAGAAGTGGATGGTCTTCTTTG
ATGCAGAGCTATAATAATAGTCGTATAGGACCATCTGCACAAGTGTCCTCACCGTCTTCAG
TGCTAAAGTTCCAACATGCAAGCAATCCATTTGCAAACGTCAATCCTATTCATAACTTGAAT
AGCAAGGAAAAGGAGCAAAGAGTTCATAAAAGTAGTTCTTTTCATGCTCCCGAAACATATG
GGGACGGGATACCATCTTCAACTGGTGGACATGGTTCCCGTAGGAGGTATCTAGGAAGC
CTGCATTCATTTGGTCCTTCAACTGATACTGTTCAGCTTGGTGATTCTCAACCTCTGTCAG
CACAACCAACATTTAGGACAAGTCAAGAATTAGCTGCTTCATGTAAAAGTAGCTGCAGGCT
TTTTGGTTTCTCCTTGACTGAGGGAGGACATGATACTGCCAAGGAGGACAACATGGTGCA
AGCAACCTCGTCATTGGGTGCTGGAGCTTTCTTACCTCGCATTGGGGAACAGTTTAACAC
ACAACCTCCTGCAGTGACAAACACAGTTGGAAGCAGTTATACCAAAGTAAGCAACCTCTA
CGCTGTCAGAGATATTGTTTATGATATTGCATTATAG

>GhARF3-4
ATGGGGTGTTTAATCGATCTGAACACTACTGAAGAAGATGAAACGCCGTCGTCTTGTTCTT
TATCACCATCTTCTGTATTGAGTGCTTCTGGTTTTAGTTCTTCAGTTTGTTTAGAGCTTTTG
CATGCTTGTGTTGGTCCACTTATATCTTTACCAAAGAAAGGAAGTGTAGTGGTGTACTTCC
CTCAAGGCCACTTGGAACAAGCTGCCGAATTTTCCGGCTTAGCTTCAGTTTATGATCTTCC
TCCTCATGTGTTTTGTCGGGTTCTTGATGTTAAGCTCCATGCTGAGGGTGCTACAGATGA
GGTTTATGCTCAAGTTTCACTGGTTCCTGATAATGAGCAAACTGAGCAGAATTTGCAAGAG
GTAGATGGTGACGATGAGGATGCCGAAGCAGATATGAAGTTAGCCACGCCGCACATGTTC
TGTAAGACCCTTACCGCTTCCGACACCAGTCCTCATGGTGGTTTCTCCGTTCCACGTCGA
GCTGCCGAGGACTGCTTCGCTCCCTTGGATTATGATCAGCAGAGGCCATCACAAGAGCT
CGTTGCAAAAGATCTACACGGTGTAGAATGGAGATTTCGACACATTTATAGAGGGCAACCA
CGGAGGCATTTGCTCACTACTGGGTGGAGTGCTTTCGTAAACAAGAAGAAACTCGTATCC
GGAGATGCTGTGCTTTTTCTTAGAGGCGAGGATGGAGAACTGAGGCTGGGAATCCGAAG
AGCTGCCCAAATTAAAAACGGGGCTTCTTTTCTTTCTTTGTGCAGCAAGCAGTTGAACCG
CAGAACTTTTGCAGATGCGGTCCATGCTATATCTATGAAAAGTGTTTTCAGCATTTACTACA
ATCCAAGGGCTAGTTCATCGGAGTTCATAGTTCCGGTCTGTAAATTCAGGAAGAGCCTTG
ATCGTTCATTTTCTGTTGGAATGAGGTTCAAAATGCGGTTTGAAACTGAAAATGCACCAGA
ACGAAGATCCTCGGGACTCATAACTGGAACTAGTGATTTGGATCCTGTTAGATGGCCTGG
GTCAAAATGGAAATGCTTGTCGGTGAGGTGGGATGATATTGACGCCAACAAGCACGGGA
GGGTTTCTCCCTGGGAAATTGAACTTTCCGGTTCTATTTCTAGTTCTAACTGCTTGCTCTC
ACCCTGTTCGAAAAGGAACAGGGTTGGATTGCCTTCAGGAAAACCGGAATTTATGGTTCC
TGATGGAATTGGAGCACCCGACTTTGGGGAACCTTTGCGGTTCCAAAAGGTCTTGCAAG
GTCAAGAAATATTGGGTTTTAGCACTCTTTATAATAGTGCTGATAGTCACAACATGCATTGG
TCTGAAATACGACGGTGCTTTCCTGGATCTAATGGTTCTGGCATTGCTACAATTGGAAATG
TTGGTAGAGACCCTCCGTTGAATCCCGTTATTTCATATAAAGGTGTAGGCTTTGGGGAATC
TTTTGGATTCCATAAGGTCTTGCAAGGTCAAGAAAATTTTCTAAGCTCTCTGTATAGAAAAG
GCTCAACTATGGAAGAGACTCGAGGAAATGACAGTGCTGGTCTCACTAATGTTGGGCAAA
TGTCGGGAACTAGAAGTGGATGGTCTTCCTCGATGCAGGGATATAATACACATAGTCCTGT
ACAACCATCTGTGCAAGTTTCCTCACCGTCTTCTGTGTTGATGTTCCAACAAGCAAGCAAT
CCAGTTCCGAACTTCAATCCTGCTCGTAACTTCAACCAGGAAATGGAGCGGGGAGTGAG
TTCTTTTCGTGTGCCCGAAACTTATGGAGCCAAGTTACTGTCATCGTCAATCAGCGAACAT
GATTCACAACCTCTGGCAGCTCAACCTTCTTTTGGGACCAATCAAGAATTAGCCTCCTGC
AAAAATAGCTGCAGGCTTTTTGGTTTCTCCTTAACTGAAGGAGATCTTGATGCAACTAAGG
AAGACAGCGTGGTACATGCAACATTATCATTGGGTCGTGGATCGCTTTTACCTTGTATCGG
AGAAAATTTCCATCCAAATCCTCCAGCAGTGACAAGCACAGTTTGGAAGCAATTGTACTAA

>GhARF4-1
ATGGAAATTGATCTGAACCATGCACTGAGTGAGGTGGAGAAGACTGCGGTTTGCAATGG

GGACTGTGACAAACTGAACTGTGTGTGCTCTTCATCTTCTTCAAACTTAGCTTCGCCTCCA
TGTACCTCTTCCATTTACTTGGAGCTTTGGCATGCTTGTGCTGGCCCTCTCACTTCACTCC
CGAAAAAGGGAAATCTTGTTGTTTACTTCCCTCAGGGTCACTTGGAGCAGCTGGCCTCTG
CCTCTCCTTTCTCTCCTTTGGAAATCTCCACCTTTGATCTTCCCCCTCACATCTTTTGCAA
AGTGGTGAATGTCCAGCTTCTTGCCAATAAGGAGAATGATGAGGTCTATACACAGCTCACT
TTACTTCCTCAGCCGGAGTTGAGAGGGCCTAATTTGGAGAGCAAGCAGCTGGATGAACTA
GGTGTGGATGAGGGAGATGATGGATCGCCTAAAAGATCAACCCCTCACATGTTTTGTAAG
ACGCTAACCGCATCGGACACCAGCACTCACGGAGGGTTCTCCGTTCCTCGTAGAGCTGC
AGAAGACTGTTTTCCTCCATTGGATTATAAACAGACGAGGCCGTCTCAAGAACTTGTTGC
CAAGGACCTGCACGGAGTTGAGTGGAGGTTTCGACATATATATAGGGGTCAACCGAGGC
GACATCTCCTTACTACAGGCTGGAGTATTTTTGTTAGTCAAAAGAATCTTGTTGCTGGTGA
TGCAGTGCTCTTTCTGAGGCATGAACTTCTGACATTATTTGGAGTTCTCGCTGATTTATCTT
TTCTTCAACACTATAGGGGTGAGGATGGAGAGCTCAGGTTGGGAATTAGAAGAGCTGTAC
GACCGAGGAATTGTCTTCCTGAATCGGTTATTGCTAAACAGAATTCATATCTGAATGTTCTT
TCTCCGGTGGCTAATGCATTATCCATGAAAAGCATGTTTCATGTCTTCTACAGTCCAAGGG
CCAGCCATGCAGAGTTTGTCATACCCTTTCGGAAGTATTTTAAAAGCATAGCAAATTCAGT
GTGCATTGGAACAAGATTCAAAATGAGATTTGACATGGATGATTCGCCCGAGAGAAGGTTT
AGTGGTGTAGTGATGGGAATGGGGGACTCGGATCCTTACAAATGGCCCAATTCAAGGTG
GAGATGCTTAATGGTCAGGTGGGATGAAGATAATATGATTGATCGCCACGAGAGAGTCTC
ACCTTGGGAAATTGATCCTTCTGCTTCTCTCCCACCCTTTAGTATTCAATCTTCCCCGAGG
TTGAAGAAACTACGAACAGGTCCGCAGGCTGCCACACCCGACACCCTCATTGCGGGGG
GGAGTCGATTTTTGGACTTTGAGGAACCGTTGAGATCCTCTAAGGTCTTGCAAGGTCAAG
AAAATGTAGGTTTTGTATCACCCTTATATGGGCGTGATACTGTAAGCCGCCCGCTAGATTTT
GAGATGCAATCTCCTGCACATCATCAAAGTATTGCGTCAACAGGGATTGAAAAGAGTAATA
TTAGCGAGTTTATGAGAGTTCGCTCCACCACATACACAGGCTTTGCGGATTCTAATAGGTT
TCCGAAGGTCTTGCAAGGTCAAGAAATTTGCCAATTGAGATCCCTGACACAAAAGGCTGA
TCTCAATCTCGGTGTTTGGGCGAAAACCAATCTTGGTTGCAATTCATTCAACATGCATCAA
ACACTGAGAACCAATTGCTATCCGCTAGCATCGGAAGGCCTTCGAAATATGTATTTCCCTT
ACAGTGAATTTCTCAAAACCGTCCAAGAGCCGACAATGAGCTCTTATGCATGTCCACTTC
CAAGAGGTAATGTCCCGTTTAATGCTTCCTCAATTAGGACAGGGGTAGGGGTTATCGTGG
ATGGGTTCAGAAAACCGAATCAACAGAATGAGCATAAGCCATTGGAGAATATTCCAAGTCC
TGCTTCGGAAAACAATTTGAGGAACCAACAGGATGACTCCTTCAAAAGAAATGTTGCTGG
ATGTAAACTCTTTGGGTTTCCTTTGAATGTGGAGTCGCCTACTCCAAACTCACAAAATTCT
GGTAAGAGGAGTTGTACAAAGGTTCACAAGCAAGGCAGCTTGGTTGGAAGAGCTATTGAT
CTCTCGAGACTGTACGGATATGACGACTTGATGATTGAACTTGAACGTCTATTCGGAATGG
AAGGCGTATTAAGTGATCCGGATAAAGGGTGGCGGGTATTGTACACAGATAGCGAGAACG
ACGTAATGGTGGTTGGAGATGACCCTTGGCATGAATTTTGTGAGGTGGTGTCGAAGATCC
ACGTATACACCCAAGAAGAAGTGGAGAAGATGACAATAGGAACGGGGAGCGATGACACG
CAAAGCTGTTTGGAGCAAGCAGCAGTGATAATGGAAGTATCAAAGTCATCCTCAGTGGGG
CAGCCAGATTCATCATCTCCAATGTTTTGGTGTTTTGCAAAGGCACCTAAGTTTGATACAT
GCATATTGAGGAATGGTGTCATTTCAAAGGCAGTTGAAATGACTCACGTTAATGTCAAGTT
TATAGGTGCTTTGAATCTTGAGAACCCATTCCAAACATGGGTTCCCAAAGCTTGGGGAATT
TTTATTGGCTAA

>GhARF4-2
ATGGAAATTGATCTGAACCATGCAGTAAACGAGGTGGAGAAGACTGTACTTTGCAATGGG
AGCTGTGAAAAAGCTAGTGCTTGCGTTTACTGTTTGTCGTCGTCGTTGTCGTCATCATCAT
CTTCTTCTTATTCTTCTTCTTCATGTTCTTCAAACTCAGGTTCACCTCCTTGTTCATCTTCCA
TTGACTTGGAGCTTTGGCATGCTTGTGCTGGTCCTCTCACATCACTTCCAAAGAAGGGAA
ATGTGGTTGTATACTTCCCTCAAGGTCACTTGGAACAACTTGCCCTTGCTTTTCCTTTCTC
TCCCTTGGACACCTTTGATCTTCCTCCCCAAATCTTTTGCAAAGTGATGAATGTCCAGCTT
CTGGCCAATAAGGAGAATGATGAGGTCTATACGCAAGTCACTTTACTTCCTCAACCGGAG
TTGGGAGGGCATAATTTGGGGAGCAAGCAGCTGGATGAGGTAGGGGCGGATGAGCATG
CCGATGGGTCGCCTTCGAAGCCAACCTCTCACATGTTTTGCAAGACACTAACAGCTTCTG
ACACTAGCATCCATGGAGGGTTCTCTGCTCCTCGTAGAGCTGCTGAAGACTGTTTTCCTC
GGCTGGATTATAAACAGACAAGGCCCTCACAGGAGCTTGTTGCTAAAGACCTCCATGGAG
TTGAGTGGAGGTTTCGCCATATATATAGGGGTCAACCGAGGCGCCATCTCCTTACTACCG
GCTGGAGTGTTTTTGTGAGTCAAAAGAATCTTATTGCTGGGGATGCAGTGCTCTTTCTGA
GGGGCGAAGATCGGGAGCTGAGGTTGGGAATTAGAAGAGCTGTTCGACCAAGAACTGG
TCTTCCAGATTCAATTATTGCGAAACATAATTCATATCCAAAGGTTCTTTCTGCAGTGGCTA

ATGCATTATCCACCAAAAGAGTGTTTCAAGTCTGCTACTGTCCAAGGGCCAGTCATGCCG
AGTTCGTCATACCCTTCCAAAAGTATATTAAAAGCATCACAAATCCAATGTGCACTGGGGC
AAGATTCAGAATGAAATTTGAAATGGATGATTTGCCGGAGAGAAGGTCTAATGGTGTAGTG
ACAGGAATAGGGGACTCAGATCCTTATAAATGGCCTAACTCAAAGTGGAGATGCGTAATG
GTTAGGTGGGATGATGATATCGCAAGTGATCACCAAGAGAGAGTTTCACCTTGGGAAATT
GATCCTCCTGTTTTTCCCCCACCCTTAAGTATTCCATCTCCATTCAGGTTAAAGAAACTGC
GGACAGGTCTGCAGACTGCAGCACTTGACACCCCTATCACTGGGGTTGGATATTTGGACT
TTGAGGAATCGACAAGATCCTCTAAGGTCTTGCAAGGTCAAGAAAATGTAGGTTTCATATC
ACCCGTATATGGATGTGGTACAGTGAACCATCCCCTCAATTCCGAGATGCAATCTATGGCA
CATCAAAGTCTTGCATCGACAGGAATTGGAAAGAGTAACATTAGTGATTTTATGAGGGCTC
ACTCCTCCTCTTACACAGGCTTTGCGGAAACTAATCGGTTTCCGAAGGTCTTGCAAGGTC
AAGAAATTTGCTCATTGAGGTCCCTCACACACAAGGCTGATCTCAACCTGGGTGTTTGGG
CAAAAACCAATCATGGTTGCAATTCTTTCAACATGAATCAAGCACCCAACACCAACTGCTA
TCCAGAAGGACTTCGAAATATGTATTTTCCTTATAATGAGTTTTACAAAGCTGGCCAGGAAC
CTAAAATGTATTCTTGCGCATCTAAACTTCCAAGGGGCAATGTTTTGTTCAATGCTTCCTCA
ATTAAGCCGGGGGTTAGTGTGGACGACATCAGGAAACCAAACCCTCCGAATGACCATAAG
CCAATGGAGAATATCCCTAGTCCCGGTTTTGGAAAAAAATTGAGGAACCAACAGAATGAG
TGCTACAAAGGAAATGTGGCTGGATGTAAACTCTTTGGATTCTCCTTGACTGCGGAATCA
CCTACTCTGAACTCACAAAATTCTGGTAAGCGGAGTTGTACAAAGGTTCACAAGCAAGGC
AGCTTGGTTGGAAGAGCCATTGATCTCTCAAGACTTTATGGATATAAAGACTTGATGACTG
AACTAGAACATCTTTTCGGTATGGAAGGTCTTTTAAATGATCCTGATAAAGGGTGGCGGGT
TTTGTACACTGACAGGGACAATGATGTAATGGTAGTTGGAGACGACCCGTGGCATGAATT
TTGTGATGTGGTATCCAAGATCCATATATACACAGGAGAAGAAGTGGAGAAGATGACCATA
GGAATGGGTAGTGATGAGACTCAAAGCTGTTTGGAAGAAGCAGCAGTGATAATGGAAGCA
TCAAAGTCGTCCTCGGTGGGGCAGCCAGATTCCTCTCCCACTGTAATGAGGGGTATGAA
GATCAGTGTTTGCTTCTAG

>GhARF4-3
ATGGAAATTGATCTGAACCATGCATTAAACGAGGTGGAGAAGACTGTACTTTGCAATGGCA
GCTGTGAAAAAGCTAGTGCTTGCGTTTACTGTTTGTCGTCGTCGTCGTCATCATCATCGTC
ATCATCATCTTCTTCTTCTTCTACTTCATGTACTTCAAACTCAGGTTCACCTCCTTGTTCAT
CTTCCATTGACTTGGAGCTTTGGCATGCTTGTGCTGGTCCTCTCACATCACTTCCAAAGA
AGGGAAATGTGGTTGTATACTTCCCTCAAGGTCACTTGGAACAACTTGCCCTTGCTTTTC
CTTTCTCTCCCTTGGACACCTTTGATCTTCCTTCCCAAATCTTTTGCAAAGTGATGAATGT
CCAGCTTCTGGCCAATAAGGAGAATGATGAGGTCTATACGCAAGTTACTTTACTTCCTCAA
CCGGAGTTGAGAGGGCATAATTTGGGGAGCAAGCAGCTGGATGAGGTAGAGGCGGATG
AGCATGCCGATGGGTCGCCTTCGAAACCAACCTCTCACATGTTTTGCAAGACACTAACAG
CTTCTGACACTAGCATCCATGGAGGGTTCTCTGCTCCTCGTAGAGCTGCTGAAGACTGTT
TTCCTCGGCTGGATTATAAACAGACAAGGCCCTCACAGGAGCTTGTTGCTAAAGACCTCC
ATGGAGTTGAGTGGAGGTTTCGCCATATATATAGGGGTCAACCAAGGCGACATCTCCTTAC
TACCGGCTGGAGTGTTTTTGTGAGTCAAAAGAATCTTATTGCTGGGGATGCAGTGCTCTT
TCTGAGGGGCGAAGATGGGGAGCTGAGGTTGGGAATTAGAAGAGCTGTTCGACCAAGA
ACTGGTCTTCCAGATTCAATTATTGCGCAACATAATTCATATCCAAAGGTTCTTTCTGCAGT
GGCTAATGCATTATCCACCAAAAGCGTGTTTCAAGTCTGCTACTGTCCAAGGGCCAGTCA
TGCCGAGTTCGTCATACCCTTCCAAAAGTATATTAAAAGCATCACAAATCCAATATGCACTG
GGGCAAGATTCAGAATGAAGTTTGAAATGGATGATTTGCCAGAGAGAAGGTGCAACGGT
GTAGTGACAGGAACAGGGGACTCAGATCCTTATAAATGGCCTAACTCAAAGTGGAGATGC
TTAATGGTTAGGTGGGATGATGATATCGCAAGTGATCACCAAGAGAGAGTTTCACCTTGG
GAAATTGATCCTTCTGTTTTTCCCCCACCCTTAATTATTCCATCTCCATTCAAGTTAAAGAA
ACTGCGGACAGGTCTGCAGACTGCTGCACTTGACACCCCTGTCACTGGAGGGGTTGGAT
ATTTGGACTTTGAGGAATCGACAAGATCCTCTAAGGTCTTGCAAGGTCAAGAAAATGTAG
GTTTCATATCACCTGTATATGGATGTGGTACAGTGAACCATCCCCTCCATTTCGAGATGCAA
TCTATGGCACATCAAAGTCTTGCATCGACAGGACTTGGAAAGAGTAACATTAGTGATTTTA
TGAGGGCTCACTCCTCTTCTCACACAGGCTTTGCGGAAACAAATAGGTTTCCGAAGGTCT
TGCAAGGTCAAGAAATTTGCTCATTGAGGTCCCTCACACACAAGGCTGATCTCAACCTTG
GTGTTTGGGCAAAAACCAATCATGGTTGCAATTCTTTCAACATGAATCAAGCACCCAACAC
CAACTTCTATCCAGAAGGACTTCGAAATATGTATTTTCCTTATAATGAGTTTTACAGAGCTG
GCCAGGAACCTAAAATGTGTTCTTATGCATCTACACTTCCAAGGGGCAATGTTTTGTTCAA
TGCTTCCTCAATTAAGCCGGGAGTTAGTATGGACGACATCAGGAAACCAAACCCTCCGAA
TGACCATAAGCCAATGGAGAATATCCCTAGCCCCGGTTTTGGAAAAAAATTGAGGAATCAA

CAGGATGAGTGCTACAAAGGAAATGTGTCTGGATGTAAACTCTTTGGATTCTCCTTGACTG
CGGAATCACCTACTCTGAACTCACAAAATTCTGGTAAGCGGAGTTGTACAAAGGTTCACA
AGCAAGGCAGCTTGGTTGGAAGAGCCATTGATCTCTCAAGACTCTATGGATATAAAGACTT
GATAACTGAACTAGAACATCTTTTCGGTATGGAAGGTCTTTTAAGTGCTCCTGATAAAGGG
TGGCGGGTTTTGTACACTGACAGGGACAATGATGTAATGGTAGTTGGAGACGACCCGTG
GCATGAATTTTGTGATGTGGTATCCAAGATCCACATATACACAGGAGAAGAAGTGGAGAAG
ATGACCATAGGAATGGGTAGTGATGAGACTCAAAGCTGTTTGGAAGAAGCAGCAGTGATA
ATGGAAGCATCAAAGTCGTCCTCGGTGGGGCAGCCAGATTCCTCTCCCACTGTAATGAG
GGGTATGAAGATCAGTGCTTGCTTTTAG

>GhARF5-1
ATGGGTTCCTTTGTTGAAGAGAAGATGAAAACAGGAGTGTTCGGGAGCAGGAAGGCAAT
AAATTCGGAGTTATGGCATGCCTGCGCAGGTCCACTTGTTTCGTTGCCTCAGGTGGGGAA
TCTAGTTTACTACTTCCCTCAAGGACACAGTGAACAGGTATCAGTGTCCACAAAAAGAATG
ATGACCTCTCAAGTTCCCAGCTACCCGAATCTTCAATCTCAGTTGATGTGCCAAGTTCACA
ATGTTACACTACATGCAGACACAGAAACAGATGAAGTTTATGCTCAAATGAGTCTTCAACC
AGTGCACTCTGAAAAAGATGTGTTTAATATACCAGACTTCGGAGTGAACCCCAACAAGCAT
CCAAATGAAATTTTCTGCAAAACTTTGACTGCAAGTGATACAAGTACACATGGTGGCTTTT
CAGTGCCACGTAGGGCAGCAGAGAAGCTCTTTCCTCAGCTGGATTACACAATGCAGCCC
CCATCTCAAGAGCTTGTTATGCGAGACATACATGATAATACTTGGACGTTTCGCCACATATA
CCGCGGGCAACCAAAGCGACATCTTCTTACAACTGGGTGGAGTTCGTTTGTTGGATCAAA
AAGGCTTAGGGCAGGCGATTCCATTCTCTTTATCAGGCATGCTATAGTCACACACTGCATT
GCTTTGAGCTGTAGACTTGCATTTGTTTGGCATGCAAATCGTCATCAAGCAACTTTGCCAT
CATCAGTTTTATCTGCAGATAGTATGCACATTGGTGTCCTTGCCGCTGCTGCTCAAGCTGC
TGCAAATAGAACCCCATTCACAATCTTCTACAATCCAAGATCCTGTCCTTCTGAATTTGTCA
TCCCCATGGCTAAATACTGTAAATCTGTTTACGCAACTCAAGTCTCAGCTGGTATGAGGTT
TGGAATGATGTTTGAAACAGAAGAATCCGGAAAACGTAGATATATGGGTACAATAGTTGGC
ATTAGTGACTTGGATCCTCTGAGTTGGCCTGGCTCGAAGTGGCGAAACCTTCAGGTTGA
GTGGGATGAACCTGGATGTCATGATAAACAGAATAGGATCAGCCCATGGGAAGTTGAAAC
TCCTGAAAGCCTCTTTATTTTTCCTTCTATAAATTCAGGACTCAAGCGACCATATCCTTGGA
TTTTGGGAGCAGAATCTGAATGGGGAAACTTGATCAATAGGCCTCTTCTCCAGTTTCCTG
AAAATGGAAGTGGGAATCTTCCTTACCCAATCTCAAACTCATATTCCAAGCAACTGATGAA
GATGATGCTGAAGCCTCATCTTGTTAACCATCCTGGAACTTTTTCATCCACTTCACAACAA
ATCTCTGCTGCAAAGGGATCTCCATTAAACGAGATGAAGAATGTGCAATATACAAGCAATC
AGGAACCTCAGCTTATGCAATCAGAAAATTTGTTGATAGAAAAACCAAATCCTTCCCAGTT
AGCCCTTGACCAACCTGATCCCATCAACTCAAATTTGCTGAAAATAAATGCTAACGGGAAT
CCACATCCCGCAAACGAGTTTGAAGGCCAAACACAAGCCAGGTGTAATAACAAAAAATTA
AAGTCGGAACCAGAACATTCAACTGATCAGTTAAGTCAGTTGAACTCGACATTGGAATGC
ACTGAGGAAAAATTGGTGGCAAATACTATAAGTCCAACTATATGCAACTAA

>GhARF5-2
ATGGGTTCTGTCGTTGAAGAGAAGATCAAACAAGGAGGTTTGGTTAATGTAGGTGCACAG
TCCACTCTGCTTGAGGAAATGAAGCTATTGAAAGAAATGCAAGATCAATCTGGTACCCGTA
AGGCTATAAATTCCGAGTTATGGCATGCCTGTGCTGGTCCACTTGTTTCCTTGCCTCAGGT
GGGAAGTCTTGTGTATTACTTTCCTCAAGGACATAGCGAACAGGTAGCAGTGTCCACTAA
AAGAATGGCGACTTCTCAAATTCCCAACTACCCAAATCTTCCATCTCAGTTAATGTGCCAA
GTTCATAACGTTACATTACATGCAGACAGAGACACCGACGAAATATATGCCCAAATGAGTC
TTCAACCAGTGAACTCTGAAAAAGATGTGTTCCCTATACCAGACTTCGGATTGAAGCTGA
GCAAGCATCCTAATGAATTTTTCTGCAAAACTTTGACTGCAAGTGATACAAGTACACACGG
TGGTTTTTCAGTGCCACGTAGAGCAGCTGAGAAGCTCTTTCCTTCATTGGATTATTCCATG
CAACCTCCAACGCAAGAGCTTGTTGTGAGAGATTTGCATGATAACACCTGGACGTTTCGT
CATATATACCGTGGGCAGCCGAAGCGACACCTTCTTACTACAGGGTGGAGTTTGTTTGTA
GGATCAAAAAGACTTAGAGCTGGTGATTCCGTTCTCTTTATCAGGGATGAGAAATCACAGT
TATTGGTGGGTGTAAGGCGGGCTAATCGTCAACAAACCACATTGCCATCATCTGTTCTATC
TGCTGATAGTATGCACATTGGTGTCCTTGCTGCCGCCGCTCATGCTGCTGCCAATAGAAG
TCCATTCACAATTTTCTACAATCCAAGAGCATGCCCTTCAGAATTTGTCATCCCTTTGCCTA
GATACCGTAAATCTGTATATGGGTCTCAAGTCTCAGTCGGTATGAGGTTTGGAATGATGTT
CGAAACGGAGGAGTCCGGGAAACGTAGATATATGGGTACAATAGTTGGTATTAGCGACTT
GGATCCTCTAAGATGGCCTGGCTCAAAGTGGCGAAACCTTCAGGTTGAATGGGATGAAC
CTGGATGTAATGATAAACAGAATAGGGTGAGCGCATGGGAAATCGAAACTCCTGAAAGCC

TCTTTATTTTTCCTTCGTTAACTTCAAGTCTGAAGCGACCATTGTATCCTGGATTTTCAGGA
GCAGAATCTGAATGGGGAAGCTTGATGAAAAGGCCCCTACTCCAGTTTCCTGAAAATGGA
AATGGGAATCTTCCCTATTCAATGTCAAATTTATGTTCCGAACAATTGATGAAGATGATGTT
GAAGCCTCAGCTTGTTAACCATCCTGGAATTTTTGCTTCCCCCTTACAACAAATCGCTGAT
GTAAAGATACCTCCACTAGAAGAAATGAAGAACTTGCAGTCTAAAAGCCACCCAAAACCC
CAGGTTATCCAATCAGAAAATATGTTGATAGAGAACCAAAATCTTTCCCACCCAGTCCCTG
ACCATCCTGATCCCATAACTTCAAATATGTCCAAAATCAATGCTAATTGGAACCCACATCCT
GCAAATATTCTAACACAAGCTGGGACTGGGAGCAGTAATGAAAAATTAAAGTTGGAATCAA
AGCATTCAGCCGAGCAACTGACTTCGACATCGGAATGCAATGAGGAAAAATTGGTGGCAA
GTACTCCCCAGATCCCACTCCATGTGCAAAATAATCCCTGGTCGATTCAGTCACAATTGGA
TTCATCAGTCCTCCAAGCTCATCAAATGCTTGTATCCCAAGCTGATATTAGTACTTTAAACA
GCTTTCTTCCTTTCTCAGACACCGATGAGTGGACGTCAAATCTTTCTTCTTGCCAACCTCT
TTCTGGGGCGTACAAATCACCTGGTCCGATACCAATGGTTGGGTTACAGGACTCTTCAGC
TGTCTTTCCAGTTGAAACTGATGATTCGTTAACTACGGTGGGTGAGGAAATATGGGATCCA
AAGCTGAATAGTTGCAGAGTTTCATCCCAAGCAGACCAATTGGCTTCATTCACTCAGCAA
GATCCATGCAGTCTTAATTCTGGTGGGGTAAGGGATTTGTCTGATGACAGCAACAATCAAA
GTGGGATATATAGTAGCTGTCTTAACATTGATGTTAGCAATGGTTGCAGCACTGTGATTGAT
CCTTTTGTTTCCAGTGCCATTCTAGATGAGTTTTGCTCATTGAAAGATGCTGATTTTCAAAA
CCCTTCAGATTGTTTGGTCGGGAACTTTGGTTCTTGTCAGGATGTTCAGTCTCAGATTACC
TCTGCTAGCCTTGCAGATTCTCAAGCTTTCTCTCGACAAGACTTGCCTGACAGCTCCGGT
GGCAATATCGATTTTGATGACAGTGGTCTTCTGCAAAACAATTCCTGGAAGCAAACAGCTC
CACGCGTTAGAACATATACAAAGGTTCAGAAGGCAGGATCTGTAGGAAGGTCGATTGATG
TCACGAGTTTTAAGAATTATGATGAACTAATCTCTGCAATAGAATGCATGTTTGGACTCAAG
GGTCTGCTGGATGATCCCAGAGGTTCAGGCTGGAAATTGGTGTATGTGGATTATGAGAAT
GATGTTCTTCTCGTTGGAGACGATCCTTGGGAGGAATTTGTCGGGTGTGTTCGTTGCATC
CGAATTCTATCCCCTACAGAAGTACAACAGATGAGTGAAGAAGGAATGAAGCTTCTCAAC
AGTGCTGCCACAGTGCAAGGCATCAATGGCTCTAACTCAGATGGTTCCAATGCAAATGCT
TAA

>GhARF5-3
ATGGGTTCTGTCGTTGAAGAGAAGATCAAACAAGGAGGTTTGGTTAATGTAGGTGCACAG
TCCACTCTGCTTGAGGAAATGAAGCTATTGAAAGAAATGCAAGATCAATCTGGTACCCGTA
AGGCTATAAATTCCGAGTTATGGCATGCCTGTGCTGGTCCACTTGTTTCCTTGCCTCAGGT
GGGAAGTCTTGTGTATTACTTTCCTCAAGGACATAGCGAACAGGTAGCAGTGTCCACTAA
AAGAATGGCGACTTCTCAAATTCCCAACTACCCAAATCTTCCATCTCAGTTAATGTGCCAA
GTTCATAACGTTACATTACATGCAGACAGAGACACCGACGAAATATATGCCCAAATGAGTC
TTCAACCAGTGAACTCTGAAAAAGATGTGTTCCCTATACCAGACTTTGGATTGAAGCTGAG
CAAGCATCCTAATGAATTTTTCTGCAAAACTTTGACTGCAAGTGATACAAGTACACACGGT
GGTTTTTCAGTGCCACGTAGAGCAGCTGAGAAGCTCTTTCCTTCATTGGATTATTCCATGC
AACCTCCAACGCAAGAGCTTGTTGTGAGAGATTTGCATGATAACACCTGGACGTTTCGTC
ATATATACCGTGGGCAGCCGAAGCGACACCTTCTTACTACGGGGTGGAGTTTGTTTGTAG
GATCAAAAAGACTTAGAGCTGGTGATTCCGTTCTCTTTATCAGGGATGAGAAATCACAGTT
ATTGGTGGGTGTAAGGCGTGCTAATCGTCAACAAACCACATTGCCATCATCTGTTCTATCT
GCTGATAGTATGCACATTGGTGTCCTTGCTGCCGCCGCTCATGCTGCTGCCAATAGAAGT
CCATTCACAATTTTCTACAATCCAAGAGCATGCCCTTCAGAATTTGTCATCCCTTTGCCTAG
ATACCGTAAATCTGTATATGGGTCTCAAGTCTCAGTCGGTATGAGGTTTGGAATGATGTTT
GAAACGGAGGAGTCCGGGAAACGTAGATATATGGGTACAATAGTTGGTATTAGCGACTTG
GATCCTCTAAGATGGCCTGGCTCGAAGTGGCGAAACCTTCAGGTTGAATGGGATGAACC
TGGATGTAATGATAAACAGAATAGGGTGAGCGCATGGGAAATCGAAACTCCTGAAAGCCT
CTTTATTTTTCCTTCGTTAACTTCAAGTCTGAAGCGACCATTGTATCCTGGATTTTCAGCAG
CAGAATCTGAATGGGGAAGCTTGATGAAAAGGCCCCTACTCCAGTTTCCTGAAAATGGAA
ACGGGAATCTTCCCTATTCAATGTCGAATTTATGTTCTGAACAATTGATGAAGATGATGTTG
AAGCCTCAGCTTGTTAACCATCCTGGAATTTTTGCTTCCCCCTTACAACAAATCGCTGATG
TAAAGGTACCTCCATTAGAAGAAATGAAGAACTTGCAGTCTAAAAGCCACCCAAAACCCC
AGGTTATCCAATCAGAAAATATGTTGATAGAGAACCGAAATCTTTCCCACCCAGTCCCTGA
CCAACCTGATCCCATAACTTCAAATATGTCCAAAATCAATGCTAATGGGAACCCACATCCT
GCAAATATTCTAACACAAGCTGGGACTGGGAGCAGTAATGAAAAATTAAAGTTGGATTCAA
AGCATTCAGCCGAGCAGCTGACTTCGACATCGGAATGCAATGAGGAAAAATTGGTGGCA
AGTACTCCCCATATTCCACTCCAAGTGCAAAATAATCCCTGGTCGATTCAGTCACAGTTGG
ATTCGTCAGTCCTCCAAGCTCATCAAATGCTTGTATCCCAAGCTGATATTAGTACTTTAAAC

AGCTTTCTTCCTTTCTCAGACACTGATGAGTGGACGTCAAATCTTTCTTCTTGCCAACCTC
TTTCTGGGGCATACAAATCACCTGGTCCGATACCAATGGTTGGGTTACAGGACTCTTCAG
CTGTCTTTCCAGTTGAAACTGATGATTCGTTAACTACGGTGGGTGAGGAAATATGGGATCA
AAAGCTGAATAATTGCAGAGTTTCATCCCAAGCAGACCAATTGGCTTCATTCACTGAGCAA
GATCCATGCAGTCTTAATTCTGGTGGGGTGAGGGATTTGTCTGATGACAGCAACAATCAA
AGTGGGATATATAGTAGCTGTCTTAACATTGATGTTAGCAATGGTTGCAGCACCGTGATTGA
TCCTTTTGTTTCCAGTGCCATTCTAGATGAGTTTTGCTCATTGAAAGATGCTGATTTTCAAA
ACCCTTCAGATTGTTTGGTTGGGAACTTTAGTTCTTGTCAGGATGTTCAGTCTCAGATTAC
CTCTGCTAGCCTTGCAGATTCTCAAGCTTTCTCTCGACAAGACTTGCCTGACAGCTCCGG
TGGCAATATCGATTTTGATGACAGTGGCCTTCTGCAAAACAATTCCTGGAAGCAAACAGCT
CCACGCGTTAGAACATATACAAAGGTTCAGAAGGCAGGATCTGTAGGAAGGTCGATTGAT
GTCACGAGTTTTAAGAATTATGATGAACTAATCTCTGCAATAGAATGCATGTTTGGACTCAA
GGGTCTGCTGGATGATCCCAGAGGTTCAGGCTGGAAATTGGTGTATGTGGATTATGAGAA
TGATGTTCTTCTCGTTGGAGACGATCCTTGGGAGGAATTTGTTGGGTGTGTTCGTTGCAT
CCGAATTCTATCCCCTACAGAAGTACAACAGATGAGTGAAGAAGGAATGAAGCTTCTCAA
CAGTGCTGCCACAGTGCAAGGCATCAATGGCTCTAACTCAGAAGGTTCCAATGCAAATGC
TTAA

>GhARF6-1
ATGAGGCTTTCTTCAGCTGGTTTCAGTCCACAAGCTCCGGAAGACGTTGCAGGAGAGAA
GAGAGTTCTGAACTCTGAACTTTGGCATGCATGTGCGGGTCCTCTTGTTTCTCTACCTCC
GGTTGGAAGTAGGGTTGTTTACTTCCCACAGGGTCATAGCGAACAGGTAGCTGCATCAAC
CAACAAGGAGGAAGTGGATGTCCACGTACCTAACTACCCAAGCTTACCTCCACAACTTATA
TGTCAGCTCCATAATGTCACCATGCATGCAGATGTTGAGACAGATGAAGTATATGCACAAA
TGACATTGCAACCACTGAATCCGCAAGAACAGAAGGAGGCTTACCTTCCAGCAGAATTGG
GCACTCCCAGCAGACAGCCAACAAATTATTTCTGTAAAACATTAACAGCCAGTGACACAA
GCACTCATGGAGGGTTCTCTGTTCCTCGCCGAGCTGCTGAAAAAGTGTTTCCCCCACTG
GACTTCTCCCAGCAGCCTCCTGCTCAAGAGTTGATTGCAAGGGACTTACACGATAATGAA
TGGAAATTTAGGCATATATTTCGGGGTCAGCCCAAAAGGCATCTCTTGACAACAGGATGG
AGCGTATTTGTAAGTGCTAAAAGACTAGTTGCAGGTGATTCAGTTCTTTTTATCTGGAATGA
AAAAAATCAATTACTTCTTGGCATCCGACGAGCTAATCGACCTCAAACTGTAATGCCTTCAT
CTGTTTTATCGAGTGATAGCATGCACTTAGGGCTTCTTGCTGCTGCTGCTCATGCAGCTTC
AACAAATAGCCGCTTCACTATATTTTATAACCCAAGGGCTAGTCCATCAGAATTTGTAATAC
CTTTGACAAAATATATCAAAGCTGTCTATCATACTCGAGTTTCTGTTGGTATGCGCTTTAGA
ATGCTGTTTGAAACAGAAGAATCAAGTGTTCGTCGATACATGGGTACAATCACTGGCATAA
GTGACTTGGATCCTGTTGGCTGGGATGAATCAACAGCTGGAGAAAGGCAGCCTAGAGTC
TCCTTGTGGGAGATTGAACCATTGACAACATTCCCTATGTATCCATCACCATTTCCATTAAG
ACTTAAGCGACCATGGCCAGCAGGATTACCTTCTTTCCACGGCATCAAGGATGATGGTCT
AGGTATGAATTCTCTGCTTATGTGGCTACAAGGAGATGCAGGTAGAGGAATGCCATCTCT
GAATTTTCCAGGGATTGGAGTTACACCGTGGATGCAGCCAAGGCTGGATGCTGCTTCCAT
GCTGGGTTTGCAGACTGACATGCACCAAGCTATGGCTGCTGCTGCTCTGCAAGACATGA
GAGCAGTGGATCCCTCCAAATCAGCAACTACTACCCTTCTGCAATTCCAGCAACCCCAAA
ATCTATCCTGCAGGCCTGCTGCTTTAATGCATTCCCAGATGTTGCAGCAGTCTCAGCCTC
AGGCTTTTCTTCAGGGTGTTGAAGACAACCAACATCAGTCTGAGACTCAGGCTCAAACCC
AACCGCCTCTTGTTCAGCAACAATTGCAGCAGCAGAATTCATTTAATAACCATCAACACCA
ACAGCAGTTACAGCATCCGCTGTCACAGCAACACCAGCAACTGGTCGATCATCAGCATAT
TTCTACTGGAGTGTCTGCCATGTCACAGTATGCTCCGGCCTCACAGTCCCGGTCGTCAC
CTTTCCAAGCCATACCTTCACTATGCCAACAACAGAGTTTTTCTGACTCAAATGGGCACAC
CGTGACCAGCCCCATTGTATCTCCCTTGCACAGTCTTTTGGGATCCTTTCCCCAAGATGA
ACCGTCCGGTCTGCTCAACTTGCCTAGATCCAACCCAGTAATAACATCTGCAGCATGGCC
ATCTAAGCGGGCTGCTGTTGAAGTTCTGTCATCTGGATCTCCACAATGTGTTCTGCCCCA
GGTGGAACAGTTGGGGCCCACCCAAACAAACATTTCTCATAATTCTATTTCGTTGCCACC
CTTTCCTGGCAGGGAGTGCTCGATAGACCAAGCAGCGGGTACTGATCCACAGAGCCATC
TCTTATTTGGTGTTAATATAGAGACTTCATCTCTTCTATTGCAAAACGGGATGTCAAGCCTT
AGGGGAATTGGCAGCGAGAGTGACTCCACTACTATACCTTTCTCTTCTAATTATGCGAGTA
CTGCAGGCACTGATTTTTCAGTTAATCCAGCAATGACACCTTCCAGTTGCATTGATGAATT
GGGATTCTTGCAGTCTCCAGAGAATGTGGGCCAAGAAAACCCACAAACCAGAACCTTTG
TTAAGGTTTATAAATCAGGGTCCTTCGGGAGGTTGTTGGATATCTCCAAATTTAACAGCTAC
AATGAGCTGCGCAGTGAACTCGCACGCATGTTTGGCCTTGAAGGCCAGTTGGAGGACCC
TTTGAGATCAGGCTGGCAGCTTGTATTTGTTGATCGGGAGAATGACGTTCTTCTCCTTGG

TGATGATCCTTGGCCGGAGTTTGTGAACAGTGTGTGGTGCATCAAGATACTTTCACCGCA
AGAAGTGCAGCAAATGGGCAAACGAGGCCTGGAGCTTCTAAACTCTGTTCCAGTTCAGA
GGCTCTCGAATGGCAGTTGTGATGACTATGCGAGCCAGCAGGACTCCAGAAATTTGAGC
TCTGGTATTGCCTCTGTGGGGTCATTGGACTACTGA

>GhARF6-2
ATGAGGCTTTCTTCAGCTGGTTTCAGTCCACAAGCTCCGGAAGGAGAGAAGAGAGTTCT
GAACTCTGAACTTTGGCATGCATGTGCGGGTCCTCTTGTTTCTCTACCTCCAGTTGGAAG
TAGGGTTGTTTACTTCCCACAGGGTCATAGCGAACAGGTAGCTGCATCAACCAACAAGGA
GGAAGTGGATGTCCACGTACCTAACTACCCAAGCTTACCTCCACAACTTATATGTCAGCTC
CATAATGTCACTATGCATGCAGATGTTGAGACAGATGAAGTATATGCACAAATGACATTGCA
ACCACTGAATCCGCAAGAACAGAAGGAGGCTTACCTTCCAGCAGAATTGGGCACTCCCA
GCAGACAGCCAACAAATTATTTCTGTAAAACATTAACAGCCAGTGACACAAGCACTCATGG
AGGGTTCTCTGTTCCTCGCCGAGCTGCTGAAAAAGTGTTTCCCCCACTGGACTTCTCCC
AGCAGCCTCCTGCTCAAGAGTTGATTGCAAGGGACTTACACGATAATGAATGGAAATTTAG
GCATATATTTCGGGGTCAGCCCAAAAGGCACCTCTTGACAACAGGATGGAGCGTATTTGT
AAGTGCTAAAAGACTAGTTGCAGGTGATTCAGTTCTTTTTATCTGGAATGAAAAAAATCAAT
TACTTCTTGGCATCCGACGAGCTAATCGACCTCAAACTGTAATGCCTTCATCTGTTTTATCA
AGTGATAGCATGCACTTAGGGCTTCTTGCTGCCGCTGCTCATGCAGCTTTAACAAATAGC
CGCTTCACTATATTTTATAACCCAAGGGCTAGTCCATCAGAATTTGTAATACCTTTAACAAAA
TATATCAAAGCTGTCTATCATACTCGAGTTTCTGTTGGTATGCGCTTTAGAATGCTGTTTGA
AACAGAAGAATCAAGTGTTCGCCGATACATGGGTACAATCACTGGCATAAGTGACTTGGAT
CCTGTTGGCTGGGATGAATCAACAGCTGGAGACAGGCAGCCTAGAGTCTCCTTGTGGGA
GATTGAACCATTGACAACATTCCCTATGTATCCATCACCATTTCCATTAAGACTTAAGCGAC
CATGGCCAGCAGGATTACCTTCTTTCCACGGCATCAAGGATGATGGTCTAGGCATGAATT
CTCCACTTATGTGGCTACAAGGAGATGCAGGTAGAGGAATGCCATCTCTGAATTTTCCAG
GGATTGGAGTTACACCGTGGATGCAGCCAAGGCTGGATGCTGCTTCCATGCTGGGTTTG
CAGACTGACATTCACCAAGCTATGGCTGCAGCTGCGCTGCAAGAAATGAGAGCAGTGGA
TCCCTCCAAATCAGCAACTACTACCCTTCTGCAATTCCAGCAACCCCAAAATCTATCCTGC
AGGCCTGCTGCTTTAATGCAGTCCCAGATGTTGCAGCAGTCTCAGCCTCAGGCTTTTCTT
CAGGGTGTTGAAGATAACCAACATCAGTCTCAGACTCAGGCTCAAACCCAACCGCCTCTT
GTTCAGCAACAATTGCAGCAGCAGAATTCATTTAATAACCATCAACACCAACAGCAGTTAC
AGCATCCGCTGTCACAGCAACACCAGCAACTGGTCGATCATCAGCATATTTCTACTGGAG
TGTCTGCCATGTCACAGTATACTCCGGCCTCACAGTCCCGGTCGTCACCTTTCCAAGCCA
TACCTTCACTATGCCAACAACAGAGTTTTTCTGACTCAAATGGGCACACCATGACCAGCC
CTATTATATCTCCCTTGCATGGTCTTTTGGGATCCTTTCCCCAAGATGAATCGTCCAGTCTG
CTCAACTTGCCTAGATCCAACCCAGTAATAACATCTGCTGCATGGCCATCTAAGCGGGCT
GCTGTTGAAGTTCTGTCATCTGGATCTCCACAATGTGTTCTGCCCCAGGGGGAACAGTTG
GGGCCCACCCAAACAAACATTTGTCATAATTCTATTTCGTTGCCACCCTTTCCTGGCAGG
GAGTGCTCGATAGACCAAGCAGGGGGTACTGATCCACAGAGCCATCTCTTATTTGGTGTT
AATATAGAGCCTTCATCTCTTCTATTGCAAAACGGGATGTCAAGCCTTAGGGGAGTTGGCA
GCGAGAGTGACTCCACTACCATACCCTTCTCTTCTAATTATGCGAGTACTGCAGGCACTGA
TTTTTCAGTTAATCCAGCAATGACACCTTCCAGTTGCATTGATGAATTGGGATTCTTGCAG
TCTCCAGAGAATGCGGGCCAAGAAAACCCACAAACTAGAACCTTTGTTAAGGTTTATAAAT
CAGGGTCCTTCGGGAGGTCGTTGGATATCTCCAAATTTAGCAGCTACAATGAGCTGCGCA
GTGAACTCGCTCGCATGTTTGGCCTTGAAGGCCAGTTGGAGGACCCTTTGAGATCAGGC
TGGCAGCTTGTATTTGTTGATCGGGAGAATGATGTTCTTCTCCTTGGTGATGATCCTTGGC
CGGAGTTTGTGAACAGTGTGTGGTGCATCAAGATACTTTCACCGCAAGAAGTGCAGCAAA
TGGGCAAACGAGGCCTGGAGCTTCTAAACTCTGTTCCAGTTCAGAGGCTCTCGAATGGC
AGTTGTGATGACTATGCGAGCCAGCAGGACTCGAGAAATTTGAGCTCTGGTATTGCGTCT
GTGGGGTCATTGGACTACTGA

>GhARF6-3
ATGAGACTTTCTTCTGCTGGTTTCAATCCACCAAACCAGGAAGACACTGCAGGAGAAAAG
AGGGTCCTCAACTCTGAACTGTGGCATGCGTGTGCTGGACCTCTTGTTTCTCTACCACAT
GTTGGAAGTAGGGTTGTTTATTTCCCACAGGGTCATAGTGAGCAGGTAGCTGCAACAACC
AACAAGGAAGTGGATGCCCACATACCTAACTACCCAAGCTTACCTCCACAACTTATTTGTC
AGCTTCATAATGTTACCATGCATGCTGATGTCGAAACAGATGAAGTATATGCACAGATGAC
CTTGCAACCGTTGAGTCCGCAAGAACAAAAGGAGGCTTATCTACCTGCGGAATTGGGCA
CTCCCAGCAAACAGCCAACAAACTATTTCTGCAAAACATTAACAGCCAGTGACACAAGCA

CTCATGGAGGGTTTTCTGTTCCTCGCCGAGCAGCTGAAAAAGTGTTTCCTCCACTGGACT
TCACCCAGCAGCCTCCAGCTCAAGAGTTAATCGCAAGAGACTTGCATGATAATGAATGGA
AATTTAGGCATATATTTCGGGGCCAGCCCAAAAGGCACCTCTTGACGACTGGATGGAGTG
TCTTTGTAAGTGCTAAAAGACTAGTTGCAGGTGATTCAGTGCTTTTTATATGGAATGATAAA
AATCAATTACTTCTTGGCATCCGGCGAGCTAATCGACCTCAAACTGTAATGCCTTCATCAG
TGCTATCAAGTGATAGCATGCATTTAGGGCTTCTTGCTGCCGCTGCTCATGCAGCTGCAA
CAAATAGTCGGTTCACTATATTTTATAATCCAAGGGCTAGTCCATCAGAATTTGTCATAACTC
TCTCAAAATATGTCAAAGCTGTCTATCATACTCGAGTTTCTGTTGGAATGCGCTTTAGGATG
CTGTTTGAAACAGAAGAATCCAGTGTTCGTCGATACATGGGGACGATAACTGGCATAAGT
GACTTAGATCCTGTTCGGTGGCCAAATTCACATTGGAGATCAGTCAAGGTTGGCTGGGAT
GAATCAACAGCTGGTGAAAGGCAGCCAAGAGTTTCCTTATGGGAAATTGAACCATTGACA
ACATTCCCAATGTATCCATCTCCATTTCCTTTAAGGCTCAAGCGCCCATGGCCTCCAGGAT
TACCTTCTTTCCATGGCCTCAAGGATGATGATCTAGGCATGAGTTCACTTATGTGGCTACA
AGGAGATGCTGGTCGAGGAATGCAGCATCTAAATTTTCAGGGTATTGGAGTTTCACCATG
GATGCAGTCGAGGCTAGATGCCTCCATGCTTGGTTTGCAGACTGACATGTACCAAGCTAT
GGCTGCTGCAGCATTGCAAGAGATGAGAACTGTGGATCCTTCCAGATCTGGAACTGCTT
CCCTTCAATTCCAGCAACCCCAAAATGGCCCTTGCAGGCCTGCTGCTCTAATGCAACCCC
AGATGTTGCAGCAGACTCTGCCTCAGGCCATTCTTCAGGGTGTTGAAGACAACCAGCAT
CAGTCTCAGCCACATCTACTTCAGCAACAATTGCAGCACCAGAATTCATTTAATAACCAAC
AGCAACACCAGCAGCAGCCTTTGTTTAACAACCAACAGCAACAGCAGCCACAGCATTCAA
TGTCCCAGCAGCACCAGCAACTGGTTGATCATCAGCAGATTCCTAGTCCAGTGTCTGCCA
TGTCACAGTATGCTTCAGCCTCTCAATCTCAGTCATCACCGTTGCAATCCATACCTTCACT
AGGCCAACAACAGAGTTTTTCTGATTCAAACGGGAACCCTGTGACCAGCCCTGTTGTTTC
TCCTTTACATAGTCTTTTGGGTTCTTTCCCCCAAGATGAGTCTTCCAATCTGCTCAACTTG
CCTAGAACTAACCCCATAATGACTACTTCTGCATGGCCATCTAAGCGGGTTGCCGTTGAC
GTTCTCTCATCTGGATCTCCACAGTGTGTTCTACCACAGGTGGAACAGTTGGGGCCTTCC
CAGACAAACATGTCTCAAAATTCTATTTCGTTGCCACCTTTTCCTGGTAGGGAGTGCTCGA
TAGACCAGGGGGGTACCGACCCACAGAGCCATCTCTTATTTGGTGTTAATATAGAGCCTT
CATCTGTTCTAATGCAAAATGGAATGTCAGGACTTAGGGGAGTTGGCACTGATAGTGATTC
CACTTCTATACCCTTCTTTTCTAATTATATGAGTACTGCAGGGAATGATTTTTCGGTTAATCC
AGCAATGACACCTTCCAGTTGCATTGATGAATCTGGATTCTTGCAGTCTCAGGAAAATGTG
GGCCAATCAAACCCACAAACTAGAACCTTTGTTAAGGTTTATAAATCAGGGTCCTTCGGGA
GATCATTGGATATTTCTGCATTCAGCAACTACAATGAACTTCGCAGTGAACTGGCACATAT
GTTTGGCCTTGAAGGCCAGTTGGAGGACCCTCTGAGATCAGGCTGGCAGCTTGTATTTG
TTGACCGGGAGAATGATGTTCTTCTCCTTGGCGATGACCCCTGGCCGGAGTTCGTGAAC
AGTGTCTGGTGTATCAAGATACTTTCCCCACAGGAAGTGCAGCAAATGGGCAAACGAGG
CCTGGAGCTTCTAAACTCTGTGCCAGTTCAGAGGCTCTCTAATGGCAGTTGTGATGACTA
TGTGAGCCGGCAGGACTCGAGAAATCTTAGCTCTGGTATCGCCTCCGTGGGTTCTTTGG
ACTACTGA

>GhARF6-4
ATGAGACTTTCTTCAGCTGGTTTCAATCCTCCAAACCAGGAAGACACTGCAGGAGAAAAG
AGGGTCCTCAACTCTGAACTGTGGCATGCGTGTGCTGGACCTCTTGTTTCTCTACCACAT
GTTGGAAGTAGGGTTGTTTATTTCCCACAGGGTCATAGTGAGCAGGTAGCTGCAACAACC
AACAAGGAAGTGGATGCCCACATACCGAACTACCCAAGCTTACCTCCACAACTTATTTGTC
AGCTTCATAATGTTACCATGCATGCAGATGTTGAAACAGATGAAGTATATGCACAGATGAC
CTTGCAACCGTTGAGTCCGCAAGAACAAAAGAAGGCTTATCTACCTGCGGAATTGGGCA
CTCCCAGCAAACAGCCAACAAACTATTTCTGCAAAACATTAACAGCCAGTGACACAAGCA
CTCATGGAGGGTTTTCTGTTCCTCGCCGAGCAGCTGAAAAAGTGTTTCCTCCACTGGACT
TCTCCCAGCAGCCTCCAGCTCAAGAGTTAATCGCAAGAGACTTGCATGATAATGAATGGA
AATTTAGGCATATATTTCGGGGCCAGCCCAAAAGGCACCTCTTGACGACTGGATGGAGTG
TCTTTGTAAGTGCTAAAAGACTAGTTGCAGGTGATTCAGTGCTTTTTATATGGAATGATAAA
AATCAATTACTTCTTGGCATCCGGCAAGCTAATCGACCTCAAACTGTAATGCCTTCATCAG
TGCTATCAAGTGATAGCATGCATTTAGGGCTTCTTGCTGCTGCTGCTCATGCAGCTGCAAC
AAATAGCCGGTTCACTATATTTTATAATCCAAGGGCTAGTCCATCAGAATTTGTCATAAATCT
CTCAAAATATGTCAAAGCTGTCTATCATACTCGAGTTTCTGTTGGAATGCGCTTTAGGATG
CTGTTTGAAACAGAAGAATCCAGTGTTCGTCGATACATGGGGACGATAACTGGCATAAGT
GACTTAGATCCTGTTCGGTGGCCAAATTCACATTGGAGATCAGTCAAGGTTGGCTGGGAT
GAATCAACAGCTGGTGAAAGGCAGCCAAGGGTTTCCTTATGGGAAATTGAACCATTGACA
ACATTCCCAATGTATCCATCTCCATTTCCTTTAAGGCTTAAGCGCCCATGGCCTCCAGGAT

TACCTTCTTTCCATGGCCTCAAGGATGATGATCTTGGCATGAGTTCACTTATGTGGCTACA
AGGAGATGCTGGTCGAGGAATGCAGCATCTAAATTTTCAGGGTATTGGAGTTTCGCCATG
GATGCAGCCGAGGCTAGATGCCTCCATGCTTGGTTTGCAGACTGACATGTACCAAGCTAT
GGCTGCTGCAGCATTGCAAGAGATGAGAACTGTGGATCCTTCCAGATCTGGAGCTGCTT
CCCTTCAATTCCAGCAACCCCAAAATGGCCCTTGCAGGCCTGCTGCTCTAGTGCAACCC
CAGATGTTGCAGCAGTCTCTGCCTCAGGCCATTCTTCAGGGTGTTGAAGACAACCAGCAT
CAGTCTCAGCCACATCTACTTCAGCAACAATTGCAGCACCAGAATTCATTTAATAACCAAC
AGCAACACCAGCAGCAGCCTTTGTTTAACGACCAACAGCAACAGCAGCCACAGCATTCA
ATGTCCCAGCAGCACCAGCAACTGGTTGATCATCAGCAGATTCCTAGTCCAGTGTCTGCC
ATGTCACAGTATGCTTCAGCCTCTCAATCTCAGTCATCACCGTTGCAATCCATACCTTCAC
TAGGCCAACAACAGAGTTTTTCTGATTCAAACGGGAACCCTGTGACCAGCCCTGTTGTTT
CTCCTTTACATAGTCTTTTGGGTTCTTTCCCCCAAGATGAGTCTTCCAATCTGCTCAACTT
GCCTAGAACTACCCCTGTAATGACTACTTCTGCATGGCCATCTAAGCGGGCTGCCGTTGA
CGTTCTCTCATCTGGATCTCCACAGTGTGTTCTACCACAGGTGGAACAGTTGGGGCCTTC
CCAGACAAACATGTCTCAAAATTCTATTTCATTGCCACCTTTTCCTGGCAGGGAGTGCTCG
ATAGACCAGGGGGGTACCGACCCACAGAGCCATCTCTTATTTGGTGTTAATATAGAGCCG
TCATCTGTTCTAATGCAAAATGGAATGTCAGGCCTTAGGGGAGTTGGCACTGATAGTGATT
CCACTTCTATACCCTTCTTTTCTAATTATATGAGTACTGCAGGGAATGATTTTTCGGTTAATC
CAGCAATGACACCTTCCAGTTGCATTGATAAATCTGGATTTTTGCAGTCTCAGGAAAATGT
GGGCCAATCAAACCCACAAACTAGAACCTTTGTTAAGGTTTATAAATCAGGGTCCTTCGG
GAGATCATTGGATATTTCTGCATTCAGCAACTACAATGAACTTCGCAGTGAACTGGCACAT
ATGTTTGGCCTTGAAGGCCAGTTGGAGGACCCTCTGAGATCAGGCTGGCAGCTTGTATTT
GTTGACCGGGAGAATGATGTTCTTCTCCTTGGCGATGACCCCTGGCCGGAGTTCGTGAA
CAGTGTCGGGTGTATCAAGATACTTTCCCCACAGGAAGTGCAGCAAATGGGCCAACGAG
GCCTGGAGCTTCTAAACTCTGTGCCAGTTCAGAGGCTCTCTAATGGCAGTTGTGATGACT
ATGTGAGCCGGCAGGATTCGAGAAATCTTAGCTCTGGTATCGCCTCCGTGGGTTCTTTGG
ACTACTGA

>GhARF6-5
ATGAGACTCTCTTCATCAGGGTTGAATCAGCAAACTCAGGAAGGGGAGAAGAAATGTTTG
AATTCTGAACTTTGGCATGCATGTGCTGGTCCTTTGGTGTCTCTTCCACCTGTTGGAAGC
CGAGTTGTTTACTTTCCACAAGGTCATAGTGAACAGGTTGCTGCTTCTACTAACAAGGAAG
TAGATGCTCATATACCGAACTATCCGAGCTTACCCCCACAACTTATCTGTCAGCTTCACAAT
GTGACCATGCATGCAGATGTGGAAACAGATGAAGTGTATGCTCAAATGACCTTGCAGCCA
CTGAGTCCACAAGAGCAAAAGGATGTCTACCTACTGCCTGCAGAACTAGGTGCTCCCAG
CAAACAGCCAACCAATTATTTTTGTAAAACACTAACAGCAAGTGACACTAGCACTCATGGA
GGATTCTCTGTTCCGCGCCGTGCTGCTGAGAAAGTCTTTCCTCCTCTTGACTACACCCAG
CAACCACCAGCTCAAGAGTTGATTGCCAGGGATCTTCATGACAATGAGTGGAAATTTAGA
CATATATTTCGAGGCGAGAATGATAAGAATCAATTGTTGTTGGGTATACGGAGAGCAAACC
GTCCTCAGACTGTTATGCCTTCTTCTGTTTTATCAAGTGATAGCATGCACATTGGTCTTCTT
GCTGCTGCAGCTCATGCGGCTGCAACCAATAGCCGTTTTACTATATTTTACAACCCAAGGG
CTAGTCCTTCAGAGTTTGTCATTCCTCTTGCCAAGTATGTTAAAGCTATCTCTCACACCCG
GATTTCTGTAGGCATGCGGTTTAGAATGCTGTTTGAGACAGAAGAGTCAAGTGTCCGACG
ATACATGGGAACAATAACTGGCATTAGTTACTTAGATCCTGTTCGCTGGCCAAATTCGCATT
GGCGCTCTGTTAAGGTTGGCTGGGATGAATCCACTGCAGGAGAGAGGCAGCCAAGAGTT
TCCTTGTGGGAGGTTGAACCGTTAACAACCTTCCCTATGTATCCATCTCCCTTCCCCCTGC
GGCTGAAGCGACCATGGCCGTCTGCACTACCCTCCTTCCATGCTTTCAAAGACGGTGATA
TGAGCATTAATTCCCAATTGATGTGGCTTCAAGGTGGGGTCGGGGATCAAGGACTTCAGT
CTTTAAACTTCCAAGGATTTGGAGTTGCACCTTGGATGCAACCAAGGCTTGATACTTCTTC
AATACCAGGTGTACAACCTGATCTTTACCAAGCAATGGTGACTGCAGCACTTCAGGATATG
AGGACTGTTGATTCCTCCAAAATAGGCTCTCAATCTCTCTTGCAATGCCAGCAAAACCAGA
GCACATCCACTGGGCTGCCTGCTCTAGTTCAGAGGCAAATGTTACAACAGTCCCAAACAC
AAAATGGGTTTCTTCCAAGTTTTCAGGAGAATCAGACTGCTTCACAGGTTCAACTTCTGCA
GCAGTTGCAGTGCCCCAATTTGTACAGTGACCAGCGACAAAAGCAGCAGCAGCAACAGT
CTCAAGAAACACAGCAACTGCCACCTGTTCCACAGCAGATTTCTAATGTAATCCCTGCTTT
TCCTTCTGTGTCAGCCAACCAAGCGCAGTCATCTCTGCCAGCTGTTGATTCACAATGCCA
GCAGTCGACATTTTCCGACCATCTTGGGAACTCTGTAGCTACATCTGATGTTTCTTCTATG
CAAAGCATCTTAGGTTCATTATCCCAGATGGGAGCTTCCCATTTACTCAACTTGAATGGAT
CAAACCCAATTCTCTCTTCTTCTACTTTCTTGTCCAAGCCTGCAGCTATTGAACCACGGCT
TTCATCCGGAATGGCCAACTCTGTACTTCCCCAGGTTGAACAGTTGGGAACTGCACAATC

AAATGCATCTGAACCTAACAACTTATTACCTCCATTTCCTGGAAGGGAGTATTCTGCTTACC
ACAATGCAACTGATCCACAGAACAATCTTCTCTTTGGGGTTAGCATTGATTCCTCGTCTCT
TATGCTGCATCATGGGATGACAAACCCGAAAAGCATCAGAAATGAGAATGATTCGATGTCC
CTTCCGTACGCTGCTTCAAATTTCACAAGTGCTTCTGGCACAGATTTTCCTCTTAATTCAG
ACATGACTACCTCAAGTTGTGTCGATGAATCAGGTTACTTGCAGTCCTCTGAAAATGTGGA
CCAAGTAAACCCTCCAACTGGAACCTTTGTCAAGGTTCACAAGTCGGGGTCCTTTGGGC
GCTCATTGGATATTTCCAAGTTTGGCAGCTATGATGAGCTGCGCTGTGAGCTTGCTCGAC
TATTTGGCCTTGAAGGCCAATTAGAGGACCCTCAGAGATCAGGCTGGCAGCTTGTATTTG
TTGACAGGGAGAACGATATTCTTCTCCTTGGTGATGACCCATGGCAGGAATTCGTGAACA
ATGTGTGGTATATCAAGATACTATCCCCACTTGAAGTACAACAAATGGGGAAAGGTCAGAA
TCCTGCTACTTCTATTCCGAACCAAAGGCTTAACACCACCACCACCGCTACCACCACCAG
CAGCAATGGCAACCACTGTGATGACTACATGAGCAGACAGGACTCGAGAAGTTCTGTTG
CATCAATGGGGTCTCTTGAATACTAA

>GhARF6-6
ATGAGACTCTCTTCATCAGGGTTGAATCAGCAAACACAGGAAGGGGAGAAGAAATGTTTG
AATTCTGAACTTTGGCATGCATGTGCTGGTCCTTTGGTTTCTCTTCCACCTGTTGGAAGC
CGAGTTGTTTACTTTCCACAAGGTCATAGTGAACAGGTTGCTGCTTCTACTAACAAGGAAG
TAGATGCTCATATACCGAACTATCCGAGCTTACCCCCACAACTTATCTGTCAGCTTCACAAT
GTGACCATGCATGCAGATGTGGAAACAGATGAAGTGTATGCTCAAATGACCTTGCAGCCA
CTGAGTCCACAAGAGCAAAAGGATGTCTACCTACTGCCTGCAGAACTAGGTGCTCCCAG
CAGACAGCCAACCAATTATTTTTGTAAAACACTAACAGCAAGTGACACTAGCACTCATGGA
GGATTCTCTGTTCCTCGCCGTGCTGCTGAGAAAGTCTTTCCTCCTCTTGACTACACCCAG
CAACCACCAGCTCAAGAGTTGATTGCCAGGGATCTTCATGACAATGAGTGGAAATTTAGA
CATATATTTCGAGGCGATTTGTCCCTGCTCTCATATGCAGGTCAGCCAAAGAGGCATCTTC
TTACAACTGGGTGGAGTGTTTTTGTAAGTGCAAAGAGACTAGTTGCTGGTGACTCAGTCC
TTTTTATCTGGAATGATAAGAATCAATTGTTGTTGGGTATACGGAGAGCAAACCGTCCTCA
GACTGTTATGCCTTCTTCTGTTTTATCTAGTGATAGCATGCACATTGGTCTTCTTGCTGCTG
CAGCTCATGCGGCTGCAACCAATAGCCGTTTTACTATATTTTACAACCCAAGGGCTAGTCC
TTCAGAGTTTGTCATTCCTCTTGCCAAGTATGTTAAAGCTATCTCTCACACCCGGGTTTCT
GTAGGCATGCGATTTAGAATGCTGTTTGAGACAGAAGAGTCAAGTGTCCGACGATACATG
GGTACAATAACTGGCATTAGTGACTTAGATCCTGTTCGCTGGCCAAATTCGCATTGGCGCT
CTGTTAAGGTTGGCTGGGATGAATCTACTGCAGGAGAGAGGCAGCCAAGAGTTTCCTTG
TGGGAGATTGAACCGTTAACAACCTTCCCTATGTATCCATCTCCCTTCCCCCTGCGGCTG
AAGCGACCATGGCCGTCTATACTACCCTCCTTCTATGCTTTCAAAGATGGTGATATGAGCA
TTAATTCCCAATTGATGTGGCTTCAAGGTGGGGTTGGGGATCAAGGACTTCAGTCTTTAAA
CTTCCAAGGATTTGGAGTTGCACCTTGGATGCAACCAAGGCTTGATACTTCTTCAATACCA
GGTGTCCAACCTGATCTTTACCAAGCAATGGTGACTGCAGCACTTCAGGATATGAGGACG
GTTGATTCCTCCAAAATAGGCTCTCAATCTCTCTTGCAATGCCAGCAAAACCAGAGCACAT
CCACTGGGTCGCCTGCTCTAGTTCAGAGGCAAATGTTACAACAGTCCCAAACACAAAATG
GGTTTCTTCCAAGTTTTCTTGAGAATCAGACTGCTTCACAGGTTCAACTTCTGCAGCAGTT
GCAGTGCCCCAATTTGTACAATGACCAGCGACAGAAGCAGCAGCAGCAGCGACAGTCTC
AAGAAACACAGCAACTGCCACCTGTTCCGCAGCAGATTTCTAATGTAATCCCTGCTTTTCC
TTCTGTGTCAGCCAACCAAGCGCAGTCATCTCTGCCAGCTGTTGATTCACAATGCCAGCA
GTCGACATTTTCCGACCATCTTGGGAACTCCATAGCTACATCTGATGTTTCTTCTATGCAAA
GTATCTTAGGTTCATTATCCCAGATGGGAGCTTCCCATTTACTCAACTTGAATGGATCAAAC
CCAATCCTCTCTTCTTCTACTTTCTTGTCCAAGCCTGCAGCTATTGAACCACAGCTTTCAT
CCAGAGTGGCCAACTCTGTACTTCCCCAGGTTGAACAGTTGGGAACTGCACAATCAAATG
CATCTGAACTTCACAACTTATTACCTCCATTTCCTGGAAGGGAGTATTCTGCTTACCACAAT
GCAACTGATCCACAGAACAATCTTCTCTTTGGGGTTAGCATTGATTCTTCGTCTCTTATGC
TGCATCATGGGATGACAAACCCGAAAAGCATCAGAAATGAAAATGATTCAATGTCCCTTCC
GTACGCTGCTTCAAATTTCACAAGTGCTTCTGGCACAGATTTTCCTCTTAATTCAGACATG
ACTGCCTCAAGTTGTGTCGATGAATCAGGTTACTTGCAGTCCTCTGAAAATGTGGACCAA
GTAAACCCTCCAACTGGAACCTTTGTCAAGGTTCACAAGTCGGGGTCCTTTGGGCGCTC
ATTGGATATTTCCAAGTTTGGCAGCTATGATGAGCTGCGCTGTGAGCTTGCTCGACTATTT
GGCCTTGAAGGCCAATTAGAGGACCCTCAGAGATCAGGCTGGCAGCTTGTATTTGTTGAC
AGGGAGAACGATATTCTTCTCCTTGGTGATGACCCATGGCAGGAATTCGTGAACAATGTG
TGGTATATCAAGATACTATCCCCACTTGAAGTACAGCAAATGGGGAAAGGTCAGAATCCTG
CTACTTCTATTCCGAACCAAAGGCTTAACGCCACCACCACCGCTACCACCAGCAGCAGCA
ATGGCAACCACTGCGGTGACTACATGAGCAGACAGGACTTGAGAAGTTCTGTTGCATCAA

TGGGGTCTCTTGAATACTAA
>GhARF6-7
ATGAGACTCTCTTCATCAGGGTTTAATCAGCAAACTGAGGAAGGGGAGAAGAAATGTTTG
AATTCTGAACTTTGGCATGCATGTGCGGGTCCTCTGGTGTCCCTGCCGCCTGTTGGAAG
CCGTGTTGTATATTTTCCCCAAGGTCATAGTGAACAGGTTGCTGCTTCAACCAACAAGGAA
GTAGATGCTCACATACCGAACTATCCGAGCTTACCCCCTCAGCTTATCTGTCAGCTTCACA
ATGTGACCATGCATGCAGATGTAGAAACAGATGAAGTGTATGCTCAAATGACCTTGCAGC
CACTGAGCCCGGATTACTCCCTGCAACCTCCAGCTCAAGAGTTAGTTGCCAGGGATCTTC
ATGAAAATGAATGGAAATTTAGGCATGTATTTCGCGGGCAGCCAAAGAGGCATCTTCTTAC
AACTGGGTGGAGTGTTTTTGTTAGTGCTAAGAGACTTGTTGCTGGTGACTCAGTCCTTTTT
ATTTGGAATGAGAAGAATCAGTTGTTGCTGGGTATCCGGAGAGCAAATCGTCCTCAGACT
GTTATGCCATCTTCTGTTTTATCAAGTGACAGCATGCACATTGGTCTTCTTGCTGCAGCCG
CTCATGCGGCTGCAACCAATAGCCGTTTTACTATCTTTTATAACCCAAGGGCCAGTCCTTC
AGAGTTTGTCATTCCTCTCGCCAAGTATGTTAAAGCAGTCTATTACACCCGGGTTTCTGTA
GGCATGCGGTTTAGAATGCTGTTTGAGACAGAAGAGTCAAGTGTCCGCCGATATATGGGT
ACGATAACTGGCATTAGTGACTTAGATCCTGGTCGCTGGCCAAATTCGCATTGGCGCTCT
GTTAAGGTTGGCTGGGATGAATCCACTGCAGGAGAGAAGCAGCCAAGGGTGTCCTTGTG
GGAGATTGAACCATTAACAACCTTCCCTATGTATTCATCACCCTTCCCCCTGCGATTGAAA
CGACCATGGCCATCTGCGCTACCCTCTTTCCATGCTTTCAAAGATGGTGATATGAACATCA
ATTCCCAAATGATGTGGCTTCAAGGTGGGATTGGAGATCAAGGACTTCAGTCTTTAAACTT
CCAAGGTTTTGGAGTTGCACCCTGGATGCAGCCAAGGCTTGATACTTCTTCAATACAAAG
TGTCCAACCTGATCTTTACCAAGCAATGGCTGCTGCTGCATTTCAGGAAATGAGGACTGT
TGATTCATCCAAAATAGGCTCTCAGTCTCTCTTGCAATTCCAGCAACCACAGAGCATGTCC
AATGGGTCGCCTGCTATAATTCAAAGGCAGATGCTACAGCAGTCTCAAACACAAAATGCTT
TTCTTCAGGGATTTCAGGAGAACCAGACTACTTTCCAGGTTCAGCTTCTGCAGCAGTTGC
AGCGTTCCAATTCATACAATAATCACCGACAACAACAACAACAACAACAACAGCAGCAGC
AGCAGCAACAACAGCAACAACAGCAACAATCTCAGCAAATGCAACAAATACCACAGTTTT
CTGATCAACAGCAGATTTCTAATCTAATCCCTGCTTTTCCTAAAGCCTCCGGCAGCCAGGC
CCAGTCATCATCTCTGCCAACTGCTGCTTCAGAATGCCAGCAGCAGACATTTTCTGATCC
TCTTGGGAACTCTCTAGCCATATCTAATGCTTTGTCAATGCAAAGTATCTTAGGTTCATTGT
CCCATGCTGGAGCTTCCCATTTACACAACTTGAAGGGATCAAACCCAATTGTCTCTTCTTC
TTTGTTGTCCAAGCCAGTAGCTATAGAACCACAGCTTTCGTCTGAAACTGCTAACTACGTA
CTGCCCCAGGTTGAACATCTGGGAATGGTACAGTCAAATGTACTCTCTAACGTATTACCTC
CATTTCCAGGAAGAGAGTATTCTGCGTACCAGAGTTCAACTGATCCACAAAACAATTTTCT
CTTTGGGGTTAGCATTGATTCATCATCTCTTGTGCTGCAGCATGGGATGACCAACCTGAAA
AATATTGGAAATGAAAATGATTCATTGTCTCTGCCATATGCCGCTTCAAATTTCACAAGTGC
TTCTGGCACAGATTTCCCTCTTAATTCAGATATGACTACCTCAAGTTGTGTGGATGAATCA
GGTTACTTGCAGTCTTCTGAATATGTGGACCAAGTAAACCCTCCGACTGGAACCTTTGTTA
AGGTTCACAAGTTGGGGTCCTTCGGGCGCTCATTGGATATTTCCAAGTTTAGCAGCTATAA
TGAGCTGCGTTGTGAGCTTGCTCGAATGTTTGGTCTGGAAGGCCAACTAGAGGACCCTC
AGAGATCAGGCTGGCAGCTTGTATTTGTTGACAGGGAGAATGATATTCTTCTCCTTGGTGA
TGACCCTTGGCAGGAATTCGTGAACAACGTGTGGTATATCAAGATACTATCTCCACTTGAA
GTGCAACAAATGGGGAAAGAAGGCCTGAATCCTGCCGCCTCTGACCCAAGCCAAACGCA
CCACCAATAG

>GhARF6-8
ATGAGACTCTCTTCATCAGGGTTTAATCAGCAAACTGAGGAAGGGGAGAAGAAATGTTTG
AATTCTGAACTTTGGCATGCATGTGCGGGTCCTCTGGTGTCCCTGCCGCCTGTTGGAAG
CCGTGTTGTATATTTTCCCCAAGGTCATAGTGAACAGCTTTTGGTGTTTATGACTCCCTCT
GCAGGTTGCTGCTTCAACCAACAAGGAAGTAGATGCTCACATACCGAACTATCCTATAAAC
AACCAACCAATTATTTTTGCAAAACACTGACGGCAAGTGACACAAGCACTCATGGAGGAT
TTTCTGTGCCTCGTCGAGCTGCTGAAAAAGTCTTTCCTCCTCTTGATTACTCCCTGCAAC
CTCCAGCTCAAGAGTTAGTTGCCAGGGATCTTCATGAAAATGAATGGAAATTTAGGCATGT
ATTTCGCGGGCAGCCAAAGAGGCATCTTCTTACAACTGGGTGGAGTGTTTTTGTTAGTGC
TAAGAGACTTGTTGCTGGTGACTCAGTCCTTTTTATTTGGAATGAGAAGAATCAGTTGTTG
CTGGGTATCCGGAGAGCAAATCGTCCTCAGACTGTTATGCCATCTTCTGTTTTATCAAGTG
ACAGCATGCACATTGGTCTTCTTGCTGCAGCCGCTCATGCGGCTGCAACCAATAGCCGTT
TTACTATCTTTTATAACCCAAGGGCCAGTCCTTCAGAGTTTGTCATTCCTCTCGCCAAGTAT
GTTAAAGCAGTCTATTACACCCGGGTTTCTGTAGGCATGCGGTTTAGAATGCTGTTTGAGA
CAGAAGAGTCAAGTGTCCGCCGATATATGGGTACAATAACTGGCATTAGCGACTTAGATCC

TGGTCGCTGGCCAAATTCGCATTGGCGCTCTGTTAAGAAGACTTACATTCCATCCCAGGT
TGGCTGGGATGAATCCACTGCAGGAGAGAAGCAGCCAAGGGTGTCCTTGTGGGAGATT
GAACCATTAACAACCTTCCCTATGTATTCATCACCGTTCCCCCTGCGATTGAAGCGACCAT
GGCCATCTGCGCTACCATCTTTCCATGCTTTCAAAGATGGTGATACAAACATCAATTCCCA
AATGATGTGGCTTCAAGGTGGGATTGGAGATCAAGGACTTCAGTCTTTAAACTTCCAAGG
TTTTGGAGTTGCACCCTGGATGCAGCCAAGGCTTGATACTTCTTCAATACAAAGTGTCCAA
CCTGATCTTTACCAAGCAATGGCTGCTGCTGCATTTCAGGAAATGAGGACTGTTGATTCAT
CCAAAATAGGCTCTCAGTCTCTCTTGCAATTCCAGCAACCACGGAGCATGACCGATGGGT
CGCCGGCTATAATTCAAAGGCAGATGCTACAGCAGTCTCAAACACAAAATGCCTTTCTTCA
GGGATTTCAGGAGAACCAGACTACTTCCCAGGTTCAGCTTCTGCAGCAGTTGCAGCGTT
CCAATTCATACAATAATCACCGACAACAGCAGCAGCAACAGCAACAACAGCAACAATCTC
AGCAAATGCAACAAACACCACAGTTTTCTGATCAACAGCAGATTTCTAATCTAATCCCTGC
TTTTCCTAAAGCCTCTGGCAGCCAGGCCCAGTCCTCATCTCTGCCAACTGCTGCTTCTGA
ATGCCAGCAGCAGACATTTTCTGATCCTCTTGGGAACTCTCTAGCCTTATCTAATGCTTCG
TCAATGCAAAGTATCTTAGGTTCATTGTCCCATGCTGGAGCTTCCCATTTACTCAACTTGAA
GGGATCAAACCCAATTATCTCTTCTTCTTTGTTGTCCAAGCCAGTAGCTATTGAACCACAG
CTTTCATCTGAAACTGCTAACTACGTACTGCCCCAGGTTGAACATCTGGGAATGGTACAGT
CAAATGTACTCTCTAACTTATTACCTCCATTTCCAGGAAGAGAGTATTCTGCTTACCAGAGT
TCAACTGATCCACAAAACAATTTTCTGTTTGGGGTTAGCATTGATTCATCATCTCTTATGCT
GCAGCAAGGGATGACCAACCTGAAAAATATTGGAAATGAAAATGATTCATTGTCTATGCCA
TATGCCGCTTCAAATTTCACCAGTGCTTCTGGCACAGATTTCCCTCTTAATTCAGATATGAC
TACCTCAAGTTGTGTGGATGAATCAGGTTACTTGCAGTCTTCTGAATATGTGGACCAAGTA
AACCCTCCGACCGGAACCTTTGTTAAGGTTCACAAGTTGGGGTCCTTTGGGCGCTCATT
GGATATTTCCAAGTTTAGCAGCTATAATGAGCTGCGTTGTGAGCTTGCTCGAATGTTTGGT
CTGGAAGGCCAACTAGAGGACCCTCAGAGATCAGGCTGGCAGCTTGTATTTGTTGACAG
GGAGAATGATATTCTTCTCCTTGGTGATGACCCTTGGCAGGAATTCGTGAACAACGTGTG
GTATATCAAGATACTATCTCCACTTGAAGTGCAACAAATGGGGAAAGAAGGCCTGAATCCT
GCCGCCTCCGACCCAAGCCAAACGCACCACCAATAG

>GhARF7-1
ATGAAGGCTCCACCAAATGGATTTATGGCAAATTCTGCAGAAGGAGAAAGGAAGAGTATC
AATTCAGAATTATGGCATGCTTGTGCTGGACCACTTGTTTCTTTGCCTCCAGTTGGAAGTT
TGGTTGTTTACTTTCCTCAAGGTCACAGCGAGCAGGTTGCGGCGTCGATGCAAAAGGAG
ACTGATTTCATACCAAGCTACCCTAACCTTCCTTCCAAGTTGATATGCATGCTCCATAATGT
CACATTGCATGCCGATCCGGAAACAGATGAGGTGTATGCCCAGATGACACTTCAACCTGT
GAACAAATATGACAAGGAAGCGTTACTGGCATCTGATATTGGCCTCAAGCAAAGCCGGCA
ACCTGCTGAGTTCTTTTGCAAGACTCTTACAGCAAGTGACACTAGCACTCATGGTGGATT
TTCTGTGCCTCGACGAGCAGCTGAGAAAATCTTCCCTCCCCTGGATTTCTCGATGCAACC
ACCTGCTCAGGAGCTAGTAGCTAAAGATTTACATGACAATACATGGACATTTAGACATATTT
ATCGAGGTCAACCAAAGAGGCATCTTCTGACTACTGGTTGGAGTGTCTTTGTTAGCACAA
AAAGACTCTTTGCCGGTGATTCTGTTCTTTTCATAAGGGATGAGAAGTCTCAACTTCTGTT
GGGTACAAGGCCTGCAAATAGACAGCAGCCAGCCCTTTCTTCATCTGTGATTTCTAGTGAT
AGCATGCATATAGGGATCCTCGCTGCTGCAGCGCATGCTGCAGCAAATAACAGCCCATTT
ACTATATTCTATAATCCAAGGGCAAGCCCTTCTGAGTTTGTGATACCCTTGGCAAAGTTTAA
CAAAGCCATGTATACCCAAGTTTCCCTTGGCATGCGGTTTAGAATGATGTTTGAAACCGAG
GAGTCTGGAGTACGCAGGTACATGGGTACAATTACTGGTATCAGTGACTTGGATCCTGTG
CGATGGAAAAATTCACAGTGGCGCAATCTTCAGGTTGGATGGGATGAATCTACAGCTGGT
GAGCGGCCCAGCCGAGTTTCAATTTGGGACATTGAACCTGTTATAACTCCTTTTTACATCT
GTCCACCTCCGTTTTTCAGGCCCAGGTTTCCAAAGCAACCGGGGATGCCAGATGATGAG
TCTGACATTGAGAATGCCTTCAAGAGAGCTATGCCCTGGCTAGGAGATGACTTTGGTATG
AAAGATGCTCCTAGTTCAATCTTTCCTGGTTTGAGTCTAGTCCAGTGGATGAGCATGCAAC
AAAATAATCAGTTTCCAGCTGCTCAATCAGGATTCTTTCCATCAATGCTCCTGCGCTTCCA
GCATCAAATATGCAATTTAACAAAGCAAATACGAACCAAATCAATCAGTTGA

>GhARF7-2
ATGAAGGCTCCACCAAATGGATTTATGGCAAATTCTGCAGAAGGAGAAAGGAAGAGTATC
AATTCAGAATTATGGCATGCTTGTGCTGGACCACTTGTTTCTTTGCCTCCAGTTGGAAGTT
TGGTTGTTTACTTTCCTCAAGGTCACAGCGAGCAAGTTGCGGCGTCGATGCAAAAGGAG
ACTGATTTCATACCAAGCTACCCTAACCTTCCTTCCAAGTTGATATGCATGCTCCATAATGT
CACATTGCATGCCGATCCAGAAACAGATGAGGTGTATGCCCAGATGACACTTCAACCTGT

GAACAAATATGACAAGGAAGCGTTACTGGCATCTGATATGGGCCTCAAGCAAAGCCGGCA
ACCTGCTGAGTTCTTTTGCAAGACTCTTACAGCAAGTGACACTAGCACTCATGGTGGATT
TTCTGTGCCTCGACGAGCAGCTGAGAAAATCTTCCCTCCCCTGGATTTCTCGATGCAACC
ACCTGCTCAGGAGCTAGTAGCTAAAGATTTACATGACAATACATGGACATTTAGACATATTT
ATCGAGGTCAACCGAAGAGGCACCTTCTGACTACTGGTTGGAGTGTCTTTGTTAGCACAA
AAAGGCTCTTTGCCGGTGATTCTGTTCTTTTCATAAGGGATGAGAAGTCTCAACTTCTGTT
GGGTACAAGGCGTGCAAATAGACAGCAGCCAGCTCTTTCTTCATCTGTGATTTCTAGTGAT
AGCATGCATATAGGGATCCTGGCTGCTGCAGCGCATGCTGCAGCAAATAACAGCCCATTT
ACTATATTCTATAATCCAAGGGCAAGCCCTTCTGAGTTTGTGATACCCTTGGCAAAGTATAA
CAAAGCCATGTATACCCAAGTTTCCCTTGGCATGCGGTTTAGAATGATGTTTGAAACCGAG
GAGTCTGGAGTACGCAGGTACATGGGTACAATTACTGGTATCAGTGACTTGGATCCTGTG
CGATGGAAAAATTCACAGTGGCGCAATCTTCAGGTTGGATGGGATGAATCTACAGCTGGT
GAGCGGCCCAGCCGAGTTTCAATTTGGGACATTGAGCCTGTTATAACTCCTTTCTACATCT
GTCCACCTCCGTTTTTCAGGCCCAGGTTTCCAAAGCAACCGGGGATGCCAGATGATGAG
TCTGATATTGAGAATGCCTTCAAGAGAGCTATGCCCTGGCTAGGAGATGACTTTGGTATGA
AAGATGCTCCTAGTTCAATCTTTCCTGTTTCCAGCTGCTCAATCAGGATTCTTTCCATCAAT
GGTTTCTTCGAATCCGCTCCATAA
>GhARF8-1
ATGAAGCTTTCAGCATCAGGCCAGGGTCAGCAGGCTCATGAAGGGGAAAACAAGTGCTT
GAACTTAGAGCTATGGCATGCCTGCGCTGGCCCACTGGTTTGTCTACCAACTGTGGGGA
CCCGTGTGGTCTACTTTCCTCAGGGTCATAGCGAGCAGGTAGCAGCCACCACTAACAAG
GAGGTTGACACTCACATTCCGAATTACCCGGACTTGCCTCCTCAGTTGATATGTCAGCTC
CAAAATGTTACCATGCACGCTGATGTTGAGACAGATGAAGTATATGCCCAAATGTCGTTGC
AGCCTTTGACGCCTGAAGAGCAGAAGGGTACATTTCTTCCTATGGAGTTTGGAATTCCAA
GCAAGCAGCCAACCAATTATTTCTACAAGACACTGACAGCAAGTGACACTAGTACCCATG
GCGGTTTTTCTGTTCCTCGTCGTGCTGCTGAGAAAGTCTTTCCGCCACTGGACTTCTCAC
AGCAGCCTCCAGCTCAGGAACTTATTGCAAGGGATCTTCATGACATTGAGTGGAAATTTA
GACATATATTTAGAGGACAGCCTAAACGCCACCTTCTTACTACAGGCTGGAGTGTGTTTGT
TAGTGCCAAGAGACTTGTTGCAGGGGATTCTGTTCTTTTTATTTGGAATGAAAAGAACCAG
CTTCTTTTGGGAATTCGCCGTGCCACTCGTCCACAAACTGTAATGCCATCATCTGTTTTAT
CTAGTGATAGCATGCACATTGGACTACTTGCTGCTGCAGCTCATGCTGCGGCAACTAATA
GCTGCTTTACAGTATTTTATAATCCAAGGGCTAGCCCATCTGATTTTATAATACCCCTTTCAA
AATATGTCAAAGCTGTTTTCCACACACGTGTCTCAGTTGGGATGCGTTTTCGGATGCTTTT
TGAGACAGAAGAATCAAGTGTTCGTAGGTATATGGGTACAATAACTGGTATAAGTAGTTTG
GATCCTGTTTGTTGGCCAAATTCCCATTGGCGGTCTGTGAAGGTTGGTTGGGATGAGTCA
GCAACAGGTGAGAGGCAGCCAAGGGTATCATTGTGGGAGATTGAGCCTTTAACAACATTT
CCAATGTATCCATCTCTATTTCCCCTCAGATTGAAACGCCCTTGGCATCCGGGTTCCTCAT
CTTTGTTTGATAACAGAGATGATATATCTAATGGGTTAACGTGGCTAAGGGGAGGATCTGG
AGAGCAAGGACTACAATCTCTGAATCTTCAGTCCTTCTGCTCATATCCATGGATGCAGCAG
AAACTAGATTTGAGTTTCCCTGGAAATGATTATAATCTGCAGTACCAACATATGCTGGCCAA
TGGGTTGCAGAACTTGGGAAGCGGGGATCCGCCGAGACAGCAGTTGCAGCAATCTTTTC
AATATGTTCAACAGCCAGGCAGCCATAATTTACTGTTGCAGCAGCAGCAGCAGCAGCAGC
AGCAGTCTGTTTCACAGTTAGTTCCACACAACATTGTGCAGGCACAGCAATCCATAATTCG
AATCGAGAGCTTTCTCCCAGTCCCTGGACGGGAACAAGTAGGCAATCAATCAGAGGAAC
AGGCTCAGCCGCAGCATAACATGACTCAAAGTGATCAGCTCCAGCAGAGGCAGCAGTTG
AATGTGCCTTCATCATTTCTGAAACCAGATTTTATTGACTCAGGATCTGGTCCCCCTGTTC
TGGATATGCTGGGTTCCTTGTGTCCTGAAAGCAGTGCAAATCTTTTGAACTTCTCCACAAC
TGGTCAGTCAATGCTAGCTGACCAGTCACCCCAACTGTCCTGGGCTCCGAAGTATGCTCA
TTTGAATGTAAATACCTTTGCTGGTTCAACATCAGTTCCACAAGTTTCTCCTGGAAAAGAT
GCTACGGTGGAACTAGATATTGGTACCTCTGATGCCCAGAATTCTACTTTTTTTGGTGTTAA
CGACGATTCATTTGGCCTTCTACTGCCCACCTCAATGCGTGGTTTCACTACATCTTCAAGT
GAAGCTGATATGCCCTCAATTCCATTAGGGGATCCCTCATTCCAGAATCCTTTGTATGGTT
GCATGCAATACTCTTCAGAGTTGCAGAGCACAGGGCAAGTGGACCCACCAACTTCATCTC
GAACATTTATCAAGGTTTATAAATCAGGGTCGGTGGGGCGCTCATTGGACATCTCCCGGT
TCAGAAGCTACAATGAGCTGAGAGAGGAGCTGGCTCAGATGTTTGGAATTGAGGGGAAG
TTGGAAGACCCTCTTAGATCAGGCTGGCAGCTTGTATTTGTCGACAGGGAGAATGATATT
CTTCTCCTTGGAGACGACCCATGGGAGGCATTTGTGAATAATGTTTGGTACATAAAGATAC
TTTCACCAGAGGATGTGCAGAAGATGGGATAG

>GhARF10-1

ATGAGGAAAGAGGCAGAAAAGAGCTTGGATCCTCAGCTATGGCATGCCTGTGCTGGATC
CATGGTTCAAATTCCACCAGTGAACTCCAAAGTCTTCTACTTTCCTCAAGGCCATGCCGA
ACACTCTTTATCTCCGGTCGATTTTTCATCTTCTCCTCCAATCCCGGCTCTAGTTCTTTGCC
GGGTGGCTTCTGTCAAATTCTTGGCTGATGCTGAAACCGATGAAGTGTATGCCAAGATCA
TGCTTGTACCATTGCCCAACCCTGAGCCTGATCTCGAGAACGTTGCCGTTTTGGGTGGT
GGGTCTGATAATGTGGAAAAGCCTGCTTCTTTTGCTAAGACATTGACTCAATCCGATGCAA
ACAACGGGGGTGGTTTTTCGGTTCCCAGATATTGTGCTGAAACCATATTTCCGAGACTGG
ATTACACTGCCGATCCTCCGGTTCAAACTGTGATTGCCAGGGATGTTCATGGTGAGATTT
GGAAGTTTAGGCATATCTATAGGGGAACACCTAGGAGACATTTATTGACTACCGGCTGGAG
TAGTTTCGTTAACCATAAGAAACTTGTTGCTGGGGACTCCATTGTGTTCTTGAGAGCCGAA
AACGGTGAGCTTTGTGTTGGGATAAGGCGGGCCAAGCGTGGGAATGATACTGGAGCTGA
ATCTGGTCTTGGAAATGGAAACTACGTTAGTCCTTATGGAGGGTTTTCAGGTTTCTTGAAG
GAGGATGAGAGTAAGATAACCCGAAAAGGGAATCCGAGAGGGAAGGGAAAAGTGAGAG
CAGAGGCTGTTGTGGAGGCAGTGGGGCTTGCCGCTAATGGCCGGCCTTTTGAGATAGTT
TACTATCCGAGAGCAAGCACACCGGAGTTTTGTGTTAAGGCATCTGCAGTAAGGGCGGC
AATGAGGGTTCCTTGGTGTTCTTTGATGAGGTTCAAGATGGCTTTCGAGACCGAGGATTG
TTCTAGGATTAGTTGGTTCATGGGGACTGTATCTTCTGTTCATATTGCAGACCCCCTTCGT
TGGCCTAATTCCCCGTGGCGCCTTCTTCAGGTAACATGGGATGAACCAGATTTGCTGCAA
AATGTTGAACGCGTGAGTCCCTGGTTGGTTGAATTGGTACCGAACATGCTTCCCGTCCAC
CTGTCACCCTTCTCAACAGTGACCCCCAGAAAGAAGTTGAGGCTTCCTGAGCACCTCGA
TTTCCCCCTTGTTGAACAATTTCCGATGCCATCGTTTTCCGGTCATCCCCTCAGGTCAAG
CAACCCATTATGTTGTTTATCTGATAATGCTCCTGCAGGCATACAGGGAGCCAGGCATGCT
CAAGTCAGATTATCTTCATCAGATCCCCATCTTAATAAACTGAAGTCCGGACTGTTTCCGT
CTGGCTTCCAGCTGTTTGATCCGCAAGCTCGAGTTCCTAATGGCATCTCAATGACAAAGC
ACACAGACAGTAACGATGATAATCTATCTTGCTTATTAACAGTAGGGAATTCCAGTCCAAAG
AAGAAATCTGAAAACGGAAAGAGACATCAGTTTTTACTCTTTGGTCAGCCAATACTTACCG
AGCAGCAGCTCTCTCGTAGCTGTTCAACTGGAGTAAAAACTGCTCTCGAGAATGAAGACA
AAAGAAAAGATTATTCCAATGGTTCAGAATCTGCTCTCGAGAATCAATTGTCTCCAGAGAA
GTCATTTACTACCAGATTATTGTGGCAGCAGGACTATCAAGCCCCAGAACCTGGCTCAGC
CACTGGTCATTGCAAGGTATTCTTGGAATCTGAGGATGTGGGACGAACTCTTGACCTCAC
AGTTCTCGGTTCTTATGAAGAGCTATACATGAGGTTGGCTAACATGTTTGGAAGAGAAAGA
TCAGAGATGTTGGGCCATGTCTTGTATCGAGATGCAACAGGTGCTGTCAAACAAACTGGA
GATGAACCATTCAGTACTTTTATGAAGACGGTCAAAAGATTGAACATAAGGATGGATTCAC
GCAATGATACCGATGGAAGGTCATGGCTCACAGGGATTCGAACTGCCAAAAACAGACTAG
AAGGGCCAAATAAGAGAGGTCCCTTGAGCATATTTGCATGA

>GhARF10-2
ATGAGGAAAGAGGCAGAAAAGAGCTTGGATCCTCAGCTATGGCATGCCTGTGCTGGATC
CATGGTTCAAATTCCACCAGTGAACTCCAAAGTCTTCTACTTTCCTCAAGGCCATGCCGA
ACACTCTTTATATCCGGTCGATTTTTCATCTTCTCCTCCAATCCCGGCTCTACTTCTTTGCC
GGGTGGCTTCTGTCAAATTCTTGGCTGATGCTGAAACCGATGAAGTGTATGCCAAGATCA
TGCTTGTACCATTGCCCAACACTGAGCCTGATCTCGAGAACGATGCCGTTTTCGGTGGTG
GGTCTGATAATGTGGAAAAGCCTGCTTCTTTTGCTAAGACATTGACTCAATCCGATGCAAA
CAACGGAGGTGGTTTTTCGGTTCCCAGATATTGTGCTGAAACCATATTTCCGAGACTGGA
TTACACTGCCGATCCTCCGGTTCAAACTGTGATTGCCAGGGATGTTCATGGTGAGATTTG
GAAGTTTAGGCATATCTATAGGGGAACCCCTAGGAGACATTTATTGACTACTGGCTGGAGT
AGTTTCGTTAACCATAAGAAACTTGTTGCTGGGGACTCCATTGTGTTCTTGAGAGCCGAA
AACGGTGAGCTTTGTGTTGGGATAAGGCGGGCCAAGCGTGGGAATGATACTGGAGCTGA
ATCTGGTCTTGGAAATGGAAACGACGTTAGTCCTTATGGAGGGTTTTCAGGTTTCTTGAA
GGAGGATGAGAGTAAGATAACCCGAAAACGGAGTCCGAGAGGGAAGGGAAAAGTGAGA
GCAGAGGCTGTTGTGGAGGCAGTGGCGCTTGCCGCTAATGGCCAGCCTTTTGAGATAGT
TTACTATCCGAGAGCAAGCACACCGGAGTTTTGTGTTAAGGCATCTGCAGTAAGGGCGGC
AATGAGGGTTCCTTGGTGTTCTTTGATGAGGTTCAAGATGGCTTTCGAGACCGAGGATTG
TTCTAGGATTAGTTGGTTCATGGGGACTGTATCTTCTGTTCATATTGCAGACCCCCTTCGT
TGGCCTAATTCCCCGTGGCGCCTTCTTCAGGTAACATGGGATGAACCAGATTTGCTGCAA
AATGTTGAACGCGTGAGTCCCTGGTTGGTTGAATTGGTACCGAACATGCTTCCCGTCCAC
CTGTCACCCTTCTCAACAGTGACCCCCAGAAAGAAGTTGAGGCTTCCTGAGCACCTCGA
TTTCCCCCTTGTCGAACAATTTCCGATGCCACCGTTTTCTGGTCATCCCCTCAGGTCAAG
CAACCCCTTACGTTGTTTATCTGATAATGCTCCTGCAGGCATACAGGGAGCCAGGCATGC
TCAATTCAGATTATCTTCATCAGATCCTCATCTTAATAAACTGAAGTCTGGACTGTTTCCGT

CTGGCTTCCAGCTGTTTGATCCACAACCTCGAGTTCCCAATGGCATCTCAATGACAAAGC
ACACAGACAGTAACAATGATAATCTATTGTGCTTATTAACAGTAGGGAATTCTAGTCCAAAA
AAGAAATCTGAAAACGGAAAGAGACACCAGTTTTTACTCTTTGGTCAGCCGATACTTACCG
AGCAGCAGCTTTCTCATAGCTGTTCAACTGGAGTAAAAACTTCGGTCAAGAATGAAGACA
AAAGAAAAGATTCTTCCAATGGTTCAGGATCTGCTCTTGAGAATCAATTGTCTCCAGAGAA
GTCATTTACTACCACATTATTGTGGCGGCAGGACTATCAAACCCCAGAACCTGGCTCAGC
CACTGGTCATTGCAAGGTATTCTTGGAATCTGAGGATGTGGAACGAACTCTTGACCTCAC
AGTTCTCGGTTCTTATGAAGAGCTATACAGGAGGTTGGCTAACATGTTTGGAAGAGAAAGA
TCAGAGATGTTGGGCCATGTCTTGTATCGAGATGCAACAGGTGCTGTCAAACAAACTGGA
GATGAACCATTCAGTACGTTTATGAAGACGGTCAAAAGATTGAACATAAGGATGGATTCAC
GCAATGATGCCGATGGAAGGTCATGGCTCACAGGTATTCGAACTGCCGAAAACAGACTAG
AAGGGCCAAACAAGAGAGGTCCCTTGAGCATATTTGCATGA

>GhARF11-1
ATGATGGCGAATCGGGTAGAGTCGTTTTCGCAGACAAATAATGTCTCCTCTGAAGGAAAT
GGATGCGATGATCTGTACATGGAATTATGGAAGTTATGTGCCGGACCACTGGTTGAGTTG
GAAGCATCCACGAATCAGACGTTGAATCAGAGGATTCCGTTGTTTAATCTTCCATCGAAGA
TTCTTTGCCGTGTCGTTCATATTCAGTTGCTGGCTGAAAAAGAAACAGATGAAGTTTATGC
ACAAGTGACTTTACTGCCGGAACCAAGTCAATCTGAGCCAACAACCCCTGATCTGTGCCC
CCCTGAGTCTCAAAGACCTACAGTCCACTCATTCTGCAAGGTTTTAACTGCATCAGATACA
AGCACCCATGGAGGATTTTCTGTTCTTCGCAAACATGCTACTGATTGCCTTCCTAAACTGG
ACATGAATGATGCAACGCCAACCCAGGAATTGGTTGCAAAGGATCTTCATGGTTATGAGT
GGCGCTTTAAGCATATTTTCAGAGGCCAACCAAGGAGACATTTGCTTACAACAGGATGGA
GTACGTTTGTTACTTCAAAGAGATTAGTAGCTGGTGACTCCTTTGTGTTTTTGAGAGGGGA
AAATGGTGAGCTACGTGTTGGAGTGAGGCGTGTTGTTCGCCAACATAGCATCATGCCGTC
GTCAGTGATTTCAAGTCAGAGCATGCATGTAGGAGTGCTTGCAACTGCATCTCATGCAGT
TTCAACACAAACCCTCTTTGTTGTCTACTATAAGCCAAGGACAAGTCAGTTCATCATTGGG
TTGAACAAATATTTAGAGGCTCTTAGTAATAAGTTTGCTGTTGGCATGAGATTTAAGATTAA
GTTTGAAGGGTTTTCTGGCACGATCGTGGGGGTTGAAGATTTTTCTCCTCTTTGGAAAGA
TTCAAAATGGCGATCATTGAAGGTACAATGGGATGACCCTGCCTCTATCCCAAGGCCTGAT
AGGGTTTCACCGTGGGAGATAGAACCCTTTGCTGCTCCCATTCCACCTTCTTTGACTCAA
CCAGTTGCAGCTAAGAACAAAAGGCCCCGGCCACCTGCTGAAATTCCTGCCACGGATTTA
TCTTCAACGACATCAGCTCCTTATTCTGGAGTTACACATTGCCATGATCTAACACCACAAA
ACATTGCTGCCGAAGCAAAAGGAAATGAAAATCCTGTTATGGGGCACCACATGTGGACAG
AGATGAGCAGTGGTTGCAGCTCTGTCGTGAAGACTCTGAATAAAGGAAGTTGGTTATCTT
CTCCTGGCACATGTGTTCCTCATCATCTGTTTCCTGAAGCAAGAGAAGATAAAACGAGATT
CTCTGCTTGGCCTGTTCCTTCGGGCTTTTCAAACCCACAGCTCAACGAAGATTCAACCTT
TGATTCAACTGAAAAGGCTAAGAGATCTGAGACATCTGCTAGCTGCCGATTGTTCGGAATT
GATCTGATAAACCATTCTGCAAGCTCAACACCTTTGGAGAGAACACCTGCACAACTTTCTA
CTATGATAATGGGTATAACTGAATTATCAGCAAAAGAGAACCAGAGCATGCAAAGTTGTTCT
AGTTTTACAAGAAGTCGTACAAAGGTTCAGATGCAGGGTATAGCCGTAGGTCGTGCTGTG
GATTTGGCCATGTTTGAAGGGTATGATCAGCTTATAGATGAACTGGAGGAGATGTTTGATA
TTAAGGGAGAGCTTCGCCCTAGGAATAAGTGGGAGATTGTCTACACTGATGATGAAGGCG
ATATGATGCTTGTAGGCGATGATCCATGGCCGGAATTTTGCAACATGGTAAGAAGAATATTT
ATTTGTTCAAGCCAAGATGTGAAAAAGCTGAAGACGGGAAGCAAACTTCCCTTGGCTTCT
ATCGAAGGTGAAGGGACGGTAATAAGTTCAGACTCATCTGAAAACTAA

>GhARF11-2
ATGATGGCGAATCGGGTAGAGTCGTTTTCGCAGACAAATAATGTCTCCTCTGAAGGAAAT
GGATGCGATGATCTGTACATGGAATTATGGAAGTTATGTGCCGGACCACTGGTTGAGTTG
GAAGCATCCACGAATCAGACGTTGAATCAGAGGATTCCGTTGTTTAATCTTCCATCGAAGA
TTCTTTGCCGTGTCGTTCATATTCAGTTGCTGGCTGAAAAAGAAACAGATGAAGTTTATGC
ACAAGTGACTTTGTTGCCGGAACCAAGTCAACCTGAGCCAACAACCCCTGATCTGTGCC
CCCCTGAGTCTCAAAGACCTACAGTCCACTCATTCTGCAAGGTTTTAACTGCGTCAGATA
CAAGCACTCATGGAGGATTTTCTGTTCTTCGCAAACATGCTACTGATTGCCTTCCTAAACT
GGACATGAATGATGCAACACCAACCCAGGAATTGGTTGCAAAGGATCTTCATGGTTATGA
GTGGCGCTTTAAGCATATTTTCAGAGGCCAACCAAGGAGACATTTGCTCACAACAGGATG
GAGTACATTTGTTACTTCAAAGAGATTAGTAGCTGGTGACTCCTTTGTGTTTCTGAGAGGG
GAAAATGGTGAGCTACGTGTTGGAGTGAGGCGTGTTGTTCGCCAACATAGCATCATTCCG
TCGTCAGTGATTTCAAGTCAGAGCATGCATGTAGGAGTGCTTGCAACTGCATCTCATGCT

GTTTCAACACAAACCCTCTTTGTTGTCTACTATAAGCCAAGGACAAGTCAGTTCATCATTG
GGTTGAACAAATATTTAGAGGCTCTTAGTAATAAGTTTGCTGTTGGCATGAGATTTAAGATG
AAGTTTGAAGGGGAGGATTCTCCTGAGAGAAGGTTTTCTGGCACGATTGTGGGGGTTGA
AGATTTTTCTCCTCTTTGGAAAGATTCAAAATGGCGATCATTGAAGGTACAATGGGATGAA
CCTGCCTCTATCCCAAGGCCTGATAGGGTTTCACCGTGGGAGATAGAACCCTTTGCTGCT
CCCATTCCACCATCTTTGACTCAACCGGTTGCAGCTAAGAACAAAAGGCCTCGACCACCT
GCTGAAATTCCTGCCCCAGATTTATCTTCAACGACATCAGCTCCTTATTCTGGAGTTACGC
ATTGCCATGATCTAACACCACAAAACATTGCTGCCGAAGCAAAAGGAAATGAAAATCCTGT
TATGGGGCACCACATGTGGACAGAGATGAGCAGTGGTTGCAGCTCTGTCGCAAAGGCTC
TGAATAAAGGAAGTTGGCTATCTTCTCCTGGCACATGTGTTCCTCATCATCTGTTTCCTGA
TGCAAGAGAGGATAAAACGAGATTCTCTGCTTGGCCTGTTCCTTCGGGTTTTTCAAACCC
ACAGCTCAACGAAGATTCAACCTTTGATTCAACTGAAAAGGCTAAGAGATCTGAGACAGC
TGCTAGCTGCCGATTGTTCGGTATTGATCTGATAAACCATTCTACAAGCTCAACACCGTTG
GAGAGAACACCTGCACAACTTTCTACTATGATAATGGGTACAACTGAAGTACCAGGCCCA
AGTACTCTATCATCCACTTATTCTGATCAGAAGTCTGAAATTTCAAAAGATTCTAAAGAGAA
GAAGCTGGAACAATTACAGTTATCAGCAAAAGAGAACCAGAGCATGCAAAGTTGTTCTAG
TTTTACAAGAAGTCGTACAAAGGTTCAAATGCAGGGGATAGCCGTAGGTCGTGCTGTGGA
TTTGGCCATTTTTGAAGGGTATGGTCAGCTTATAGATGAACTGGAGGAGATGTTTGATATTA
AGGGAGAGCTTCGCCCTAGGAATAAGTGGGAGATTGTCTACACTGATGATGAAGGTGATA
TGATGCTTGTAGGCGATGATCCATGGCCGGAATTTTGCAACATGGTAAGAAGAATATTTATT
TGTTCAAGCCAAGATGTGAAAAAGCTGAAGACAGGAAGCAAACTTCCCTTGGCTTCTATC
GAAGGTGAAGGGACGGTAATAAGTTCAGACTCATCTGAAAACTAA

>GhARF11-3
ATGGCGAATCGGGGAGGGGTGTTTTCACAGACAAATAATGTTTCCTCCGAAGGAAATGGA
GGGGATGATCTGTACATGGAGTTATGGAAGTTATGTGCGGGACCCCTGGTTGAGGCTCCT
CGAGCTAGAGAGAGAGTCTATTATTTTCCTCAAGGACATATGGAACAGTTGGAAGCGTCG
ACGAATCAGGAGTTGAATCAGAGGATTCCTTTGTTTAATCTTCCATCGAAGATACTTTGCA
GTGTTGTTCACATTCAGTTTCTGGCTGAACAAGAAACAGATGAAGTTTATGCACAAATAAC
TTTGATGCCGGAGCCAAATCAACCTGAGCCAACAACTCCTGATGCATGTCTTCCGGAGCC
TCCAAAACCTACTGTCCATTCTTTCTGCAAGGTTTTAACAGCCTCTGATACCAGCACTCAT
GGAGGTTTTTCTGTTCTTCGCAAACATGCCACTGAATGCCTTCCTCCACTGGACATGAAC
CAGGCAACGCCAACCCAGGAATTGGTTGCCAAGGATCTTCATGGCTATGAGTGGCGCTTT
AAGCATATTTTTAGAGGCCAGCCAAGGAGGCATTTGCTCACAACGGGATGGAGTACATTT
GTTACTTCAAAGAGATTAGTAGCTGGTGATTCCTTTGTGTTCTTGAGAGGGGAAAATGGG
GAGCTGCGCGTTGGAGTGAGGCGTGTTGCTCATCAACAGAGCAGCATGCCTACATCAGT
GATTTCAAGTCAGAGCATGCATTTAGGAGTACTAGCAACTGCATCTCATGCCGTTTCAACA
CAAACCCTCTTTGTTGTCTACTATAAACCAAGGACAAGTCAGTTCATTATTGGGTTGAACA
AATATTTAGAGGCTCTGAGCAATAAGTTTACAGTTGGCATGAGGTTTAAGATGAGATTTGAA
GGAGAGGATTCTCCTGAGAGAAGGTTTTCTGGCACAATCGTTGGGGTTGAAGACTTTTCT
CCTTACTGGAAAGATTCAAAATGGCGTTCATTGAAGGTACAATGGGATGAACCTGCCTCTA
TACCAAGACCTGACAGGGTTTCACCATGGGAGATAGAACCCTTCGCTGCTCCTATTCCAC
CAACTCTGGGTCAACCTGTTGCTGCTAAGAACAAAAGGCCTAGACCAACTGCTGAAATTC
CTGCTCTAGATTTATCTTCAACAGCATCAGCTCCTTGGAATTCTGGAGTTATGCATACCCAT
GATCTAATGCGGCGTAACATTACTGCCGAAGCAAACAGAAATGAAAATCATGTCATATGGC
ATATGCAGACTGAAATGAGCAGCAGTTGCACCTCTGTCTTGAAGACTCAGAACGAAGGGA
GCTGGCTATCTTCTCCTTGCATGAGTGTTTCTAAGCATCGATTTCCTGATGCAAGAGATGA
TAGCAAATGTGCATCTGGTTGGCCTGTTCTTTCAGGATTGTCAAACCCACAAGTGAACAAT
GATTCAACCTTTGATCCAAAAGAAAAGGTAAAGAAATCTGAGACAGCTTCTAGCTGCCGAT
TGTTTGGCATTGAGTTGATAAACCACTCTGCTAGCTCAAGGCAATTGGAGAGGACACCTA
CCCAACTTTCTACTATGACTGCCAGTACAGCTGAAGGACATCATACCTTGTCACCTAATAAT
TCTTGCCAGAAGTCTGAAATTTCAAAAGATTCTAATGAGAAGCAGGAACAGTTACAATTAC
AAGCAAAAGAGATCCAGAGCAGGCAAAGTTGCCCTAGTTCTACAAGAAGTCGTACCAAG
GTCCAGATGCAGGGGGTAGCTGTCGGTCGCGCTGTGGACCTGGCCATGTTGGAAGGGT
ACGATCAGCTTATAGATGAACTAGAGGAGATGTTTGATATTAAGGGAGAGCTTCGCCCTAG
GAATAAGTGGGAGATTGTCTACACTGATGATGAAGGGGATATGATGCTTGTAGGCGATGA
CCCATGGCAGGAATTCTGTAGCATGGTAAGAAGAATATTTATCTGTTTAAGTCAGGATGTG
AAAAAACTGAGTACAGGAAGCAAACTTCCCATGGCTTGTATTGAAGGTGGTGAAGGAACT
GTGATAAGCTCAGAGTCAATTGAAAATTAA

>GhARF11-4
ATGTCGAATCGGGGAGGGGTGTTTTCACAGACAAATAATGTTTCCTCCGAAGGAAATGGA
GGAGATGATCTATACATGGAGTTATGGAAGTTATGTGCGGGACCCCTGGTTGAGGCTCCT
CGAGCTAGAGAGAGAGTCTATTATTTTCCTCAAGGACATATGGAACAGTTGGAAGCGTCG
ACGAATCAGGAGTTGAATCAGAGGATTCCTTTGTTTAATCTTCCATCGAAGATACTTTGCA
GTGTTGTTCACATTCAGTTTCTGGCTGAACAAGAAACAGATGAAGTTTATGCACAAATAAC
TTTGATGCCGGAGCCAAATCAACCTGAGCCAACAACTCCTGATGCATGTCCTCCGGAGC
CTCCAAAACCTACTGTCTGTTCTTTCTGCAAGGTTTTAACAGCCTCTGATACCAGCACTCA
TGGAGGTTTTTCTGTTCTTCGCAAACATGCCACTGAATGCCTTCCTCCACTGGACATGAA
CCAGGCAACGCCAACCCAGGAATTGGTTGCCAAGGATCTTCATGGCTATGAGTGGCGCT
TTAAGCATATTTTTAGAGGCCAGCCAAGGAGGCATTTGCTCACAACGGGATGGAGTACATT
TGTTACTTCCAAGAGATTAGTAGCTGGTGATTCCTTTGTGTTCTTGAGAGGGGAAAGTGG
GGAGCTGCGCGTTGGAGTGAGGCGTGTTGCTCGTCAACAGAGCAGCATGCCTACATCA
GTGATTTCAAGCCAGAGCATGCATTTAGGAGTACTAGCAACTGCATCTCATGCCGTTTCAA
CACAAACCCTCTTTGTTGTCTACTATAAACCAAGGACAAGTCAGTTCATTATTGGGTTGAA
CAAATATTTAGAGGCTCTTAACAATAAGTTTACAGTTGGCATGAGGTTTAAGATGAGATTTG
AAGGGGAGGATTCTCCTGAGAGAAGGTTTTCTGGCACAATCGTTGGGGTTGAAGACTTTT
CTCCTTACTGGAAAGATTCAAAATGGCGTTCATTGAAGGTACAATGGGATGAACCTGCCT
CTATACCAAGACCTGACAGGGTTTCACCATGGGAGATAGAACCCTTCGCTGCTCCTATTC
CACCAACTCTGGGTCAACCTGTTGCTGCTAAGAACAAAAGGCCTAGACCAACTGCTGAAA
TTCCTGCTCTAGAATTACCTTCAACAGCATCAGCTCCTTGGAATTCTGGAGTTATGCATAC
CCATGATCTAATGCGGCGTAACATTACTGCCGAAGCAAACAGAAATGAAAATCATATCATAT
GGCATATGCAGACTGAAATGAGAAGCAGTTGCAGCTCTGTCTTGAAGACTCAGAACGAA
GGGAGCTGGCTATCTTCTCCTTGCATGAGTGTTTCTAAGCATCGATTTCCTGATGCAAGA
GACGATAGCAAATGTGCATCTGGTTGGCCTGTTCTTTCAGGATTGTCAAACCCACAAGTG
AACAATGATTCAACCTTTGATCCAATAGAAAAGGTAAAGAAATCTGAGACAGCTTCTAGCT
GCCGATTGTTTGGCATTGAGTTGATAAACCACTCTGCTAGCTCAAGGCAATTGGAGAGGA
CACCTACCCAACTTTCTACTATGACTGCCAGTACAGCTGAAGGACATCATACCTTGTCACC
TAATAATTCTTGCCAGAAGTCTGAAATTTCAAAAGATTCTAATGAGAAGCAGGAACAGTTAC
AATTACAAGCAAAAGAGATCCAGAGCAGGCAAAGTTGCCCTAGTTCTACAAGAAGTCGTA
CCAAGGTCCAGATGCAGGGGGTAGCCGTCGGTCGCGCTGTGGACCTGGCCATGTTGGA
AGGGTACGATCAGCTTATAGATGAACTAGAGGAGATGTTTGATATTAAGGGAGAGCTTCGC
CCTAGGAATAAGTGGGAGATTGTCTACACTGATGATGAAGGGGATATGATGCTTGTAGGC
GATGACCCATGGCAGGAATTCTGTAGCATGGTAAGAAGAATATTTATCTGTTTAAGTCAGG
ATGTGAAAAAGCTGAGTACAGGAAGCAAACTTCCCATGGCTTGTATTGAAGGTGGTGAAG
GAACTGTGATAAGCTCAGAGTCAATTGAAAATTAA

>GhARF16-1
ATGATTACTTTTATGGAGTCGAAAGAGAAACCGAAAGAGATGGAGAAATGCTTGGATTCTC
AGCTATGGCATGCTTGTGCTGGTGGAATGGTTCAAATGCCTTCAGTTAACACCAAAGTCTT
TTACTTTCCTCAAGGCCATTCCGAGCACGCTTGTGGCACTGTTGATTTCAGGAACTGTCC
TCGAATACAAGCTTATATACTCTGCAGAGTCGCCGCCGTTAAGTTCATGGCCGATCCTGAA
ACCGACGAGGTTTTCGCCAAAATCAGGCTGATCCCAGTTAACACCAATGACCCTGATTTT
GAAGATGATGGAATAGGAAGCATTAATGGGAATGAAACACAAGAAAAACCGGCTTCCTTT
GCAAAGACATTGACTCAATCAGATGCTAACAATGGTGGGGGATTTTCAGTTCCAAGGTAC
TGTGCTGAAACGATATTTCCCAGATTGGATTACTCTGCTGATCCCCCGGTTCAGACCATTC
TGGCTAAGGATGTCCATGGGGAAACTTGGAAGTTTAGGCATATTTACAGGGGTACCCCAA
GGAGACATCTTTTGACTACGGGCTGGAGTACTTTTGTCAACCATAAGAAGCTTGTAGCTG
GTGACTCGATTGTGTTTCTTAGGGCGGAAAATGGGGATCTCTGCATCGGAATTAGGAGGG
CGAAGAGGGGGATTGGTGGAGGACCCGAGTCTTCAACCGGATGGAATGCTACTGCTGG
AAATTGTATGATCCCGTATGGGGGGTTTTCAGCATTTCTGAGGGAAGATGAGGGCAAGCT
GATGAGAAATGGAAGCAGTAATGGGGTTAGCTCAAATGGTAATTTGATGGGGAAGAGGAA
GGTTAGGCCTGAACAAGTTATTGAAGCTGCAACACTTGCTTCCAATGGCCAACAATTCGA
GGTCGTTTACTACCCAAGGGCGAGTACTCCTGAGTTCTGCGTGAAGGCCTCTTTAGTGAA
GGCTGCATTGCAGATCCGGTGGTGCTCAGGAATGAGGTTCAAAATGGCATTCGAAACAG
AGGATTCTTCTCGGATTAGTTGGTTTATGGGCACTATATCTTCAGTTCAAGTCGCAGATCC
CCTCCACTGGCCTGACTCACCTTGGAGGCTTCTTCAGGTTACATGGGACGAGCCAGATT
TGCTTCAGAATGTCAAACGAGTTAGTCCTTGGCTGGTGGAATTGGTTTCGAACATGCCCG
CGATTCATCTATCTCCCTTTTCACCACCAAGGAAAAAGTTGAGACTGCCGCAGCACTCGG
ATTTTCACCTAGATGGTCAACTTCCAATGCCGACATTTTCAGGCAACCTCCTTGGGGCCA

GCAACCCCTTTGGGTGTTTACCCAACACTGCTCCTGCTGGCATGCAGGGAGCCAGGCAT
GCTCATTACGGTCTATCTTTATCAGATCTCCACCTCAATAAACTGCAGTCAGGTCTCTTTCC
GGCTAGTTTCCCACCACTTGATTACGCTCCTGTACCTAATAGGACCTCCAATGGTGGCCC
AAACATTCGAAAGCCTAGCATGAGTGAGAACGTTTCTTGTGTGCTAACCATGTCCCATCC
CTCACAGAATACCAAGAAAGCTGATGGTGCAAAGACACCTCAGCTTGTACTTTTCGGTCG
GCCGATTCTTACTGAACAGCAAATCTCTCTCAGCTGCTCTGGTGATACAATCTCACCAGTT
CTTACTGGAAATAGTTCTTCAGAATATCTGGATAAGGCAGCAAACTTTTCCGATGGTTCTG
GATCTGCTCTTCATCAACAAGGCCTTCCTGAGCGAGCATCCTGCAAAGGTCTCCCGTGGT
ACAAAAACAATCGCCAAGAAACTGAACCCAATTTAGAGACCGGTCACTGTAAAGTTTTCAT
GGAATCAGAGGATGTCGGTCGTACACTCGACCTTTCTTTGCTGGGGTCTTATGATGAACT
GCAGAGAAAGTTGGCAGACATGTTTGGTATAGAAAACTCCGAAACTCTGAGCCATTTACT
CTATCGGGATGCTACGGGTGCTGTCAAACAAATTGGAGAAGAACCATTCAGTGACTTTAT
GAAAACCGCGAGGAGATTAACGATTCTAACGGATTCAAGCAGCGACAACGTAGGAGAATA
TAGAGGAAGAAGCAAACCTTGA

>GhARF16-2
ATGATTACTTTTATGGAGTCGAAAGAGAAACCGAAAGAGATGGAGAAATGCTTGGATTCTC
AGCTATGGCATGCTTGTGCTGGCGGAATGGTTCAAATGCCTTCAGTTAACACCAAAGTCT
TTTACTTTCCTCAAGGCCATTCCGAGCACGCTTGTGGCACTGTTGATTTCAGGAACTGTC
CTCGAATACAAGCTTATATACTCTGCAGAGTCGCCGCCGTTAAGTTCATGGCCGACCCTG
AAACGGACGAGGTTTTCGCCAAAATCAGGTTGATCCCAGTTAACACCAATGACCCTGATT
TTGAAGATGATGGAATTGGAAGCATTAATGGGAATGAAACACAAGAAAAACCGGCTTCCTT
TGCAAAGACATTGACTCAATCAGATGCTAACAATGGTGGGGGATTTTCAGTTCCAAGGTA
CTGTGCTGAAACGATATTTCCAAGATTGGATTATTCTGCTGATCCCCCGGTTCAGACCATT
CTGGCTAAGGATGTCCATGGGGAAACCTGGAAGTTTAGGCATATTTACAGGGGTACCCCA
AGGAGACATCTTTTGACTACGGGCTGGAGTACGTTTGTCAACCATAAGAAGCTTGTAGCT
GGTGACTCGATTGTGTTTCTTAGGGCGGAAAATGGGGATCTCTGCATCGGAATTAGGAGG
GCGAAGAGGGGGATTGGTGGAGGACCCGAGTCTTCAACCGGATGGAATGCTACTGCTG
GAAATTGTATGATCCCGTATGGGGGGTTTTCAGCATTTCTGAGGGAAGAGGAGGGTAAGC
TGATGAGAAATGGAAGCGGTAATGGGGTTAGCTCAAATGGTAATTTGATGGGGAAGAGGA
AGGTTAGGCCTGAACAAGTTATTGAAGCTGCAACACTTGCTTCCAATGGCCAACAATTCG
AGGTCGTTTACTACCCAAGGGCGAGTACTCCTGAGTTCTGCGTGAAGGCCTCTTTAGTGA
AGGCTGCATTGCAGATCCGGTGGTGCTCAGGAATGAGGTTCAAAATGGCATTCGAAACA
GAGGATTCTTCTCGGATTAGTTGGTTTATGGGCACTATATCTTCAGTTCAAGTCGCGGATC
CCCTCCACTGGCCTGACTCACCTTGGAGGCTTCTTCAGGTTACATGGGACGAGCCAGAT
TTGCTTCAGAATGTAAAACGAGTTAGTCCTTGGCTGGTGGAATTGGTTTCGAACATGCCC
GCGATTCATCTATCTCCCTTTTCACCACCAAGGAAAAAGTTGAGACTGCCGCAGCACTCG
GATTTTCACCTAGATGGTCAACTTCCAATGCCAACATTTTCAGGCAACCTCCTTGGGGCC
AGCAACCCCTTTGGGTGTTTACCCAACACTGCTCCTGCTGGCATGCAGGGAGCCAGGCA
TGCTCATTACGGTCTATCTTTATCAGATCTCCACCTCAATAAACTGCAGTCAGGTCTCTTTC
CGGCTAGTTTCCCACCACTTGATTACGCTCCTGTACCTAATAGGACCTCCAATGGTGGCC
CAAACATTCAAAAGCCTAGCATGAGTGAGAACGTTTCTTGTGTGCTAACCATGTCCCATCC
CTCACAGAATTCCAAGAAAGCTGATGGTGCAAAGACACCTCAGCTTGTACTTTTCGGTCG
GCCTATTCTTACTGAGCAGCAGATCTCTCTCAGCTGCTCTGGTGATACAAGCTCACCAGT
TCTTACTGGAAATAGTTCTTCAGAATATCTGGATAAGGCAGCAAACTTTTCCGATGGTTCT
GGATCTGCTCTTCATCAACAAGGCCTTCCTGAGCGAGCATCCTGCGAAGGTCTCCCGTG
GTACAAAAACAATCGCCAAGAAGCTGAACCCAATTTAGAGACTGGTCACTGTAAAGTTTTC
ATGCAATCAGAGGATGTCGGTCGTACGCTCAACCTTTCTTTGCTGGGGTCTTATGATGAA
CTGCGGAGAAAGTTGGCAGACATGTTTGGTATAGAAAACTCCGAAACTCTGAGCCATTTA
CTCTATCGGGATGCTACGGGTGCTGTCAAACAAATCGGAGAAGAACCATTCAGTGACTTT
ATGAAAACCGCGAGGAGATTAACGATTCTAACGGATTCAAGCAGCGACAACGTAGGAGAA
TATAGAGGAAGAAGCAAATCTTGA

>GhARF17-1
ATGCCGCCTTCACGGTCTAGTCCTCCGGAGCTACGTCATGTTGATCCAAGGATCTGGCGA
GCTTGTGCCGGCTCTTCCGTTCAGATCCCCACCGTTAATTCTAGGGTTTATTACTTCCCTC
AAGGCCACCTTGAACAAGTTTGCGGTTCCACTCCCAAACTGTCCTCTCTCGTACTGTCTA
GGCCTCTCATCAATTGCGTTATCTCAGACGTTCATTATCTTGCCGATCCGAGAACCGATGA
GGTCTTCGCTAAGCTTTTACTCACCCCTGTTGAAACTTCTAGGCTTCCTAATCAATTTCTAA
ATGTGAATGGTGAAGTTGAGGATTCTGATAGGAATAAGATCGTGTCGTTTGCTAAGGTTTT

AACACCCTCTGATGCCAACAACGGCGGTGGATTCTCCGTCCCGCGGTTTTGTGCCGATT
CTGTTTTCCCGCCGCTCGACTACAATGCTGATCCGCCAGTTCAGACTCTCTCCGTCACCG
ACGTTCGCGGCGGTGTTTGGGAGTTTCGTCACATTTATCGAGGGACGCCACGTAGGCAT
CTGCTCACTACGGGATGGAGCAAGTTCGTTAATCAAAAGAAGCTTATCGCCGGCGATTCT
GTTGTTTTCATGAGGGACTGTAATTGGAAAATGTTTATTGGAGTCCGACGAGCGATGAAG
GCGGGAGAAGGCGGAGGGGATTCTGGGAGGTGGAGAGAGCCGAGTGATGGAGGAGCA
ATGAAAGGGGAAGGAAGGGGGAGGATGACGGCGGAGGCAGTGGCTGAAGCGGCGGAG
AAGGCAGCGAGGGGGTTCCCGTTCGAGGTTGTGTATTATCCTCGGGCCGGTTGGACTGA
TTTCGTGGCAAGGGCGGTGTTGGTGGAGGCTGGAATCAGTACTTACTGGGCTGCGGGG
ACCAGAGTGAAGATGGCAGTTGAGACGGAGGACTCCTCGCGATTGGCTTGGTTTCAAGG
GACAGTTATGTCTGCTGCTGTACCGGATTCTGGCCCCTGGATTGGCTCGCCTTGGCGAAT
GCTTCAGGTTGCTTGGGATGAACCTGAAGTTCTCCAGAATGCAAGGAAAGTGAACCCAT
GGCAAGTTCAAATTTCTTCCTCCTCACCGCTTCATTCCTCGTTCCCCTCGGAAAAGAGGC
TGAAGTTTTCTCAGGATTCAGGGCTGGCTGATGCAGAGGGTGAAATCTTTCCTATATCAG
GGTTAACTAATTCAACAATGGGGTATTTGAATCCATCACTGTTGAATTACAATTCTTTTCCT
GCTGGCATGCAGGGAGCCAGGCAATATCATTTTCACTTGCAAAGTTTAACCAATGATATGA
GTGAGAATACCCCAATGATGTCCACTGATAACTTCTCTGGCAACTATGTGGTACCCAAGCC
AACTAGGATATCCACTGAGCTCAATATTGGCAGTTCACAGTCTGACAACTTGTCACCTGAT
AGTCAGAGCAGCATGGTTTCCTTTGGCACGGAACTTATCGAACATGCGGGTTGCAACTCA
AGCAAAGTACGTGTTGGTTCGTTTCGATTGTTTGGCAAGATAATTGCTTTGAAAGAGCCTG
TCAGAAGCAGATTTGATGATGTTGGTTGCATGGATGATGTTGGAGGTAAAAGGCATGATG
AAGCGGTGAGTGAAAAGAACTCCTTAGATCTGTCATTGACTTATGGCTGTTCGAAGCTGC
TTGACAGGCTAGATGTCCAATGCCAACGAGCCTCAACTTTTGAAGGCTTCTCCTTGTGA

>GhARF17-2
ATGCCGCCTTCACGGTCTAGTCCTCCGGAGCTTCGTCATGTTGATCCAAGGATCTGGCGA
GCTTGTGCCGGCTCTTCCGTTCAGACCCCCACCGTTAATTCTAGGGTTTATTACTTCCCTC
AAGGCCACCTTGAACAAGTTTGCGGTTCCACGCCCAAACTGTCCTCTCTCGTACTGTCTA
GGCCTCTCATTAATTGCGTTATCTCCGACGTTCATTATCTTGCCGATCCGAAAACCGATGA
GGTCTTCGCTAAGCTTTTACTCACCCCTGTTGAAACTTCTAGGCTTCCTAATCAATTTCTAA
ATGTGAATGGTGAAGTTGAGGATTCTGATAGGAATAAGATCGTGTCGTTTGCTAAGGTTTT
AACACCCTCTGATGCCAACAACGGCGGTGGATTCTCCGTCCCGCGGTTTTGTGCCGATT
CTGTTTTCCCGCCGCTCGACTACAATGCTGATCCGCCGGTTCAGACTCTCTCCGTCACC
GACGTTCGCGGCGGTGTTTGGGAGTTTCGTCACATTTATCGAGGGACGCCACGTAGGCA
TCTGCTCACTACGGGATGGAGCAAGTTCGTTAATCAAAAGAAGCTTATCGCCGGCGATTC
TGTTGTTTTCATGAGGGACTGTAATTGGAAAATGTTTATTGGAGTCCGGCGAGCAATGAAG
GCGGGAGAAGGCGGAGGGGATTCGGGGAGGTGGAGAGAGCCGAGTGATGGAGGAGCA
ATGAAAGGGGAAGGAAGGGGGAGGATGACGGCGGAGGCAGTGGCTGAAGCGGCGGAG
AAGGCAGCGAGGGGGTTCCCGTTCGAGGTTGTGTATTATCCTCGGGCCGGTTGGACTGA
TTTCGTGGCAAGGGCGGTGTTGGTGGAGGCTGGAATCAGTACTTACTGGGCTGCGGGG
ACCAGAGTGAAGATGGCAGTTGAGACGGAGGACTCCTCGCGATTGGCTTGGTTTCAAGG
GACAGTTATGTCTGCTGCTGTACCGGATTCTGGCCCCTGGATTGGCTCGCCTTGGCGAAT
GCTTCAGGTTGCTTGGGATGAACCTGAAGTTCTCCAGGATGCAAGGAAAGTGAACCCAT
GGCAAGTTCAAATTTCTTCCTCCTCACCGCTTCCTTCCTCGTCCCCCTCCGAAAAGAGGC
TGAAGTTTTCTCAGGATTCAGGGCTGGCTGATGCAGAGGGTGAAATATTTCCTGTGTCAG
GGTTAACTAATTCAACAATGGGGTATTTGAATCCATCACTGTTGAATTACAATTCTTTTCCT
GCTGGCATGCAGGGAGCCAGGCAATATCATTTTCACTTGCAAAGTTTAACCAACGATGTG
AGTGAGAATACCCCAGTGATGTCCACTGATAACTTCTCTGGCAACTATGTGGTACCCAAG
CCAACTAGAATATCCACTGAGCTCAATATTGGCAGTTCACAGTCTGACAACTTGTCACCTG
ATAGTCAGAGCAGCATGGTTTCCTTTGGCACGGAACTTATTGAACATGGGGGTTGCAACT
CAAGCAAAGTAGGTGTTGGTTCGTTTCGATTGTTTGGCAAGATAATTGCTTTGAAAGAGCC
TGTCAGAAGCAGATTTGATGATGTTGGTTGCATGGATGATGTTGGAGGTAAAAGGCATGAT
GAAGCTGTGAGTGAAAAGAACTCCTTAGATCTTTCATTGACTTATGGCTGTTCGAAGCTGC
TTGACAGGCTAGATGTCCAATGCCAACGAGCCTCAACTTTTGAAGGCTTCTCCTTGTGA

>GhARF17-3
ATGCCGCCTCCATGGCCGCCGGCTGCGGAGGTTCGCCACGTCAATTTAAGGATCTGGCG
AGCTTGCGCTGGCTCTTCCGTTCAAATCCCCACTGTTAACTCTGTTGTTTACTACTTCCCT
CAAGGACACGTGGAACAATCTTGCGGTTCGACTCCGTTACTCTCTTCTCTCGTGCTCTCT
AGGCCTCTCATCCCTTGCGTTGTCTCCGACGTTCATTGCCTCGCCGATCCCAGAACTGAT

GAAGTCTTCATCAAACTCTTCCTCGTCCCTGTCGAGCCTCCTAGACTACCCAATGAATTTC
TAGACGTTAATGGTGAAGTTGAATATCCTGATAAGGTCGTGTCCTTCGCCAAGATTTTAAC
GCCGTCCGATGCCAACAATGGCGGCGGATTCTCCGTCCCCCGGTTTTGTGCTGACTCTA
TTTTCCCGCCTCTTGACTACAATGCTGACCCGCCTGTTCAGACTCTCACCGTCACCGACG
TTCGCGGCTGGGTTTGGGAGTTCCGCCACATTTACAGAGGAACGCCGCGTCGGCATCTG
CTCACTACGGGATGGAACAAATTTGTTAATCAAAAGAAGCTCATCGCCGGCGATTCTGTT
GTCTTCATGAGGGACTGTAATGGTAAAATGTTTATTGGAGTCCGTCGGGCTTTGAAGAGA
GGAGAAGGCGGTGGGGATTCCGGGAGGTGGAGAGAGCCAACTGGTGGAGGAGAAACG
AAAGGGGACGGAAGGGGGAGGATGACGGCGGAGGTGGTTGTGGAAGTGGCGGAGAGG
GCCGCGAAGCGGTTGCCATTCGAGGTTGTGTACTATCCTCGGCCCGGTTGGACAGATTT
CGTGGTGAGAGCGGAGTTGGTGGAGGCTGGACTCAATATATACTGGGCTGGTGGGACCA
GAGTGAAGATGGTAGTTGAAACAGAGGATTCCTCGAGAATGACGTGCTTTCAAGGGACG
GTTATTTCTGGTGCTTTGTCGGATTCCGACCCCTGGATTGGCTCTCCTTGGCGAATGCTT
CTGGTTGCATGGGATGAACCTGATCTCCAGAATGTAAGGAGAGTGAACCCATGGCAAGTT
GAAATTGCTACTTCCTTACCGCTTCAGTCTTCATTTCCCTTGGCGAAGAAGTCCAAGTTTT
CTCAGGAATCTGGGCTGGCTGATGCAGAAGGAGAAATAATGTTCCCTATGACAGGGTTAA
CCAATTCAACAATGAGGTACATGAATCCATCACTGTTGAATTACAATTCTTTTCCTGCTGGC
ATGCAGGGAGCCAGGCAAAATCATTTTCACGTGCAAGGTTTAACCAACCATGTGAGTGAG
AATACCCCAATGATGTCCACTGATGCTTCCTCTAGCAACTATTGGGTGCCCAAGTTAAAGA
GGATATCCACTGAGCTTAACATTGGCAGTTCACAGTCTGACAACTTGTCCCCAGATAGCC
AGAGCAGCATGGTATCCTTTGGCACTGAATTTACTGAAAATGCAGGCTGCAACTTGAGCA
AAGTAGGTGTTAATTCGTTTCAATTGTTTGGCAAGACGATTCATATGAAGGAGCCTGGTGG
AAGCATGTTTGGTAATGTTGGTAGCATGGAAGATGATAGTGGTAAAAGATATGATGAAGCT
GTGAGTGAAAAGAACTCGTTAGATCTTTCATCAACTAACAACTATTCAAAGCTGCATGACA
GGATAGATGTCAAAAGTGAGAGTGCTTCAGCTTTCAAAGGCTTTTCTTTGTGA

>GhARF17-4
ATGCCGCCTCCACGGCCGCCGGCTGCGGAGGTTCGCCACGTCGATTTAAGGATCTGGC
GAGCTTGCGCTGGCTCTTCCGTTCAAATCCCCACTGTTAACTCTATTGTTTACTACTTCCC
TCAAGGACACGTGGAACAATCTTGCGGTTCGACTCCGTTACTCTCCTCTCTCGTGCTCTC
TAGGCCTCTCATCCCTTGCGTTGTCTCCGACGTTCATTGCCTCGCCGATCCCAGAACTGA
TGAAGTCTTCATCAAACTCTTCCTCGTCCCTGTCGAGCCTTCTAGACTACCCAATCAATTT
CTAGACGTTAATGGTGAAGTTGAAGATCCTGATAAGATCGTGTCCTTCGCCAAGATTTTAA
CGCCGTCCGATGCCAACAATGGCGGCGGATTCTCCGTCCCCCGGTTTTGTGCTGACTCT
ATTTTCCCGCCTCTTGACTACAATGCTGACCCGCCTGTTCAGACTCTCACCGTCACCGAC
ATTCGCGGCGGGGTTTGGGAGTTCCGCCACATTTACAGAGGAACGCCGCGTCGGCATCT
GCTCACTACGGGATGGAACAAATTTGTTAATCAAAAGAAGCTCATCGCCGGCGATTCTGT
TGTCTTCATGAGGGACTGTAATGGTAAAATGTTTATTGGAGTCCGTCGGGCTTTGAAGAG
AGGAGAAGGCGGTGGGGATTCCGGGAGGTGGAGAGAGCCAACTGGTGGAGGAGCAAC
GAAAGGGGACGGAAGGGGGAGGATGACGGCGGAGGTGGTTGTGGAAGTGGCGGAGAG
GGCCGCGAAGGGGTTGCCATTCGAGGTTGTGTACTATCCTCGGCCCGGTTGGACAGATT
TCGTGGTGAGAGCGGAGTTGGTGGAGGCTGGACTCAATATATACTGGGCTGGTGGGACC
AGAGTGAAGATGGCAGTTGAAACAGAGGATTCCTCGAGAATGACGTGCTTTCAAGGGAC
GGTTATTTCTGGTGCTTTGTCGGATTCCGGCCCCTGGATTGGCTCTCCTTGGCGAATGCT
TCTGTGGTTTCTCATCCGGAGTAACATGGTTGCATGGGATGAACTTGATCTCGAGAATGTA
AGGAGAGTGAACCCATGGCAAGTTGAAATTGCTACTTCCTTACCGCTTCAGTCTTCATTTC
CCTTGGCGAAGAAGTCCAAGTTTTCTCAGGAATCTGGGCTGGCTGATGCAGAAGGAGAA
ATAATGTTCCCTATGACAGGGTTAACCAATTCAACAATGAGGTACATGAATCCATCACTGTT
GAATTACAATTCTTTTCCTGCTGGCATGCAGGGAGCCAGGCAAAATCATTTTCACGTGCAA
GGTTTAACCAACCATATGAGTGAGAATACCCCAATGATGTCCACTGATGCTTCATCTAGCA
ACTATTGGGTGCCCAAGTTAAAGAGGATATCCACTGAGCTTAACATTGGCAGTTCACAGTC
TGACAACTTGTCCCCAGATAGCCAGAGCAGCATGGTATCCTTTGGCACTGAATTTACTGA
AAATGCAGGCTGCAACTTGAGCAAAGTAGGTGTTAATTCGTTTCAATTGTTTGGCAAGAC
GATTCATATGAAGGAGCCTGGTGGAAGCATGTTTGGTAATGTTGGTAGCATGGAAGATGAT
AGTGGTAAAAGATATGATGAAGCTGTGAGAGAAAAGAACTCGTTAGATCTTTCATCAACTA
ACGACTATTCAAAGCTGCATGACAGGATAGATGTCAAAAGTGAGAGTGCTTCAGCTTTCA
AAGGCTTTTCTTTGTGA

>GhARF17-5
ATGGAGCTTGAAAAAAGCTTAGACCCAAATCTATGGCATGCATGTGCTGGATCCATGGTTC

AAATCCCACCAATAAAGTCCAAAGTCTATTATTTCCCTCAAGGCCATGCCGAGCACTCACT
TAACTCGGTGGATTTCCCGTTTTCCCGTCAAATCCCACCCCTTGTTCTTTGCCGAGTTGA
CTCAGTCAAATTCATGGCCGATGGTGAAACCGATGAAGTATATGCCAAGATCATGCTTACG
CCATTACAGAACAATGAGCCTGATGTTGGAGACGATGATGGTGTTTCAAATGGCGGCTGT
AATAATATTGAAAAAAAGGCTGCTTCTTTTGCTAAGACGTTGACTCAATCCGATGCTAACAA
CGGCGGTGGGTTTTCCGTTCCGAGATACTGTGCTGAAACCATTTTTCCCGCGTTGGATTA
TAGTGCCGATCCTCCGGTTCAAACCGTCGTCGCCGTTGATGTTCATGGTCAGCTATGGAA
GTTCAGGCATATTTATAGGGGAACTCCGAGGAGACATTTGTTGACTACAGGGTGGAGTGC
TTTCGTCAACCGGAAAAAACTCGTTGCCGGCGACTCAGTCGTGTTCTTGAGAGCTGAGA
ACGGCGGAATTCGTGTCGGGATCAGGCGAGCCAAGCTTGGGATTGGGGATGGTGATTGT
TACGGAAGTTTGGGTTTTTGTGGGAATTCGAAAGGGAACGGAAAAGTGAAGCCGGAGGA
GGTTTTGGAGGCGGCGAAGGCGGCTTCCGGTGGCCGGAAGCCGTTCGAGGTTGCTTAT
TATCCGAGAACAAGAGGGCCGGAGTTTTGTGTTAAGGCATCTGCAGTGAATACAGCAATG
AAGGTCCCTTGGTGTTGTGGTATGAGGTTCAAAATGGCTTTTGAGACTGAAGATTCTTCTA
GAATTAGTTGGTTCGTCGGAACCATATCGTCCGTTCAAGTTGCCGATCCGGTCCGGTGGC
CGGACTCGCCGTGGCGGCTTCTCGAGGTGACATGGGATGAACCGGATTTGCTGCAAAAC
GTTAACCGTGTGAATCCGTGGTCTGTTGAATTAGCATCGAACTTGCCCGCTATCCACCTCT
CGCCCTTTTTACGACCAATGAAGAAGTTGCGGCTCCCTCAACACCTTGATGGACTTTTAG
TGCCGTCTTTTCCAAGCAAGCCCCTTATCCCGAGCAGCCCGTTATATCGTTTATCTGATAA
TGCTCCTGTAGGGATACAGGGAGCCAGGCAAGTTCAATTCGGGTTACCTTTATCAGATCT
CCATCTTAAAAATAACCCGCATTCGGGACTGTGCCTGTCCGGTTTGCAGCGGTTTGATCT
ACACGATAGAATTCCCGATACATTCACAAACAGTCGCAATAATCTATCTTGCTTTTTAACAAT
GGGGAACTCTAACAAATCCAAAAACCCGAAAAAATGCCGGTTTTTACTCTTTGGTCAGCC
GATACTTACTGAGCAGCAACTGTCTCAGAGCTCTTCAAGTGAAGCTGTCTCGGAAGTTGT
TAGTGGAAACAGTTGCAAAACAAAAGATTCATCCGACGGTTCAGGATCTGTTATCGGGAA
CCAATTCTCTCCGGAGAAGTCATCTACTGCTCAATTTTTGTGGGATCGGGACAATCGAGC
CATAGAACCCTGCATGGATACCGGTCATTGCAAGGTATTCATGGAATCAGAGGATGTCGG
ACGAACTCTTGACCTCTCGGTTCTCGATTCTTATGAAGATTTATACAGGAGATTGGCTAAC
ATGTTCGGAATAGACAGATCGAAAATGTCCGAACATGTGCTGTATCGAGACGCAACCGGT
GCCATCAAACGAACTGGAGATGAACCATTCAGGTCACGGCTCACTGGAACCCGAACTGC
GGAAATTGGATTTCGAGGATCAAACAAGACAGGTCCTTTAAGCATATTTGCATGA

>GhARF17-6
ATGGAGCTTGAAAAAAGCTTAGACCCAAATCTATGGCATGCATGTGCTGGATCCATGGTTC
AAATCCCACCAATAAACTCCAAAGTCTATTATTTCCCTCAAGGCCATGCCGAGCACTCACT
TAACTCGGTGGATTTCCCGTTTTCTCGTCAATTCCCACCCCTTGTTCTTTGCCGAGTTGAC
TCGGTCAAATTCATGGCCGATGTTGAAACCGATGAAGTATATGCCAAGATCATGCTTACGC
CATTACAGAACAATAAGCCTGATGTTGGAGACGATGATGGTGTTTCAAATGGCGGCTGTAA
TAATATTGAAAAAAAGGCTGCTTCTTTTGCTAAGACGTTGACTCAATCCGATGCTAACAAC
GGCGGTGGGTTTTCCGTTCCGAGATACTGTGCTGAAACCATTTTTCCCGCGTTGGATTAT
AGTGCCGATCCTCCGGTTCAAACCGTCGTCGCCGTTGATGTTCATGGTCAGCTATGGAAG
TTCAGGCATATTTATAGGGGAACTCCGAGGAGACATTTGTTGACTACAGGTTGGAGTGCTT
TCGTCAACCGGAAAAAACTCGTTGCCGGCGACTCAGTCGTGTTCTTGAGAGCCGAGAAC
GGTGGAATTCGTGTCGGGATCAGGCGTGCCAAGCTTGGGATTGGGGATGGTGATTGTTA
TGGAAGTTTGGGTTTTTATGGGGATTCAAAAAGGAACGGAAAAGTGAAGCCGGAGGAGG
TTTTGGAGGCGGCGAAGGCGGCTTCCGGTGGCCTCGAGGTTGTTTATTATCCGAGAACA
AGCAGGCCGGAGTTTTGTGTTAGGGCATCTGCAGTGAATACAGCAATGAAGGTCCCTAG
GTGTTGTGGCATGAGGTTCAAAATGGCTTTCGAGACTGAAGATTCTTCTAGAATTAGTTGG
TTCGTCGGAACCATATCGTCCGTTCAAGTTGCAGATCCGGTCCGGTGGCCGGACTCGCC
GTGGCGGCTTCTTGAGGTGACATGGGATGAACCGGATTTGCTGCAAAACGTTAACCGTG
TGAATCCGTGGTCAGTTGAATTAGCATCGAACGTGCCCGCTATCCACCTCTCGCCCTTTTT
ACGACCGATGAAGAAATTGCGGCTCCCTCAACACCTTGACGGACTATTAGTGCAGTCTTT
TCCAAGCAAGCCCCTTAGCCCAAGCAACCCGTTTTATCGTTTATCTGATAATGCTCATGTA
GGGATACAGGGAGCCAGGCAAGTTCAATTCGGGTTACCTATATCGGATCTCCATCTTAAAA
ATAACCTGAATTCGGGACTGTGCCTGTCTGGTTTCCAGCGGTTTGATCTACACGATAGAA
CTCCCGATACATTCACAAACAGTTGCAATAATCTATCTTGTAATAAATCCAAAATCCCGAAA
AAATGCCGGTTTTTACTCTTTGGTCAGCCGATACTCACTGAGCAGCAACTGTCTCAGAGC
TCTTCAAGTGAAGCTGTCTCGGAAGTTGTTAGTGGAAACAGCTGCAAAACAAAAGATTCA
TCCAACGGCTCAGGATCTGTTATCGGGAACCAATTCTCTCCGGAGAAGTCATCTACTGCT
CGATTTTTGTGGGATCGGGACAATCGAGCCATAGAACCCTGCATGGATACCGGTCATTGC

AAGGTATTCATGGAATCGGAGGATGTCGGACGAACTCTTGACCTCTCGGTTCTCGATTCT
TATGAAGATTTATACAGGAGATTGGCTAACATGTTCGGAATAGAAAGATCGAAAATGTCCG
GACATGTGCTGTATCGAGACGCAACCGGTGCCGTCAAACGAACTGGAGATGAACCATTC
AGTGCATTTATGAAGATCACCAAAAGATTAACAATAAGAATGGATTCAAGCAACAATACTAT
TGAAAGGTCACGGCTCACCGGAACCCGAACTGCGGAAAATGGATTTCATGGATCAAACA
AGACAGGTCCTTTAAGCATGTTTGCATGA

>GhARF18-1
ATGGCTCATTTAGAAGGTAATCAAAGGGGTTCCTCGACTGCTCATGCGGTTTCAGGTTCG
GCTGGTGATGATCTATATCAAGAATTATGGAAGCTATGTGCAGGCCCCTTGGTGGAGATTC
CTCGGGTTCACGAGAGAGTTTTTTACTTCCCTCAGGGTCACATGGAACAATTAGAAGCAT
CGACAAATCAGGAACTTAGCAATCAAACCCCACTGTTTAATCTTCCTTCTAAGATCCTATGT
CGTGTTCTTCACGTGGAGTTACTGGCAGAACAAGAGACAGATGAAGTTTATGCTCAGATC
ACTTTGCAGCCTGAAGATCAAAGTGAGCCTACAAGTCTTGATCCCTTCCCAACCGAGGCT
CCAAAGAGGACAGTTCATTCCTTTAGTAAGATTCTAACGGCATCAGACACTAGCACTCATG
GAGGGTTCTCTGTTCTCCGAAAGCATGCAACTGAGTGCCTTCCTCCTTTGGACATGAATC
TAGCAACTCCAACTCAGGAGTTGGTTGCCAAAGATCTTCACGGGTATGAGTGGCGGTTCA
AGCATATATTTAGAGGGCAACCACGGAGACATTTACTTACAACAGGGTGGAGCACTTTTGT
GACTTCGAAGAGATTAGTTGCTGGAGATGCATTTGTGTTTCTTAGAGGTGATAATGGAGAA
CTAAGAGTTGGGGTTCGGCGGCTTGCTCGTCAACAGAGCACAATGCCTTCGTCTGTGATA
TCCAGCCAGAGCATGCATTTAGGAGTGCTTGCTACTGCTGCTCATGCTGTTACAACTCATA
CCCTCTTTGTTGTGTATTACAAACCAAGTACAAGCCAATTCATAATTGGAGTAAACAAGTAT
CTGGAGGCTATTAACAATGGATTCTCTGTTGGCATGCGTTTCAAGATGAGATTTGAGGGA
GAAGACTCTCCTGAAAGAAGGTTCACAGGTACCATAGTTGGGGTTGGAGATATTTCCCCA
CATTGGTCAGAATCTAAATGGCGGTCCTTGAAGATTCAATGGGATGAACCTGCAGCAATAC
AAAGGCCAGAGAGAGTTTCTCCTTGGGAAATAGAGCCATTTGTAGATTCTGCTTCTGCAA
ATTTACAACCTACTATAATGTGCAAAAGACCAAGACCTGTCGATATTCCAGCTTCTGAAATT
ACTACCAGTTCAGCTGGTTCAGCCTTCTGGTGCCGTGAGTCAATCCAGCCTCATGAACTA
ACACCAGTGGGAAGCACACTTGAGGTCCAGAGCAGTGAAAACCAGGTTATGTGTCCTAT
GAGGCAGAAAGAAGCTGATAATTGTCTTATCAATGGTAATGGAGGTTACAAATCAAGGACT
CCACCTGAAAATGCCTGGCCACCTTCTCCTCTTGTGAATGTATCTTTGAACCTTTTTCCTG
ATTCAATGGAGAACAACTACAAAACAGGAGCATTGCAAACCGCTCTCACTGGTTATGCCA
AGAGCCTAATGCATGACCAAGTTGTAAAAAGAAAAACTGAGACTTTCACAGGTTGCCGGT
TGTTTGGGTTTAATTTGACAGATAGCACTAGTGTAGCTGCCCCCCCTGACAAGGAACAAAT
GAGCACAAGTATTGACTACAATGGTGTGAGAGGGCCTATCCCTGCTGCGTTTCATGTTGA
ACAGAAACCAGAAACTTCCAAGGAGCAGAAGCAAGTTGCATCAGAGACATCAACCCAGG
AGATGCAAGCTAAGCAGGGTTCTGCAACTTCCATGAGAAGTCGTACCAAGGTACATATGC
AAGGGATTGCAGTTGGCCGTGCTGTTGACTTAACTGCACTGAAAGGATATAATGATCTCAT
AAATGAGCTGGAAAAAATGTTTGAGATCAAGGGAGAGCTTTGTCGTAGTGGTCAGTGGTC
CATTGTTTTTACTGATGATGAGGGTGATATGATGCTCGTGGGTGATGATCCCTGGGTTGAA
TTTTGTAAGATGGTGAGAAAGATCTTCATATATTCAAGCGAGGAGGTGAAGAAGATTAGTA
CAAGATGCAAATTTCCAGCAGCATCTATGGAGTGTGAAGGGACGGTTGTAAGCTTGGACT
CAGAGCATAGGTCTGTTTGA

>GhARF18-2
ATGGCTCATTTAGAAGGTAATCAAAGGGGTTCCTCGACTGCTCATGCGGTTTCAGGTTCG
GCTGGTGATGATCTATATCAAGAATTATGGAAGCTATGTGCAGGCCCCTTGGTGGAGGTTC
CTCGGGTTCACGAGAGAGTTTTTTACTTCCCTCAGGGTCACATGGAACAATTAGAAGCAT
CAACAAATCAGGAACTTAGCAATCAAACCCCACTGTTTAATCTTCCTTCTAAGATCCTATGT
CGTGTTCTTCACGTGGAGTTACTGGCAGAACAAGAGACAGATGAAGTTTATGCTCAGATC
ACTTTGCAGCCTGAAGATCAAAGTGAGCCTACAAGTCTTGATCCCTTCCCCACCGAGGCT
CCAAAGAGGACAGTTCATTCCTTTAGTAAGATTCTAACGGCATCAGACACTAGCACTCATG
GAGGGTTCTCTGTTCTCCGAAAGCATGCAACTGAGTGCCTTCCTCTTTTGGACATGAATC
TAGCAACTCCAACTCAGGAGTTGGTTGCCAAAGATCTTCACGGGTATGAGTGGCGGTTCA
AGCATATATTTAGAGGGCAACCACGGAGACATTTACTTACAACAGGGTGGAGCACTTTTGT
GACTTCGAAGAGATTAGTTGCTGGAGATGCATTTGTGTTTCTTAGAGGTGATAATGGAGAA
CTAAGAGTTGGGGTTCGGCGGCTTGCTCGTCAACAGAGCACAATGCCTTCGTCTGTGATA
TCCAGCCAGAGCATGCATTTAGGAGTGCTTGCTACTGCTGCTCATGCTGTTACAACTCATA
CCCTCTTTGTTGTGTATTACAAACCAAGGACAAGCCAATTCATAATTGGAGTAAACAAGTAT
CTGGAGGCTATTAACAATGGATTCTCTGTTGGCATGCGTTTCAAGATGAGATTTGAGGGA

GAAGACTCTCCTGAAAGAAGGTTCACAGGTACCATAGTTGGGGTTGGAGATATTTCCCCA
CATTGGTCAGAATCTAAATGGCGGTCCTTGAAGATTCAATGGGATGAACCTGCAGCAATAC
AAAGGCCAGAGAGAGTTTCTCCTTGGGAAATAGAGCCGTTTGTAGCTTCTGCTTCTACAA
ATGCACAACCTACTATAATGTGCAAAAGACCAAGACCTGTCGACATTCCAGCTTCTGAAAT
TACTACCAGTTCAGCTGGTTCAGCCTTCTGGTGTCGTGAGTCAATCCAGTCTCATGAACT
AACACCAGTGGGAAGCACACTTGAGGTCCAGAGCAGTGAAAACCAGGTTATGTGGCCTA
TGAGGCAGAAAGAAGCTGATAATTGTCTTATCAATGGTAATGGAGGTTACAAATCAAGGAC
TCCACCTGAAAATGCCTGGCCACCTTCTCCTCTTGTAAATGTATCTTTGAACCTTTTTCCT
GACTCAATGGAGAACAACTACAAAACAGGAGCATTGCAAACCGCTCTCACTGGTTATGCC
AAGGGCCTAATGCATGATCAAGTTGAAAAAAGAAAAACTGAGACTTTCACAGGTTGCCGG
TTGTTTGGGTTTAATTTGACAGATAGCGCTAGTGTAGCTGCCCCCCCTGACAAGGAACAA
ACGAGCACAAGTATTGGCTACAATGGTGTGAGAGGGCATGTCCCTGCTGCGTTTCATGTT
GAACAGAAACCAGAAACTTCAAAGGAGCAGAAGCAAGTTGCATCGGAGACATCAACCCA
GGAGATGCAAGCTAAGCAGGGTTCTGCAACTTCCATGAGAAGTCGTACCAAGGAAAACTT
CACGGTACATATGCAAGGGATTGCAGTTGGCCGTGCTATTGACTTAACTGCACTGAAAGG
ATATAATGATCTCATAAATGAGCTGGAGAAAATGTTTGAGATCAAGGGAGAGCTTTGTCGT
AGTGGTCAGTGGTCCATTGTTTTTACTGATGATGAGGGTGATATGATGCTCGTGGGTGAT
GATCCCTGGGTTGAATTTTGTAAGATGGTGAGAAAGATCTTCATATATTCAAGCGAGGAG
TGAAGAAGATTAGTACAAGATGCAAATTTCCAGCATCATCTATGGAGTGTGAAGGGACGGT
TGTAAGCTTGGACTCAGAGCATAGGTCTGTTTGA

>GhARF18-3
ATGCCTTCGTCAGTCATATCTAGCCAAAACATGCATTTAGGAGTGCTCGCTACTGCTGCTC
ATGCCGTTATGACTCAGACCCTCTTTGTTGTGTACTACAAGCCGAGGACTGTGTTCCTTAT
GTTCAGGACGAGCCATTTCATAATAGGAGTAAGTAAGTATCTCGAAGCTATTAACAACAGAT
CCTCCGTTGGTATGCGTTTCAAGATGAGATTCGAGGGCGAAGATTCCCCCGAGAGAAGG
TTCACTGGTACCATAGTCGGGGTTGGAGATGCTTCCCCACATTGGTCAGAATCTAAATGG
CGGTCCTTGAAGATTCAATGGGATGAACCTGCAATGATACAAAGGCCAGAACGAGTTTCC
CCTTGGGAAATAGAGCCATTCTCGGCTTCTGCTTCTATAAACCTCATACAACCAGCTGTAA
AGAGCAAAAGACCACGACCGGTTTATATTCCAGCTTCTGAAACGACTACAAATTCAACCG
GTTCAGCCTTTTGGTGTCACGGGTCAACCAAATCACATGAACTAGCCCGAGCAGGAAGC
ATAGCTGAAGTCCAAAGCAGTGAAAGCAGCCAAGTTGTTTGGCCTATGAGACAGAATGAA
GCCGATAACACGAGGGCTCGGCTCGAAAATGCTTGGCCACCTTCCTCTCTTGTGAACGT
CTCTCTAAACTTTTTCCGCAATCCAGCCGATGCCTCTCCTGTTGAATTGAGGACAAGCAAT
GACGTTATGCGCGACCAAGTTGAAAAAGGAAAGAAACTCGAGATTTCCACAGGCTGCCG
TTTGTTCGGTTTCAATTTGACAAATAGTAATAGTGCAGTCAGTGGAACCGTTATAGCTCCG
TCCCATATTGATGAAAAACCCGAAACTTTTCAGTCACCAAAGCAGCAAAAGCAAAATGCAT
CAGAGACATCAACCAAGGAGATAAAGGCTAAGCACGGTACCACTTCTTCTATGAGAACTC
GTACTAAGGTTCAAATGCAAGGGATTGCCGTCGGTCGTGCTATTGACTTAACCGTATTAAA
AGGATACGATGACCTCATAAATGAGCTAGAGAAAATGTTCGATATTGAGGGAGAGCTTCGT
CACCGTACTAAATGGTCTGTCGTTTTCACTGACAATGAAGGTGATATGATGCTTGTGGGCG
ACGATCCTTGGGTGGGATTTTGTAAGATGGTGAGAAAGATATTCATATATTCGGTCGACGA
GGTGAAGAAGATTAACGCGAGATGCAAATTTCAAGCCTCGTCTTTAGAAGGCGAAGGCAC
TGTTGTAAGCTTGGGTTTAGAGCATAGGTCCGGAACATGA

>GhARF18-4
ATGGCTCAACCAGAAGGTATTCCAAGGGAAACAGGTTTGGGTGTTGATGATCTGTATCCA
GAACTATGGAAATTATGTGCAGGCCCTTTAGTGGAGATTCCTCATGTTCAAGAAAGAGTCT
TTTATTTCCCTCAGGGTCACATTGAACAATTAGAAGCATCAACTAATCAAGAACTTAACTAT
GAAGCCCCACTGTTTAATCTTTCTTCAAAGATTCTATGTCGTGTTCTTCATGTCCAGTTACT
GGCTGAACAAGAAACAGAAGAGGTTTATGCACAAATAACTTTACAGCCTGAACCAGATCA
AAGTGAACTGACCAGTCCGGATCCGTTCCCGACCGAGGTTCCGAAGAGGGAAGTTCTTT
CCTTTTGTAAGATTTTAACTGCGTCGGATACGAGTACTCATGGAGGGTTTTCCGTTCTACG
GAAACATGCCACTGAGTGCTTGCCTCCATTGGATATGAATCAAGCTACCCCGACACAAGA
ATTAACCGCGAAAGATCTTCACGGATATGAGTGGCGGTTTAAGCATATATTTAGGGGACAA
CCTCGGAGACATTTACTTACGACGGGGTGGAGCACATTTGTGACTTCGAAAAGATTGGTT
GCGGGAGACGCATTCGTGTTTCTAAGAGGTGATAACGGAGAATTAAGAGTTGGGGTTCG
CCGGCTCGGTAATCAACAAAGCACAATGCCTTCGTCGGTCATATCAAGCCAAAGCATGCA
TTTAGGAGTGCTCGCTACTGCTGCTCATGCTGTTACGACTCAGACCCTCTTTGTTGTGTAC
TACAAGCCGAGGACAAGCCAATTCATAATAGGAGTAAGTAAGTATCTCGAAGCTATTAACA

ACAGATTCTCTGTTGGTATGCGTTTCAAGATGGGATTCGAGGGCGAAGATTCCCCTGAGA
GAAGGTTCACTGGTACCATAGTCGGGGTTGGAGATGCTTCCCCACATTGGTCAGAATCTA
AATGGCGGTCCTTGAAGATTCAATGGGATGAACCTGCAATGATACAAAGGCCGGAACGAG
TTTCCCCTTGGGAAATAAAGCCATTCTCGGCTTCTGCTTCTATAAACCTCATACAACCAGC
TGTAAAGAGCAAAAGACCACGACCGGTTTATATTCCAGCTTCTGAAACGACTACAAATTCA
ACCGGTTCAGCCTTTTGGTGTCACGGGTCAACCAAATCTCATGAACTAGCCCGAGCAGG
AAGCATAGCTGAAGTCCAAAGCAGTGAAAGCAGCCAAGTTGTTTGGCCTATGAGACAGAA
TGAAGCCGATGCAGGTTATAACTTGAGGACTCTGCTCGAAAATGCTTGGCCACCTTCCTC
TCTCGTGAACGTCTCTCTAAACTTTTTCCGCAATCCAGCGGATGCGTCTCCTGTTGAATTG
AGGACAAGCAAGGACGTTATGCGCGACCAAGTTGAAAAAGGAAAGAAACTCGAGATTTC
CACAGGCTGCCGTTTGTTCGGGTTCAATTTGACAAATAGTAATAGTGCAGTCAGTGGAAC
CGTTATAGCTCCGTCCCATATTGATGAAAATCCCGAAACTTTTCAGTCACCAAAGCAGCAA
AAGCAAAATGCATCAGAGACATCAACCAAGGAGATAAAGGCTAAGCACGGTACCACTTCT
TCTACAAGAACTCGTACTAAGGTTCAAATGCAAGGGATTGCAGTCGGTCGTGCTATTGAC
TTAACCCTATTAAAAGGATACGATGACCTCATAAATGAGCTAGAGAAAATGTTCGATATTGA
GGGAGAGCTTCGTCACCGTACTAAATGGTCTGTCGTTTTTACTGACAATGAAGGTGATATG
ATGCTTGTGGGTGACGATCCTTGGGTGTAA

>GhARF18-5
ATGCCTTTCAGTGGGTCCCTACACAACAGTGGCAACATCTGTTCGAGTGATGATGATCTAT
ACGCAGAGCTATGGAAGTTATGCGCAGGCCCGTTGGTGGAGATTCCTCGGAATCACGAG
AGAGTGTTTTACTTCCCTCAGGGTCACATGGAACAATTAGAAGCATCCACTAATCAGGAAC
TTAATAATCAAGCCCCACTGTTTAATCTTCCTTCTAAGATCCTATGTCGTGTTATTCATGTCC
AGTTACTGGCAGAACAAGAGACTGATGACGTTTATGCTCAGATCACTTTGCAGCCTGAAG
CAGATCAAAGTGAACCTACAAGTCCTGATCCTTGCCCAGCTGAGGCTCCAAAGAGGACA
GTTCATTCGTTTTGTAAGATTTTAACAGCATCGGATACGAGCACTCATGGAGGGTTCTCCG
TTCTTCGAAAGCATGCTACTGAGTGCCTGCCTCCTTTGGATATGAATCAGGCAACCCCTA
CACAGGAGTTGGCTGCTAAAGATCTTCATGGATATGAATGGCGGTTCAAGCATATATTTAG
AGGACAACCACGGAGACATTTGCTTACAACAGGGTGGAGCACCTTTGTCACTTCCAAGA
GATTGGTTGCGGGAGATGCATTTGTGTTTCTCAGAGGTGATAATGGAGAACTTAGAGTTG
GGGCTCGACGGCTTGCTCGCCAACAGACCACCATGCCTTCATCTGTGATTTCCAGCCAG
AGCATGCATTTAGGAGTGCTTGCTACTGCTGCTCATGCTGTTACAACGCAGACCCTCTTT
GTTGTGTACTACAAGCCAAGGACGAGTCAATTCATAATAGGTGTTAATAAGTATTTGGAGG
CCATTAAAAATGGATTCTCGGTTGGTATGCGTTTTAAGATGAGATTTGAGGGAGAAGACAC
TCCTGATAGAAGGTTCACGGGTACCATAGTTGGGGTAGGAGATTTTTCCCCACATTGGTC
GGAATCTAAATGGCGAACCTTGAAGATTCAATGGGATGAACCTGCAACAATACAAAGGCC
AGAGAGAGTTTCACCATGGGAAATAGAGCCATTTGCGCCTTCTGCTTCCATAAATCTTGTA
CAACCAGCTGTAAAGAACAAAAGGCCACGGCCTGTTGATATTCCAGTTTCTGAAATTACTA
CAAATTCAGTTGGTTCAACCTTCTGGGGTCGTGGTTCAACCCAATCTCATGAACTAACTCA
AGTAGGAAGCACACCTGAAATCCAAAGCAGTGAAAGCCAAGTTATGTGGGGTATGAGGC
AGAAAGAAGCTGATTACTGTCGAGATTATAATTCGAACGCCTGGCCACATTCTCCTCTTGT
GAATGTCTCTTTAAACCTTTTTCCTAATTCAGTGAGTGACAAAAACAGAACTGAAAAACCA
CAAATCACTCTCACTGGTTATGCCTTGCCTTCTCTATCGAGGCCAAGTAAGGGCCTGATG
CATGACCAAGTTGAGAAAGGGAAAAAATCTGAGACTTCTACAGGTTGCCGGTTGTTTGGG
TTTAATTTGACAGACACCATTAGTGCAGTTATCCCCACTGACAAGGAACAAACGAACACAA
CGGTTGATCACAATGGTGTTTGGGGGTATCTTGCAGCTGCGTCTCATATTGATCAGAATCC
AGAAACAGCTAAACAGAAGCATGTTGCAGCAGAGGCCTCGTCCAAGGAGATGCAGGCTA
AGCAGGGTGCATCCACCACTTCAACCAGAAGTCGTACTAAGGTACAAATGCAAGGGATTG
CGGTTGGTCGTGCTGTGGACTTAACTGTGTTGAAAGGATATGATGATCTCATTAATGAAGT
GGAGAAAATGTTTGATATCAAGGGAGAGCTTCGCCCCAGTGGTAAATGGTCCGTTGTTTT
CACTGATGATGAGGGTGATATGATGCTTGTTGGCGATGATCCATGGATGGATTTTTGCAAG
ATGGTGAGAAAGATCTTCATATATTCAAGCGAAGAGGTGAAGAAGATTAGTCCAAGATGCA
AATTTGCAGTGTCGTCTTTGGAGGGTGAAGGGACTGCTGTAACCATTGATTCAGAGCATA
AATCTGAAACATGA

>GhARF18-6
ATGGCTAATGTACAAGTTAATCCAAGGGGTTCTTCTATTGCTCACTCGGAGTCAGGTTCGA
GTGATGATGATCTATACGCAGAGCTATGGAAGTTATGCGCAGGCCCGTTGGTGGAGATTC
CTCGGAATCACGAGAGAGTGTTTTACTTCCCTCAGGGTCACATGGAACAATTAGAAGCAT
CCACTAATCAGGAACTTAATAATCAAGCCCCACTGTTTAATCTTTCTTCTAAGATCCTATGT

CGTGTTATTCATGTCCAGTTACTGGCAGAACAAGAGACTGATGACGTTTATGCTCAGATCA
CTTTGCAGCCTGAACCAGACCAAAGTGAACCTACAAGTCCTGATCCTTGCCCAGCTGAG
GCTCCAAAGAGGACAGTTCAATCGTTTTGTAAGATTTTAACAGCATCGGATACGAGCACTC
ATGGAGGGTTCTCCGTTCTTCGAAAGCATGCTACTGAGTGCCTACCTCCTTTGGATATGAA
TCAGGCAACCCCTACACAGGAGTTGGCTGCTAAAGATCTTCATGGATATGAATGGCGGTT
CAAGCATATATTTAGAGGACAACCACGGAGACATTTGCTTACAACAGGGTGGAGCACCTT
TGTCACTTCCAAGAGATTGGTTGCGGGAGATGCATTTGTGTTTCTCAGAGGTGATAATGG
AGAACTTAGAGTTGGGGCTCGACGGCTTGCTCGCCAACAGACCACCATGCCATCATCTG
TGATTTCCAGCCAGAGCATGCATTTAGGAGTGCTTGCTACTGCTGCTCATGCTGTTACAAC
GCAGACCCTCTTTGTTGTGTACTACAAGCCAAGGACGAGTCAATTCATAATAGGTGTTAAT
AAGTATTTGGAGGCCATTAAAAATGGATTCTCGGTTGGTATGCGTTTTAAGATGAGATTTGA
GGGGGAAGACACTCCTGATAGAAGGTTCACGGGTACCATAGTTGGGGTAGGAGATTTTTC
CCCACATTGGTCGGAATCTAAATGGCGGACCTTGAAGATTCAATGGGATGAACCTGCAAC
AATACAAAGGCCAGAGAGAGTTTCACCATGGGAAATAGAGCCATTTGCGCCTTCTGCTTC
CGTAAATCTTGTACAACCATCTGTAAAGAACAAAAGGCCACGACCTGTTGATATTCCAGTT
TCTGAAATTACTACAAATTCAGCTGGTTCAACCTTCTGGGGTCGTGGTTCAACCCAATCTC
ATGAACTAACTCAAGTAGGAAGCACACCTGAAATCCAAAGCAGTGAAAGCCAAGTTATCT
GGGGTATGAGGCAGAAAGAAGCTGATTACAGTCGAGGTTATAATTCGAACGCCTGGCCAC
ATTCTCCTCTTGTGAATGTCTCTTTAAACCTTTTTCCTAATTCAGTGAGTGACAAAAACAGA
ACTGAAAAACCACAAACCACTCTCACTGGTTATGCCTTGCCTTCTCTATCGAGGCCAAGTA
AGGGCCTGATGCATGACCAAGTTGAGAAAGGGAAAAAATCTGAGATTTCCACAGGTTGC
CGGTTGTTTGGGTTTAATTTGACAGACACCATTAGTGCAGTTATCCCCACTGACAAGGAAC
AAACGAACACAACGGTTGATCACAATGGTGTTTGGGGGTATCTTGCAGCTGCGTCTCATA
TTGATCAGAATCCAGAAACAGCTAAACAGAAGCATGTTGCAGCAGAGGCCTCGTCCAAG
GAGATGCAGGCTAAGCAGGGTGCATCCACTACTTCAACCAGAAGTCGTACTAAGGTACAA
ATGCAAGGGATTGCGGTTGGTCGTGCTGTTGACTTAACTGTGTTGAAAGGATATGATGAT
CTCATTAATGAAGTGGAGAAAATGTTTGATATCAAGGGAGAGCTTCGCCCCAGTGGTAAAT
GGTCCGTTGTTTTCACTGATGATGAGGGTGATATGATGCTTGTTGGCGATGATCCATGGAT
GGATTTTTGCAAGATGGTGAGAAAGATCTTCATATATTCAAGCGAAGAGGTGAATAAGATTA
GTCCAAGATGCAAATTTGCAGTGTCGTCTTTGGATGGTGAAGGGACTGCTGTAACCATTG
ATTCAGAGCATAAATCTGAAACATGA

>GhARF19-1
ATGGGGGAACGCGAGTTGACAGATGGTTGCTTGAGTGATGATGCAGGTGAAGCGCCGG
AGAGAAGGCACATCAACCCGGAGCTATGGCAGGCGTGTGCTGGGCCGCTGGTGAACCT
GCCGGCTGCTGGGACCCATGTTGTCTACTTTCCTCAAGGTCACAGTGAACAGGTTGCAG
CATCTATGAAGAGAGATGTGGATGCTCAAATTCCGAACTACCCAAATCTTCCTTCTAAGCTA
TTATGTCTCCTTCATAATGTCACCTTGCATGCAGACCCAGAGACAGATGAAGTCTATGCTC
AGATGACCCTCCAGCCTGTTTCTTCTTTTGATAAGGAGGCGTTACTGAGATCAGATCTTTC
TCTGAAGTCAAATAAGCCACAACTGGAATTCTTCTGTAAGACGTTGACAGCAAGTGATACG
AGCACTCATGGAGGTTTTTCTGTTCCTCGACGTGCCGCCGAAAAGATTTTCCCTCCTCTT
GATTTCTCGATGCAAAAACCTGCTCAAGAACTTGAGGCCAGGGATCTGCATGAAAATGTC
TGGAAGTTCCGCCATATCTATCGTGGAAAACCACAGCGCCACTTGCTTACAACAGGATGG
AGTCTATTTGTTAGTGGAAAGAGACTTTTTGCTGGTGACTCAGTTTTATTCATTAGAGATGA
AACACAGCAGCTTCTCTTGGGTATAAGGCGTGCTAACAGGCAACCTGCCAATCTATCATCA
TCAGTACTGTCTAGTGATAGCATGCATATTGGCATCCTCGCTGCAGCTGCTCATGCAGCA
GCAAATAATAGCCCCTTTACCGTGTTTTATAACCCAAGGGCTAGCCTATCTGAATTTGTTAT
TCCTTTAGCCAAGTACTATAAAGCTGTGTACAACCATCAAATATCACCTGGCATGCGCTTTC
GAATGATGTTTGAAACTGAAGAGTCAGGAACAAGAAGGTATATGGGTACAATTACAGGAAT
CAGCGATATTGACCCTGTAAGATGGAAAAACTCACAATGGCATAATTTGCAGGTTGGCTG
GGACGAGTCAACTGCTGAGGAAAGACGTAATCGAGTATCCATTTGGGAAATCGAACCAGT
TACAGCTCCATTTTTCATCTGTCCATCTCCATTGTTCAGATCTAAGCGTCCTAGGCAACCT
GGAATGCCGGCTGATGAATACTCCGACTTAGATAATCTATTCAAGAGGCCAATGCCTTGGC
TTGGTGATGATATATGCCTGAAGGATTCCGATGCCCATCCAGGGCTTAGTTTGGTCCAGT
GGATGAACATGCAGCAAAATCCTCTGCTGGCAAACTCTATGCAGCCAAATTTCATGCAGT
CTCTGGCTGGGTCTACTATGCAAAACTTTGACGGAGCAGATCTTTCCCATCAAATGGGCC
TTTCAGCACCACAAATGCCTCAACCCAACAACTTACAGTTCAATGCTCATAGGCTACCTCA
GAAAGTGCAGCAACTTGATCAAGTTCCAAAGCTACCATCTACAATGAACTCACTGGGATC
CATTATTCAGCCGCAACAGCTGAATGACATGACTCAGCAGTCGAGGCAAAATTTGGTTGC
TCAGCCTCTACCCTCTAGTCAAGTTTTGCAGCCTCAAGCCCTTGTCCAAAGTAACAATATC

CTTCATCAGCAGCAAACATCTAATCCAACTCATCAACTCCCTCTAAGTCTTCCTCAAAACC
TGCAGCAGCATCTTGTGGGCCCAAATCATCTGCAAAACCTAATGCATTCCCAGCTGCCTG
ATCCACTCAACCAGCATTTACAAGTGCCTGACAACCAGGTCCAGTTTCAACTGATGCAGA
AACTTCAGCAGCAACAACAGTTGCTTTTGGCACAGCAATCTGCACTTCAGCAGCCTGGTC
AACTTGCCCAACCCCAAGATCAACAAAGGCAGCTGTTAGATGCGTCTCAGAGCTTCTCTA
GTTCTGTGACAGCTAGCCAAGTGTTAGAGATGCCTCAAAACATACCTACCTTGCTACCTCA
ATCTAATGTTGCCCCACAGCAGATGCCTAAAAATAACAGCCAGGCAAATGTTTGGTTCTCT
CAGCCGCCTCTGCAGTCAAAAGTTCAGCAACAACAAACTGGAATGCTACCTGAAGTTCCT
GGTCTTGTAGGTCCCTTCCAAACTACAGCAACAAATCAGTTCTCCACAGCTGTTAGTAGT
GTAATGATGTCTGCTGCTGTAGCCGCACCTTCTGTGATTACTGATGATAATCCATCATGCT
CCACTTCGCCATCTACAAACTGTCCAAGTGTTCTTCAACCAATGATAGACAGCAGAGTCC
ACAGGAGTACTGGGTTAGGAGATGACATCAGTCAGTCTACTGCCACAGTATTGAATCCTAA
TGCCTTGGAGACGATGTCAACTAAGGCTAATATGGTTAAAGAACAGCAGCAAAAGTCTGTT
AAACCCTTGTTGAATATCTCCAAGAGTCAAAACCAAGGCTCTTTTGCCCCGCAAAACTGTA
TCAATGGTGCTACAGCACATGCAGATTGTTTGGACACATCATCTTCTACAACTTCAGTTTG
CCTTTCTCAAAGTGATGTTCATTTGCACCAAAACACACCGTCTTACAACCCTCAAACAATG
TTGTTGAGAGATACAAGTCAAGACAGAGAAGTTCAGGCATATCCAAGGAATAGTGTTTCAT
ACGGCAATAATATGGATAGCCAAATTGAGATGTCCATGAATTCTGACACTTTGTCTGCGAA
AGGCATGATGGGACTGGGGAAGGATTTTTCATATCACCTCTCTTCGGGAGGGGTACTTGC
CAGCTATGAAAACCCTAAAGATACTCAGCAAGAACTTTCTTCGTCAATGGTTTCCCAGTCA
TATAGAGTTCCAGATATGGCGTTTAACTCCATTGATCCCACTATAAACCATAGCAGCTTCAT
AAACCGCAGTGGATGGACCCCACCATCACAATTTCAGAGATTGCGAACATATACCAAGGT
ATACAAACGTGGAGCTGTTGGAAGATCAATAGATATAACTCGTTATTCAGGTTATGATGAGC
TCAAACAAGATTTGGCTCGTAGGTTTGGAATTGAGGGGCAGCTGGAAGACCGAGGGAGA
GTAGGCTGGAAACTAGTCTATGTGGATCACGAGAATGATGTTCTGCTAGTAGGAGATGAC
CCATGGGAGGAGTTTATCAACTGTGTCAGATGCATCAAAATACTCTCCCCTCAGGAAGTC
CAGCAGATGAGCGTGGATGGAGAGTTTGGGAACTCTGTCCTGCCTAATCAAGCCTGTAG
CAGCTCTGGCAATGGGAATGCATAA

>GhARF19-2
ATGAATGTGGTGGCCGCCGGAGTTGGATCTACAGCCAACGCGGCAGCTCCGGGTTCGG
CTGAAGGTGAAGCGCCGGAGAGAAGGCACATCAACCCGGAGCTATGGCAGGCGTGTGC
TGGGCCGCTGGTGAACCTGCCGGCTGCTGGGACCCATGTTGTCTACTTTCCTCAAGGCC
ACAGTGAACAGGTTGCAGCATCTATGAAGAAAGATGTGGATGCTCAAATTCCGAACTACC
CAAATCTTCCTTCTAAGCTATTATGTCTCCTTCATAATGTCACCTTGCATGCAGACCCAGAG
ACAGATGAAGTCTATGCTCAGATGACCCTCCAACCTGTTTCTTCGTTTGATAAGGAGGCG
TTACTGAGATCAGATCTTTCTCTGAAGTCAAATAAGCCACAACCTGAATTCTTCTGTAAGA
CGTTGACAGCAAGTGATACAAGCACTCATGGAGGTTTTTCTGTTCCTCGACGTGCCGCC
GAAAAGATTTTCCCTCCTCTTGATTTCTCGATGCAAACACCTGCTCAAGAACTTGTGGCCA
GGGATCTGCATGAAAATGTCTGGAAGTTCCGCCATATCTATCGTGGAAAACCAAAGCGCC
ACTTGCTTACAACAGGATGGAGTCTATTTGTTAGTGGAAAGAGACTTTTTGCTGGTGATTC
AGTTTTATTCATTAGAAATGAAACACAGCAGCTTCTCTTGGGTATAAGGCGTGCTAACAGG
CAACCTGCCAATCTATCATCATCAGTACTGTCTAGTGATAGCATGCATATTGGCATCCTCGC
TGCAGCTGCTCATGCAGCAGCAAACAATAGCCCTTTTACCGTGTTTTATAACCCAAGGGCT
AGCCTATTTGAATTTGTTATTCCTTTAGCCAAGTACTATAAAGCAGTGTACAACCATCAAATA
TCACCTGGCATGCGCTTTCGAATGATGTTTGAAACTGAAGAGTCAGGAACAAGAAGGTAT
ATGGGTACAATTACAGGAATCAGCGATATTGACCCTGTAAGATGGAAAAACTCACAATGGC
GTAATTTGCAGGTTGGCTGGGACGAGTCAACTGCTGGGGAAAGACGTAATAGAGTATCCA
TTTGGGAAATCGAACCAGTTACAGCTCCATTTTTCATCTGTCCATCTCCATTGTTCAGATCT
AAGCGTCCTAGGCAACCTGGAATGCTGGCTGATGAATACTCTGACTTAGATAATCTATTCA
AGAGGCCAATGCCTTGGCTTGGTGATGATATATGCCTGAAGGATTCCGATGCTCATCCAG
GGCTTAGCTTGGTCCAGTGGATGAACATGCAGCAAAATCCTCTGCTGGCAAACTCTATGC
AGCCAAATTTCATGCAGTCTCTGGCTGGGTCTACTATGCAAAACTTTGACGGAGCAGATC
TTTCCCATCAAATGGGCCTTTCAGCACCACAAATGCCTCAGCCCAACAACTTACAGTTCAA
TGCTCATAGGCTACCTCAGAAAGTGCAGCAACTTGATCAAGTTCCAAAGCTACCATCTACA
ATGAACTCACTGGGATCCATTATTCAGCCGCAACAGCTGAATGACATGACTCAGCAGTCA
AGGCAAAATTTGGTTGCTCGGACTCTACCCTCTAGTCAAGTTTTGCAGCCTCAAGCCCTT
GTCCGAAGTAACAATATCCTTCATCAGCAGCAAACATCTAATCCAACTCATCAACTCCCTCT
AAGTCTTCCTCAAAACCTGCAGCAGCATCTTGTGGGCCCAAATCATCTGCAAAACCTAAT
GCATTCCCAGCTGCCTGATCCACTCAACCAGCATTTACAAGTGCCTGACAACCAGGTCCA

GTTTCAACTGATGCAGAAACTTCAGCAGCAACAACAGTTGCTTTTGGCACAGCAATCTGC
ACTTCAGCAGCCTGGTCAACTTGCCCAACCCCAAGATCAACAAAGGCAGCTGTTAGATG
CGTCTCAGAGCTTCTCTAGTTCTGTGACAGCTAGCCAAGTGTTAGAGATGCCTCAAAACA
TACCTACCTTGCTACCTCAATCTAATGTTGCCCCACAGCAGATGCCTAAAAATAACAGCCA
GGCAAATGTTTGGTTCTCTCAGCCGCCTCTGCAGTCAAAAGTTCAGCAACAACAAACTGG
AATGCTACCTGAAGTTCCTGGTCTTGTAGGTCCCTTCCAAACTACAGCAACAAATCAATTC
TCCACAGCTGTTAGTAGTGTAATGACGTCTGCTGCTGTAGCCGCACCTTCTGTGATTACT
GATGATAATCCATCATGCTCCACTTCACCATCTACAAACTGTCCAAGTGTTCTTCAACCAAT
GATAGACAGCAAAGTCCACAGGAGTGCTGGGTTAGGAGATGACATCAGTCAGTCTGCTG
CCACAGTATTGAATGCTAATGCCTTGGAGACAATGTCAACTAAGGCTAATATGGTTAAAGA
ACAGCAGCAAAAGTCTGTTAAACCCTTGTTGAATATCTCCAAGAGTCAAAACCAAGGCTC
TTTTGCCCCGCAAAACTGTATCAATGGTGCTACAGCACTTGCAGATTGTTTGGACACATCA
TCTTCTACAACTTCAGTTTGCCTTTCTCAAAGTGATGCTCATTTGCACCAAAACACACTGT
CTTACAACCCTCAAACAATGTTGTTGAGAGATACAAGTCAAGAGGGAGAAGTTCAGGCAT
ATCCAAGGAATAATGTTTCATATGGCAATAATATGGATAGCCAAATTGAGATGCCCATGAATT
CTGACACTTTGTCTGCGAAAGGCATGATGGGACTGGGGAAGGATTTTTCAAATCACCTCT
CTTCGGAAGGGATACTCGCCAGCTATGAAAACCCTAAAGATGCTCAGCAAGAACTTTCTT
CGTCAATGGTTTCCCAGCCATATAGAGTTCCAGATATGGCGTTTAACTCCATTGATCCCAC
TATAAACCATAGCAGCTTCATAAACCGCAATGCATGGACCCCACCATCACAATTTCAGAGA
TTGCGAACATATACCAAGGTTTACAAACGTGGAGCTGTTGGAAGATCAATAGATATAACTC
GTTATTCAGGTTATGATGAGCTCAAACAAGATTTGGCTCGTAGGTTTGGAATTGAGGGGCA
GCTGGAAGACCGAGGGAGAGTAGGCTGGAAACTAGTCTATGTGGATCACGAGAACGATG
TTCTGCTAGTAGGAGATGACCCATGGGAGGAGTTTATCAACTGTGTCCGATGCATCAAAAT
ACTCTCCCCTCAGGAAGTCCAGCAGATGAGCATGGATGGAGAGTTTGGGAACTCTGTCC
TGCCTAATCAAGACTGTAGCAGCTCTGGCAATGGGAATGCATAA

>GhARF19-3
ATGAAGGCTCCACCAACTGGATTTTTGGCAAAGCCTGCTGAAGGAGATAGGAAGAGTATC
AATTCAGAATTATGGCATGCTTGTGCTGGACCACTGGTTTCTTTGCCACCAATTGGGAGTC
TGGTGGTTTATTTTCCTCAAGGCCACAGCGAACAAGTTGCAGCATCAATGCAGAAGGAGA
CTGATTTCATACCAAGTTACCCTAACCTTCCTTCCAAGTTGATTTGTGTCCTTCATAATGTT
ACATTGCATGCTGATCCAGAAACTGATGAGGTCTATGCCCAGATGACACTTCAACCTGTAA
ACAAAGTAGTGAAAGATGGGAAGAATCTTTATAGTACTTCTGCACAGTATGACAAGGAAGC
ATTACTGGCATCTGATATGGGCCTCAAGCACAGCAGGCAACCTGCTGAGTTCTTTTGCAA
GACTCTTACAGCTAGTGATACTAGCACTCACGGCGGATTTTCAGTCCCTCGTCGAGCAGC
TGAGAAGATCTTCCCTCCTCTGGATTTTTCCATGCAACCACCAGCTCAAGAGCTTGTAGC
AAGAGATTTGCATGAAAATGCATGGACTTTTAGACATATTTATCGAGGTCAACCAAAGAGG
CACCTTCTGACTACTGGTTGGAGCGTCTTTGTTAGCACAAAAAGACTCTTTGCTGGTGAT
TCTGTTCTTTTCATAAGAGATGCGAAGTCACAGCTTCTCTTGGGTCTAAGGCGCGCTAATA
GACAACAGCCGGCTCTCTCGTCATCAGTGATTTCTAGTGATAGCATGCATATAGGGATCCT
TGCTGCTGCAGCCCATGCTGCTGCAAATTTCAGCCCATTTACTATATTCTACAATCCAAGG
GCAAGCCCCTCTGAGTTTGTGGTACCTTTAGCGAAATATTACAAAGCCATGTACACCCAAG
TTTCTCTTGGCATGCGGTTTAGAATGATGTTTGAGACTGAGGAGTCTGGAGTACGAAGAT
ACATGGGTACAGTTACTGGTATCAGCGACCTGGATCCTGTGCGATGGAAAAACTCACAAT
GGCGCAATCTTCAGGTTGGTTGGGATGAATCTACAGCTGGAGAACGGCCCAGGCGAGTT
TCAATTTGGGAAATTGAGCCTGTTGTATCTCCTTTCTTCATATGTCCACCTCCTTTTTTCAG
ACCCAGGTTTCCAAAGCAACCAGGGATGCCAGATGATGATTCTGATGTCGAGAACGCTTT
CAAAAGAGCTATGCCTTGGCTTGGAGATGATTTTGGTATGAGAGGTACCCCTAGTTCAATC
TTCCCTGGTTTGAGTTTAGTTCAGTGGATGAATATGCAACAAAGTAATCAGTTTCCAGCTG
CTCAATCAGGATTCTTTCCATCAACGGTTTCTTTGAATCCACTGCATAATAACCTTAGCATT
GATGATCCTTCCAAATTATTGAATTTTCAAGCTCCTAGCCAGGTGCAACCACTGGGTAGCA
ATGGCTTCTCAACATCGATGTTTGTGCAACCTCAACAACTTTCAGTGCATCAATCCCAAAG
TCAGAACAAACAACTTATGGCAATGAGAAGCAATTCTGGTCTTATTGATGGAGATGCTCCA
CCGTGTTCGACCTTATCTTCTACCAATAATTGTCAGGTTTCCCCATCAAACCTTATAAACAG
AAGTCACCATGTACCATCCATATTGATGACAGATCCAGTTGTTGAGCCTCCAAGTACACTA
GCTCAAGAGCTCCTGAGCAAGCCTGACATTCAAATCAAACATGAGCCGCCCACCTCTAGA
GGACTAGACCAATCAAAGTACAAAAGTTCTGTAACAGACCAATTAGAAGCATCCTCTTCTG
GAACATCATATTGCTTGGACGCAGGCACCCTCCAGCATAATGCCTCCCTTACCTTTCTGGA
AGGTGATGTCCAATCACATTCTCGGAACAATCTTCCTTTTACAGCCAATATTGATGGATTG
GCACCTGAGACTTTGTTAACAAGGGAATATGACTCTCAAAAGGATCTTCAAAACATGCTTT

CTAATTATGGTGGGAACCCAAGAGATATTGACACTGAGTTGTCTACTGCTGCAATAAGCTG
TCAGTCATTTGGTGTGCCAAATATACCTTTCAAGACAGGATGCTCAAATGATGTTGCCATA
AATGAGACAGGGGTTTTAAATGGCGGATTGTGGACCAACCAAACTCAACGCATGCGAACA
TATACAAAGGTGCAAAAGCGTGGTTCTGTGGGAAGATCAATTGATGTGACCCGCTACAAA
GGGTATGATGAACTCCGGCATGATCTAGCCCGCATGTTCGGTATCGAGGGGCAGCTGGA
GGATCCACAAAGTTCTGACTGGAAATTAGTTTATGTGGATCATGAAAATGACATATTACTTG
TTGGTGACGATCCTTGGGAAGAATTTGTAAGTTGTGTTCAAAGCATAAAGATACTGTCGTC
AGCAGAAGTACAGCAGATGAGCTTGGATGGTAATCTTGGAAATGTGTCAGTTCCCAATCA
AGCTTGCAGTGGGACTGAAAACGGAAATGCATGGAGAGGACATTATGATGATACCTCAGC
AGTCTCATTTAACAGATGA

>GhARF23-1
ATGATAACAGTCATGGATTCTAGGAAAGAAGTTGTGAAAAATTCAGAAAAATGCTTAGATC
CTCAGCTATGGCATGCTTGTGCTGGTGGTATGGTACAAATGCCATCAGTGAACTCTAAGG
TGTTCTATTTCCCTCAGGGTCATGCTGAGCATGCCAATGGGAATGTAGATTTTGGGAATCT
CCCGATTCCGTCACTCGTCTTGTGTCGGGTTTCCGCCGTTAGGTTTATGGCGGATCCCGA
AACCGATGAGGTTTATGCTAAAATCATGTTGGTTCCTTTGAGAGAGAACAGTTTTGGGGTT
GAAGATGATGGTTTTGATGGGAATGTTGGGGTGGAGAACCCTGAAAAATCTGCTTCCTTT
GCTAAGACATTGACTCAGTCTGATGCTAACAACGGTGGGGGGTTCTCGGTTCCGCGGTAT
TGCGCGGAGACTATATTTCCTAGGTTGGATTATAATGCTGAACCCCCTGTTCAGACTATTCT
TGCTAAGGATGTTCATGGTGAGGTTTGGAAATTTAGGCATATATATAGGGGGACGCCTCGT
CGACATCTTTTGACGACGGGGTGGAGTAATTTCGTGAACCACAAGAAGCTTGTGGCTGG
TGATTCGATTGTGTTTCTTAGAGCTGATAATGGAGATCTTTGTGTGGGGATTCGTAGGGCG
AAAAGAGGACTTGGGGGTGGACATGAATTTCCGGGATGGAACTCTGCGAGTGGAACCTC
TGGTTCACAAATTGGGAGTTATTCTCCGTTTTTGAGGGAGGGGGAGAGTAAATTGATGAG
GAAGGATTGTAATGGGGATCCAAGGGGAAGAGTAAGGGCTGACTCCGTGATTGAAGCTG
CGAGTCGTGCAGCTAGTGGGCAGCCCTTTGAGGTCGTTTACTATCCACGAACTAGCACTC
CTGAGTTCTGCGTGAAGGCCTCATCCATTAGAGCTGCAATGCAAATCCAGTGGTATCCTG
GGATGAGGTTCAAAATGCCTTTCGAAACTGAGGACTCTTCACGGATTAGCTGGTTCATGG
GAGCGATATCTACTGCTAAGGTTGTCGATCCCATCCGTTGGCCTAATTCTCCATGGCGACT
ACTGCAGGTAGCATGGGATGAGCCAGATTTACTCCACAATGTGAAGCGTGTTAGCCCATG
GTTGGTTGAATTGGTCACAAACATACCAGCTATTAATCTTAATCCTTTCTCGCCACCAAGG
AAAAAGATGCGGCTTCCACAACACCCAGACATTTCCTTTCTTAACCAAATTCCAATGCCAT
CATTTTCCGGGAACTCTTTCAGATCCAGCAGCCCCATGCGTTGCATTACTGACAACATTCC
TGGAGGTATACAGGGAGCCAGGCATGAACCGTTCGGATTATCTTCATCAGATCTCCGCTC
CAACAAGCTGCATTCAGGTCTGTTTCCATCTGGTTTTCATCAACTTGATCGTACTGCCCCA
CCTACTAGACTTTCCGGCGACAACTTTTGTAGCGACAACGCGAACAATACCAATATATCTT
CCTTGTTGACAATAGGAAATCCGACCCAGAGTTTGAAACAAAGCAATGATAGCAAGACAC
CCCATATTGTATTGTTCGGTCAACTCATTTTCTGTGAGCAGCGGGCTTCTCAGAGCTGCTC
AGGTGATACAGTTGGAAACAGTTCATCAGATGGGAATACAGAGAAGACTGCTATTTCCTCT
GATGGAACTGGATCTGTGTTACATCAAAATGTTCGGGAAAATTCTTCGGATGAAGGGTTTC
CTTGGTGCAAAGAGCATCAAAAAACTGATCTAGGATTGGAGACTGGTCATTGCAAAGTGT
TTATGGAATCAGAGAATGTAGGTAGAACCCTTGATCTTTCAGTTCTGGGATCATATGAAGA
GCTGTTTGGGAAGCTGGCCAACATGTTTGGCATAGAAAGTTCAGAGATGCTGAGCAGTG
TGCTCTACCGCGATGCTGCCGGTTCAGTTAAAAACACTGGAGATGAGCCCTTCAGCGAG
TTTATGAAGACAGCAAGGAGGCTAACGATTCTCATGGATTCAAGCAGTGACAACTTAGAAA
GATAG

>GhARF23-2
ATGATAACAGTAATGGATTGTAGGAAAGAAGTGGTTAAGACTCCAGAAAAATGCTTAGATC
CTCAACTATGGCATGCTTGTGCTGGTAATATGGTGCAAATGCCAGCGGTGAACTCCAAGG
TGTTCTATTTCCCTCAAGGTCATGCTGAACATGCCAATAGGAATGTAGATTTTGGGAGTCT
TTTAATTCCTTCACTCATCCTATGTAGAATTTCGGCCATTAAGTTTATGGCAGATCCTGAAA
CTGATGAGGTTTATGCTAAAATTATGCTGGTTCCTTTCAGAGAGAATGATTTTGGGTATGAA
GATGGTTTTGATGGAAATATTGGGATGGAGAATCTTGAAAAACCACCTTCTTTTGCCAAGA
CATTGACTCAATCCGATGCTAATAATGGTGGGGGTTTCTCAGTTCCGCGGTATTGTGCAGA
GACTATATTTCCTAGGTTGGATTATAGTGCTGAACCCCCTGTTCAGACCATTCATGCAAAG
GATGTACATGGTAATGTTTGGAAATTTAGGCATATATATAGGGGAACACCTCGCCGGCATCT
TTTGACGACGGGGTGGAGTAATTTTGTGAATCATAAGAAGCTTGTGGCTGGTGATTCGAT
TGTCTTTCTTCGAGCAGAGAATGGAGATCTTTGTGTGGGAATCCGTAGGGTGAAGAGAG

GTATTGGAAGTGGACATGAGTATCCTTCTAGTTGGAATTTGGGGGATGGAAGCTCCGGTT
ATTCTCCACTCTTGAGGGAGGGGGAGAGTAAATCGATGAGGAATGATTCGAATGAGGATC
TGAGGGGAAGAATAAGGCCCGAGAATGTGATTGAAGCTGTGACTCGTGCTGCCAATGGG
CAACCCTTTGAGGTTGTTTACTATCCACGAGCAAGCACTCCTGAGTTCTGTGTGAAGGCC
TCATCAGTTAGAACTGCAACGCAAATCCACTGGTATCCTGGGATGAGGTTCAAAATGGCT
TTTGAAACCGAGGACTCCTCACGGATTAGCTGGTTTATGGGAACAATATCTATCGCTCAGG
TTGTTGATCCCATCCATTGGCCTAATTCTCCATGGCGCCTTCTTCAGGTGGCATGGGATGA
GCCAGATTTACTACACGACGTGAAGCGTGTTAGCCCATGGTTGGTTGAATTGGTGACAAA
CATACCAGCCATCCATCTTAATCCTTTCTCGCCCCCGAGGAAAAGGATGCGGCTTCCGCA
ACACCCTGATTTTTCTTTACTTGGTCAAATTCCAATGCCATCTTTTTCTGACAGCACTTTCA
GGTCCAGCAGCCCCGCATGTTGCATTACAAACAACATTCCTGGAGGCATACAGGGAGCC
AGGCATGCACCGTTTGGATTATCTTCATCAGATCTCCGTTCCAGCAAGCTGCAGTCGGGC
CTCTTTCCACATGGTTTTAATCAGCTTGATCATACTATCCCACCTATGGGACACTCCAGTGA
CTATGGGAACAATAGGAATATTTCTTCCTCGCCCACAATGGGAAATCTTACCCAAAGTTTAA
AAGAAAGCAATGAAATAAAGACACCCCATATCTTATTGTTTGGTCAACTCATTTTCTGTGAG
CAGCAGGTTTCTCAGAGCTGCTCAGATACAATTGGAAACAGTTCATCCAACGGGAATACA
GAGAAGACTATGATTTCTTCCGATGCCTCTGGATCTGCATTACATCAAAATGCTCGTGAAA
ATTCTTCAGATGAAGGGTCTCCTTGGTACAAAGAGCTCCCAAAAACCAACATGGGGTTGG
AGACTGGTCACTGCCAAGTCTTCACGGAATCAGAGAATGTGGGAAGAACCCTAGATCTTT
CAGTTCTCAGATCATATGAAGAGCTGCACGGCAAGCTAGCCAACATGTTTGGCGTAGAAA
GTTCAGATATGCTGAGCAATGTGTTCTACCACGATGCTGCTGCTTCTGTTAAGCACACAG
GAGATGAGCCCTTCAGTGAGTTTTTGAAGACAGCAAGGAGGCTGACCGTTCTTACAGATT
CAGGCAGTGACAACATGGGAAGATAG

>GhARF23-3
ATGATAACAGTAATGGATTGTAGGAAAGAAGTGGTTAAGACTCCAGAAAAATGCTTAGATC
CTCAACTATGGCATGCTTGTGCTGGTAATATGGTGCAAATGCCAGCGGTGAACTCCAAGG
TGTTCTATTTCCCTCAAGGTCATACTGAACATGCCAATAGGAATGTAGATTTTGGGAGTCTT
TTAATTCCTTCACTCATCCTATGTAGAATTTCGGCCATTAAGTTTATGGCAGATCCTGAAAC
TGATGAGGTTTATGCTAAAATTATGCTGGTTCCTTTGAGAGAGAATGATTTTGGGTATGAAG
ATGGTTTTGATGGAAATATCGGGATGGAGAATCTTGAAAAACCACCTTCTTTTGCTAAGAC
ATTGACTCAATCCGATGCTAATAATGGTGGGGGTTTCTCAGTTCCCCGGTATTGTGCAGAG
ACTATATTTCCTAGGTTGGATTATAGTGCTGAACCCCCTGTTCAGACCATTCATGCAAAGGA
TGTACATGGTAATATTTGGAAATTTAGGCATATATATAGGGGAACACCTCGCCGGCATCTTT
TGACGACGGGGTGGAGTTATTTTGTGAATCATAAGAAGCTTGTGGCTGGTGATTCGATTG
TCTTTCTTCGAGCAGAGAATGGAGATATCTGTGTGGGAATCCGTAGGGTGAAGAGAGGTA
TTGGAAGTGGACATGATTATCCTTCTAGTTGGAATTTGGCGGATGGAAGCTCTGGTTATTC
TCCACTCTTGAGGGAGGGGGAGAGTAAATCGATGAAGAATGATTCGAATGGGGATCTGA
GGGGAAGAATAAGGCCCGAGAATGTGATTGAAGCTGTGAGTCGTGCCGCCAATGGGCAA
CCCTTTGAGGTTGTTTACTATCCACGAGCAAGCACTCCTGAGTTCTGTGTGAAGGCCTCA
TCAGTTAGAGCTGCAATGCAAACCCACTGGTATCCTGGGATGAGGTTCAAAATGGCTTTT
GAAACCGAGGACTCCTCACGGATTAGCTGGTTTATGGGAACAATATCTACCGCTCAGGTC
GTTGATCCCATCCGTTGGCCTAATTCTCCATGGCGCCTTCTTCAGGTGGCATGGGATGAG
CCAGATTTACTACACGATGTGAAGTGTGTTAGCCCATGGTTGGTTGAATTGGTGACAAACA
TACCAGCCATCCATCTTAATCCTTTCTCGCCCCCAAGGAAAAGGATGCGGCTTACGCAAC
ACCCTGATTTGTCTTTACTTGGCCAAATTCCAGTGCCATCTTTTTCTGACAGCACTTTCAG
GTCCAGCAGCCCCGCATGTTGCATTACAAACAACATTCCTGGGGGCATACAGGGAGCCA
GGCATGCACCGTTTGGATTATCTTCATCAGATGTCCGTTCCAGCAAGCTGCAGTCGGGCC
TCTTTCCACATGGTTTTAATCAGCTTGGTCATACTATCCCTCCTATGGGACACTCCAGTGA
CTATGGAAACAATAGGAATATTTCTTCCTCGCATACAATGGGAAATCTTACCCAAAGTTTAA
AAGAAAGCAATGAAATAAAGACACCCCATATCTTATTGTTTGGTCAACTCATTTTCTGTGAG
CAGCAGGTTTCTCAGAGCTGCTCAGATACAATTGGAAACAGTTCATCCAACGGGAATACA
GAGAAGACTATGATTTCTTCCGATGCCTCTGGATCTGCATTACATCAAAATGCTCGTGAAA
ATTCTTCAGATGAAGGGTCTCCTTGGTACAAAGAGCTCCCAAAAACCAACATGGGGTTGG
AGACTGGTCATAGCCAAGTCTTCACGGAATCAGAGAATGTGGGAAGAACCCTAGATCTTT
CAGCTCTCAGATCATATGAAGAGCTGCACGGCAAGCTAGCCAACATGTTTCGCGTAGAAA
GTTCAGATATGCTGAGAAACATGTTCTACCGAGATGCTGCTGCTTCTGTTAAGCACACGG
GAGATGAGCCCTTCAGTGAGTTTTTGAAGACAGCAAGGAGGCTGACCGTTCTTACAGATT
CAGGCAGTGACAACATGGGAAGATAG

>GhARF24-1
ATGGACGGTGAACGGAATGGTTTGAAGGCTAAGGTCCCATTTCACCCCAGAAGACGTATC
AACGTCTATGGCTTGCCGTTGCTTCCTCCGAACAATCAAGGAGAAAAAGATGATCTACAT
GTTCAACTATGGCATGCATGCGCTGGTCCTTCAGTTTATGTGCCACACGCTGGGGAAAAG
GTTCTGTATTTCCCTCAAGCTCACATAGAACAGGTGGATGCATGTATGAATCAAGATGGGA
TAATGGAAATGCCTATCTACAATTTGCCTTCTAAGATCCTTTGCAGGGTGATGCATGTTCAG
CTTAAGGTTGAACCTGGCACAGATGAGGTCTTTGCACAAATAACACTGATTCCAGAAGCA
GAGCAAGATGAGGAAAGTTTGGAGCATAGAAATTATCAGCCTTTAGCCCAGAAAGCTTATC
CAGTGTTCTTTAGAAAGAAACTCACTCCATCAGATACAAGCACACATGGCGGATTCTCTAT
CCCAAAGCGACATGTTGATGATGGGTGGATCCCACCCCTGGACATGTCTCAGCAAACCC
CACAACAGGAATTGGTCGCAATAGACTTGCACGGTTTTGAGTGGCGCTTTCGACATATTTA
TCGAGGTCAGCCAAAAAGGCACTTGCTTACAAGCGGCTGGAGTACATTTCTGACTTCAAA
GAAGCTTCTTGCTGGGGATGAATGTATCTTTCTTAGAGGGGAAAAAGGAGAGCTCCGTCT
TGGAATTCGGCGAGCAACGACTGTTCTGAAATATACATCAACATCCGTCATATCTGGTCAT
AGCATGCGTCATGGCATACTAGCAAGTGCTTTTCATGCCTTCTCTACCAGAAGCATGTTTA
ATGTCAACTACCGTCCTTGGTCTACTTCTTCTGAATTTATCATCTCATTAGATCGGTATATGA
AGTCGGCTCAAATTGACTATTGCATCGGGACAAGATTTAGAATGCGATTAGAAGGCGAAG
AATGTGCGGAACAAAGACCTTCTGGCACTATCATAAGCATTGAAGATGTGGATCATACTAG
GTGGCCTAATTCTGAATGGAGATGTCTGAAGGTGAAATGGGATCTCACAGCAGGTGAAAA
TTTTCATCCTGAAAGAGTTTGTCCTTGGAACATTGAACCAACGGAATTTAGTATCAAGAAG
AGACCCTCCATTCTACGTAACCAAAAGAAGGCTCGTACTGATGATGTATCATCCCCTGGGT
TTTCTACCCTGCTAATGGATGGCATGTGGAGTGGTTCCATTAAATACGAATCTCAAAGTAG
CTCAGGGGTCTTGCAAGGTCAAGAAGATAGTGACACAGATGGTAATCAACCCGATGCTCT
AAGAAAACCATTACCACATTGCCTCCCACTAAATCACAGTTGGGACTCAATGCAACAGCC
AATACAGAACCAACGAGAGATTGGTGCAGCACCCTTTTCTGGTGGACAAGTAGAAAGTTT
GGGTCTTCATAATAGTTGGTCCACAACATTCTCCTCCTCAAATGGAGTACATGAAGATGCT
ATTGCTAGCAGAAAAATTTCAGTTCCAAATGTTAATTCTCAGGAATGGAGTATTTCAGAACC
AAGGAATGAAAATGAAACATCATGGTGCGAACCAAAGGAGGGACATGCATGCATGCTTTT
TGGAGTAAATTTAGTTACCGGTCCACTGGAGCCCCCTTCACCCCAACTTGTCACTTCTAG
CGAGCTTGAAAGTCATTGTTCTATTCCTCCAACTTCTCAGTCAACTGTTTCGAAACCTTATA
AGGGTACATCTAGCAAGCAGTGTGACAACTGCTGTTCTGCTAGCAATTGGAGTTGCACCA
AGGTACTCAAGCATGGGACTGCTCTTGGAAGATCAGTTGATATCACTAGATTTGACGGATA
TAAAGACCTCATCTCTGAGCTTGATCGCATGTTTGATTTTAATGGACGATTGATTGATGGAA
GCAGCGGCTGGCATGTAACCTTTACTGATGATATAGGGGAGATGAGGATGATTGGAGATC
ATTACCCATGGCAGAAATTTCAGAATGAGGTCCGAAGGATGCTGATCAACCCGAAGGAAG
AAATTGGAAGGGTGAATCAGAGCTCACTACTGGCGCCTTATGATGAAGTGTTTTAA

>GhARF24-2
ATGGACGGTGAACGGAATGGTTTGAAGGCTAAGGTCCCATTTCACCCCAGAAGACGTATC
AACGTCTATGGCTTGCCGTTGCTTCCTCCGAACAATCAAGGAGAAAAAGATGATCTACAT
GTTGAACTATGGCATGCATGCGCTGGTCCTTCAGTTTATGTGCCGCGCGCTGGGGAAAA
GGTTCTGTATTTCCCTCAAGGTCACATAGAACAGCTGGATGCATGTATGAATCAAGATGGG
ATAATGGAAATGCCTATCTACAATTTGCCTTCTAAGATCCTTTGCAGGGTGATGCATGTTCA
GCTTAAGGTTGAACCTGGCACAGATGAGGTCTTTGCACAAATAACACTGATTCCAGAAGC
AGAGCAAGATGAGGAAAGTTTGGAGCATAGAAATTATCAACCTTTACCCCAGAAAGCCTAT
CCAGTGTTCTTTAGTAAGAAACTCACTCCATCAGATACAAGCACACATGGCGGATTCTCTA
TCCCAAAGCGACATGTTGATGATGGGTGTCTCCCACCCCTGGACATGTCTCAGCAAACC
CCACAACAGGAATTGGTCGCAATAGACTTGCACAGTTTTGAATGGCGCTTTCGACATATTT
ATCGAGGTCAGCCAAAAAGGCACTTGCTTACAAGCGGCTGGAGTACATTTCTGACTTCAA
AGAAGCTTCTTGCTGGGGATGAATTTATCTTTCTTAGAGGGGAAAAAGGAGAGCTCCGTC
TTGGAATTCGGCGAGCAGCGACTGTTCTGAAATATACATCAACATCCATCATATCTGGTCAT
AGCATGCGTCATGGCATACTAGCAAGTGCTTTTCATGCGTTCTCTACTAGAAGCATGTTTA
ATGTCAACTACCGTCCTTGGTCTATTTCTTCTGAATTTATCATCTCATTTGATCGGTATATGA
AGTCGGCTCAAATTGACTATTGCATCGGGACAAGATTTAGGATGCGATTAGAAGGCGAAG
AATGTGCGGAACAAAGGCCTTCTGGCACTATCATAAGCATTGAAGATGTGGATCATACTAG
GTGGCCTAATTCTGAATGGAGATGTCTGAAGGTGAAATGGTATCCCACAGCAGGTGAAAA
TTTTCATCCTGAAAGAGTTTGTCCTTGGAACATTGAACCAACGGAATTCAGAATCAAGAAG
AGACCCTCCATTCTACATAACCAAAAGAAGGCTCGTACTGATGATGTATCATCCCCTGGGT
TTCCTACCCTGCTAATGGATGGCATGTGGAGTGGTTCCATTAAATACGAATCTCAAAGTAG
CTCAGGGGTCTTGCAAGGTCAAGAAGATAGTGACACAGATGGTAATCAACCCGATGCTCT

AAGAAAACCATTACCACATTGCCTCCCACTAAATCACAGTTGGGACTCAATGCAACAGCC
AATACAGAACCAACGAGAGATTGGTGCAGCACCCTTTTCTGGTGGACAAGAAGGGAGCA
TTTCAGAACCAAGGAATGAAAATGAAACATCATGGTGCGAACCAAATGAGGGACATGCTT
GCATGCTTTTTGGAGTAAATTTAGTTAACGGTCCACTGGAGCCCCCTTCACCCCAACTTGT
CACTTCTAGCGAGCTCGAAAGTCATTGTTCTATTCCTCCAACTTCTCAGTCAACTGTTTCG
AAACCTTCTAAGGGTACATCTAGTAAGCAGTGTGAGAACTGCTGTTCTGCTAGCAATTGGA
GTTGCACCAAGGTACTCAAGCATGGGACTGCTCTTGGAAGATCAGTTGATATCACTAGATT
TGGCGGATATAAAGACCTCATCTCTGAGCTTGATCGCATGTTTGATTTTAATGGACGATTGA
TTGATGGAAGCAGCGGCTGGCATGTAACCTTTACTGATGATGAAGGGGAGATGAGGATGA
TTGGAGATCATTACCCATGGCAGAAATTTCAGAATGAGGTCCGAAGGATGCTGATCAACC
CGAAGGAAGAAATTGGAAGGGTGAATCAGAGCTCATTAATGGCGCCTTATGATGAAGTGT
TTTAA

>GhARF24-3
ATGATCTCTATACTAAACTATGGCATGCATGCGCGGGTCCTTCTGTTTATGTTCCTCGCTCT
GGAGATAAAGTCTTGTACTTCCCTCAAGGTTGAGGCATACATGAGCGAAGATGGCACTAT
GGAAATGCCCATCTACAATTTACCTTGGAAGATCCTCTGCAGGGTTTTGCATGTTGAGCTT
AAGGTTGAACGTGACACAGATGAGATCTTTGCAGAAATTATTTTGCTTCCAGAGGCAGAG
CAAGATGAGCAAAGAATGGAGCATAGATATTATCGAGCGTCACCTCGGGAAAATTATTCTC
GTTACTTTAGTAAGAAGCTAACTCCATCGGATATAAAGACACACGGTGGATTCTCTATCCCA
AAGCGGCATGCCAATGATGGGTGTCTTCCGCTCTTGGACATGTCTCAGGAAATCCCCCA
GCAGGAACTGCTCGCAACTGACTTGCTTGGTCATCCATGGTACTTTCGACATGTTTTTCG
TGGCTATCCAAAAAGAAATTTGCTTACCACCGGTTGGAGTACCTTTGTCACCTCGAAGAA
GCTTGCTGCTGGGGATTCATTTATCTTTCTAAGAGGGGAAAATGGAGAGTTCGGTGTTGG
AGTTCGCCGATCAATGACAAAGCTACTGAACAGTCCATCTCCATCGATCATATCCGCTCAC
AGTGTGCGACATGGAATACTTGCCAGTGCTTTCCATGCCTTTGCAACCAGAAGCATCTTTA
ACGTCTACTACCGTCCTTGGGCAAGGTCTTCTGAATTTATCACTCCACTTGATCAGTATATA
AAGGCGGTTCAATTTGACTACTGCTTCGGGACAAGATGTAGAATGCGAGTTGAAGGTGGA
GAATCTGGGGAACAGAGATCCCTTGGCACTATCATTGGCACTGAAGATCTCGATCCTATTA
GATGGCAGAATTCCAAATGGAGATGTGTGAAGGTGAAATGGGATCCCACAGCGAGTTCG
GTTTTGCTTCCCAAAAGAGTTTGTCCTTGGAGCATCGATCTCACGGAATTCACCAAGAAA
AAGAAAGCTTCCACTCTGCATCATCAGAAGAGGGCTCGCCCCAACAACGCATCATCCCCT
GAGTTTTCTAGCTTGCTTATGGATGGCATGTTGCATGGTACAGCTAAAAATCAATCTCAAA
GTAGTTCAGGGGTCTTGCAAGGTCAAGAAGACAGTGCCACATGCGTGAATCAATCCAGT
GTACTGCAACAATCATTGCCGCATCTTCTCCCGCAAGATCCTGGCTCTGCCTCAATGCAA
CAGCAGATGCATAAGCAACTACAAATTCAGATTCCGACCTGTGACCGATTTTATCAATGTT
CCAGCAGCACAGGACACTTTTCTGGTAGGAAAGTACCAGGTTTGTGTAATGGGCTCTCAA
CAATCTCCTCTAACAGAGTTCATGATGATGCTCGTGCTACCAAAAACGGAACTTCTTTGTC
CAGACCAAATGGCAGTCACAGATGCATGGTTTTCGGAGTAAATTTATTTAATGGCTCACCT
AAGCTCCCTTCACCACAAGTTCTCACTTCTAGTGAGGTTCAACGTCTTTGCTCGACTCCT
CTCACTTCTCAGTCGAGTGTTTCCATTGCTTCCAAGGGTATATCTAGCAAGCAATGCAACA
ACTGTTGCTCCGTCGGCGATCGAACTTGCACCAAAGTGCTCAAGTATGGAACTAATCTTG
GAAGATCGGTTGATCTCTATCGATTCAACGGATACAAAGGCCTCATCCTTGAGCTTGATCA
TATGTTTGATTTCAACGGAAAGTTGATCGATGGAAGCAGCGGCTGGCACATAACCTATACC
GATGAAGATGGGGATATGATGCTGATTGGAGATCCTTATCCATGGCAGAAATTTCAGCATG
AAGTCCGAAGGATGGTTATCCGCCCAAAGGAAGAAATCAACAGACTGAATCCGAGCTCAC
CGAGTTCAGCATCTTACTGA

>GhARF24-4
ATGGATGGTGAAGGGCATGGTTCAAAAGCTAAGTTCCCTTTTCACTCCACTAGAGGAGAA
AACAATGATCTCTATAGTAAACTATGGCATGCATGCGCGGGTCCTTCTGTTTATGTTCCTCG
CTCTGGAGATAAAGTTTTGTACTTCCCTCAAGGTCACATGGAACAGATTAAGCCATTGGTG
CAGGTTGAGGCATACATGAGTGAAGATGGCACTATGGAAATGCCCATCTACAATTTACCTT
GGAAGATCCTCTGCAGGGTTTTGCATGTTGAGCTTAAGGTTGAACCTGACACAGATGAGA
TCTTTGCAGAAATTATTTTGCTTCCAGAGGCAGAGCAAGATGAGCAAAGCATGGAGCATA
GATATTATCGAGCGTCGCCTCGGGAAAATTATTCTCGTTACTTTAGTAAGAAGCTAACTCCA
TCGGATATAAAGACACACGGTGGATTCTCTATCCCAAAGCGGCATGCCAATGATGGGTGT
CTTCCGCTCTTGGACATGTCTCAGGAAATCCCCCAGCAGGAACTGCTCGCAACTGACTT
GCATGGTCATCCATGGTACTTTCGACATGTTTTTCGTGGCTATCCAAAAAGAAATTTGCTTA
CCACCGGTTGGAGTACCTTTGTCACCTCGAAGAAGCTTGCTGCTGGGGATTCATTAATCT

TTCTAAGAGGGGAAAATGGAGAGTTCGGTGTTGGAGTTCGCCGATCAATGACAAAGCTAC
TGAACAGTCCATCTCCATCGATCATATCCGCTCACAGTGTGCGACATGGAATACTTGCCAG
TGCTTTCCATGCCTTTGCAACCAGAAGCATCTTTAACGTCTACTACCGTCCTTGGACAAGG
TCTTCTGAATTTATCACTCCACTTGATCAGTATATAAAGGCGGTTCAATTCGACTACTGCTT
CGGGACAAGATGTAGAATGCGAGTTGAAGGTGGAGAATCTGGGGAACAGAGATCCCTTG
GCACTATCATTGGCACTGAAGATCTCGATCCTATTAGATGGCAGAATTCCAAATGGAGATG
TGTGAAGGTGAAATGGGATCCCGCAGCGAGTTCGGTTTTGCTTCCCGAAAGAGTTTGTC
CTTGGAGCATCGATCTCACGGAATTCACCAAGAAAAAGAAAACTTCCACTCTGCATCATCA
GAAGAGGGCTCGCCCCAACAACGCATCATCCCCTGAGTTTTCTTGCTTGCTTATGGATGG
CATGTTGCATGGTACAGCTAAAAATCAATCTCAAAGTAGTTCAGGGGTCTTGCAAGGTCAA
GAAGACAGTGACACATGCGTGAATCAATCCAGTGTACTGCGACAATCATTACCGCATCTTC
TTCCGCAAGATCCTGGTTGTGCCTCAATGCAACAGCAGATGCATAAGCAACTACAAATTCA
GATTCCGACCTGTGACACATTTTATCAATGTTCCAGCAGCACAGCACACTTTTCTGGTAGG
AAAGTACCCGGTTTGTGTAATGGGCTCTCAGCATTCTCCTCTAACAGAGTTCATGATGATG
CTCGTGCTACCAAAAACGGAACTTCTTTGTCCAGACCAAATGGCAGTCACAGATGCATGG
TTTTTGGAGTAAATTTATTTAATGGCTCACCCGAGCTCCCTTCACCACAAGTTCTCACTTCT
AGTGAGGTTCAATGTCTTTGTTCGACTCCTCTCACTTCTCAGTCGAGTGTTTCCATTGCTT
CCAAGGGTATATCTAGCAAGCAATGCAACAACTGTTGCTCCATCGGCGATCAAACTTGCA
CCAAAGTGCTCAAGTATGGAACTAATCTTGGAAGATCGGTTGATCTCTATCGATTCAATGG
ATACAAAGGCCTCATCCTTGAGCTTGATCATATGTTTGATTTCAATGGAAAGTTGATCGATG
GAAGCAGCGGCTGGCACATAACCTATACCGATGAAGATGGGGATATGATGCTGATTGGAG
ATCCTTATCCATGGCAGAAATTTCAGCATGAAGTCCGAAGGATGGTTATCCACCCAAAGGA
AGAAATCAACAGGCTGAATCCGAGCTCACCGAGTTCAGCATCTTACTGA

>GaARF1-1
ATGGCTTTTCCGGCATCAGAAATTCCATCTGCTGAACAACAAGCAGATGATCCTTTATATC
GTGAACTATGGCATGCCTGTGCTGGACCTCTTGTCACACTTCCTCGTGTAGGGGAGCGT
GTTTATTACTTCCCACAAGGTCACATGGAACAACTTGAGGCATCAATGCATCAAGGGTTAG
AACACCAAATGCCTTCATTCGATCTGCCATCTAAAATACTTTGCAAAGTGGCTTCTGTTCA
GCGTAAGGCTGAACCTGATACAGATGAAGTTTATGCCCAAATAACCCTGGTACCTGAAGTA
GATCAAAGTGAGGTTATGAGCCCAGATGATCCACTTCAAGAACCTGAAAGGTGCATAGTC
CATTCATTTTGCAAGACTCTTACTGCTTCCGACACAAGCACCCATGGTGGATTCTCAGTTT
TGCGCCGGCATGCAGATGATTGTCTGCCCCCGCTGGTTGCCTTAGTGAGAGCTGGAAAT
CTTCAATCATCTAAGCTGGGTGCTAATGGGGAGCTGCGTGTTGGAGTGAGGAGGCTTATG
AGACAACAGGCAAATATGCCTTCTTCTGTTATATCTAGTCATAGCATGCATCTTGGGGTGCT
TGCCACTGCATCTCATGCCCTTTCTACGCGAAGTATGTTTTCCATCTTCTACAAGCCCAGA
ACAAGTTTGTCTGAGTTCATAGTGAGTGTAAACAAATTTCTTGAAGCTCAAAGCCATAAGC
TATCAGTTGGGATGAGGTTCAAAATGAGATTTGAGGGTGAAGAGGTACCTGAAAGAAGAT
TCAGTGGCACAATTGTTGGTGTTGAGGATAATAAATCATCTGCATGGGCTGATTCTGAGTG
GAGATCTCTCAAGGTTCAATGGGATGAACCTTCATCCATCATACGTCCTGATAGGGTGTCC
CCATGGGAATTAGAGCCTCTTGTTGCAACTAGTAACTCTTCCATCTCACAACCTGCACAAA
GGAACAAGCGGGCTAGGCCACCCGTTCTACCTTCACCATCTTCAGATCTTTCTTCACTTG
GTATATGGAAATCACCAGTTGAATCTCCTTTCTCGTATTGTGATGCACAACGTGGGCACCC
ATCACCTAAATTATCCTCCACTGCAAAGCCTAATTCTGTTGGCTTTAGTAGGAATAGCTCCC
TGGCTGCAGTTTCTAGCAGCTCAATGTATTGGCCTAACCGAGTTGAGAATGTTACAGAATC
TGTTGCACCAGTTGTGAACAAAGAATCTAGTGAAAGAAAGCAGGGAACTGGGAATGGCT
GCAGACTTTTTGGTATTCAGTTACTTGACAAAATAAACATGGAAGAAAATTCACCTGTGGC
TACAATTTCTGGGACTGGTGTGGATGACCAGCCACTTCATTCACTAGATGCTAATTCTGAC
CAGCAATCTGATCCATCAAATCTTAATCAGTCTGATCTTCCTTCTATAAGTTGTGAACCTGA
GAAGTGCCTGAGATCTCCTCAGGAGTCACAGAGCAAGCAAATTCGGAGCTGCACAAAGG
TTCACATGCAAGGTATGGCAGTTGGAAGGGCTGTTGATTTGACACGATTCGACTGTTATG
AGGATTTGCTAAAGAAGCTGGAATACATGTTTGACATTAAAGGTCAGCTTTGTGGATCAAC
AAAGAATTGGCAAGTTGTCTATACCGATGATGAAGATGACATGATGATGGTTGGGGATGAT
CCCTGGAATGAGTTTTGCAGCATGGTGAGGAAAATTTTTATCTATACATCAGAGGAAGTCC
GGAAGTTATCACCTAAGATAAAACTTCCGGTGAATGATGATGATGATGATAGCAAAGCAAC
CAAGGCTGGGGTTGACACAGTTATCAACCCTGAAGATCGTTCATCGATCGTTGGTCAAGG
GTGCTAA

>GaARF2-1

ATGACGAATACGGAGGTAGCTATGAAAGGGAATTGTGTGAACGGAAGAGGAGGAGAGAG
CTTTTCTTCTGGTTATAGTGAGCCAAATGATGGTAGGAATACCGTGGAAGGGCAAAATGGT
CATTCGACTCGTCAAGCTCCGGCTACAGACCCCGAAACGGCGTTGTATAATGAACTATGG
CATGCATGTGCTGGACCTTTGGTCACTGTTCCTCGTGAACAAGATCGTGTGTTTTACTTTC
CTCAAGGTCACATAGAACAGGTTGAGGCGTCTACTAGTCAAGTAGCAGACGAACAGATGC
CAGTGTATAATCTTCCATCAAAGATCCTTTGTCGTGTGATTAACGTTCAGTTAAAGGCTGAA
CCAGATACAGATGAAGTCTTTGCTCAAGTGACTTTACTTCCTGAGCCTAATCAAGATGAGA
ACACTGTGAACAAGGAGCCTCCTGCGCCTCAACCACCACGGTTCCATGTGCATTCCTTTT
GCAAGACCCTCACTGCGTCAGATACAAGCACCCATGGTGGGTTTTCGGTGCTCAGGCGG
CATGCAGATGAATGTCTTCCACCACTGGACATGTCACGGCAACCTCCAACCCAGGAGTT
GGTTGCTAAGGATTTGCATGGAAATGAGTGGCGATTCCGGCATATTTTCAGGGGTCAACC
ACGAAGGCACTTGCTTCAAAGTGGATGGAGTGTTTTTGTTAGCTCCAAGAGGCTTGTTGC
AGGGGATGCTTTTATATTTTTAAGAGGCGAGAATGGAGAATTACGTGTTGGTGTACGACGT
GCAATGAGGCAGCAGGGCAATGTTCCTTCATCAGTAATATCAAGTCACAGCATGCATCTTG
GTGTGCTTGCAACAGCATGGCATGCCTACATGACAAAAACAATATTCACTGTGTACTACAA
ACCTAGGACAAGTCCTGCTGAGTTTATTGTTCCTTTTGATCAGTACATGGAGTCAATGAAG
AATAATTACTCCATAGGGATGAGGTTCAAAATGAGATTTGAAGGTGAAGAGGCTCCCGAAC
AGAGGTTTACTGGAACAATAGTTGGAATTGAAGATGCTGATCCTAAAAAGTGGCAGGATTC
CAAATGGAGATGCCTCAAGGTGCGATGGGATGAGACTTCTACCATACCTCGTCCAGAGAG
AGTTTCTCCCTGGAAAATTGAACCTGCTTTGGCTCCTCCTGCGCTGAATCCCCTTCCAAT
GCCCAGGCCAAAAAGGCCCCGATCTAATGCAGTCCCTTCATCTCCTGATTCCTCTGTACT
TACTAGGGAAGGTTCATCAAAAGCTATTGTAGACCCTTCACCAGCTACTGGGTTTTCAAGG
GTCTTGCAAGGTCAAGAATTCTCGACCTTGCGAGGCAACTTTGCTGAGAGTCATGAGTCT
GACACTGCTGAAAAGTCGGTGATATGGCGACCTACGGTAGATGACGAGAAGATTGATGTG
GTTCACACTTCAAGAAGATTTGGGTCAGAGAATTGGATGTCTTCTGGGAGGCACGAACCA
GCAGCATACACAGATCTGCTCTCAGGTTTTAGGTCAAATGCTGATTCCTCCCTTGGGTATT
GTCCACCGATGGTTGATCAAACTTCATTAGCCGGTAATCCAATGAGAAGACAATTACTAGA
TCAAGAAGGGAAGCTTGGCTCTTGGTCCCTCATGTCCTCTGGTCTCTCACTCAAGTTGGT
TGACAGTAATGCTAAGCCTTCTGTGCAAGGTTCTGAGGTTCCTTATCAAGCTCGAGGAAA
TGGTAGATTTAGTGGTTTTGGTGAGTATCCTGTGCTTCAAGGTCATAGGATCGAGCACCCA
CATGGAAACTGGTTGATGCCTCCCCCAACGTCATCTAATTACGAGAATCCTATCCAATCAA
GAGATTTAATGCCCAAAGCTTCATTGGGACAAGATCATGAGAACGGAAAATCTAGAGAAG
GAAGTTGCAAGCTCTTTGGTATTCCTCTCATTAGCAATTCTGTTGCATCAGAGCCTACTGT
CTCTCCTATTAATGCCACGAACAAGGCAGCAAGTCATGTGGAAGCTGCACCAAACCAAGC
TCATACATTTACGTTTGATCAAAAGTCTGAGCAGCCAAAATTCTCACGGTTGGCAGAGAAT
CTGTCTATTTTTAATGAGCAGGAGAAATCATTTCAGCTGGGTCAGCCTCATACAAGAGAGG
TTCAAAGCAAATCTCCTAGTGCTTCAACTAGGAGTTGTACAAAGGTCCTAATGCAAGGGA
GTGCTCTTGGTAGGTCCGTGGACCTTACTAAGTTCAACAACTACGATGAACTGATTGCTG
AATTGGATCAATTATTTGAATTTGGTGGTGAATTAATGGCCCCTAAAAAGAATTGGCTTGTC
GTTTATACCGATGATGAGGGTGATATGATGCTTGTTGGAGATGATCCCTGGCAGGAATTTT
GCTCCATGGTTCGCAAGATCGGTATCTACACTAGGGAAGAGGTTCAGAAGATGAAGCCC
GGGTCATTGAATTCAAAGGGCGAGGACAATCCGGTTTCTGTGGAAGGCCCAGATGCAAA
AGACGTGAAATGTACACCAGCATCTAGTACAGAGAATTGTTAA

>GaARF2-2
ATGACTACGTCGGAGATATCGATAAAAGGAAATTGTGTCAACGGAAGAGGAGATAGTTTTT
CTTCCGGTTATACTGAGCCACGAGATACTAGGAACACCATGGAAGGGCAGAACGGTCATT
CCGCTCGTACAGCTGCCGTCAGAGAAACCGTAGACCCCGAAAGGGCGCTGTATACGGAG
CTATGGCATGCATGTGCTGGACCTCTGGTGACGGTCCCTCGCGAAGGAGAGCGCGTGTT
CTACTTTCCTCAAGGTCACATAGAACAGGTGGAGGCGTCTACTCATCAGGTATCAGAACA
GCAGATGCCGGTGTATGACCTTCCACCAAAGATCCTTTGTCGTGTGATTAACGTACAATTA
AAGGCTGAACCGGATACTGATGAGGTTTTTGCTCAAGTGACTTTGCTTCCTGAACATACTC
AAGATGAGAACATGGTGGACAAGGAGCCTCCCATTCTTGAACCCCCACGGTTCCAAGTG
CATTCGTTTTGCAAAACCCTGACTGCTTCAGATACGAGTACCCATGGTGGATTTTCAGTGC
TCAGGCGGCATGCTGATGAATGTCTTCCACCACTGGATATGTCGCTGCAACCTCCAACAC
AGGAGCTGGTTTCTAAGGATTTGCATGGAAATGAGTGGCGATTCCGGCATATCTTCAGGG
GTCAGCCACGAAGACACTTGCTTCAAAGCGGTTGGAGTGTTTTTGTTAGCTCCAAGAAG
CTTGTTGCTGGGGATGCATTTATATTTTTAAGAGGCGAGAATGGAGAATTGCGCGTTGGTG
TACGGCGAGCATTGAGACAGCAGGGCAATGTTCCTTCATCGGTTATATCAAGTCATAGCAT
GCATCTTGGTGTGCTAGCGACAGCATGGCATGCCTACACTACCAGAACCATATTCACTGT

GTATTACAAACCCAGAACAAGTCCAGCTGAGTTCATTGTTCCATTTAATCAGTACATGGAG
TCTGTAAAGAACAATTACTCAATAGGGATGAGGTTCAAAATGAGATTTGAAGGTGAAGAAG
CTCCTGAACAGAGGTTTACTGGAACAATAGTTGGAATCGAAGATGCTGATCCAAAGAGGT
GGCAGGGTTCCAAATGGAGATGCCTGAAGGTGCGATGGGATGAAACGTCTACAATACCT
CGTCCCGAGAGAGTTTCTCCTTGGAAAATTGAACATGCTTTGTCTCCTCCTGCCCTTAATC
CCCTTCCAATGCCCCGGCCAAAAAGGCCTCGAACTAATGCTGTATCTTCATCCCCTGATT
CCTCTGTACTTACTAGGGAAGGTTCTTCCAAAGTTACTGTAGACCCTTTGCCGGCCAGTT
CATTTTCAAGGGTCTTGCAAGGTCAAGAATTCTCGACCTTGAGAGGCACATTTGCTGAGA
GTAATGATTCTGACACTGCTGATAGGTCAGTGATGTGGCCACCTTCAATAGATGATGAGAA
GATTGATGTAGCTCATGGTGAAAGAAAATTTGGGTCAGAGAATTGGATGCCCTCTAGGAG
GCATGAACCAACTTACACAGATTTGCTCTCAGGTTTTGGGTCGAATGCTGATACATCACGT
GGATATTATCCATCCGTTGTTGATCAAACTTCAGTAGCTGGTAATTCGGGGAAAAAACAATT
ACTAGATCAAGAAGGGAAGCTTGGCGCTTGGTCCCTCCTGCCATCTGGTCTCTCACTCAA
GTTGTCTGACAGTAGTACAGACCCTCCTTTGCAAGGTTCGGATGTGCCTTATCAGGCACG
GGGAAATGGTAGATTTTGTGGTTTTGGTGACTACCCTATACTTGAAGGTCGTAGGATTGAA
TGCACACGTGGTAATTGGTTGATGCCTCCCCCATCCACTTCTTGTTATGATAATTCAGTCC
ATTCAAGAGATTTAATGCCGAAAACATCATTGGTTCAAGAGCATAAGAATGGAAAATCTAGA
GAAGGAAACTGCAAGCTCTTTGGTATTCCTCTCATAAGTATCTCTAGCGCTTCAGGGCCT
GCAGTCTCCCATATTAGTGCTTTCGCCAAGCCTGTAGGACATATGCAAGCTGCATTGCAC
CAAATTCATGCACTTAAATCTGATAAAAGGTCTGAAAATTCAAACGCCTCCCAGATGGCAG
AGGATGTTTCTGCTTTTAATGAGCAGGAGAAAATGGGTCAGCCCCATGCACGGGAGTTTC
AAAGCAAACCGTCTACTGCTTCAACTAGGAGTTGTACTAAGGTTCTTATGCAGGGGACTG
CTCTTGGAAGGTCTGTGGACCTTACTAAGTTCAACAACTATGATGAGTTGATCGCTGAATT
GGATCAATTATTTGAGTTTGGAGGTGAATTAATGGCCCCTCAAAAGAACTGGCTTGTTGTT
TATACTGATGATGAGGGTGATATGATGCTTGTTGGCGATGATCCTTGGCAGGAATTTTGTG
CCATGGTCCGCAAGATTGGTATCTACACTAGGGAAGAGGTCCAGAAGATGAAGCCAGGG
TCGTTGGGTTCAAAGTTTGAGGACATTCCAGTTCCTTCAGAGGGTACAGTTGCAAAAGAA
GTAAACTGTCCATCAGCATCTAGTGCAAAGAATTGTTCAGGATTTTTGATGGAGACATGGG
GAGTGGCATGGTAA

>GaARF2-3
ATGAAGGCTCCACCAACTGGATTTTTGGCAAATCCTGCTGAAGCAGGAGATAGGAAGAGT
ATCAATTCAGAATTATGGCATGCTTGTGCTGGACCACTGGTTTCTTTGCCACCAATTGGAA
GTCTGGTGGTTTATTTTCCTCAAGGCCACAGCGAACAAGTTGCAGCATCAATGCAGAAGG
AGACTGATTTCATACCAAGTTACCCTAACCTTCCTTCCAAGTTGATTTGTGTCCTCCATAAT
GTTACATTGCATGTAAGAATGAGCATACCACCATTATCACAAGCTAACTTTGCTGATCCAGA
AACTGATGAGGTCTATGCCCAGATGACTCTTCAACCTGTAAACAAATATGACAAGGAAGCA
TTACTGGCATCTGATATGGGCCTCAAGCAAAGCAGGCAACCTGCTGAGTTCTTTTGCAAG
ACTCTTACAGCTAGTGATACTAGCACTCATGGTGGATTTTCAGTCCCTCGTCGAGCAGCT
GAGAAGATCTTCCCTTCTCTGGATTTTTCCATGCAACCACCTGCTCAAGAGCTTGTAGCA
AGAGATTTGCATGAAAATGCATGGACTTTTAGACATATTTATCGAGGTATTGTTCTAGATATT
GCTCTCTTTTCATTTCTCAAGCAGAGTCAACCAAAGAGGCACCTTCTGACTACCGGTTGG
AGTGTCTTTGTTAGCACAAAAAGACTCTTCGCTGGTGATTCTGTTCTTTTCATAAGAGATG
CGAAGTCACAGCTTCTCTTGGGTCTAAGGCGCGCTAATAGACAACAGCCAGCTCTCTCAT
CATCAGTGATTTCTAGTGATAGCATGCATATAGGGATCCTTGCTGCTGCAGCCCATGCTGC
TGCAAATTTCAGCCCATTTACTATATTCTACAATCCAAGGACTCAAAAGTTTTATAATTTTGA
TAGGGCAAGCCCCTCTGAGTTTGTGGTACCCTTAGCAAAATATAACAAAACCATGTACACC
CAAGTTTCTCTTGGCATGCGGTTTAGAATGATGTTTGAGACTGAGGAGTCTGGAGTACGA
AGATACATGGGTACAGTTACTGGTATCAGCGACCTGGATCCTGTGCGATGGAAAAACTCA
CAATGGCGCAATCTTCAGGTTGGTTGGGATGAATCAACAGCTGGAGAACGGCCCAGGCG
AGTTTCAGTTTGGGAAATTGAGCCTGTTGTATCTCCTTTCTTCATATGTCCACCTCCTTTTT
TCAGACCTAGGTTTCCGAAGCAACCAGGGATGCCAGATGATGATTCTGATGTCGAGAACG
CTTTCAAAAGAGCTATGCCTTGGCTTGGAGATGATTTTGGTATGAGAGGTACCCCTAGTTC
AATCTTCCCTGGTTTGAGTTTAGTTCAGTGGATGAATATGCAACAAAGTAATCAGTTTCCA
GCTGCTCAATCAGGATTCTTTCCATCAACGGTTTCTTCGAATCTGCTGCATAGTAACCTTA
GCATTGATGATCCTTCCAAATTATTGAATTTTCAAGCTCCTGTATTACCTGCACCGAATATG
CAATTTAATAAAGCTAACCCAAACCAAGTCAACCAGTTGCCTCAGGCACCTACGACTTGGT
CCCAGCAGATATTGCAGACTCCGGTAAATCAACATCAGCAGCAGCAACCCCAACAGCAAT
TGCAGCAACAACAGCCACAACAACCACAGCAGCAGCCACAGCCACAGTCACATCTTCTT
CATCAACAGCAGCCTCAGCCACAGCCATCTCCAAAGCAACAGCAACACATCCACCAGGA

ACAGAGACAACAGCCACAACAGCAGCAGCCGCTGCAACAACAACCAACCCTACCGCCTC
AAGTAACTAATGGCATTGTTGCTCCTAACCAGATCTCAAATCAAAATTTGCATCAGCCAGC
TGTTTACTCTCACCTGCAGCAGCAACAATTGTTGACAAGCAATAGCCTGTCTACCCAAACT
ACCCTCTCTGCTCATATGACTTCATATCCTTTGACGTCATTACCACAAGATACACGGGTTCA
GCAGCAGATGGAACAGCAACCTAACCTCATGCAGAGGCAGCAGCAACAGACACAACTGC
AACAAAGCCTGTCCCAGAGGACACAGCAGCAGCCGCAGATTCTGCAACTATCACAGCAG
GGCCTCTCGGAGCAACTGCAATTACAACTTCTCCAAAAATTGCAGCAGCAGCAGCAGCA
GTCGGCTCAACAATTACTCTCCGCAGCTGGGTCACTGCTGCAGCCTCCAATGTTGCAGC
AACAGCAAACTCATCAACAGAACCAACCATTGCAGCAGTTGCCTCTTTCTCAGAGCCAGG
TGCAACCACTGGGTAGCAATGGCTTCTCAACATCGATGTTTGTGCAACCTCAACAACTTT
CAGTGCATCAATCCCAAAGTCAGAGCAAACAACTTATGGCAATGAGAAGCAATTCTGGTC
TTATTGATGGAGATGCTCCACCTTCAACCTTATCTTCTACCAATAATTGTCAGGTTTCCCCA
TCAAACCTTATAAACAGAAGTCACCATGTACCATCCATATTGATGACAGATCCAGTTGTTGA
GCCTCCAAGTACACTAGCTCAAGAGCTCCTGAGCAAGCCTGATATTCAAATCAAACATGA
GCCGCCCACCTCTAGAGGACTAGACCAATCAAAGTACAAAAGTTCTGTAACAGACCAATT
AGAAGCATCCTCTTCTGGAACATCATATTGCTTGGACGCAGGCACCATCCAGCATAATGC
CTCCCTTTCCTTTCTGGAAGGTGATGTCCAATCACATTCTCGGAACAATCTTCCTTTTACA
GCTAATATTGATGGATTGGCACCTGAGACTTTGTTAACAAGGGAATATGACTCTCAAAAGG
ATCTTCAAAACATGCTTTCTAATTATGGTGGGAACCCAAGAGATATTGACACTGAGTTGTCT
ACTGCTGCAATAAGCTGTCAGTCATTTGGTGTGCCAAATATACCTTTCAAGACAGGATGCT
CAAATGATGTTGCCATAAATGAGACAGGAGTTTTAAATGGCGGATTGTGGACCAACCAAAC
TCAACGCATGCGAACATATACAAAGGTGCAAAAGCGTGGTTCTGTGGGAAGATCAATTGA
TGTGACCCGCTATAAAGGGTATGATGAACTACGGCATGATCTAGTCCGTATGTTCGGTATC
GAGGGGCAGCTGGAGGATCCACAAAGTTCTGACTGGAAATTAGTTTATGTGGATCATGAA
AATGACATATTACTTGTTGGTGACGATCCTTGGGAAGAATTTGTAAGTTGTGTTCAGAGCA
TAAAGATACTGTCGTCAGCAGAAGTACAGCAGATGAGCTTGGATGGTAATCTTGGAAATGT
GTCAGTTCCCAATCAAGCTTGCAGTGGGACTGAAAACGGAAATGCATGGAGAGGACATTA
TGATGATACCTCAGCAGTCTCATTTAACAGA

>GaARF3-2
ATGGGGGGATTAATCGATCTGAACTCTACGGAAGACGATGAAACGCCATTATCTGGTTCTT
TGTCTCCATCTTCATCTTCAGCTTCCGTGTTAAGTGCCCCTGGTTCTGGTTCTTCTGTTTG
TTTAGAACTTTGGCATGCGTGTGCTGGTCCACTTATATCTTTGCCAAAGAGAGGAAATGTA
GTGGTGTACTTCCCTCAAGGCCACTTGGAACAAGTTTCCGATTTTTCCGGTGTAGCTGCA
GCTTATGATCTCCCTCCCCACGTGTTTTGTCTGGTTGTTGATGTCAAGCTCCATGCTGAG
GGTGCCACAGATGAGGTTTACGCCCAAGTTTCATTGGTTCCTGAAACGGAGCAATCTGAG
CAGAAGTTGGAGGGAGGGAAGACTGAGGCAGATGGTGAAGAGGAGGATACTGAAACCA
ATATCAAGTCAACCACGCCCCATATGTTCTGCAAGACACTAACCGCTTCCGATACCAGCAC
GCATGGTGGCTTCTCTGTTCCTCGTCGAGCTGCCGAGGACTGCTTTCCTCCCTTGGACT
ATAATCAGCAAAGGCCCTCACAAGAGCTTGTTGCGAAAGACCTGCATGGTTCTGAATGGA
GATTTCGACACATCTATAGGGGTCAACCACGGAGACATTTGCTGACTTCCGGATGGAGTG
CATTTGTAAATAAGAAGAAGCTTGTCTCTGGAGATGCGGTGCTTTTTCTGAGGGGTGAAG
GTGGAGAACTGAGGCTTGGAATCCGAAGAGCTGCTCAAATTAAAGATGGCTCTTCTTTTC
CATCATCTTGCAGCCAGCAGTTGAATTGCAGCAATTTTGCAGATGTGGTTCATGCTATTTC
TATGAAAAGTGTATTCAGCATTTACTACAATCCAAGGGCCAGTTCATCGGACTTCATAATAC
CCGTGCATAAATTCTGGAAGTGTCTTGATCCCTCATTTTCTATTGGAATGAGGTTCAAAATG
CAATTTGAAGCTGAAGATGCAGCAGAAAGAAGACACTCAGGAGTAATAACTGGAATTAGT
GATATAAATCCTGTTCAATGGCCTGGTTCAAAATGGAGATGCCTGATGGTAAGGTGGGATG
ATATTGATGCCAACAGGCATAGTAGGGTTTCTCCCTGGGAAATTGAGCCATCTGGTTCAGT
TTCCGGTTCTAACAGCTTGATCTCTCCTGGTTCAAAAAGGAACCGAGTTGGATTTCCTTCA
GGAAATTCTGAATTTATGGTTCCTGATGGAATTAGAGCATCAGACTTTGGGGAGTCTTTGT
GGTCCCAGGTATTGCAAGTTCAAGAAAATCTGGGTTTTAACTCTCTTTATGATGGTTCTGAT
AGTCCGAATATGCATTGGTCTGAAATAAGGCGTTGCATTCCTGGTTCTATTGGTTCTGATTT
TTCTGCAATAGGAAATATTGGTAGAGACTCACTGGTGAGTCCTGATATTTCCCGTAAAAGT
GTAGGCTTTGGGGAATCTTTCCGATTCCATAAGGTCTTGCAAGGTCAAGAAATTTTTGTGT
CCCCTCCATATAGAAATGGTTCAACTGCAGATGAAACTCAAGAAAATGACGCTTTTGGTCT
CGCTGATGTTGGTCAGCTGTCGGGAACTAGAAGTGGATGGTCTTCCTTGATGCAGAGGTA
TAATACTCATAGTCGTACACGACCATCTGCACCATCTACACAAACGTCCTCACCATCTTCA
GTGTTAACGTTCCTACAAGTGAGCAATCCAATTCTGAATTTCAGTCCTATTTATAATTCTAAT
AACCAAAAAAGGGAACAGGGAGTTAACAAACAAAGTTCTTTTCATGCACCTGAAATATACA

AGGGAAAGCTATTTCCATCTTCATCCAGTGAACATGATTCCCGTGCGAGGGATCTTGGAA
GCACAGATTTATTCGGTCATTCTATTGGTTCTGTTCAACTTGGTTTTGCTCCACCTCTAGCA
GCTCAACCAGTATTCAGGACTAGTCAAGAATTAGATTCCTCCTGTAAAAGTAGCTGCAGAC
TTTTTGGTTTCTCCTTGACTGAGGGAAGACATGATGCTAGCAAGGAAGAAGACGTGGTAC
AAGCAACCTCATCATTGGCGCCTGGAGCGATTTTACCTTGTGTTAGGGAAGAGTTTCACC
CAAAGCCTTCGTCGGTGACGGACACAGTTGGAAGCAATTATACTGAAGTAAGCAATCTCT
ATGCTGTCAGAGATATGGTTTTAGATATTGCATTGTAG

>GaARF3-3
TTAATCGATCTGAACACAACGGAAGACGAGGAAACGCCGTCATATGGTTCTTTATCCCCTT
CTTCATCTTCAGCTTCCGTGTTAAGTGCTTCTGGTTCTGCTTCAAGTTCTCCCGTTTGTTT
AGAGCTCTGGCATGCGTGTGCCGGTCCACTTATATCTTTGCCCAAGAGAGGAAGTGTAGT
GGTTTACTTCCCTCAGGGCCACTTGGAACAAGTGTCCGATTTTTCCGGCGTAGCTCCAGC
ATATGATCTCCCTCCTCACGTGTTTTGTCGGGTTGTTGATGTCAAGCTCCATGCTGAGGG
TGCCACAGATGAGGTTTACGCTCAAGTTTCACTTGTTCCTGAAAATGAGCAAATTGAGCA
GAAGTTGAAAGAAGGGAACATAGAAGTAGATGGTGAAGAGGATGCCGAAGCAGATATCAA
GTCCACCACGCCCCACATGTTCTGCAAGACTCTTACGGCTTCTGATACCAGTACACATGG
AGGCTTCTCTGTTCCTCGTCGAGCTGCTGAGGACTGCTTCCCTCCCTTGGATTATAATCA
GCAGCGGCCCTCCCAAGAGCTTGTTGCTAAAGACCTGCATGGCCTGGAATGGAGATTTC
GACACATCTATAGGGGACAACCACGGAGGCATTTGCTTACAACTGGATGGAGTGCTTTTG
TAAATAAGAAGAAGCTTGTCTCTGGAGACGCTGTGCTTTTTCTTAGGGGTGAGAATGGAG
AACTGAGGCTGGGAATCCGAAGAGCTGCTCACATTAAAAATGGCACTTCTTTTCATTCTTT
GTGCACCCAGCAGTTGAACCGCAGCAATTTCGCAGATGTGGTCCATGCTATATCTATGAA
AAGTGTGTTCAGCATTTACTACAATCCAAGGGCCAGTTCATCAGAGTTCATAATACCAGTG
CATAAGTTCTGGAAGAGTCTTGATCATTCTTTTTCTGTTGGAATGAGATTTAAGATGCGGTT
TGAATCTGAAGATGCAGCAGAAGGAAGATACACAGGAGTTGTAACCGGAATTAGTGAGAT
GGATCCTGTTAGATGGTCTGGTTCAAAATGGAGATGCCTGCTGGTAAGGTGGGATGATAT
TGAAACCAATAGGCATATTAGGGTCTCTCCCTGGGAAATTGAACCATCCAGTTCAATATCTA
GTTCTAACAGCTTGCTCTCTCCTGGTTCTAAAAGGAACAGGGTTGGACTGCCTTCAGGGA
AACCTGAATTTATGGTTCCTGAAGGAATTGGAGCATCAGACTTTGGGGAATCTTTGCGGTT
CCAGAAGGTCTTGCAAGGTCAAGAAATTTTGGGTTTTAACACTCATAATGATGGTGCTAAT
AGTCAGATAATGCACCGGTCTGATATAAGTCGGCGCTTTCCTTGCTCTAATGGTTCTGGTA
TTGCTGCTATACGAAATATTGGTAGAGACACATTGGTGAATCCTGATATTTCTTATAAGGGT
GTAGGCTTTGAGGAATCTTTCAGATTCCAAAAGGTCTTGCAAGGTCAAGAAACTTTTGTAA
GCCCTCCATGTAGAAGAGGTCCAACTGTAGATGACACTCGAGAAAGTGACAGTCCTGGT
GCCCCTGATGTTGGTCAGTTGTCGGGAACTAGAAGTGGATGGTCTTCTTTGATGCAGAG
CTATAATACTCATAGTCGTATAGGACCATCTGCACAAGTGTCCTCACCGTCTTCAGTGCTAA
AGTTCCAACATGCAAGCAATCCATTTGCAAACGTCAATCCTATTCATAACTTGAATAGCCAG
GAAAAGGAGCAAAGAGTTCATAAAAGTAGTTCTTTTCATGCTCCCGAAACATATGGGGAC
GGGATACCATCTTCAACTGGTGGACATGGTTCCCGTAGGAGGACAAGTCAAGAATTAGCT
GCTTCATGTAAAAGTAGCTGCAGGCTTTTTGGTTTCTCCTTGACTGAGGGAGGACATGAT
GCTGCCAAGGAGGACAACATGGTGCAAGCAACCTCGTCATTGGGTGCTGGAGCTTTCTT
ACCTCGCATTGGGGAACAGTTTAACACACAACCTCCTGCAGTGACAAACACAGTTGGAA
GCAGTTATACCAAAGTAAGCAACCTCTACGCTGTCAGAGATATTGTTTATGATATTGCATTA

>GaARF3-4
ATGGGGTGTTTAATCGATCTGAACACTACTGAAGAAGATGAAACGCCGTCGTCTTGTTCTT
TATCACCATCTTCTGTATTGAGTGCTTCTGGTTTTAGTTCTTCAGTTTGTTTAGAGCTTTTG
CATGCTTGTGTTGGTCCACTTATATCTTTACCAAAGAAAGGAAGTGTAGTGGTGTACTTCC
CTCAAGGCCACTTGGAACAAGCTCCCGAATTTTCCGGCTTAGCTTCAGTTTATGATCTTCC
TCCTCATGTGTTTTGTCGGGTTCTTGATGTTAAGCTCCATGTAAGCTACTTATATACATGTTT
TTTTCAAAGCTGTCGCTTTTATTTTGTTTGTAAGAGCTATAAAGCTGAGGGTGCTACAGATG
AGGTTTATGCTCAAGTTTCACTGGTTCCTGATAATGAGCAAACTGAGCAGAATTTGCAAGA
GGTAGATGGTGACGATGAGGATGCCGAAGCAGATATGAAGTTAGCCACGCCGCACATGTT
CTGTAAGACCCTTACGGCTTCCGATACCAGTACTCATGGTGGTTTCTCCGTTCCACGTCG
AGCTGCGGAGGACTGCTTCGCTCCCTTGGATTATGATCAGCAGAGGCCATCACAAGAGC
TCGTTGCAAAAGATCTACACGGTGTAGAATGGAGATTTCGACACATTTATAGAGGGCAACC
ACGGAGGCATTTGCTCACTACTGGGTGGAGTGCTTTCGTAAACAAGAAGAAACTCGTATC
CGGAGATGCTGTGCTTTTTCTTAGAGGCGAGGATGGAGAACTGAGGCTGGGAATCCGAA
GAGCTGCCCAAATTAAAAACGGGGCTTCTTTTCTTTCTTTGTGCAGCAAGCAGTTGAACC

GCAGCACTTTTGCAGATGCGGTCCATGCTATATCTATGAAAAGTGTTTTCAGCATTTACTAC
AATCCAAGGGCTAGTTCATCGGAGTTCATAGTTCCGGTCTGTAAATTCAGGAAGAGCCTT
GATCGTTCATTTTCTGTTGGAATGAGGTTCAAAATGCGGTTTGAAACTGAAAATGCACCAG
AACGAAGATCCTCGGGACTCATAACTGGAACTAGTGATTTGGATCCTGTTAGATGGCCTG
GTTCAAAATGGAAATGCTTGTCGGTGAGGTGGGATGATATTGACGCCAACAAGCACGGG
AGGGTTTCTCCCTGGGAAATTGAACTTTCCGGTTCTATTTCTAGTTCTAACTGCTTGCTCT
CACCCTGTTCGAAAAGGAACAGGGTTGGATTGCCTTCAGGAAAACCGGAATTTATGGTTC
CTGATGGAATTGGAGCACCCGACTTTGGGGAACCTTTGCGGTTCCAAAAGGTCTTGCAA
GGTCAAGAAATATTGGGTTTTAGCACTCTTTATAATGGTGCTGATAGTCACAACATGCATCG
GTCTGAAATACGACGGTGCTTTCCTGGATCTAATGGTTCTGGCATTGCTACAATTGGAAAT
GTTGGTAGAGACCCTCCGTTGAATCCCGTTATTTCGTATAAAGGTGTAGGCTTTGGGGAAT
CTTTTGGATTCCATAAGGTCTTGCAAGGTCAAGAAATTTTTCTAAGTTCTCTGTATAGAAAA
GGCTCAACTATGGAAGAGACTCGAGGAAATGACAGTGCTGGTCTCACTAATGTTGGGCAA
ATGTCGGGAACTAGAAGTGGATGGTCTTCCTCGATGCAGGGATATAATACACATAGTCCTG
TACAACCATCTGTGCAAGTTTCCTCACCGTCTTCTGTGTTGATGTTCCAACAAGCAAGCAA
TCCAGTTCCGAACTTCAATTCTACTCGTAACTTCAACCAGGAAATGGAGCGGGGAGTGAG
TTCTTTTCGTGTGCCCGAAACTTATGGAGCCAAGTTACTGTCATCGTCAATCAGCGAACAT
GATTCACAACCTCTGGCAGCTCAACCTTCTTTTGGGACCAATCAAGAATTAGCCTCCTGC
AAAAATAGCTGCAGGCTTTTTGGTTTCTCCTTAACTGAAGGAGATCTTGATACAACTAAGG
AAGACAGCGTGGTACATGCAACATTATCATTGGGTCGTGGATCGCTTTTACCTTGTATCGG
AGAAAATTTCCATCCAAATCCTCCAGCAGTGACAAGCACAGGAAGCAATTGTACTAAAAGT
AAGCAATCTCTATGCTGTCAAAGATATGCATTT

>GaARF4-1
ATGGAAATTGATCTGAACCATGCACTGAGTGAGGTGGAGAAGACTGCGGTTTGCAATGG
GGACTGTGACAAACTGAACTGTGTGTGCTCTTCATCTTCTTCAAACTTAGCTTCGCCTCCA
TGTACCTCTTCCATTTACTTGGAGCTTTGGCATGCTTGTGCTGGCCCTCTCACTTCACTCC
CCAAAAAGGGAAATCTTGTTGTTTACTTCCCTCAGGGTCACTTGGAGCAGCTGGCCTCTG
CCTCTCATTTCTCTCCTTTGGAAATCTCCACCTTTGATCTTCCCCCTCACATCTTTTGCAAA
GTGGTGAATGTCCAGCTTCTTGCCAATAAGGAGAATGATGAGGTCTATACGCAGCTCACT
TTACTTCCTCAGCCGGAGTTGAGAGGGCCTAATTTGGAGAGCAAGCAGCTGGATGAACTA
GGTGTGGATGAGGGAGATGATGGATCGCCTAAAAGATCAACCCCTCACATGTTTTGTAAG
ACGTTAACCGCATCGGACACCAGCACTCACGGAGGGTTCTCCGTTCCTCGTAGAGCTGC
AGAAGACTGTTTTCCTCCACTGGATTATAAACAGACGAGGCCGTCTCAAGAACTTGTTGC
CAAGGACCTGCACGGAGTTGAGTGGAGGGGTGAAGATGGAGAGCTCAGGTTGGGAATT
AGAAGAGCTGTACGGCCGAGGAATTGTCTTCCTGAATCAGTCATTGCTAAACAGAATTCAT
ATCCGAACGTTCTTTCTCCGGTGGCTAATGCATTATCCACGAAAAGCATGTTTCATGTCTT
CTACAGTCCAAGGGCCAGCCATGCTGAGTTTGTCATACCCTTTCGGAAGTATTTTAAAAGC
ATAGCAAATTCAGTGTGCATTGGAACAAGATTCAAAATGAGATTTGACATGGATGATTTGC
CCGAGAGAAGGTTTAGTGGTGTAGTGATGGGAATGGGGGACTCAGATCCTTACAAATGG
CCCAATTCAAGGTGGAGATGCTTAATGGTCAGGTGGGATGAAGATAATATGATCGATCGCC
ACGAGAGAGTCTCACCTTGGGAAATTGATCCTTCCGCTTCTCTCCCACCCTTGAGTATTC
AGTCTTCCCCGAGGTTGAAGAAACTGCGAACAGGTACGCAGGCTGCCGCACCCGACAC
CCTCATTACAGGGGGGAGTCGATTTTTGGACTTTGAGGAACCGTTGAGATCCTCTAAGGT
CTTGCAAGGTCAAGAAAATGTAGGTTTTGTATCACCCTTATATGGGCATGATACTGTAAGC
CGCCCGCTAGATTTTGAGATGCAATCTCCTGCACATCATCAAAGTCTTGCGTCAACAGGG
ATTGAAAAGAGCAATATTAGTGAGTTTATGAGGGTTTGCTCCACCACATACACAGGCTTTG
CGGATTCTAATAGGTTTCCGAAGGTCTTGCAAGGTCAAGAAATTTGCCAATTGAGATCCCT
GACACAAAAGGCTGATCTCAATCTCGGTGTTTGGGCGAAAACCAATGTTGGTTGCAATTC
ATTCAACATGCATCAAACACTGAAAACCAATTGCTATCCACTAGCATCGGAAGGCCTTCGA
AATATGTATTTCCCTTACAGTGAATTTCTCAAAACCGTCCAAGAGCCAACAATGAGCTCTTA
TGCATGTCCACTTCCAAGAGGTAATGTCCCGTTTAATGCTTCCTCAATTAGGACAGGGGTA
GGGGTTATCGTGGATGGGTTCAGAAAGCCGAATCAACTGAATGAGCATAAGCCATTGGAG
AATATTCCAAGTCCTGCTTCGGAAAACAATTCGAGGAACCAACAGGATGACTCCTTCAAAA
GAAATGTTGCTGGATGTAAACTCTTTGGGTTTCCTTTGAATGTGGAGTCGCCTACTCCAAA
CTCACAAAATTCTGGTAAGAGGAGTTGTACAAAGGTTCACAAGCAAGGCAGCTTGGTCG
GAAGAGCTATTGATCTCTCGAGACTGTACGGATATGATGACTTGATGATTGAACTTGAACG
TCTATTTGGAATGGAAGGCGTATTAAGTGATCCGGATAAAGGGTGGCGGGTATTGTACACA
GATAGCGAGAACGACGTAATGGTGGTTGGAGATGACCCTTGGCATGAATTTTGTGAGGTG
GTGTCGAAGATCCACATATACACCCAAGAAGAAGTGGAGAAGATGACAATAGGAACGGGG

AGCGATGACACGCAAAGCTGTTTGGAGCAAGCAGCAGTGATAATGGAAGCATCAAAGTCA
TCCTCAGTGGGGCAGCCAGATTCATCATCTCCAATGTAA

>GaARF4-3
ATGGAAATTGATCTGAACCATGCATTAAACGAGGTGGAGAAGACTGTACTTTGCAATGGCA
GCTGTGAAAAAGCTAGTGCTTGCGTTTACTGTTTGTCGTCGTCGTCGTCATCATCATCGTC
ATCATCATCTTCTTCTTCTTCTACTTCATGTACTTCAAACTCAGGTTCACCTCCTTGTTCAT
CTTCCATTGACTTGGAGCTTTGGCATGCTTGTGCTGGTCCTCTCACATCACTTCCAAAGA
AGGGAAATGTGGTTGTATACTTCCCTCAAGGTCACTTGGAACAACTTGCCCTTGCTTTTC
CTTTCTCTCCCTTGGACACCTTTGATCTTCCTTCCCAAATCTTTTGCAAAGTGATGAATGT
CCAGCTTCTGGCCAATAAGGAGAATGATGAGGTCTATACGCAAGTTACTTTACTTCCTCAA
CCGGAGTTGAGAGGGCATAATTTGGGGAGCAAGCAGCTGGATGAGGTAGAGGCGGATG
AGCATGCCGATGGGTCGCCTTCGAAACCAACCTCTCACATGTTTTGCAAGACACTAACAG
CTTCTGACACTAGCATCCATGGAGGGTTCTCTGCTCCTCGTAGAGCTGCTGAAGACTGTT
TTCCTCGGCTGGATTATAAACAGACAAGGCCCTCACAGGAGCTTGTTGTTAAAGACCTCC
ATGGAGTTGAGTGGAGGTTTCGCCATATATATAGGGGTCAACCAAGGCGACATCTCCTTAC
TACCGGCTGGAGTGTTTTTGTGAGTCAAAAGAATCTTATTGCTGGGGATGCAGTGCTCTT
TCTGAGGGGCGAAGATGGGGAGCTGAGGTTGGGAATTAGAAGAGCTGTTCGACCAAGA
ACTGGTCTTCCAGATTCAATTATTGCGCAACATAATTCATATCCAAAGGTTCTTTCTGCAGT
GGCTAATGCATTATCCACCAAAAGCGTGTTTCAAGTCTGCTACTGTCCAAGGGCCAGTCA
TGCCGAGTTCGTCATACCCTTCCAAAAGTATATTAAAAGCATCACAAATCCAATGTGCACT
GGGGCAAGATTCAGAATGAAGTGTGAAATGGATGATTTGCCAGAGAGAAGGTGCAACGG
TGTAGTGACAGGAATAGGGGACTCAGATCCTTATAAATGGCCTAACTCAAAGTGGAGATG
CTTAATGGTTAGGTGGGATGATGATATCGCAAGTGATCACCAAGAGAGAGTTTCACCTTG
GGAAATTGATCCTTCTGTTTTTCCCCCACCCTTAATTATTCCATCTCCATTCAAGTTAAAGA
AACTGCGGACAGGTCTGCAGACTGCTGCACTTGACACCCCTGTCACTGGAGGGGTTGG
ATATTTGGACTTTGAGGAATCGACAAGATCCTCTAAGGTCTTGCAAGGTCAAGAAAATGTA
GGTTTCATATCACCTGTATATGGATGTGGTACAGTGAACCATCCCCTCCATTTCGAGATGC
AATCTATGGCACATCAAAGTCTTGCATCGACAGGACTTGGAAAGAGTAACATTAGTGATTT
TATGAGGGCTCACTCCTCTTCTCACACAGGCTTTGCGGAAACAAATAGGTTTCCGAAGGT
CTTGCAAGGTCAAGAAATTTGCTCATTGAGGTCCCTCACACACAAGGCTGATCTCAACCT
TGGTGTTTGGGCAAAAACCAATCATGGTTGCAATTCTTTCAACATGAATCAAGCACCCAAC
ACCAACTTCTATCCAGAAGGACTTCGAAATATATATTTTCCTTATAATGAGTTTTACAGAGCT
GGCCAGGAACCTAAAATGTGTTCTTATGCATCTACACTTCCAAGGGGCAATGTTTTGTTCA
ATGCTTCCTCAATTAAGCCGGGGGTTAGTGTGGACGACATCAGGAAACCAAACCCTCCGA
ATGACCATAAGCCAATGGAGAATATCCCTAGTCCCGGTTTTGGAAAAAAATTGAGGAATCA
ACAGGATGAGTGCTACAAAGGAAATGTGTCTGGATGTAAACTCTTTGGATTCTCCTTGACT
GCGGAATCACCTACTCTGAACTCACAAAATTCTGGTAAGCGGAGTTGTACAAAGGTTCAC
AAGCAAGGCAGCTTGGTTGGAAGAGCCATTGATCTCTCAAGACTCTATGGATATAAAGACT
TGATAACTGAACTAGAACATCTTTTCGGTATGGAAGGTCTTTTAAGTGCTCCTGATAAAGG
GTGGCGGGTTTTGTACACTGACAGGGACAATGATGTAATGGTAGTTGGAGACGACCCGT
GGCATGAATTTTGTGATGTGGTATCCAAGATCCACATATACACAGGAGAAGAAGTGGAGAA
GATGACCATAGGAATGGGTAGTGATGAGACTCAAAGCTGTTTGGAAGAAGCAGCAGTGAT
AATGGAAGCATCAAAGTCGTCCTCGGTGGGGCAGCCAGATTCCTCTCCCACTGTAATGA
GGGGTATGAAGATCAGTGCTTGCTTTTAG

>GaARF5-1
ATGGATTTCAGTGTTCTCAATGAAGCGTTGGTCTCTAAATCCTTCAATAAGATCGCTGATAT
ATGCGATAATCTCATGCTTCAGGTAGCAGCCGAGGGCGTTCCTTTTCGAGACGAATGGCC
TTTCGCAATTCATCTTCTGGGTCACATCTACATCGATGATATTAATAGTGCAAGGTTTCTCT
GGAAATCAATACCTCCTGCAATCAAAGAGAGCCAACCGGAGGTGGTTGCGGCCTGGAAA
ATAGGTCAAAAGCTGTGGACAAGGGACTATGCTGGTGTCTACGAGGCTATTCGTGGTTTT
GATTGGACTCAACAAACACAAGTTCTTGTTGCTGCTTTCTCAGAGCTTTACACTAAGAGGA
TGTTTGAACTGCTGCAATCTGCTTATTCGACAATAAGCATCCAAGATGCAGCTCAATTTCTT
GGAATGAGTGAGGAAGATGCCTCGAATTATGTACTCGGACAAGGTTGGACGGTCGATCCT
ACTTCTCGAATGATAACCGTGATGAAGCAGGCTATCGTGAAGGAGCAGAAGTTAGACCCG
GGCAAATTGCAGCGACTAACTGAATACGTCTTCCACCTCGAACATTAA

>GaARF5-2
ATGGGTTCTGTCGTTGAAGAGAAGATCAAACAAGGAGGTTTGGTTAATGTAGGTGCACAG

TCCACTCTGCTTGAGGAAATGAAGCTATTGAAAGAAATGCAAGATCAATCTGGTTCGTCCT
TTGTTGGTACCCGTAAGGCTATAAATTCCGAGTTATGGCATGCCTGTGCTGGTCCACTTGT
TTCCTTGCCTCAGGTGGGAAGTCTTGTGTATTACTTTCCTCAAGGACATAGCGAACAGGT
AGCAGTGTCCACTAAAAGAATGGCGACTTCTCAAATTCCCAACTACCCAAATCTTCCATCT
CAGTTAATGTGCCAAGTTCATAACGTTACATTACATGCAGACAGAGACACCGACGAAATAT
ATGCCCAAATGAGTCTTCAACCAGTGAACTCTGAAAAAGATGTGTTCCCTATACCAGACTT
CGGATTGAAGCTGAGCAAGCATCCTAATGAATTTTTCTGCAAAACTTTGACTGCAAGTGAT
ACAAGTACACACGGTGGTTTTTCAGTGCCACGTAGAGCAGCTGAGAAGCTCTTTCCTTCA
TTGGATTATTCCATGCAACCTCCAACGCAAGAGCTTGTTGTGAGAGATTTGCATGATAACA
CCTGGACGTTTCGTCATATATACCGTGGGCAGCCGAAGCGACACCTTCTTACTACAGGGT
GGAGTTTGTTTGTAGGATCAAAAAGACTTAGAGCTGGTGATTCCGTTCTCTTTATCAGGGA
TGAGAAATCACAGTTATTGGTGGGTGTAAGGCGGGCTAATCGTCAACAAACCACATTGCC
ATCATCTGTTCTATCTGCTGATAGTATGCACATTGGTGTCCTTGCTGCCGCCGCTCATGCT
GCTGCCAATAGAAGTCCATTCACAATTTTCTACAATCCAAGAGCATGCCCTTCAGAATTTG
TCATCCCTTTGCCTAGATACCGTAAATCTGTATATGGGTCTCAAGTCTCAGTCGGTATGAG
GTTTGGAATGATGTTCGAAACGGAGGAGTCCGGGAAACGTAGATATATGGGTACAATAGTT
GGTATTAGCGACTTGGATCCTCTAAGATGGCCTGGCTCAAAGTGGCGAAACCTTCAGGTT
GAATGGGATGAACCTGGATGTAATGATAAACAGAATAGGGTGAGCGCATGGGAAATTGAA
ACTCCTGAAAGCCTCTTTATTTTTCCTTCGTTAACTTCAAGTCTGAAGCGACCATTGTATCC
TGGATTTTCAGGAGCAGAATCTGAATGGGGAAGCTTGATGAAAAGGCCCCTACTCCAGTT
TCCTGAAAATGGAAATGGGAATCTTCCCTATTCAATGTCAAATTTATGTTCCGAACAATTGA
TGAAGATGATGTTGAAGCCTCAGCTTGTTAACCATCCTGGAATTTTTGCTTCCCCCTTACA
ACAAATCGCTGATGTAAAGATACCTCCATTAGAAGAAATGAAGAACTTGCAGTCTAAAAGC
CACCCAAAACCCCAGGTTATCCAATCAGAAAATATGTTGATAGAGAACCAAAATCTTTCCC
ACCCAGTCCCTGACCATCCTGATCCCATAACTTCAAATATGTCCAAAATCAATGCTAATGG
GAACCCACATCCTGCAAATATTCTAACACAAGCTGGGACTGGGAGCAGTAATGAAAAATTA
AAGTTGGAATCAAAGCATTCAGCCGAGCAACTGACTTCGACATCGGAATGCAATGAGGAA
AAATTGGTGGCAAGTACTGTAAACACAACTATGTCGAACCAACTTTCTTTCCCTACCCAGC
CCCAGATCCCACTCCATGTGCAAAATAATCCCTGGTCGATTCAGTCACAATTGGATTCATC
AGTCCTCCAAGCTCATCAAATGCTTGTATCCCAAGCTGATATTAGTACTTTAAACAGCTTTC
TTCCTTTCTCAGACACCGATGAGTGGACGTCAAATCTTTCTTCTTGCCAACCTCTTTCTGG
GGCGTACAAATCACCTGGTCCGATACCAATGGTTGGGTTACAGGACTCTTCAGCTGTCTT
TCCAGTTGAAACTGATGATTCGTTAACTACGGTGGGTGAGGAAATATGGGATCCAAAGCT
GAATAGTTGCAGAGTTTCATCCCAAGCAGACCAATTGGCTTCATTCACTCAGCAAGATCCA
TGCAGTCTTAATTCTGGTGGGGTAAGGGATTTGTCTGATGACAGCAACAATCAAAGTGGG
ATATATAGTAGCTGTCTTAACATTGATGTTAGCAATGGTTGCAGCACCGTGATTGATCCTTT
TGTTTCCAGTGCCATTCTAGATGAGTTTTGCTCATTGAAAGATGCTGATTTTCAAAACCCTT
CAGATTGTTTGGTCGGGAACTTTGGTTCTTGTCAGGATGTTCAGTCTCAGATTACCTCTG
CTAGCCTTGCAGATTCTCAAGCTTTCTCTCGACAAGACTTGCCTGACAGCTCCGGTGGCA
ATATCGATTTTGATGACAGTGGTCTTCTGCAAAACAATTCCTGGAAGCAAACAGCTCCACG
CGTTAGAACATATACAAAGGTTCAGAAGGCAGGATCTGTAGGAAGGTCGATTGATGTTAC
GAGTTTTAAGAATTACGATGAACTAATCTCTGCAATAGAATGCATGTTTGGACTCAAGGGT
CTGCTGGATGATCCCAGAGGTTCAGGCTGGAAATTGGTGTATGTGGATTATGAGAATGAT
GTTCTTCTCGTTGGAGACGATCCTTGGGAGGAATTTGTCGGGTGTGTTCGTTGCATCCGA
ATTCTATCCCCTACAGAAGTACAACAGATGAGTGAAGAAGGAATGAAGCTTCTCAACAGT
GCTGCCACAGTGCAAGGCATCAATGGCTCTAACTCAGATGGTTCCAATGCAAATGCTTAA

>GaARF6-1
ATGAGGCTTTCTTCAGCTGGTTTCAGTCCACAAGCTACGGAAGGAGAGAAGAGAGTTCT
GAACTCTGAACTTTGGCATGCATGTGCGGGTCCTCTTGTATCTCTACCTCCGGTTGGAAG
TAGGGTTGTTTACTTCCCACAGGGTCATAGCGAACAGGTAGCTGCATCAACCAACAAGGA
GGAAGTGGATGTCCACGTACCTAACTACCCAAGCTTACCTCCAGAACTTATATGTCAGCTC
CATAATGTCACCATGCATGCAGATGTTGAGACAGATGAAGTATATGCACAAATGACATTGC
AACCACTGAATCCGCAAGAACAGAAGGAGGCTTACCTTCCAGCAGAATTGGGCACTCCC
AGCAGACAGCCAACAAATTATTTCTGTAAAACATTAACAGCCAGTGACACAAGCACTCATG
GAGGGTTCTCTGTTCCTCGCCGAGCTGCTGAAAAAGTGTTTCCCCCACTGGACTTCTCC
CAGCAGCCTCCTGCTCAAGAGTTGATTGCAAGGGACTTACACGATAATGAATGGAAATTTA
GGCATATATTTCGGGGTCAGCCCAAAAGGCATCTCTTGACAACAGGATGGAGCGTATTTG
TAAGTGCTAAAAGACTAGTTGCAGGTGATTCAGTTCTTTTTATCTGGAATGAAAAAAATCAA
TTACTTCTTGGCATCCGACGAGCTAATCGACCTCAAACTGTAATGCCTTCATCTGTTTTATC

GAGTGATAGCATGCACTTAGGGCTTCTTGCTGCTGCTGCTCATGCAGCTTCAACAAATAG
CCGCTTCACTATATTTTATAACCCAAGGGCTAGTCCATCAGAATTTGTAATACCTTTGACAA
AATATATCAAAGCTGTCTATCATACTCGAGTTTCTGTTGGTATGCGCTTTAGAATGCTGTTT
GAAACAGAAGAATCAAGTGTTCGTCGATACATGGGTACAATCACTGGCATAAGTGACTTG
GATCCTGTCCGGTGGCCAAATTCACATTGGCGATCAGTTGGCTGGGATGAATCAACAGCT
GGAGAAAGGCAGCCTAGAGTCTCCTTGTGGGAGATTGAACCATTGACAACATTCCCTATG
TATCCATCACCATTTCCATTAAGACTTAAGCGACCATGGCCAGCAGGATTACCTTCTTTCC
ACGGCATCAAGGATGATGGTCTAGGTATGAATTCTCCGCTTATGTGGCTGCAAGGAGATG
CAGGTAGAGGAATGCCATCTCTGAATTTTCCAGGGATTGGAGTTACACCGTGGATGCAGC
CAAGGCTGGATGCTGCTTCCATGCTGGGTTTGCAGAATGACATGCACCAAGCTATGGCTG
CTGCTGCTCTGCAAGACATGAGAGCAGTGGATCCCTCCAAATCAGCAACTACTACCCTTC
TGCAATTCCAGCAACCCCAAAATCTATCCTGCAGGCCTGCTGCTTTAATGCATTCCCAGAT
GTTGCAGCAGTCTCAGCCTCAGGCTTTTCTTCAGGGTGTTGAAGACAACCAACATCAGTC
TGAGACTCAGGCTCAAACCCAACCGCCTCTTGTTCAGCAACAATTGCAGCAGCAGAATTC
ATTTAATAACCATCAACACCAACAGCAGTTACAGCATCCGCTGTCACAGCAACACCAGCAA
CTGGTCGATCATCAGCATATTTCTACTGGAGTGTCTGCCATGTCACAGTATGCTCCGGCCT
CACAGTCCCGGTCGTCACCTTTCCAAGCCATACCTTCACTATGCCAACAACAGAGTTTTT
CTGACTCAAATGGGCACACCGTGACCAGCCCCATTGTATCTCCCTTGCACAGTCTTTTGG
GATCCTTTCCCCAAGATGAACCGTCTGGTCTGCTCAACTTGCCTAGATCCAACCCAGTAAT
AACATCTGCAGCATGGCCATCTAAGCGGGCTGCTGTTGAAGTTCTGTCATCTGGATCTCC
ACAATGTGTTCTGCCCCAGGTGGAACAGTTGGGGCCCACCCAAACAAACATTTCTCATAA
TTCTATTTCGTTGCCACCCTTTCCTGGCAGGGAGTGCTCGATAGACCAAGCAGCGGGTAC
TGATCCACAGAGCCATCTCTTATTTGGTGTTAATATAGAGACTTCATCTCTTCTATTGCAAA
ACGGGATGTCAAGCCTTAGGGGAGTTGGCAGCGAGAGTGACTCCACTACTATACCTTTCT
CTTCTAATTATGCGAGTACTGCAGGCACTGATTTTTCAGTTAATCCAGCAATGACACCTTC
CAGTTGCATTGATGAATTGGGATTCTTGCAGTCTCCAGAGAATGTGGGCCAAGAAAACCC
ACAAACCAGAACCTTTGTTAAGGTTTATAAATCAGGGTCCTTCGGGAGGTCGTTGGATATC
TCCAAATTTAGCAGCTACAATGAGCTGCGCAGTGAACTTGCACGCATGTTTGGCCTTGAA
GGCCAGTTGGAGGACCCTTTGAGATCAGGCTGGCAGCTTGTATTTGTTGATAGGGAGAAT
GACGTTCTTCTCCTTGGTGATGATCCTTGGCCGGAGTTTGTGAACAGTGTGTGGTGCATC
AAGATACTTTCACCGCAAGAAGTGCAGCAAATGGGCAAACGAGGCCTGGAGCTTCTAAA
CTCTGTTCCAGTTCAGAGGCTCTCGAATGGCAGTTGTGATGACTATGCGAGCCAGCAGG
ACTCGAGAAATTTGAGCTCTGGTATTGCCTCTGTGGGGTCATTGGACTACTGA

>GaARF6-3
ATGGGTCGGGGTAGGTATTGTCCGAGTAGTTACTTGCGTTTCTATTCTTCCTTTCGTTTCC
TTTCTACGAATGCCGCCTCTGAATCTGCTCCTAATTTCACCTTAGAATTCACCGATCTCTG
CTCCAAAGGACTCACAAAGGAAGCCTTTGATAGGTTCTATCCTCAAATATGGGCAGACCC
ACCTCTGTTTTCCCACCTCATCCAATCTTGCATACCGCAAAACTCGCCATCTCTTGGCAAA
CAGCTTCATTCTTTGGTCATCACATCTGGGTCCTCAAAAGATAGGTTTATATCCAATCACTT
GCTCAACATGTATTCCAAGTTCGGGAACTTACAAGCTGCCGTTTCCTTGTATAATGTGATG
GTTGTGAAGAATGTAATGTCTCGTAACATTTTGATCAATGGACATTTGCTTGTTGGGGACT
TGGATAGTGCCAAAAAGTTGTTCGATGAAATGCCTGAAAGAAATGCCGCCACGTGGAATG
CGCTTGTTGTGGGGTATATCCAGTTCGAGTTTAATGAGGAGGGACTAAGTGTGTTCAGAG
AAATGCATGTTTCTGGGTTCAGGCCGGATGACTTCACTCTTTCTAGTGTTCTGAGAGGGT
GTGCCGGATTGAAAGCTTTGTCAGTAGGGAGGCAAGTTCATTGTTGTGTGATGAAATGCG
GGTTTGGGATTCATTTAGTTGTTGGGAGTTCCTTAGCTCACATGTATATGAAATGTGGAAG
TTTGGAGGAAGGGGAAGCGGTAATTAAATCAATGCCAATTCGTAATATGGTTGCTTGGAAT
ACTCTCATTGCAGGGAATGCTCAAAACGGCTATGGTGAGAGTGTTTTGGCCCTTTACAGT
ATGATGAAGATGACTGGTTTTAGACCTGATAAGATTACATTTGTTAGTGTCCTGAGTTCATG
TTCAGAGTTGGCAACCTTAGGACAAGGTCAGCAGATTCATGCTGAGGTGATTAAAACCAG
TGCAAGCTCAGTTATCGATGTGATAAGTACATTGATTAGCATGTATTCAAGGTGTGGTTGCT
TGGAAGATTCCATTAAGGTTTTCATGGAATGTGAAGTAGCAGATCTTGTGTCATGGAGCTC
GATGATCGCTGCATATGGGTTTCACGGAAGAGGAGTGGAAGCAGTTGAGTTGTTTGAACA
TATGGAAAAAGAAGGATTGGAGCCAAATGATGTTACTTTCTTGAGCTTGCTTTATGCTTGC
AGTCACTGTGGGCTTAAAGATAAAGGACTTGAGTTCCTTAACTTGATGACAGAGAAGTATG
GAATAAAGCCTAGGGTACAACACTATACTTGCATAGTCGACTTGCTCGGTAGGTCTGGCT
GTTTGAATGAAGCAGAAGCTATGATAAGATCAATGCCGGTGAAAGGAGATGCTATCATATG
GAAAACTTTGTTGTCTGCATGTAAGATCCACAAGAATGCCGACATGGCAAGAGAAGTAGC
TGAAGAAGTGCTTAAGCTTGATCCTCAGGATTCAGCTTCCTATGTTCTCCTTTCGAACATT

CATGCATCTGCAAAAAGGTGGCTGGAGGTTTCGGTGGTAAGAAAAACCATGAGAGATAG
GAGGGTGAAGAAAGAGCCTGGCATAAGTTGGTTGGAAATCAAGAATCAAGTTCATCAGTT
TTGCATGAGTGATAAAGCGCATCCGCAATCGGAGGAGATTGATTTGTACTTGAAGGAACT
GACCGCTGAGATGAAGTCACACGGATATGTGCCTGACACAAGTTTAGTTCTACATGACAT
GGATAATGAGGAGAAAGAATACAGGTTGAGTCACCATAGTGAAAAAATGGCTATTGCATTT
GCTCTAATGAATACTCCTGCTGGAGCCCCTATAAGGGTGATGAAGAATTTGAGGATCTGCA
GTGATTGCCATGTTGCTTTTAAGATCATATCAGCGATCAAAAATAGAGAAATTATTGTACGA
GATGCTAGTAGATTTCATCATTTCCAAAATGGAAAATGTTCTTGTAGAGATTACTGGTGA

>GaARF6-5
ATGAGACTCTCTTCATCAGGGTTGAATCAGCAAACTCAGGAAGGGGAGAAGAAATGTTTG
AATTCTGAACTTTGGCATGCATGTGCTGGTCCTTTGGTGTCTCTTCCACCTGTTGGAAGC
CGAGTTGTTTACTTTCCACAAGGTCATAGTGAACAGGTTGCTGCTTCTACTAACAAGGAAG
TAGATGCTCATATACCGAACTATCCGAGCTTACCCCCACAACTTATCTGTCAGCTTCACAAT
GTGACCATGCATGCAGATGTGGAAACAGATGAAGTGTATGCTCAAATGACCTTGCAGCCA
CTGAGTCCACAAGAGCAAAAGGATGTCTACCTACTGCCTGCAGAACTAGGTGCTCCCAG
CAAACAGCCAACCAATTATTTTTGTAAAACACTAACAGCAAGTGACACTAGCACTCATGGA
GGATTCTCTGTTCCGCGCCGTGCTGCTGAGAAAGTCTTTCCTCCTCTTGACTACACCCAG
CAACCACCAGCTCAAGAGTTGATTGCCAGGGATCTTCATGACAATGAGTGGAAATTTAGA
CATATATTTCGAGGCGAGAATGATAAGAATCAATTGTTGTTGGGTATACGGAGAGCAAACC
GTCCTCAGACTGTTATGCCTTCTTCAGTTTTATCAAGTGATAGCATGCACATTGGTCTTCTT
GCTGCTGCAGCTCATGCGGCTGCGACCAATAGCCGTTTTACTATATTTTACAACCCAAGG
GCTAGTCCTTCAGAGTTTGTCATTCCTCTTGCCAAGTATGTTAAAGCTATCTCTCACACCC
GGATTTCTGTAGGCATGCGGTTTAGAATGCTGTTTGAGACAGAAGAGTCAAGTGTCCGAC
GATACATGGGTACAATAACTGGCATTAGTGACTTAGATCCTGTTCGCTGGCCAAATTCGCA
TTGGCGCTCTGTTAAGGTTGGCTGGGATGAATCCACTGCAGGAGAGAGGCAGCCAAGAG
TTTCCTTGTGGGAGGTTGAACCGTTAACAACCTTCCCTATGTATCCATCTCCCTTCCCCCT
GCGGCTGAAGCGACCATGGCCGTCTGCACTACCCTCCTTCCATGCTTTCAAAGACGGTG
ATATGAGCATTAATTCCCAATTGATGTGGCTTCAAGGTGGGGTCGGGGATCAAGGACTTC
AGTCTTTAAACTTCCAAGGATTTGGAGTTGCACCTTGGATGCAACCAAGGCTTGATACTTC
TTCAATACCAGGTGTACAACCTGATCTTTACCAAGCAATGGTGACTGCAGCACTTCAGGAT
ATGAGGACAGTTGATTCCTCCAAAATAGGCTCTCAATCTCTCTTGCAATGCCAGCAAAACC
AGAGCACATCCACTGGGCTGCCTGCTCTAGTTCAGAGGCAAATGTTACAACAGTCCCAAA
CACAAAATGGGTTTCTTCCAAGTTTTCAGGAGAATCAGACTGCTTCACAGGTTCAACTTCT
GCAGCAGTTGCAGTGCCCCAATTTGTACAGTGACCAGCGACAAAAGCAGCAGCAGCAAC
AGTCTCAAGAAACACAGCAACTGCCACCTGTTCCACAGCAGATTTCTAATGTAATCCCTG
CTTTTCCTTCTGTGTCAGCCAACCAAGCGCAGTCATCTCTGCCAGCTGTTGATTCACAAT
GCCAGCAGTCGACATTTTCCGACCATCTTGGGAACTCCGTAGCTACATCTGATGTTTCTTC
TATGCAAAGTATCTTAGGTTCATTATCCCAGATGGGAGCTTCCCATTTACTCAACTTGAATG
GATCAAACCCAATTCTCTCTTCTTCTACTTTCTTGTCCAAGCCTGCAGCTATTGAACCACG
GCTTTCATCCGGAGTGGCCAACTCTGTACTTCCCCAGGTTGAACAGTTGGGAACTGCAC
AATCAAATGCAGCTGAACTTAACAACTTATTACCTCCATTTCCTGGAAGGGAGTATTCTGCT
TACCACAATGCAACTGATCCACAGAACAATCTTCTCTTTGGGGTTAGCATTGATTCCTCGT
CTCTTATGCTGCATCATGGGATGACAAACCCGAAAAGCATCAGAAATGAGAATGATTCGAT
GTCCCTTCCGTACGCTGCTTCAAATTTCACAAGTGCTTCTGGCACAGATTTTCCTCTTAAT
TCAGACATGACTACCTCAAGTTGTGTCGATGAATCAGGTTACTTGCAGTCCTCTGAAAATG
TGGACCAAGTAAACCCTCCAACTGGAACCTTTGTCAAGGTTCACAAGTCGGGGTCCTTT
GGGCGCTCATTGGATATTTCCAAGTTTGGCAGCTATGATGAGCTGCGCTGTGAGCTTGCT
CGACTATTTGGCCTTGAAGGCCAATTAGAGGACCCTCAGAGATCAGGCTGGCAGCTTGTA
TTTGTTGACAGGGAGAACGATATTCTTCTCCTTGGTGATGACCCATGGCAGGAATTCGTG
AACAATGTGTGGTATATCAAGATACTATCCCCACTTGAAGTACAACAAATGGGGAAAGGTC
AGAATCCTGCTACTTCTATTCCGAACCAAAGGCTTACCACCACCACCGCTACCACCACCA
GCAGCAATGGCAACCACTGCGATGACTACATGAGCAGACAGGACTTGAGAAGTTCTGTT
GCATCAATGGGGTCTCTTGAATACTAA

>GaARF6-8
ATGAGACTCTCTTCATCAGGGTTTAATCAGCAAACTGAGGAAGGGGAGAAGAAATGTTTG
AATTCTGAACTTTGGCATGCATGTGCGGGTCCTCTGGTGTCCCTGCCGCCTGTTGGAAG
CCGTGTTGTATATTTTCCCCAAGGTCATAGTGAACAGGTTGCTGCTTCAACCAACAAGGAA
GTAGATGCTCACATACCGAACTATCCTAGCTTACCCCCTCAGCTTATCTGTCAGCTTCACA

ATGTGACCATGCATGCAGATGTAGAAACAGATGAAGTGTATGCTCAAATGACCTTGCAGC
CACTGAGCCCGCAAGAGCAAAAGAATGTGTTCCTCCTGCCTGCTGAACTGGGTACTCCC
AGTAAACAACCAACCAATTATTTTTGCAAAACACTGACGGCAAGTGACACAAGCACTCATG
GAGGATTTTCTGTGCCTCGCCGAGCTGCTGAAAAAGTCTTTCCTCCTCTTGATTACTCCC
TGCAACCTCCAGCTCAAGAGTTAGTTGCCAGGGATCTTCATGAAAATGAATGGAAATTTAG
GCATGTATTTCGCGGGCAGCCAAAGAGGCATCTTCTTACAACTGGGTGGAGTGTTTTTGT
TAGTGCTAAGAGACTTGTTGCTGGTGACTCAGTCCTTTTTATTTGGAATGAGAAGAATCAG
TTGTTGCTGGGTATCCGGAGAGCAAATCGTCCTCAGACTGTTATGCCATCTTCTGTTTTAT
CAAGTGACAGCATGCACATTGGTCTTCTTGCTGCAGCCGCTCATGCGGCTGCAACCAATA
GCCGTTTTACTATCTTTTATAACCCAAGGGCCAGTCCTTCAGAGTTTGTCATTCCTCTCGC
CAAGTATGTTAAAGCAGTCTATTACACCCGGGTTTCTGTAGGCATGCGGTTTAGAATGCTG
TTTGAGACAGAAGAGTCAAGTGTCCGCCGATATATGGGTACAATAACTGGCATTAGCGACT
TAGATCCTGGTCGCTGGCCAAATTCGCATTGGCGCTCTGTTAAGGTTGGCTGGGATGAAT
CCACTGCAGGAGAGAAGCAGCCAAGGGTGTCCTTGTGGGAGATTGAACCATTAACAACC
TTCCCTATGTATTCATCACCGTTCCCCCTGCGATTGAAGCGACCATGGCCATCTGCGCTAC
CATCTTTCCATGCTTTCAAAGATGGTGATACGAACATCAATTCCCAAATGATGTGGCTTCAA
GGTGGGATTGGAGATCAAGGACTTCAGTCTTTAAACTTCCAAGGTTTTGGAGTTGCACCC
TGGATGCAGCCAAGGCTTGATACTTCTTCAATACAAAGTGTCCAACCTGATCTTTACCAAG
CAATGGCTGCTGCTGCATTTCAGGAAATGAGGACTGTTGATTCATCCAAAATAGGCTCTCA
GTCTCTCTTGCAATTCCAGCAACCACGGAGCATGACCGATGGGTCGCCGGCTATAATTCA
AAGGCAGATGCTACAGCAGTCTCAAACACAAAATGCCTTTCTTCAGGGATTTCAGGAGAA
CCAGACTACTTCCCAGGTTCAGCTTCTGCAGCAGTTGCAGCGTTCCAATTCATACAATAAT
CACCGACAACAGCAGCAGCAACAGCAACAACAGCAACAATCTCAGCAAATGCAACAAAC
ACCACAGTTTTCTGATCAACAGCAGATTTCTAATCTAATCCCTGCTTTTCCTAAAGCCTCTG
GCAGCCAGGCCCAGTCCTCATCTCTGCCAACTGCTGCATCTGAATGCCAGCAGCAGACA
TTTTCTGATCCTCTTGGGAACTCTCTAGCCTTATCTAATGCTTCGTCAATGCAAAGTATCTT
AGGTTCATTGTCCCATGCTGGAGCTTCCCATTTACTCAACTTGAAGGGATCAAACCCAATT
ATCTCTTCTTCTTTGTTGTCCAAGCCAGTAGCTATTGAACCACAGCTTTCATCTGAAACTG
CTAACTACGTACTGCCCCAGGTTGAACATCTGGGAATGGTACAGTCAAATGTACTCTCTAA
CTTATTACCTCCATTTCCAGGAAGAGAGTATTCTGCTTACCAGAGTTCAACTGATCCACAA
AACAATTTTCTGTTTGGGGTTAGCATTGATTCATCATCTCTTATGCTGCAGCAAGGGATGA
CCAACCTGAAAAATACTGGAAATGAAAATGATTCATTGTCTATGCCATATGCCGCTTCAAAT
TTCACCAGTGCTTCTGGCACAGATTTCCCTCTTAATTCAGATATGACTACCTCAAGTTGTG
TGGATGAATCAGGTTACTTGCAGTCTTCTGAATATGTGGACCAAGTAAACCCTCCGACCG
GAACCTTTGTTAAGGTTCACAAGTTGGGGTCCTTTGGGCGCTCATTGGATATTTCCAAGTT
TAGCAGCTATAATGAGCTGCGTTGTGAGCTTGCTCGAATGTTTGGTCTGGAAGGCCAACT
AGAGGACCCTCAGAGATCAGGCTGGCAGCTTGTATTTGTTGACAGGGAGAATGATATTCT
TCTCCTTGGTGATGACCCTTGGCAGTAA

>GaARF7-2
ATGAAGGCTCCACCAAATGGATTTATGGCAAATTCTGCAGAAGCAGGAGAAAGGAAGAGT
ATCAATTCAGAATTATGGCATGCTTGTGCTGGACCACTTGTTTCTTTGCCTCCAGTTGGAA
GTTTGGTTGTTTACTTTCCTCAAGGTCACAGCGAGCAAGTTGCGGCGTCGATGCAAAAG
GAGACTGATTTCATACCAAGCTACCCTAACCTTCCTTCCAAGTTGATATGCATGCTCCATAA
TGTCACATTGCATGTAAGCAAAAGCAGGGCACTATCATCCCAAGCCGATCCAGAAACAGA
TGAGGTGTATGCCCAGATGACACTTCAACCTGTGAACAAATATGACAAGGAAGCGTTACT
GGCATCTGATATGGGCCTCAAGCAAAGCCGGCAACCTGCTGAGTTCTTTTGCAAGACTCT
TACAGCAAGTGACACTAGCACTCATGGTGGATTTTCTGTGCCTCGACGAGCAGCTGAGAA
AATCTTCCCTCCCCTGGATTTCTCGATGCAACCACCTGCTCAGGAGCTAGTAGCTAAAGA
TTTACATGACAATACATGGCCATTTAGACATATTTATCGAGGTATGGTTCTCTTCCAGATATT
GCTGTCCGTCCTTTTCATAATCGGAAAAATGGAATTATATATGCATAATGGCTTCTTTGCAG
GTCAACCGAAGAGGCACCTTCTGACTACTGGTTGGAGTGTCTTTGTTAGCACAAAAAGGC
TCTTTGCCGGTGATTCTGTTCTTTTCATAAGGGATGAGAAGTCTCAACTTCTGTTGGGTAC
AAGGCGTGCAAATAGACAGCAGCCAGCTCTTTCTTCATCTGTGATTTCTAGTGATAGCATG
CATATAGGGATCCTGGCTGCTGCAGCGCATGCTGCAGCAAATAACAGCCCATTTACTATAT
TCTATAATCCAAGGTATTATTTTATTAATTCTGATAGGGCAAGCCCTTCTGAGTTTGTGATAC
CCTTGGCAAAGTATAACAAAGCCATGTATACCCAAGTTTCCCTTGGCATGCGGTTTAGAAT
GATGTTTGAAACCGAGGAGTCTGGAGTACGCAGGTACATGGGTACAATTACTGGTATCAG
TGACTTGGATCCTGTGCGATGGAAAAATTCACAGTGGCGCAATCTTCAGGTTGGATGGGA
TGAATCTACAGCTGGTGAGCGGCCCAGCCGAGTTTCAATTTGGGACATTGAGCCTGTTAT

AACTCCTTTTTACATCTGTCCACCTCCGTTTTTCAGGCCCAGGTTTCCAAAGCAACCGGG
GATGCCAGATGATGAGTCTGATATTGAGAATGCCTTCAAGAGAGCTATGCCCTGGCTAGG
AGATGACTTTGGTATGAAAGATGCTCCTAGTTCAATCTTTCCTGGTTTGAGTCTAGTCCAG
TGGATGAGCATGCAACAAAATAATCAGTTTCCAGCTGCTCAATCAGGATTCTTTCCATCAA
TGGTTTCTTCGAATCCGCTCCATAATAGCCTTGGCACCGATGATCCTGCCAAATTATTGAA
CTTTCAAGCTCCTGCGCTACCAGCATCAAATATGCAATTTAACAAAGCAAATACGAACCAA
ATCAATCAGTTGACTCAGGCACCTATGACATGGCCCCAGCAACAGCAACTTAAACAGTTAT
TGCAGACTCCTTTAAATCAGCAGCAGCAATCCCTACAGCAATTGCAGCGACAACTGCCAC
AACCACCACAGCAGCAGCCTCAGCCTCAGCCACAGCCGCATCTTATTCAACAGCAGCAG
CCTCAGCCTCAGTCTCTGTCTCAGTCTCAGTCTCAGTCTCAGTCTCAGTCTCAGTCACAT
TCGCAGCCACAGCCACAGCCACAGCCACAGCCACAGCCACAGCCACAGCCACAGCCAC
AGGCACCTCCACTGCAGCAGCACCTGCAGCAACAACATCAAAAAAGACAACAGACGCAA
CCACAGCAGCAGCTGCAACAAGCATTCCTCCCTTCTCAAGTAAATAATGGCATCATTGCCT
CTAACCAGTTCCCAAATCAAAATTTGCACCAGCCAGCTGTTTACTCACAGCTTCAGCAGC
AACAATTGATGACAAGCAGTAGCCAATCTGCCCAGACTGCCCCCTCTGCCAATAAAACTT
CATATCCTTTGACATCATTATCGCAAGATACACAGATTCAGCAACAGATGGAACAGCAACC
TGACCTCATGCAGAGGCAGCTGCAACAGACACAGTTGCAGCAGTCACAACTACAATTATT
GCAACAAAGCCTGTCCCAGAGGACACAGCAGCAGCCACAGCTTCTGCAATTGTCACAGC
AGGGCTTCTCGGAGCAACAGCTTCAGTTACAACTGTTACAGAAATTGCAGCAACAGCAGC
AGCAGCAGCAATCATCTCAACAATTACTCTCCCCAGCTGGATCACTGTTGCAGCCTCAAA
TGTTGCAGCAACAGCCGACCCATCAACAGAGCCAACCATTGCAGCAATTGCCTCTTTCAC
AAAGCCAATCTCAACCTCTTGGCAGCAATGGCTTCTCAACGTCAATGCCTGTTCAACCTC
AACAGGTTTCAGTGAACCAACCCCAAATTCACCAGCCATTTACTGCTATGAGAACTCATTC
TGGTCTTACTGACGGAGATGCTCCATCATGTTCAACCTCACCTTCTACCAATAACTGTCAG
GTTTCCTCATTGAACTTTTTAAACAAAAATCAGCAAGTTCCATCCATATTGGTGTCAGATCC
TCTTGTTGAGCCTGCAAATACCCTGGTTCAAGAGCTTCAGAGCAAGCCTGATATTAGAATC
AAACATGAACTACCCACTTCTAAAGGACCAGAACTATCAAAGTACAAAAGTACTGTGACAG
ATCAATTAGAAGCATCCTCTTCTGGAACATCATACTGCCTGGATGCGGGCACCATCCAGCA
TAATTTCTCCCTCCCCACCTTTTTGGAAGGTGATCTTCGATCACACTCTCGGAATAATCTT
CCTTTTACAACAAATATTGATGGATTGGCACCTGACACTTTGTTATCAAGGGGGTATGACT
CTCAAAAGGATCTTCAAAACATGTTGTCGAACTATGGTGGCAACCCAAGAGATATTGATGC
GGAGTTGTCCACTCCTGCGATAAGCTCTCAGTCGTTTGGTGTGCCAAACATACCTTTTAA
GACTGGGTGCTCAAATGATGTTGCTATCAGTGACACAGGAGTTCTAAATGGTGGACTGTG
GGCCAACCAAACTCAACGCATGCGAACATATACAAAGGTGCAAAAGCGTGGGTCTGTAG
GAAGGTCAATTGATGTGACCCGCTACAAAGGGTATGATGAGCTTAGGCATGATCTAGCGC
GCATGTTTGGTATAGAGGGGCAACTGGAAGATCCGCAAAGTTCTGACTGGAAATTAGTTT
ACGTGGATCATGAAAATGACATATTACTTGTTGGTGATGATCCTTGGGAAGAATTTGTAAGT
TGTGTTCAGAGCATAAAGATACTGTCATCTGGGGAAGTACAGCAGATGAGCTTGGAAGGT
GATCTTGGAAATGTGGCAGTTCCGAATCAAGCTTGCAGTGGGACTGACAGTGGAAATGCA
TGGAGAGGACAATATGATGATACCTCAGCAGCGTCATTTAACAGA

>GaARF8-1
ATGAAGCTTTCAGCATCAGGCCAGGGTCAGCAGGCTCATGAAGGGGAAAACAAGTGCTT
GAACTTAGAGCTATGGCATGCCTGCGCTGGCCCACTGGTTTGTCTACCAACTGTGGGGA
CCCGTGTGGTCTACTTTCCTCAGGGTCATAGCGAGCAGGTAGCAGCCACCACTAACAAG
GAGGTTGACACTCACATTCCGAATTACCCGGACTTGCCTCCCCAGTTGATATGTCAGCTC
CAAAATGTTACCATGCACGCTGATGTTGAGACAGATGAAGTATATGCCCAAATGTCGTTGC
AGCCTTTGACGCCTGAAGAGCAGAAGGGTGCATTTCTTCCTATGGAGTTGGGAATTCCAA
GCAAGCAGCCAACCAATTATTTCTACAAGACACTGACAGCAAGTGACACTAGTACCCATG
GCGGTTTTTCTGTTCCTCGTCGTGCTGCTGAGAAAGTCTTTCCGCCACTGGACTTCTCAC
AGCAGCCTCCAGCTCAGGAACTTATTGCAAGGGATCTTCATGACATTGAGTGGAAATTTA
GACATATATTTAGAGGACAGCCTAAACGCCACCTTCTTACTACAGGCTGGAGTGTGTTTGT
TAGTGCCAAGAGACTTGTTGCAGGGGATTCTGTTCTTTTTATTTGGAATGAAAAGAACCAG
CTTCTTTTGGGAATTCGCCGTGCCACTCGTCCACAAACTGTAATGCCATCATCTGTTTTAT
CTAGTGATAGCATGCACATTGGACTACTTGCTGCTGCAGCTCATGCTGCGGCAACTAATA
GCTGCTTTACAGTATTTTATAATCCAAGGGCTAGCCCATCTGATTTTATAATACCCCTTTCAA
AATATGTCAAAGCTGTTTTCCACACACGTGTCTCGGTTGGGATGCGTTTTCGGATGCTTTT
TGAGACAGAAGAATCAAGTGTTCGTAGGTATATGGGTACAATAACTGGTATAAGTAGTTTG
GATCCTGTTCGTTGGCCAAATTCTCATTGGCGGTCTGTGAAGGTTGGTTGGGATGAGTCA
ACAACAGGTGAGAGGCAGCCAAGGGTATCATTGTGGGAGATTGAGCCTTTAACAACATTT

CCAATGTATCCATCTCTATTTCCCCTCAGATTGAAACGCCCTTGGCATCCGGGTTCCTCAT
CTTTGTTTGATAACAGAGATGATATATCTAATGGGTTAACGTGGCTAAGGGGAGGATCTGG
AGAGCAAGGACTACAATCTCTGAATCTTCAGTCCTTCTGCTCATATCCATGGATGCAGCAG
AAACTAGATTTGAGTTTCCCTGGAAATGATTATAATCTGCAGTACCAACATATGCTGGCCAA
TGGGTTGCAGAACTTGGGAAGCGGGGATCCGCCGAGACAGCAGTTGCAGCAATCTTTTC
AATATGTTCAACAGCCAGGCAGCCATAATTTACTGTTGCAGCAGCAGCAGCAGCAGCAGC
AGCAGCAGCAGTCTGTTTCACAGTTAGTTCCACACAACATTGTGCAGGCACAGCAATCCA
TAATTCGAATCGAGAGCTTTCTCCCAGTCCCTGGACGGGAACAAGTAGGCAATCAATCGG
AGGAACAGGCTCAGCAGCAGCATAACATGACTCAAAGTGATCAGCTCCAGCAGAGGCGG
CAGTTGAATGTGCCTTCATCATTTCTGAAACCAGATTTTATTGACTCAGGATCTGGTCCCC
CTGTTCTGGATATGCTGGGTTCCTTGTGTCCTGAAAGCAGTGCAAATCTTTTGAACTTCTC
CACAACTGGTCAGTCAATGCTAGCTGACCAGTTACCCCAACTGTCCTGGGCTCCGAAGTA
TGCTCATTTGAATGTAAATACCTTTGCTGGTTCAACATCAGTTCCACAAGTTTCTCCTGGA
AAAGATGCTACGGTAGAACTAGATATTGGTACCTCTGATGCCCAGAATTCTACTTTTTTTGG
TGTTAACGACGATTCATTTGGCCTTCTACTGCCCACCTCAATGCGTGGTTTCACTACATCT
TCAAGTGAAGCTGATATGCCCTCAATTCCATTAGGGGATCCCTCATTCCAGAATCCTTTGT
ATGGTTGCATGCAATACTCTTCAGAGTTGCAGAGCACAGGGCAAGTGGACCCACCAACTT
CATCTCGAACATTTATCAAGTTGCCATTTGGAGATTTAATCCCTGTTTTGGCAGCGGCGAA
TCGTGAAGCTCGGGGTTCTCCTCTCGCGAATTTGTAG

>GaARF8-2
ATGAAGCTTTCTGCTTCAGGCCTGGGTCAGCAGGCCGCTCATGAAGGGGAAAACAAATG
CTTGAACTCAGAGCTATGGCACGCCTGCGCTGGCCCACTGGTGTGCCTCCCCACCGTG
GGGAGCCGGGTGGTCTACTTTCCTCAGGGTCATAGCGAGCAGGTAGCAGCCACCACTAA
CAAGGAGGTTGACGCCCACATTCCAAATTACCCTAATTTGCCTCCTCAGTTGATATGCCAG
CTCCACAATGTTACCATGCATGCTGATGTTGAGACAGATGAAGTATATGCTCAAATGACGC
TACAGCCTTTGACACCAGAAGAGCAGAAGGATACATTTCTTCCAATGGAGTTGGGAATTC
CAAGCAAGCAGCCAACAAATTATTTTTGCAAGACACTTACTGCAAGTGATACTAGTACCCA
TGGTGGTTTTTCTGTTCCTCGTCGTGCTGCTGAGAAAGTCTTTCCTCCACTGGATTTCTC
GCAGCAACCACCTGCTCAGGAACTCATTGCAAGGGATCTCCATGACATTGAGTGGAAATT
TAGACATATCTTTAGAGGGTTCAGTGCTAGAATCTGCACTTATGTATGCTTTACGGGGTTGA
GGAAGTTACTGCCATGGAATGAAAATAACCAGCTTCTTTTGGGAATTCGCCGTGCCACTC
GCCCACAAACTGTTATGCCATCATCTGTTTTATCTAGTGATAGCATGCACATTGGACTCCTT
GCTGCTGCAGCTCATGCTGCTGCAACTAATAGCTGTTTTACAGTATTTTATAATCCAAGGTA
TATGGGTACAGTAACTGGCATAAGTGATTTGGATCAAGTTCGCTGGCCAAATTCCCATTGG
CGGTCTGTGAAGGTTGGTTGGGACGAATCAACAGCAGGTGAGAGGCAGCCGAGGGTAT
CGTTGTGGGAGATTGAGCCTTTAACAACTTTTCCAATGTATCCATCTTTGTTTCCCCTTAGA
TTGAAACGCCCTTGGCATCCTGGTTCCTCATCTTTGCATGATAACAGAGATGATGTATCTA
GTGGGTTAATGTGGCTAAGAGGGGGATCTGGGGAGTCCTTCAGCACATTTCCGTGGATG
CAGCAGAGAATAGATCCAAATTTCCCTGTAAATGATCATAATCTGCAGTACCAAGCCATGC
TTGCCAATGGGTTGCAGAACCTAGGAAGTGGGGATCCACTGAGACAGCAGTTGCAACAA
TCTTTGCCGTATGTTCAACAGCCAGGCAGCCATAGTTTACTGTTGCAGCAGCAGCAACAA
CGAGGTGTTTCACAATCAGTTCCACACAACATTGTGCAGGTACAATCCCAAATTCTAAGTG
AGAACTTGCCTTCTGTCCTTGGACGGGAACAAGTAAGCAATCACCCGGAGGAACAGGCT
CAGCAACAGCATAACATGATTCAAAGTGATCAGCTTCTGCAGAGGCAGCCAGCAAATTTG
CCTTCATCATTTCTGAAAACGGATTTTATAGACTCAGGAAAGTTCTCAGGATCAGTCCTCC
CTGTCCAGAATATGTTGGGTTCATTATGTCCTGAAAGCAGTGCAAATCTTTTGAACTTTTC
CAGATCCGGTCAGTCAATGCTAGTTGGGCAGTTAACACAGCGGTCCTGGGCTCCGAAGT
ATACCCATTCAGAGGTAAATGCCTTTGGTAGCTCTACATCACTTCCACAAGTGTTTCCTGG
AAAAGATGCTATAATAAAGCCAGATATTGGCAACTCCGGTGCCCAGAATTCTGCTCTTTTT
GGTAATAACAATTTATATGGCCTTCTGCTTCCCACAATGATGCCTGCTATTGCAACATCTTC
ATGTGAAGCTGATGTGCCCTCAATTCCATTAGGGGACTCCTCATTCCAGAATCCTCTGTAT
GGTTGCATGCAAGACTCTTCAGAGCTGCAGAGCAGAGGGCAAGTTGACCCACCGACCC
CAACTCCAACATTTGTCAAGGTTTATAAATCAGGGTCAGTGGGACGCTCGTTAGACATCTC
CAGGTTCAGCAGTTACCATGAACTGCGAGAGGAGCTGGCTCAGATGTTTGGAATTGAGG
GGAAGTTGGAAGACCCTTTAAGATCAGGCTGGCAGCTTGTATTTGTCGACAGGGAGAATG
ATATTCTTCTCCTTGGAGACGACCCATGGGAGGCATTTGTGAATAATGTTTGGTACATAAA
GATACTTTCACCTGAGGATGTGCAGAAGATGGGCGAGCAACGGGCAGAGCCTTTTAGCC
CCACAACCCCAGGTCAAAGGATGAATAGCACCGGCACTGACACCGGCACTGGTAGACTC
TCATCTGTTGGTTCCCTTGAATACTAA

>GaARF10-2
ATGAGAAAAGAGGCAGAAAAGAGCTTGGATCCTCAGCTATGGCATGCCTGTGCTGGATC
CATGGTTCAAATTCCACCAGTGAACTCCAAAGTCTTCTACTTTCCTCAAGGCCATGCCGA
ACACTCTTTATATCCGGTCGATTTTTCATCTTCTCCTCCAATCCCGGCTCTACTTCTTTGCC
GGGTGGCTTCTGTCAAATTCTTGGCTGATGCTGAAACCGATGAAGTGTATGCCAAGATCA
TGCTTGTACCATTGCCCAACACTGAGCCTGATCTCGAGAACGATGCCGTTTTCGGTGGTG
GGTCTGATAATGTGGAAAAGCCTGCTTCTTTTGCTAAGACATTGACTCAATCCGATGCAAA
CAACGGAGGTGGTTTTTCGGTTCCCAGATATTGTGCTGAAACCATATTTCCGAGACTGGA
TTACACTGCCGATCCTCCGGTTCAAACTGTGATTGCCAGGGATGTTCATGGTGAGATTTG
GAAGTTTAGGCATATCTATAGGGGAACCCCTAGGAGACATTTATTGACTACTGGCTGGAGT
AGTTTCGTTAACCATAAGAAACTTGTTGCTGGGGACTCCATTGTGTTCTTGAGAGCCGAA
AACGGTGAGCTTTGTGTTGGGATAAGGCGGGCCAAGCGTGGGAATGATACTGGAGCTGA
ATCTGGTCTTGGAAATGGAAACTACGTTAGTCCTTATGGAGGGTTTTCAGGTTTCTTGAAG
GAGGATGAGAGTAAGATAACCCGAAAACGGAGTCCGAGAGGGAAGGGAAAAGTGAGAG
CAGAGGCTGTTGTGGAGGCAGTGGCGCTTGCCGCTAATGGCCAGCCTTTTGAGATAGTT
TACTATCCGAGAGCAAGCACACCGGAGTTTTGTGTTAAGGCATCTGCAGTAAGGGCGGC
AATGAGGGTTCCTTGGTGTTCTTTGATGAGGTTCAAGATGGCTTTCGAGACCGAGGATTG
TTCTAGGATTAGTTGGTTCATGGGGACTGTATCTTCTGTTCATATTGCAGACCCCCTTCGC
TGGCCTAATTCCCCGTGGCGCCTTCTTCAGGTAACATGGGATGAACCAGATTTGCTGCAA
AATGTTGAACGCGTGAGTCCCTGGTTGGTTGAATTGGTACCGAACATGCTTCCCGTCCAC
CTGTCACCCTTCTCAACAGTGACCCCCAGAAAGAAGTTGAGGCTTCCTGAGCACCTCGA
TTTCCCCCTTGTCGAACAATTTCCGATGCCACCGTTTTCCGGTCATCCCCTCAGGTCAAG
CAACCCCTTACGTTGTTTATCTGATAATGCTCCTGCAGGCATACAGGGAGCCAGGCATGC
TCAATTCAGATTATCTTCATCAGATCCTCATCTTAATAAACTGAAGTCTGGACTGTTTCCGT
CTGGCTTCCAGCTGTTTGATCCACAACCTCGAGTTCCCAATGGCATCTCAATGACAAAGC
ACACAGACAGTAACGATGATAATCTATCGTGCTTATTAACAGTAGGGAATTCTAGTCCAAAA
AAGAAATCTGAAAACGGAAAGAGACACCAGTTTTTACTCTTTGGTCAGCCGATACTTACTG
AGCAGCAGCTTTCTCATAGCTGTTCAACTGGAGTAAAAACTTCGGTCAAGAATGAAGACA
AAAGAAAAGATTCTTCCAATGGTTCAGGATCTGCTCTTGAGAATCAATTGTCTCCAGAGAA
GTCATTTACTACCAGATTATTGTGGCGGCAGGACTATCAAACCCCAGAACCTGGCTCAGC
CACTGGTCATTGCAAGGTATTCTTGGAATCTGAGGATGTGGGACGAACTCTTGACCTCAC
AGTTCTCGGTTCTTATGAAGAGCTATACAGGAGGTTGGCTAACATGTTTGGAAGAGAAAGA
TCAGAGATGTTGGGCCATGTCTTGTATCGAGATGCAACAGGTGCTGTCAAACAAACTGGA
GATGAACCATTCAGTACTTTTATGAAGACGGTCAAAAGATTGAACATAAGGATGGATTCAC
GCAATGATGCCGATGGAAGGTCATGGCTCACAGGTATTCGAACTGCCGAAAACAGACTAG
AAGGGCCAAACAAGAGAGGTCCCTTGAGCATATTTGCATGA

>GaARF11-2
ATGATGGCGAATCGGGTAGTCTCGTTTTCGCAGACAAATAATGTCTCCTCTGAAGGAAATG
GATGCGATGATCTGTACATGGAATTATGGAAGTTATGTGCCGGACCACTGGTTGAGTTGG
AAGCATCCACGAATCAGACGTTGAATCAGAGGATTCCGTTGTTTAATCTTCCATCGAAGAT
TCTTTGCCGTGTCGTTCATATTCAGTTGCTGGCTGAAAAAGAAACAGATGAAGTTTATGCA
CAAGTGACTTTACTGCCGGAACCAAGTCAATCTGAGCCAACAACCCCTGATCTGTGCCCC
CCTGAGTCTCAAAGACCTACAGTCCACTCATTCTGCAAGGTTTTAACTGCATCAGATACAA
GCACCCATGGAGGATTTTCTGTTCTTCGCAAACATGCTACTGATTGCCTTCCTAAACTGGA
CATGAATGATGCAACGCCAACCCAGGAATTGGTTGCAAAGGATCTTCATGGTTATGAGTG
GCGCTTTAAGCATATTTTCAGAGGCCAACCAAGGAGACATTTGCTTACAACAGGATGGAG
TACGTTTGTTACTTCAAAGAGATTAGTAGCTGGTGACTCCTTTGTGTTTTTGAGAGGGGAA
AATGGTGAGCTACGTGTTGGAGTGAGGCGTGTTGTTCGCCAACATAGCATCATGCCGTC
GTCAGTGATTTCAAGTCAGAGCATGCATGTAGGAGTGCTTGCAACTGCATCTCATGCAGT
TTCAACACAAACCCTCTTTGTTGTCTACTATAAGCCAAGGACAAGTCAGTTCATCATTGGG
TTGAACAAATATTTAGAGGCTCTTAGTAATAAGTTTGCTGTTGGCATGAGATTTAAGATGAA
GTTTGAAGGGGAGGATTCTCCTGAGAGAAGGTTTTCTGGCACGATCGTGGGGGTTGAAG
ATTTCTCTCCTCTTTGGAAAGATTCAAAATGGCGATCATTGAAGGTACAATGGGATGAACC
TGCCTCTATCCCAAGGCCTGATAGGGTTTCACCGTGGGAGATAGAACCCTTTGCTGCTCC
CATTCCACCTTCTTTGACTCAACCAGTTGCAGCTAAGAACAAAAGGCCCCGGCCACCTGC
TGAAATTCCTGCCACGGATTTATCTTCAACGACATCAGCTCCTTATTCTGGAGTTACACATT
GCCATGATCTAACACCACAAAACATTGCTGCCGAACCAAAAGGAAATGAAAATCCTGTTAT
GGGGCACCACATGTGGACAGAGATGAGCAGTGGTTGCAGCTCTGTCGTGCAGACTCTG

AATAAAGGAAGTTGGTTATCTTCTCCTGGCACATGTGTTCCTCATCATCTGTTTCCTGAAG
CAAGAGAAGACAAAACGAGATTCTCTGCTTGGCCTGTTCCTTCGGGCTTTTCAAACCCAC
AGCTCAACGAAGATTCAACCTTTGATTCAACTGAAAAGGCTAAGAGATCTGAGACATCTG
CTAGCTGCCGATTGTTCGGAATTGATCTGATAAACCATTCTGCAAGCTCAACACCTTTGGA
GAGAACACCTGCACAACTTTCTACTATGATAATGGGTATAACTGAAGTACCAGGCCCGAGT
AATCTATCATCCACTTATTCTGATCAGAAGTCTGAAATTTCAAAAGATTCTAAAGAGAAGAA
GCTGGAACAATTACAGTTATCAGCAAAAGAGAACCAGAGCATGCAAAGTTGTTCTAGTTTT
ACAAGAAGTCGTACAAAGGTTCAGATGCAGGGTATAGCCGTAGGTCGTGCTGTGGATTTG
GCCATGTTTGAAGGGTATGATCAGCTTATAGATGAACTGGAGGAGATGTTTGATATTAAGG
GAGAGCTTCGCCCTAGGAATAAGTGGGAGATTGTCTACACTGATGATGAAGGCGATATGA
TGCTTGTAGGCGATGATCCATGGCCGGAATTTTGCAACATGGTAAGAAGAATATTTATTTGT
TCAAGCCAAGATGTGAAAAAGCTGAAGACGGGAAGCAAACTTCCCTTGGCTTCTATCGAA
GGTGAAGGGACGAATGGAGGATCAGCTCTAACTCTCTTTGCCTTCATGGAAGCTGCGAA
GTTTGCCGCCTATTGGTTACCTTTTTGTAGGAAGAACGGTATAACGACGAGGAGCCCCGA
CGAATATTTTGCATCCAATTGCTCAAGAACCATTGAACCTGGGAAGATTAGAAAAGAAAAC
GTTGTTGAAACCGGAGGAGTTTATGATGAGTACATAACCGACGATAAAGTTCATCAGGCTT
TCAGTAAATGGAGTACTGATCATGGATTTACCTGTCAAAACCATCCCTCTGATTCAGGTTTT
GTTGGAGAAAAACCAAGACAGAGACATTACAGGCCGTTGTTTGCCAAACCAGGTTTATGT
CTCTACACAGAAGAGCACCTCACATCTCCTCACCATTTCAAAACCGGTGCCCTTAATGTTC
GTGTTTCGGCTGTTATGACAAATGCGCCTATAATTTTAACCTTAAGAAACAAGAGGTGGCA
AATTGGTCTCCTTGAAGTGGCTTCTTCAAGATACAGTCCAATAACATTTGGTGTTAAAGCTA
TGGGGCTGTTCATGGGGTTAGGCTATTCAAGCTATGCATATTCTTCCATTTTGTCAATTCCA
ATCATTACATATTCCTTTGTCCCTCAACTTGCTCTCCTCAATGGTCTCAATATCTTCCCAAAT
GTCTCTGAGCCATGGTTTCTCCTTTACGTGTTTTTGTTTCTTGGAGCATATGGGCAAGATT
TCTTAGAGCACTGTGTTGGTGGGGGAACAATACAAAGATGGTGTAGTGATCAGAGAATGT
GGATGATCAAAGGGCTTTCATGTTTCTTGTTTGGGTTAACCGAGTTCTTGCTTAAATCCAT
TGGGATTCCAACACAGGGTTTCAATGTCACCAGAAAGGCAATCGACGATGAGCAAAGGA
AAAGATACGAGCAGGGGTTGTTTGAATTTGGGCAACAAGGAAATGGGGTTAATGCCATTG
TTGTTATCAGCTTTAATTACCTGCCGATTTATGAAGCTGTGGTATTGAGGAGTGATAAAGGG
AAAATGCCTATTAGAATTTCATTTACTGCAACAACTCTGGTATTGGCTGTTAATTGA

>GaARF11-3
ATGTCTTTGGGGTTTATAACTCGAGCCAACCACCCTGTTGCCTGTTTCGAAGCAGCCAAC
GAAAGAGACGTCAAAAATAACACCGTTAACCCCAACCACACTCATCCCAATGCCCCCCCA
TCATCCGTACCTAAAACGCCGTTGAGAAACGGCTTTCCATCAGTCGGAGAGAGTTCAATA
ATGGCGAATCGGGGAGGGGTGTTTTCACAGACAAATAATGTTTCCTCCGAAGGAAATGGA
GGGGATGATCTGTACATGGAGTTATGGAAGTTATGTGCGGGACCCCTGGTTGAGGCTCCT
CGAGCTAGAGAGAGAGTCTATTATTTTCCTCAAGGACATATGGAACAGTTGGAAGCGTCG
ACGAATCAGGAGTTGAATCAGAGGATTCCTTTGTTTAATCTTCCATCGAAGATACTTTGCA
GTGTTGTTCACATTCAGTTTCTGGCTGAACAAGAAACAGATGAAGTTTATGCACAAATAAC
TTTGATGCCGGAGCCAAATCAACCTGAGCCAACAACTCCTGATGCATGTCTTCCGGAGCC
TCCAAAACCTACTGTCCATTCTTTCTGCAAGGTTTTAACAGCCTCTGATACCAGCACTCAT
GGAGGTTTTTCTGTTCTTCGCAAACATGCCACTGAATGCCTTCCTCCACTGGACATGAAC
CAGGCAACGCCAACCCAGGAATTGGTTGCCAAGGATCTTCATGGCTATGAGTGGCGCTTT
AAGCATATTTTTAGAGGCCAGCCAAGGAGGCATTTGCTCACAACGGGATGGAGTACATTT
GTTACTTCAAAGAGATTAGTAGCTGGTGATTCCTTTGTGTTCTTGAGAGGGGAAAATGGG
GAGCTGCGCGTTGGAGTGAGGCGTGTTGCTCGTCAACAGAGCAGCATGCCTACATCAGT
GATTTCAAGTCAGAGCATGCATTTAGGAGTACTAGCAACTGCATCTCATGCCGTTTCAACA
CAAACCCTCTTTGTTGTCTACTATAAACCAAGGACAAGTCAGTTCATTATTGGGTTGAACA
AATATTTAGAGGCTCTGAGCAATAAGTTTACAGTTGGCATGAGGTTTAAGATGAGATTTGAA
GGAGAGGATTCTCCTGAGAGAAGGTTTTCTGGCACAATCGTTGGGGTTGAAGACTTTTCT
CCTTACTGGAAAGATTCAAAATGGCGTTCATTGAAGGTACAATGGGATGAACCTGCCTCTA
TACCAAGACCTGACAGGGTTTCACCATGGGAGATAGAACCCTTCGCTGCTCCTATTCCAC
CAACTCTGGATTTATCTTCAACAGCATCAGCTCCTTGGAATTCTGGAGTTATGCATACCCAT
GATCTAATGCGGCGTAACATTACTGCCGAAGCAAACAGAAATGAAAATCATGTCATATGGC
ATATGCAGACTGAAATGAGCAGCAGTTGCACCTCTGTCTTGAAGACTCAGAACGAAGGGA
GCTGGCTATCTTCTCCTTGCATGAGTGTTTCTAAGCATCGATTTCCTGATGCAAGAGATGA
TAGCAAATGTGCATCTGGTTGGCCTGTTCTTTCAGGATTGTCAAACCCACAAGTGAACAAT
GATTCAACCTTTGATCCAAAAGAAAAGGTAAAAAAATCTGAGACAGCTTCTAGCTGCCGAT
TGTTTGGCATTGAGTTGATAAACCACTCTGCTAGCTCAAGGCGATTGGAGAGGACACCTA

CCCAACTTTCTACTATGACTGCCAGTACAGCTGAAGGACATCATACCTTGTCACCTAATAAT
TCTTGCCAGAAGTCTGAAATTTCAAAAGATTCTAATGAGAAGCAGGAACAGTTACAATTAC
AAGCAAAAGAGATCCAGAGCAGGCAAAGTTGCCCTAGTTCTACAAGAAGTCGTACCAAG
GTCCAGATGCAGGGGGTAGCTGTCGGTCGCGCTGTGGACCTGGCCATGTTGGAAGGGT
ACGATCAGCTTATAGATGAACTAGAGGAGATGTTTGATATTAAGGGAGAGCTTCGCCCTAG
GAATAAGTGGGAGATTGTCTACACTGATGATGAAGGGGATATGATGCTTGTAGGCGATGA
CCCATGGCAGGAATTCTGTAGCATGGTAAGAAGAATATTTATCTGTTTAAGTCAGGATGTG
AAAAAACTGAGTACAGGAAGCAAACTTCCCATGGCTTGTATTGAAGGTGGTGAAGGAACT
GTGATAAGCTCAGAGTCAATTGAAAATTAA

>GaARF16-1
ATGAAGGAATCAGAGAAGAGCTTAGATCCACAGTTATGGCATGCATGTGCTGGGCCCATG
GTTCAAATCCCACCATTAAACTCCAAGGTCTTTTACTTCCCTCAAGGCCACGCTGAGCAC
TCACTCGCAGCCGTCGATTTCCCCTCGTCTCCGCCGGTCCCGGCTCTAGTTCTTTGCCG
AGTGGCTTCTCTCAAATTCATGGCGGATACCGAAACCGACGAGGTATACGCCAAGATCCT
CCTCATGCCGTTACCCAGCACAGAGCTCGACATAGAAAACGACACCGTTTTCGGCTCCGA
TAACGCGGAGAAGCCTGCTTCTTTCGCTAAGACATTGACCCAATCCGACGCCAACAACG
GCGGTGGGTTTTCGGTCCCGAGGTACTGCGCCGAAACCATTTTCCCGTCGTTGGATTAC
ACGGCGGACCCTCCTGTTCAAACCGTCGTCGCCGTGGATGTCCACGGCGAAACGTGGA
AGTTCAGGCATATTTATAGAGGGACCCCAAGGAGGCATTTATTGACTACGGGGTGGAGCA
CTTTCGTGAACCATAAGAAGCTCGTTGCCGGCGATTCCATCGTGTTCTTAAGGTCCGAAA
ACGGCGGCCTGTGCGTCGGGATCCGACGAGCCAAGCGTGGGACTGGAAACGGACCCG
AAGCTGGGTCTCCATTTTTGTCTTTCTTAAGGGAGGACGAGAGTAAGATGATGATGATGAA
CCGAAATGGGGATTGGAGAGGAAAGGGGAAACTGAAGGCGGAAGCTGTTTTACAGGCG
GCTACGCTGGCGGCCAGCGGTCAGCCGTTCGAGGTTGTTTATTACCCAAGAGCGAGCAC
ACCGGAGTTTTGCGTTAAGGCGTCGTCGGTGAAGGCGGCAATGAGGGTTCCTTGGTGTT
GTGGGATGAGGTTTAAGATGGCTTTCGAGACAGAAGATTCTTCAAGGATTAGTTGGTTCAT
GGGGACTCTGTCTTCAGTTCAAGTTGTCGATCCCATTCGGTGGCCTAATTCCCCATGGCG
ACTTCTTCAGGTAACATGGGATGAACCTGATTTGCTTCAAAACGTTAAACGTGTTAGTCCT
TGGTTGGTTGAATTGGTATCGAATGTGCCTGCCATCCACTTGTCGCCGTTCTCCCCACCG
AGAAAGAAGTCTAGGTTTCCCCAACATCTCGATTTTCCTCTCGACGGACAATTTCCTATGT
CGGCATTTTCAAGCAATGGCAATCCCCATGGGACCGGCTGTCCGTTAGTTTTATCTGATAA
TGCTCCTGCAGGCATACAGGGAGCCAGGCATGCTCAATTTGGGTTATCTTTATCCGACCT
CCATCTTAATAATAAACTGCAGTCCGGACTTTTTCTGCCCGGTTTCCAGCGGTTCGATCCC
CACTCTAGAATTTCCGATGGCATCATGATGGCAAGGCGCCCTAATGGTACTGATAATCTTT
CTTGCTTGTTAACAATTGGGAATTCTAATGTGAATGAGAAATCTGGCAACACAAAAAGACA
CCAGTTTTTACTCTTTGGTCAGCCGATACTTACTGAGCAACAGCTCTCTCGTAGCTGTTCA
AGTGAAGTTGTCTCACAAGTTATTAACGGAAATAGTTCGTTAGATGGAAGTGCCGAAAAAA
CAAAAGATACTTCTGATGGTTCTCGATCTTCTCTTGAGAAGTCATCTACTGCTGGATTTTTG
TGGCACCAGGATTATAGAAGCACGGAAACCGGCCTCGATATTGGCCATTGCAAGGTATTC
TTGGACTCGGAGGATGTCGGACGAACTCTTGACCTCTCAGTTCTTGGCTCTTATGAAGAG
CTGTACAGGAGATTGGCCAACATGTTTGGAATAGAAAGATCCAAGATGTTGGGCCATGTG
TTGTATCGAGATGCAACAGGTGCTGTCAAACAAACTGGAGAAGAACCATTCAGTGCATTTA
TGAAAACAGCAAAAAGATTGACAATAAGGATGGATTCAAGCAATGAAACTGTTGCAAGGT
CTTGGCTTACCGGGATTCGAACAGCCGAAAACGGGCTAGGAGGGCCGAGCAAAAGAGG
TCCCTTAAGCATATTTGCGTGA

>GaARF16-2
ATGGAGAAATGCTTGGATTCTCAGCTATGGCATGCTTGTGCTGGCGGAATGGTTCAAATG
CCTTCAGTTAACACCAAAGTCTTTTACTTTCCTCAAGGCCATTCCGAGCACGCTTGTGGC
ACTGTTGATTTCAGGAACTGTCCTCGAATACAAGCTTATATACTCTGCAGAGTCGCCGCCG
TTAAGTTCATGGCCGACCCTGAAACGGACGAGGTTTTCGCCAAAATCAGGTTGATCCCAG
TTAACACCAATGACCCTGATTTTGAAGATGATGGAATTGGAAGCATTAATGGGAATGAAAC
ACAAGAAAAACCGGCTTCCTTTGCAAAGACATTGACTCAATCAGATGCTAACAATGGTGG
GGGATTTTCAGTTCCAAGGTACTGTGCTGAAACGATATTTCCAAGATTGGATTATTCTGCT
GATCCCCCGGTTCAGACCATTCTGGCTAAGGATGTCCATGGGGAAACCTGGAAGTTTAG
GCATATTTACAGGGGTACCCCAAGGAGACATCTTTTGACTACGGGCTGGAGTACGTTTGT
CAACCATAAGAAGCTTGTAGCTGGTGACTCGATTGTGTTTCTTAGGGCGGAAAATGGGGA
TCTCTGCATCGGAATTAGGAGGGCGAAGAGGGGGATTGGTGGAGGACCCGAGTCTTCAA

CCGGATGGAATGCTACTGCTGGAAATTGTATGATCCCGTATGGGGGGTTTTCAGCATTTCT
GAGGGAAGATGAGGGTAAGCTGATGAGAAATGGAAGCGGTAATGGGGTTAGCTCAAATG
GTAATTTGATGGGGAAGAGGAAGGTTAGGCCTGAACAAGTTATTGAAACTGCAACACTTG
CTTCCAATGGCCAACAATTCGAGGTCGTTTACTACCCAAGGGCGAGTACTCCTGAGTTCT
GCGTGAAGGCCTCTTTAGTGAAGGCTGCATTGCAGATCCGGTGGTGCTCAGGAATGAGG
TTCAAAATGGCATTCGAAACAGAGGATTCTTCTCGGATTAGTTGGTTTATGGGCACTATATC
TTCAGTTCAAGTCGCGGATCCCCTCCACTGGCCTGACTCACCTTGGAGGCTTCTTCAGG
TTACATGGGACGAGCCAGATTTGCTTCAGAATGTAAAACGAGTTAGTCCTTGGCTGGTGG
AATTGGTTTCGAACATGCCCGCGATTCATCTATCTCCCTTTTCACCACCAAGGAAAAAGTT
GAGACTGCCGCAGCACTCGGATTTTCACCTAGATGGTCAACTTCCAATGCCAACATTTTC
AGGCAACCTCCTTGGGGCCAGCAACCCCTTTGGGTGTTTACCCAACACTGCTCCTGCTG
GCATGCAGGGAGCCAGGCATGCTCATTACGGTCTATCTTTATCAGATCTCCACCTCAATAA
ACTGCAGTCAGGTCTCTTTCCGGCTAGTTTCCCACCACTTGATTATGCTCCTGTACTTAAT
AGGACCTCCAATGGTGGCCCAAACATTCGAAAGCCTAGCATGAGTGAGAACGTTTCTTGT
GTGCTAACCATGTCCCATCCCTCACAGAATTCCAAGAAAGCTGATGGTGCAAAGACACCT
CAGCTTGTACTTTTCGGTCGGCCGATTCTTACTGAGCAGCAGATCTCTCTCAGCTGCTCT
GGTGATACAAGCTCACCAGTTCTTACTGGAAATAGTTCTTCAGAATATCTGGATAAGGCAG
CAAACTTTTCCGATGGTTCTGGATCTGCTCTTCATCAACAAGGCCTTCCTGAGCGAGCAT
CCTGCGAAGGTCTCCCGTGGTACAAAAACAATCGCCAAGAAGCTGAACCCAATTTAGAGA
CTGGTCACTGTAAAGTTTTCATGCAATCAGAGGATGTTGGTCGTACGCTCAACCTTTCTTT
GCTGGGGTCTTATGATGAACTGCAGAGAAAGTTGGCAGACATGTTTGGTATAGAAAACTC
CGAAACTCTGAGCCATTTACTCTATCGGGATGCTACGGGTGCTGTCAAACAAATCGGAGA
AGAACCATTCAGTGACTTTATGAAAACCGCGAGGAGATTAACGATTCTAACGGATTCAAGC
AGCGACAACGTAGGAGAATATAGAGGAAGAAGCAAATCTTGA

>GaARF17-1
ATGCCGCCTTCACGGTCTAGTCCTCCGGAGCTTCGTCATGTTGATCCAAGGATCTGGCGA
GCTTGTGCCGGCTCTTCCGTTCAGATCCCCACCGTTAATTCTAGGGTTTATTACTTCCCTC
AAGGCCACCTTGAACAAGTTTGCGGTTCCACTCCCAAACTGTCCTCTCTCGTACTGTCTA
GGCCTCTCATCAATTGCGTTATCTCCGACGTTCATTATCTTGCCGATCCGAAAACCGATGA
GGTCTTCGCTAAGCTTTTACTCACCCCTGTTGAAACTTCTAGGCTTCCTAATCAATTTCTAA
ATGTGAATGGTGAAGTTGAGGATTCTGATAGGAATAAGATCGTGTCATTTGCTAAGGTTTTA
ACGCCCTCTGATGCCAACAACGGCGGTGGATTCTCCGTCCCGCGGTTTTGTGCCGATTC
TGTTTTCCCGCCGCTCGACTACAATGCTGATCCGCCGGTTCAGACTCTCTCCGTCACCGA
CGTTCGCGGCGGTGTTTGGGAGTTTCGTCACATTTATCGAGGGACGCCACGTAGGCATC
TGCTCACTACGGGATGGAGCAAGTTCGTTAATCAAAAGAAGCTTATCGCCGGCGATTCTG
TTGTTTTCATGAGGGACTGTAATTGGAAAATGTTTATTGGAGTCCGGCGAGCAATGAAGG
CGGGAGAAGGCGGAGGGGATTCGGGGAGGTGGAGAGAGCCGAGTGATGGAGGAGCAA
TGAAAGGGGAAGGAAGGGGGAGGATGACGGCGGAGGCAGTGGCTGAAGCGGCGGAGA
AGGCAGTGAGGGGGTTCCCGTTCGAGGTTGTGTATTATCCTCGGGCCGGTTGGACTGAT
TTCGTGGCGAGGGCGGTGTTGGTGGAGGCTGGAATCAGTACTTACTGGGCTGCGGGGA
CCAGAGTGAAGATGGCAGTTGAGACGGAGGACTCCTCGCGATTGGCTTGGTTTCAAGGG
ACAGTTATGTCTGCTGCTGTACCTGATTCTGGCCCCTGGATTGGCTCGCCTTGGCGAATG
CTTCAGGTTGCTTGGGATGAACCTGAAGTTCTCCAGAATGCAAGGAAAGTGAACCCATG
GCAAGTTCAAATTTCTTCCTCCTCACCGCTTCATTCCTCGTTCCCCTCGGAAAAGAGGCT
GAAGTTTTCTCAGGATTCAGGGCTGGCTGATGCAGAGGGTGAAATCTTTCCTGTGTCAG
GGTTAACTAATTCAACAATGGGGTATTTGAATCCATCACTGTTGAATTACAATTCTTTTCCT
GCTGGCATGCAGGGAGCCAGGCAATATCATTTTCACTTGCAAAGTTTAACCAACGATGTG
AGTGAGAATACCCCAGTGATGTCCACTGATAACTTCTCTGGCAACTATGTGGTACCCAAG
CCAACTAGAATATCCACTGAGCTCAATATTGGCAGTTCACAGTCTGAAAACTTGTCACCTG
ATAGTCAGAGCAGCATGGTTTCCTTTGGCACAGAACTTATTGAACATGGGGGTTGCAACT
CAAGCAAAGTAGGTGTTGGTTCGTTTCGATTGTTTGGCAAGATAATTGCTTTGAAAGAGCC
TGTCAGAAGCAGATTTGATGATGTTGGTTGCATGGATGATGTTGGAGGTAAAAGGCATGAT
GAAGCTGTGAGTGAAAAGAACTCCTTAGATCTTTCATTGACTTATGGCTGTTCGAAGCTGC
TTGACAGGCTAGATGTCCAATGCCAACGAGCCTCAACTTTTGATGGCTTCTCCTTGTGA

>GaARF17-2
ATGCCGCCTCCACGGCCGCCGGCTGCGGAGGTTCGCCACGTCAATTTAAGGATCTGGC
GAGCTTGCGCTGGCTCTTCCGTTCAAATCCCCACTGTTAACTCTGTTGTTTACTACTTCCC
TCAAGGACACGTGGAACAATCTTGCGGTTCGACTCCGTTACTCTCTTCTCTCGTGCTCTC

TAGGCCTCTCATCCCTTGCGTTGTCTCCGACGTTCATTGCCTCGCCGATCCCAGAACTGA
TGAAGTCTTCATCAAACTCTTCCTCGTCCCTGTCGAGCCTCCTAGACTACCCAATCAATTT
CTAGACGTTAATGGTGAAGTTGAATATCCTGATAAGGTCGTGTCCTTCGCCAAGATTTTAA
CGCCGTCCGATGCCAACAATGGCGGCGGATTCTCCGTCCCCCGGTTTTGTGCTGACTCT
ATTTTCCCGCCTCTTGACTACAATGCTGACCCGCCTGTTCAGACTCTCACCGTCACCGAC
GTTCGCGGCGGGGTTTGGGAGTTCCGCCACATTTACAGAGGAACGCCGCGTCGGCATC
TGCTCACTACGGGATGGAACAAATTTGTTAATCAAAAGAAGCTCATCGCCGGCGATTCTG
TTGTCTTCATGAGGGACTGTAATGGTAAAATGTTTATTGGAGTCCGTCGGGCTTTGAAGAG
AGGAGAAGGCGGTGGGGATTCCGGGAGGTGGAGAGAGCCAACTGGTGGAGGAGAAAC
GAAAGGGGACGGAAGGGGGAGGATGACGGCGGAGGTGGTTGTGGAAGTGGCGGAGAG
GGCCGCGAAGCGGTTGCCATTCGAGGTTGTGTACTATCCTCGGCCCGGTTGGACAGATT
TCGTGGTGAGAGCGGAGTTGGTGGAGGCTGGACTCAATATATACTGGGCTGGTGGGACC
AGAGTGAAGATGGTAGTTGAAACAGAGGATTCCTCGAGAATGACGTGCTTTCAAGGGAC
GGTTATTTCTGGTGCTTTGTCGGATTCCGGCCCCTGGATTGGCTCTCCTTGGCGAATGCT
TCTGGTTGCATGGGATGAACCTGATCTCCAGAATGTAAGGAGAGTGAACCCATGGCAAGT
TGAAATTGCTACTTCCTTACCGCTTCAGTCTTCATTTCCCTTGGCGAAGAAGTCCAAGTTT
TCTCAGGAATCTGGGCTGGCTGATGCAGAAGGAGAAATAATGTTCCCTATGACAGGGTTA
ACCAATTCAACAATGAGGTACATGAATCCATCAATGTTGAATTACAATTCTTTTCCTGCTGG
CATGCAGGGAGCCAGGCAAAATCATTTTCACGTGCAAGGTTTAACGAACCATGTGAGTGA
GAATACCCCAATGATGTCCACTGATGCTTCCTCTAGCAACTATTGGGTGCCCAAGTTAAAG
AGGATATCCACTGAGCTTAACATTGGCAGTTCACAGTCTGACAACTTGTCCCCAGATAGC
CAGAGCAGCATGGTATCCTTTGGCACTGAATTTACTGAAAATGCAGGCTGCAACTTGAGC
AAAGTAGGTGTTAATTCGTTTCAATTGTTTGGCAAGACGATTCATATGAAGGAGCCTGGTG
GAAGCATGTTTGGTAATGTTGGTAGCATGGAAGATGATAGTGGTAAAAGATATGATGAAGC
TGTGAGTGAAAAGAACTCGTTAGATCTTTCATCAACTAACAACTATTCAAAGCTGCATGAC
AGGATAGATGTCAAAAGTGAGAGTGCTTCAGCTTTCAAAGGCTTTTCTTTGTGA

>GaARF17-6
ATGGAGCTTGAAAAAAGCTTAGACCCAAATCTATGGCATGCATGTGCTGGATCCATGGTTC
AAATCCCACCAATAAACTCCAAAGTCTATTATTTCCCTCAAGGCCATGCCGAGCACTCACT
TAACTCGGTGGATTTCCCGTTTTCTCGTCAAATCCCACCTCTTGTTCTTTGCCGAGTTGAC
TCGGTCAAATTCATGGCCGATGTTGAAACCGATGAAGTATATGCCAAGATCATGCTTACGC
CATTACAGAACAACAAGCCTGATGTTGGAGACGATGATGGTGTTTCAAATGGCGGCTGTA
ATAATATTGAAAAAAAGGCTGCTTCTTTTGCTAAGACGTTGACTCAATCCGATGCTAACAAC
GGCGGTGGGTTTTCCGTTCCGAGATACTGTGCTGAAACCATTTTTCCCGCGTTGGATTAT
AGTGCCGATCCTCCGGTTCAAACCGTCGTCGCCGTTGATGTTCATGGTCAGCTATGGAAG
TTCAGGCATATTTATAGGGGAACTCCGAGGAGACATTTGTTGACTACAGGTTGGAGTGCTT
TCGTCAACCGGAAAAAACTCGTTGCCGGCGACTCAGTCGTGTTCTTGAGAGCCGAGAAC
GGTGGAATTCGTGTCGGGATCAGGCGAGCCAAGCTTGGGATTGGGGATGGTGATTGTTA
TGGAAGTTTGGGTTTTTATGGGGATTCAAAAAGGAACGGAAAAGTGAAGCCGGAGGAGG
TTTTGGAGGCGGCGAAGGCGGCTTCCGGTGGCCGGAAGTCGTTCGAGGTTGTTTATTAT
CCGAGAACAAGCAGGCCGGAGTTTTGTGTTAGGGCATCTGCAGTGAATACAGCAATGAA
GGTCCCTTGGTGTTGTGGCATGAGGTTCAAAATGGCTTTCGAGACTGAAGATTCTTCTAG
AATTAGTTGGTTCGTCGGAACCATATCGTCCGTTCAAGTTGCCGATCCGGTCCGGTGGCC
GGACTCGCCGTGGCGGCTTCTTGAGGTGACATGGGATGAACCGGATTTGCTGCAAAACG
TTAACCGTGTGAATCCGTGGTCAGTTGAATTAGCATCGAACGTGCCCGCTATCCACCTCT
CGCCCTTTTTACGACCGGTGAAGAAATTGCGGCTCCCTCAACACCTTGACGGACTATTAG
TGCAGTCTTTTCCAAGCAAGCCCCTTAGCCCAAGCAACCCGTTTTATCGTTTATCTGATAA
TGCTCCTGTAGGGATACAGGGAGCCAGGCAAGTTCAATTCGGGTTACCTTTATCGGATCT
CCATCTTAAAAATAACCTGAATTCGGGACTGTGCCTGTCTGGTTTCCAGCGGTTTGATCTA
CACGATAGAATTCCCGATACATTCACAAACAGTTGCAATAATCTATCTTGTAATAAATCCAAA
ATCCCGAAAAAATGCCGGTTTTTACTCTTTGGTCAGCCGATACTTACTGAGCAGCAACTGT
CTCAGAGCTCTTCAAGTGAAGCTGTCTCGGAAGTTGTTAGTGGAAACAGCTGCAAAACAA
AAGATTCATCCAACGGCTCAGGATCTGTTATCGGGAACCAATTCTCTCCGGAGAAGTCAT
CTACTGCTCGATTTTTGTGGGATTGGGACAATCGAGCCATAGAACCCTGCATGGATACCG
GTCATTGCAAGGTATTCATGGAATCGGAGGATGTCGGACGAACTCTTGACCTCTCGGTTC
TCGATTCTTATGAAGATTTATACAGGAGATTGGCTAACATGTTCGGAATAGAAAGATCGAAA
ATGTCCGGACATGTGCTGTATCGAGACGCAACCGGTGACGTCAAACGAACTGGAGATGA
ACCATTCAGTGCATTTATGAAGATCGCCAAAAGATTAACAATAAGAATGGATTCAAGCAACA
ATACTATTGAAAGGTCACGGCTCACCGGAACCCGAGCTGCGGAAAATGGATTTCATGGAT

CAAACAAGACAGGTCCTTTAAGCATATTTGCATGA

>GaARF18-1
ATGGCTCATTTAGAAGGTAATCAAAGGGGTTCCTCGACTGCTCATGCGGTTTCAGGTTCG
GCTGGTGATGATCTATATCAAGAATTATGGAAGCTATGTGCAGGCCCCTTGGTGGAGATTC
CTCGGGTTCACGAGAGAGTTTTTTACTTCCCTCAGGGTCACATGGAACAATTAGAAGCAT
CGACAAATCAGGAACTTAGCAATCAAACCCCACTGTTTAATCTTCCTTCTAAGATCCTATGT
CGTGTTCTTCACGTGGAGTTACTGGCAGAACAAGAGACAGATGAAGTTTATGCTCAGATC
ACTTTGCAGCCTGAAGATCAAAGTGAGCCTACAAGTCTTGATCCCTTCCCAACCGAGGCT
CCAAAGAGGACAGTTCATTCCTTTAGTAAGATTCTAACGGCATCAGACACTAGCACTCATG
GAGGGTTCTCTGTTCTCCGAAAGCATGCAACTGAGTGCCTTCCTCCTTTGGACATGAATC
TAGCAACTCCAACTCAGGAGTTGGTTGCCAAAGATCTTCACGGGTATGAGTGGCGGTTCA
AGCATATATTTAGAGGGCAACCACGGAGACATTTACTTACAACAGGGTGGAGCACTTTTGT
GACTTCGAAGAGATTAGTTGCTGGAGATGCATTTGTGTTTCTTAGAGGTGATAATGGAGAA
CTAAGAGTTGGGGTTCGGCGGCTTGCTCGTCAACAGAGCACAATGCCTTCGTCTGTGATA
TCCAGCCAGAGCATGCATTTAGGAGTGCTTGCTACTGCTGCTCATGCTGTTACAACTCATA
CCCTCTTTGTTGTGTATTACAAACCAAGTACAAGCCAATTCATAATTGGAGTAAACAAGTAT
CTGGAGGCTATTAACAATGGATTCTCTGTTGGCATGCGTTTCAAGATGAGATTTGAGGGA
GAAGACTCTCCTGAAAGAAGGTTCACAGGTACCATAGTTGGGGTTGGAGATATTTCCCCA
CATTGGTCAGAATCTAAATGGCGGTCCTTGAAGATTCAATGGGATGAACCTGCAGCAATAC
AAAGGCCAGAGAGAGTTTCTCCTTGGGAAATAGAGCCATTTGTAGATTCTGCTTCTGCAA
ATTTACAACCTACTATAATGTGCAAAAGACCAAGACCTGTCGATATTCCAGCTTCTGAAATT
ACTACCGGTTCAGCTGGTTCAGCCTTCTGGTGCCGTGAGTCAATCCAGTCTCATGAACTA
ACACCAGTGGGAAGCACACTTGAGGTCCAGAGCAGTGAAAACCAGGTTATGTGTCCTAT
GAGGCAGAAAGAAGCTGATGATTGTCTTATCAATGGTAATGGAGGTTACAAATCAAGGACT
CCACCTGAAAATGCCTGGCCACCTTCTCCTCTTGTGAATGTATCTTTGAACCTTTTTCCTG
ATTCAATGGAGAACAACTACAAAACAGGAGCATTGCAAACCGCTCTCACTGGTTATGCCA
AGAGCCTAATGCATGACCAAGTTGTAAAAAGAAAAACTGAGACTTTCACAGGTTGCCGGT
TGTTTGGGTTTAATTTGACAGATAGCACTAGTGTAGCTGCCCCCCCTGACAAGGAACAAAT
GAGCACAAGTATTGACTACAATGGTGTGAGAGGGCCTATCCCTGCTGCGTTTCATGTTGA
ACAGAAACCAGAAACTTCCAAGGAGCAGAAGCAAGTTGCATCGGAGACATCAACCCAGG
AGATGCAAGCTAAGCAGGGTTCTGCAACTTCCATGAGAAGTCGTACCAAGGTACATATGC
AAGGGATTGCAGTTGGCCGTGCTGTTGACTTAACTGCACTGAAAGGATATAATGATCTCAT
AAATGAGCTGGAAAAAATGTTTGAGATCAAGGGAGAGCTTTGTCGTAGTGGTCAGTGGTC
CATTGTTTTTACTGATGATGAGGGTGATATGATGCTCGTGGGTGATGATCCCTGGGTTGAA
TTTTGTAAGATGGTAAGAAAGATCTTCATATATTCAAGCGAGGAGGTGAAGAAGATTAGTAC
AAGATGCAAATTTCCAGCAGCATCTATGGAGTGTGAAGGGACGGTTGTAAGCTTGGACTC
AGAGCATAGGTCTGTTTGA

>GaARF18-4
ATGGCTCAACCAGAAGGTATTCCAAGGGAAACAGGTTTGGGTGTTGATGATCTGTATCCA
GAACTATGGAAATTATGTGCAGGCCCTTTAGTGGAGATTCCTCATGTTCAAGAAAGAGTCT
TTTATTTCCCTCAGGGTCACATTGAACAATTAGAAGCATCAACTAATCAAGAACTTAACTAT
GAAGCCCCACTGTTTAATCTTTCTTCAAAGATTCTATGTCGTGTTCTTCATGTCCAGTTACT
GGCTGAACAAGAAACAGATGAGGTTTATGCACAAATAACTTTACAGCCTGAACCAGATCAA
AGTGAACTGACCAGTCCGGATCCGTTCCCGACCGAGGTTCCGAAGAGGGAAGTTCATTC
CTTTTGTAAGATTTTAACTGCGTCGGATTCGAGTACTCATGGAGGGTTTTCCGTTCTACGG
AAACATGCCACCGAGTGCTTGCCTCCATTGGATACAAATCAAGCTACCCCGACACAAGAA
TTAACCGCGAAAGATCTTCACGGATATGAGTGGCGGTTTAAGCATATATTTAGGGGACAAC
CTCGGAGACATTTACTTACGACGGGGTGGAGCACATTTGTGACTTCGAAAAGATTGGTTG
CGGGAGACGCATTCGTGTTTCTAAGAGGTGATAACGGAGAATTAAGAGTTGGGGTTCGC
CGGCTCGGTAATCAACAAAGCACAATGCCTTCGTCGGTCATATCAAGCCAAAGCATGCAT
TTAGGAGTGCTCGCTACTGCTGCTCATGCTGTTACGACTCAGACCCTCTTTGTTGTGTACT
ACAAGCCAAGGACGAGCCAATTCATAATAGGAGTAAGTAAGTATCTCGAAGCTATTAACAA
CAGATTCTCCGTTGGTATGCGTTTCAAGATGGGATTCGAGGGCGAAGATTCCCCCGAGA
GAAGGTTCACTGGTACCATAGTCGGGGTTGGAGATGCTTCCCCACATTGGTCAGAATCTA
AATGGCGGTCCTTGAAGATTCAATGGGATGAACCTGCAATGATACAAAGGCCGGAACGAG
TTTCCCCTTGGGAAATAAAGCCATTCTCGGCTTCTGCTTCTATAAACCTCATACAACCAGC
TGTAAAGAGCAAAAGACCACGACCGGTTTATATTCCAGCTTCTGAAACAACTACAAATTCA
ACCGGTTCAGCCTTTTGGTGTCACGGGTCAACCAAATCTCATGAACTAGCCCGAGCAGG

AAGCATAGCTGAAGTCCAAAGCAGTGAAAGCAGCCAAGTTGTTTGGCCTATGAGACAGAA
TGAAGCCGATGCAGGTTATAACATGAGGACTCTGCTCGAAAATGCTTGGCCACCTTCCTC
TCTCGTGAACGTCTCTCTAAACTTTTTCCGCAATCCAGCGGATGCGTCTCCTGTTGAATTG
AGGACAAGCAAGGACGTTATGCGCGACCAAGTTGAAAAAGGAAAGAAACTCGAGATTTC
CACAGGCTGCCGTTTGTTCGGGTTCAATTTGACAAATAGTAATAGTGCAGTCAGTGGAAC
CGTTATAGCTCCGTCCCATATTGATGAAAATCCCGAAACTTTTCAGTCACCAAAGCAGCAA
AAGCAAAATGCATCAGAGACATCAACCAAGGAGATAAAGGCTAAGCACGGTACCAGTTCT
TCTACAAGAACTCGTACTAAGGTTCAAATGCAAGGGATTGCAGTCGGTCGTGCTATTGAC
TTAACCCTATTAAAAGGATACGATGACCTCATAAATGAGCTAGAGAAAATGTTCGATATTGA
GGGAGAGCTTCGTCACCGTACTAAATGGTCTGTCGTTTTTACTGACAATGAAAGTGATATG
ATGCTTGTGGGTGACGATCCTTGGGTGGAATTTTGTAAGATGGTGAGAAAGATATTCATAT
ATTCGGTAGACGAGGTGAAGAAGATTAACGCGAGATGCAAATTTCAAGCCTCGTCTTTAG
AAGGCGAAGGCACTGTTGTAAGCTTGGGTTCAGAGCATAGGTCTGAAACATGA

>GaARF18-6
ATGGCTAATGCACAAGTTAATCCAAGGGGTTCTTCTATTGCTCACTCGGAGTCAGGTTCG
AGTGATGATGATCTATACGCAGAGCTATGGAAGTTATGCGCAGGCCCGTTGGTGGAGATT
CCTCGGAATCACGAGAGAGTGTTTTACTTCCCTCAGGGTCACATGGAACAATTAGAAGCA
TCCACTAATCAGGAACTTAATAATCAAGCCCCACTGTTTAATCTTTCTTCTAAGATCCTATGT
CGTGTTATTCATGTCCAGTTACTGGCAGAACAAGAGACTGATGACGTTTATGCTCAGATCA
CTTTGCAGCCTGAAGCAGACCAAAGTGAACCTACAAGTCCTGATCCTTGCCCAGCTGAG
GTTCCAAAGAGGACAGTTCATTCGTTTTGTAAGATTTTAACAGCATCGGATACGAGCACTC
ATGGAGGGTTCTCCGTTCTTCGAAAGCATGCTACTGAGTGCCTACCTCCTTTGGATATGAA
TCAGGCAACCCCTACTCAGGAGTTGGCTGCTAAAGATCTTCATGGATATGAATGGCGGTT
CAAGCATATATTTAGAGGACAACCACGGAGACATTTGCTTACAACAGGGTGGAGCACCTT
TGTCACTTCCAAGAGATTGGTTGCGGGAGATGCATTTGTGTTTCTCAGAGGTGATAATGG
AGAACTTAGAGTTGGGGCTCGACGGCTTGCTCGCCAACAGACCACCATGCCATCATCTG
TGATTTCCAGCCAGAGCATGCATTTAGGAGTGCTTGCTACTGCTGCTCATGCTGTTACAAC
GCAGACCCTCTTTGTTGTGTACTACAAGCCAAGGACGAGTCAATTCATAATAGGTGTTAAT
AAGTATTTGGAGGCCATTAAAAATGGATTCTCGGTTGGTATGCGTTTTAAGATGAGATTTGA
GGGAGAAGACACTCCTGATAGAAGGTTCACGGGTACCATAGTTGGGGTAGGAGATTTTTC
CCCACATTGGTCGGAATCTAAATGGCGGACCTTGAAGATTCAATGGGATGAACCTGCAAC
AATACAAAGGCCAGAGAGAGTTTCACCATGGGAAATAGAGCCATTTGCGCCTTCTGCTTC
CATAAATCTTGTACAACCATCTGTAAAGAACAAAAGGCCACGACCTGTTGATATTCCAGTTT
CTGAAATTACTACAAATTCAGCTGGTTCAACCTTCTGGGGTCGTGGTTCAACCCAATCTCA
TGAACTAACTCAAGTAGGAAGCACACCTGAAATCCAAAGCAGTGAAAGCCAAGTTATCTG
GGGTATGAGGCAGAAAGAAGCTGATTACAGTCGAGGTTATAATTCGAACGCCTGGCCACA
TTCTCCTCTTGTGAATGTCTCTTTAAACCTTTTTCCTAATTCAGTGAGTGACAAAAACAGAA
CTGAAAAACCACAAACCACTCTCACTGGTTATGCCTTGCCTTCTCTATCGAGGCCAAGTAA
GGGCCTGATGCATGACCAAGTTGAGAAAGGGAAAAAATCTGAGATTTCCACAGGTTGCC
GGTTGTTTGGGTTTAATTTGACAGACACCATTAGTGCAGTTATCCCGACTGACAAGGAACA
AACGAACACAACGGTTGATCACAATGGTGTTTGGGGGTATCTTGCAGCTGCGTCTCATAT
CGATCAGAATCCAGAAACAGCTAAACAGAAGCATGTTGCAGCAGAGGCCTCGTCCAAGG
AGATGCAGGCTAAGCAGGGTGCATCCACTACTTCAACCAGAAGTCGTACTAAGGTACAAA
TGCAAGGGATTGCGGTTGGTCGTGCTGTTGACTTAACTGTGTTGAAAGGATATGATGATC
TCATTAATGAAGTGGAGAAAATGTTTGATATCAAGGGAGAGCTTCGCCCCAGTGGTAAATG
GTCCGTTGTTTTCACTGATGATGAGGGTGATATGATGCTTGTTGGCGATGATCCATGGATG
GATTTTTGCAAGATGGTGAGAAAGATCTTCATATATTCAAGCGAAGAGGTGAATAAGATTAG
TCCAAGATGCAAATTTGCTGTGTCGTCTTTGGATGGTGAAGGGACTGCTGTAACCATTGA
TTCAGAGCATAAATCTGAAACATGA

>GaARF19-1
ATGATAACAGTCATGGATTCTAGGAAAGAAGTTGTGAAAAATTCAGAAAAATGCTTAGATC
CTCAGCTATGGCATGCTTGTGCTGGTGGTATGGTACAAATGCCATCAGTGAACTCTAAGG
TGTTCTATTTCCCTCAGGGTCATGCTGAGCATGCCAATGGGAATGTAGACTTTGGGAATCT
CCCGATTCCGTCGCTCGTCTTGTGTCGGGTTTCCGCCGTTAGGTTTATGGCGGATCCCG
AAACTGATGAGGTTTATGCTAAAATCATGTTGATTCCTTTGAGAGAGAATAGTTTTGGGGTT
GAAGATGATGGTTTTGATGGGAATGTTGGGGTGGAGAATCCTGAAAAATCTGCTTCCTTT
GCTAAGACATTGACTCAGTCCGATGCTAACAATGGTGGGGGGTTCTCGGTTCCGCGGTAT
TGCGCGGAGACTATATTTCCTAGGTTGGATTATAATGCTGAACCCCCTGTTCAGACTATTCT

TGCTAAGGATGTTCATGGTGAGGTTTGGAAATTTAGGCATATATATAGGGGGACGCCTCGT
CGACATCTTTTGACGACGGGGTGGAGTAATTTCGTCAACCACAAGAAGCTTGTGGCTGG
TGATTCGATTGTGTTTCTTAGAGCTGATAATGGAGATCTTTGTGTGGGGATCCGTAGGGCG
AAAAGAGGACTTGGGGGTGGACATGAATTTCCTGGATGGAACTCTGTGAGTGGAACCTC
TGGTTCACAAGTTGGGAGTTATTCTCCGTTTTTGAGGGAGGGGGAGAGTAAATTGATGAG
GAAGGATTGTAATGGGGATCCAAGGGGAAGAATAAGGGCTGACTCCGTGATTGAAGCTG
CAAGTCATGCAGCTAGTGGGCAGCCCTTTGAGGTCGTTTACTATCCACGAACTAGCACTC
CTGAGTTCTGCGTGAAGGCCTCATCCGTTAGAGCTGCAATGCAAATCCAGTGGTATCCTG
GGATGAGGTTCAAAATGCCTTTTGAAACTGAGGACTCTTCACGGATTAGCTGGTTCATGG
GAACGATATCTACTGCTCAGGTTGTCGATCCCATCCGTTGGCCTAATTCTCCATGGCGACT
ACTGCAGGTAGCATGGGATGAGCCAGACTTACTCCACAATGTGAAGCGTGTTAGCCCATG
GTTGGTTGAATTGGTCACAAACATACCAGCTATTAATCTTAATCCTTTCTCGCCCCCAAGG
AAAAAGATGCGGCTTCCACAACACCCAGACATTTCCTTTCTTAACCAAATTCCAATGCCAT
CATTTTCCGGGAACTCATTCAGATCCAGCAGCCCCATGCGTTGCATTACGGACAACATTC
CTGGAGGCATACAGGGAGCCAGGCATGAACCGTTCGGATTATCGTCATCAGATCTCCGCT
CCAACAAGCTGCATTCAGGTCTGTTTCCATCTGGTTTTCATCAACTTGATCGTACTGCCCC
ACCTACTAGACTTTCCGGCAACAACTTTTGTAGCGACAACGCCAACAATACAAATATATCTT
CCTTGTTGACAATAGGAAATCCAACCCAGAGTTTCAAACAAAGCAATGATAGCAAGACAC
CCCATATTGTATTGTTTGGTCAACTCATTTTCTGTGAGCAGCGGGCTTCTCAGAGCTGCTC
AGGTGATACGGTTGGAAACAGTTCATCAGATGGGAATACAGAGAAGACTGCTATTTCCTC
CGATGGAACTGGATCTGTGTTACATCAAAATGTTCGGGAAAATTCTTCGGATGAAGGGTTT
CCTTGGTGCAAAGAGGATCAAAAAACTGATCTAGGATTGGAGACCGGTCATTGCAAAGTG
TTTATGGAATCAGAGAATGTAGGTAGAACCCTTGATCTTTCCGTTCTCGGATCATATGAAGA
GCTGTATGGGAAGCTGGCCAACATGTTTGGCATAGAAAGTTCAGAGATGCTGAGCAGTGT
GCTCTACCGTGATGCTGCCGGTTCAGTTAAACACACTGGAGATGAGCCCTTCAGCGAGT
TTATGAAAACAGCAAGGAGGCTAACGATTCTCATGGATTCAAGCAGTGACAACTTAGAAA
GATAG

>GaARF19-3
ATGAAGGCTCCACCCAATGGATTTTTGGCAAATCCCGCAGAAGGAGAAAGGAAGAGTATC
AATTCAGAATTATGGCATGCTTGTGCTGGACCACTTGTTTCTTTACCGCCAGTTGGAAGTT
TGGTGGTTTACTTTCCTCAAGGCCATAGTGAACAAGTTGCAGCATCGATGCAGAAGGAGA
CTGATTTCGTACCAAGCTACCCTAATCTTCCTTCCAAGTTAATTTGCATGCTCCATAATGTC
ACATTGCATGTAAGCAAAAAACTAGCCAACCATTATCCCGAGCCGGAAACCGATGAGGTC
TATGCTCAGATGACTCTTCAACCTGTAAACAAATATGACAGAGAAGCATTACTAGCATCTGA
TATGGGCCTCAAGCAAAGCAGACAACCTGCTGAGTTCTTTTGCAAGACGCTTACAGCTAG
TGACACTAGCACTCATGGTGGCTTTTCAGTGCCTCGTCGAGCAGCTGAGAAGATCTTCCC
TCCTTTGGATTTTTCGATGCAACCACCCGCTCAAGAGCTACAAGCTAGAGATTTACATGAG
AATCCATGGACATTTAGACATATCTATCGAGGTATGATTCTCTTCATTGCTCTTCCGTTTCAT
GAGCTAGTAGCTAGTACCTCAATGCATAAGGCTTCTGGTCAACCAAAGAGGCATCTTCTAA
CTACTGGTTGGAGTGTCTTTGTTAGCAACAAAAGACTTTTTGCTGGTGATTCTGTTCTTTT
CATAAGAGACGAGAAGTCACAGTTTCTCTTAGGTATAAGGCGGGCCAATAGACAGCAGCC
AGCTCTCTCTTCATCAGTAATTTCTAGTGATAGCATGCATATAGGGATCCTTGCTGCTGCAG
CTCATGCTGCTGCAAATAACAGCCCATTTACCATATTCTACAATCCAAGATATTATAATTTTG
ATAGGGCAAGCCCTTCCGAGTTTGTGGTACCCTTGGCGAAGTATAACAAAGCCATGTATA
CCCAAGTTTCTCTTGGAATGCGGTTTAGAATGATGTTTGAAACCGAGGAGTGCGGAGTAC
GTAGATACATGGGTACGATTACTGGTATCAGTGACCTGGATCCTGTACGATGGAAGAATTC
ACAGTGGCGCAATCTTCAGGTAGGATGGGATGAATCTACTGCTGGTGAGCGACCAACCC
GAGTTTCAATTTGGGACATCGAGCCTGTTGTAACTCCTTTCTACATATGCCCACCACCGTT
TTTCAGGCCTAGATTCCCCAAGCAACCGGGAATGCCAGATAATGAAAATGATGTTGAGAAT
GCTTTTAAGAGAGCTATGCCTTGGCTTGGAGATGACTTTGGCATGAAAGATTCCCCCAATT
CAATCTTCCCTGGTTTGAGTTTAGTTCAGTGGATGAACATGCAACAAAATAATCAGTTTGC
AGCTGCTCAATCTGGACTCTTTCCTTCAATGGTTTCTTCGAATCCATTGCACAGTAGCCTT
GGCACTGATGATCCATCCAAGTTATTGAATTTTCAAGCTCCGATGTTACCTGCGTCAAATAT
GCAATTTAACAAAGCAAATCCAAACCAGGTCAACCAGTTGTCTCAGGCACCTATGACCTG
GCCCCAGCAACAGCAACTCCAGCAGTTATTGCAGACTCCTCTGAATCAAAATCCACAACA
GCAGTTACAGCAACGACAGTCACAGCTGCAGCCACAGTCACATCTTCAACAGCAGCCTC
AGCCTCAGCCACCTCTACAGCAGCAGCAGCAGCAACAACAAAGACAACAGGCACAGCC
ACAACCACAGCAGCAGCAGCCACCTCTACAGCAACTGCAACAACAACAAAGACAACAGG
CACAACCGCAGCTGCTGCAACAACAATTTCTACCAGCTCAAGTAAATAATGGCATCATTGC

CTCAAACCAGATGCCAAATCAAAATTTGCATCAGCCAGGTGTTTATTCTCAGATTCAGCAA
CAGCAGCAGCAACAACAAATATTGACAAGCAATGGCCAGTCAACTCAAACCACCCTTCCT
GCTAATAAGGCTTCATATCCTTTGACATCGTTAGCTCAAGATACACAGATTCAGCAACAGG
TGGAACAGCAACCTAACCTCGTACAGAGCCAACAGCAACAGACACAGTTGCAGCAAAAT
CTGTCCCAGAGGTCACAACAGCAGCCACAAATTCAACAACTGGCACAGCAGGGCCTCTC
AGAGCAACAATTCCAATTATTACAGAAATTGCAGCAACAGCAGCAGTCGTCTCAACAATTA
CTCTCACCTACTGGATCACTGTCGCAGTCTCCAGTGGTGCAACAACAGCCAATGCATCAA
CAAAACCAACCAATGCAGCAGGTGCCTCTTTCTCAGAGCCAACAGACACTTGGCAGCAA
TGGTTTCTCAACATCAATGCTAATGCAACCACAACAGCTAGTGGTGAGCCAATCCCAAAAT
CAGAACAAGCCACTTATGGCAATGAGAACCCATTCTGGTCTTACTGATGGAGATGCTCCAT
CATGTTCAACCTCACCTTCTACCAATAATTGTCAGGTTTCACCATCAAGCTTCCTAAGCAG
AAGTCAGCAAGTACCATCCATGGTAGTGACAGATCCAGTTGTTGAGCCTGCAAGTACACT
GGTTCAGGAGCTTCAGAGCAAGCCTGATATCCGAATCAAACACGAGTTGCTTGCCTCTAA
AGGTCCAGATCAATCAAAGTACAAAAGTACTGTGACAGACCAGTTAGAAGCATCCTCTTCT
GGAACATCATACTGCCTGGATGCAGGCACCATCCAGCATAATTTCTCTCTCCCCACCTTTT
TGGAAGGTGATGTCCAATCACATCCTCGGAGCAATCTTCAATTTTCAGCTAATATTGACGG
ACTGGCACCCGACACCTTGTTATCAAGGGGATATGACTCTCAAAAGGATCTTCAAAATCTG
CTTTCTAATTATGGTAGCAATCCAAGAGATATTGACACAGAGTTGTCTACTGCTGCAATAAG
TCCCCAGTCATTTGGTGTGCCAAACATACCTTTCAAGAGTGGGTGTTCAAATGATGTTGC
CATAAACGATGCAGGAGTTCTAAATGGTGGATTGTGGGCCAACCAAACTCAACGGATGCG
AACATATACAAAGGTGCAAAAGCGTGGCTCTGTGGGAAGATCAATCGATGTGACCCGCTA
CAAAGGGTATGATGAACTCAGGCATGATCTAGCCCGCATGTTTGGTATCGAGGGACAGAT
GGAAGATCCACAAAGTTCTGACTGGAAATTAGTATATGTTGACCATGAAAATGACATATTAC
TTGTTGGTGATGATCCTTGGGAAGAATTTGTAAGTTGTGTTCAGAGCATTAAAATTCTGTCA
TCCCTTGAAGTACAACAAATGAGCTTGGATGGTGACCTTGGAAATGTGCCAGTTCCCAAT
CAGGCTTGTAGTGAGATCGACAGTGGAAATGCATGGAGAGGACATTATGATGATACTTCA
GCCGCCTCTTTTAACAGA

>GaARF23-1
ATGATAACAGTAATGGATTGTAGGAAAGAAGTGGTTAAGACATCAGAAAAATGCTTAGATC
CTCAACTATGGCATGCTTGTGCTGGTAATATGGTGCAAATGCCAGCGGTGAACTCCAAGG
TGTTCTATTTCCCTCAAGGTCATGCTGAACATGCCAATAGGAATGTAGATTTTGGGAGTCT
TTTAATTCCTTCACTCATCCTATGTAGAATTTCGGCCATTAAGTTTATGGCAGATCCTGAAA
CTGATGAGGTTTATGCTAAAATTATGCTGGTTCCTTTGAGAGAGAATGATTTTGGGTATGAA
GATGGTTTTGATGGAAATATTGGGATGGAGAATCTTGAAAAACCACCTTCTTTTGCTAAGA
CATTGACTCAATCCGATGCTAATAATGGTGGGGGTTTCTCAGTTCCCCGGTATTGTGCAGA
GACTATATTTCCTAGGTTGGATTATAGTGCTGAACCCCCTGTTCAGACCATTCATGCAAAG
GATGTACATGGTAATATTTGGAAATTTAGGCATATATATAGGGGAACACCTCGCCGGCATCT
TTTGACGACGGGGTGGAGTAATTTTGTGAATCATAAGAAGCTTGTGGCTGGTGATTCGAT
TGTCTTTCTTCGAGCAGAGAATGGAGATCTCTGTGTGGGAATCCGTAGGGTGAAGAGAG
GTATTGGAAGTGGACATGAGTATCCTTCTAGTTGGAATTTGGGGGATGGAAGCTCTGGTTA
TTCTCCACTCTTGAGGGAGGGGGAGAGTAAATTGATGAAGAATGATTCGAATGGGGATCT
GAGGGGAAGAATAAGGCCCGAGAATGTGATTGAAGCTGTGAGTCGTGCCGCCAATAGGC
AACCCTTTGAGGTTGTTTACTATCCACGAGCAAGCACTCCTGAGTTCTGTGTGAAGGCCT
CATCAGTTAGAGCTGCAATGCAAACCCACTGGTATCCTGGGATGAGGTTCAAAATGGCTT
TTGAAACCGAGGACTCCTCACGGATTAGCTGGTTTATGGGAACAATATCTACCGCTCAGG
TCGTTGATCCCATCCGTTGGCCTAATTCTCCATGGCGCCTTCTTCAGGTGGCATGGGATG
AGCCAGATTTACTACACGATGTGAAGTGTGTTAGCCCATGGTTGGTTGAATTGGTGACAA
ACATACCAGCCATCCATCTTAATCATTTCTCGCCCCCAAGGAAAAGGATGTGGCTTCCGCA
ACACCCTGATTTGTCTTTACTTGGCCAAATTCCAATGCCATCTTTTTCTGACAGCAGTTTCA
GGTCCAGCAGCCCCGCATGTTGCATTACAAACAACATTCCTGGAGGCATACAGGGAGCC
AGGCATGCACCGTTTGGATTATCTTCATCAGATGTCCGTTCCAGCAAGCTGCAGTCGGGC
CTCTTTCCACATGATTTTAATCAGCTTGGTCATACTATCCCACCTATGGGACACTCCAGTGA
CTATGGAAACAATAGGAATATTTCTTCCTCGCATACAATGGGAAATCTTACCCAAAGTTTAA
AAGAAAGCAATGAAAAAAAGACACCCCATATCTTATTGTTTGGTCAACTCATTTTCTGTGA
GCAGCAGGTTTCTCAGAGCTGCTCAGATACAATTGGAAACAGTTCATCCAACGGGAATAC
AGAGAAGACTGTGATTTCTTCCGATGCCTCTGGATCTGCATTACATCAAAATGCTCATGAA
AATTCTTCAGATGAAGGGTCTCCTTGGTACAAAGAGCTCCCAAAAACCAACATGGGGTTG
GAGACTGGTCATAGCCAAGTCTTCACGGAATCAGAGAATGTGGGAAGAACCCTAGATCTT
TCAGCTCTCAGATCATATGAAGAGCTGCACGGCAAGCTAGCCAACATGTTTGGCGTAGAA

AGTTCAGATATGCTGAGAAACATGTTCTACCGAGATGCTGCTGCTTCTGTTAAGCACACAG
GAGATGAGCCCTTCAGTGAGTTTTTGAAGACAGCAAGGAGGCTGACCGTTCTTACAGATT
CAGGCAGTGACAACATGGGAAGATAG

>GaARF23-3
ATGGGGAAAAGCGAGTTGACAGATGGTTGCTTGAGTGATGATGCAGGTGAAGCGCCGGA
GAGAAGGCACATCAACCCGGAGCTATGGCAGGCGTGTGCTGGGCCGCTGGTGAACCTG
CCGGCTGCTGGGACCCATGTTGTCTACTTTCCTCAAGGCCACAGTGAACAGGTTGCAGC
ATCTATGAAGAGAGATGTGGATGCTCAAATTCCGAACTACCCAAATCTTCCTTCTAAGCTAT
TATGTCTCCTTCATAATGTCACCTTGCATGCAGACCCAGAGACAGATGAAGTCTATGCTCA
GATGACCCTCCAACCTGTTTCTTCGTTTGATAAGGAGGCGTTACTGAGATCAGATCTTTCT
CTGAAGTCAAATAAGCCACAACCTGAATTCTTCTGTAAGACGTTGACAGCAAGTGATACAA
GCACTCATGGAGGTTTTTCTGTTCCTCGACGTGCCGCCGAAAAGATTTTCCCTCCTCTTG
ATTTCTCGATGCAAACACCTGCTCAAGAACTTGTGGCCAGGGATCTGCATGAAAATGTCT
GGAAGTTCCGCCATATCTATCGTGGAAAACCAAAGCGCCACTTGCTTACAACAGGATGGA
GTCTATTTGTTAGTGGAAAGAGACTTTTTGCTGGTGATTCAGTTTTATTCATTAGAGATGAA
ACACAGCAGCTTCTCTTGGGTATAAGGCGTGCTAACAGGCAACCTGCCAATCTATCATCAT
CAGTACTGTCTAGTGATAGCATGCATATTGGCATCCTCGCTTCAGCTGCTCATGCAGCAGC
AAACAATAGCCCTTTTACCGTGTTTTATAACCCAAGGGCTAGCCTATCTGAATTTGTTATTC
CTTTAGCCAAGTACTATAAAGCAGTGTACAACCATCAAATATCACCTGGCATGCGCTTTCG
AATGATGTTTGAAACTGAAGAGTCAGGAACAAGAAGGTATATGGGTACAATTACAGGAATC
AGCGATATTGACCCTGTAAGATGGAAAAACTCACAATGGCGTAATTTGCAGGTTGGCTGG
GACGAGTCAACTGCTGGCGAAAGACGTAATAGAGTATCCATTTGGGAAATCGAACCAGTT
ACAGCTCCATTTTTCATCTGTCCATCTCCATTGTTCAGATCTAAGCGTCCTAGGCAACCTG
GAATGCTGGCTGATGAATACTCTGACTTAGATAATCTATTCAAGAGGCCAATGCCTTGGCT
TGGTGATGATATATGCCTGAAGGATTCCGATGCTCATCCAGGGCTTAGCTTGGTCCAGTG
GATGAACATGCAGCAAAATCCTCTACTGGCAAACTCTATGCAGCCAAATTTCATGCAGTCT
CTGGCTGGGTCTACTATGCAAAACTTTGACGGAGCAGATCTTTCCCACCAAATGGGCCTT
TCAGCACCACAAATGCCTCAGCCCAACAACTTACAGTTCAATGCTCATAGGCTACCTCAG
AAAGTGCAGCAACTTGATCAAGTTCCAAAGCTACCATCTACAATGAACTCACTGGGATCCA
TTATTCAGCCGCAACAGCTGAATGACATGACTCAGCAGTCAAGGCAAAATTTGGTTGCTC
AGACTCTACCCTCTAGTCAAGTTTTGCAGCCTCAAGCCCTTGTCCGAAGTAACAATATCCT
TCATCAGCAGCAAACATCTAATCCAACTCATCAACTCCCTCTAAGTCTTCCTCAAAACCTG
CAGCAGCAGCAGCAGTATCTTGTGGGCCCAAATCATCCGCAAAACCTAATGCATTCCCAG
CTGCCTGATCCACTCAACCAGCATTTACAAGTGCCTGACAACCAGGTCCAGTTTCAACTG
ATGCAGAAACTTCAGCAGCAACAACAGTTGCTTTTGGCACAGCAATCTGCACTTCAGCAG
CCTGGTCTACTTGCCCAACCCCAAGATCAACAAAGGCAGCTGTTAGATGCGTCTCAGAG
CTTCTCTAGTTCTGTGACAGCTAGCCAAGTGTTAGAGATGCCTCAAAACATACCTACCTTG
CTACCTCAATCTAATGTTGCCCCACAGCAGATGCCTAAAAATAACAGCCAGGCAAATGTTT
GGTTCTCTCAGCCGCCTCTGCAGTCAAAAGTTCAGCAACAACAAACTGGAATGCTACCTG
AAGTTCCTGGTCTTGTAGGTCCCTTCCAAACTACAGCAACAAATCAATTCTCCACAGCTGT
TAGTAGTGTAATGACGTCTGCTGCTGTAGCCGCACCTTCTGTGATTACTGATGATAATCCA
TCATGCTCCACTTCACCATCTACAAACTGTCCAAGTGTTCTTCAACCAATGATAGACAGCA
GAGTCCACAGGAGTGCTGGGTTAGGAGATGACATCAGTCAGTCTGCTGCCACAGTATTG
AATCCTAATGCCTTGGAGACAATGTCAACTAAGGCTAATATGGTTAAAGAACAGCAGCAAA
AGTCTGTTAAACCCTTGTTGAATATCTCCAAGAGTCAAAACCAAGGCTCTTTTGCCCCGCA
AAACTGTATCAATGGTGCTACAGCACATGCAGATTGTTTGGACACATCATCTTCTACAACTT
CAGTTTGCCTTTCTCAAAGTGATGCTCATTTGCACCAAAACACACTGTCTTACAACCCTCA
AACAATGTTGTTGAGAGATACAAGTCAAGAGGGAGAAGTTCGGGCATATCCAAGGAATAA
TGTTTCATATGGCAATAATATGGATAGCCAAATTGAGATGCCCATGAATTCTGACACTTTGT
CTGCGAAAGGCATGATGGGACTGGGGAAGGATTTTTCGAATCACCTCTCTTCGGGAGGG
ATACTCGCCAGCTATGAAAACCCTAAAGATGCTCAGCAAGAACTTTCTTCGTCAATGGTTT
CCCAGCCATATAGAGTTCCAGATATGGCGTTTAACTCCATTGATCCCACTATAAACCATAGC
AGCTTCATAAACCGCAATGCATGGACCCCACCATCACAATTTCAGAGATTGCGAACATATA
CCAAGGTTTACAAACGTGGAGCTGTTGGAAGATCAATAGATATAACTCGTTATTCAGGTTAT
GATGAGCTCAAACAAGATTTGGCTCGTAGGTTTGGAATTGAGGGGCAGCTGGAAGACCG
TGGGAGAGTAGGCTGGAAACTAGTCTATGTGGATCACGAGAACGATGTTCTGCTAGTAGG
AGATGACCCATGGGAGGAGTTTATCAACTGTGTCCGATGCATCAAAATACTCTCCCCTCA
GGAAGTCCAGCAGATGAGCATGGATGGAGAGTTTGGGAACTCTGTCCTGCCTAATCAAG
ACTGTAGCAGCTCTGGCAATGGGAATGCATAA

>GaARF24-1
ATGGACGGTGAACGGAATGGTTTGAAGGCTAAGGTCCCATTTCACCCCAGAAGACGTAG
CAACGTCTATGGCTTGCCGTTGCTTCCTCCGAACAATCAAGGAGAAAAAGATGATCTACAT
GTTCAACTATGGCATGCATGCGCTGGTCCTTCAGTTTATGTGCCACACGCTGGGGAAAAG
GTTCTGTATTTCCCTCAAGCTCACATAGAACAGGTGGATGCATGTATGAATCAAGATGGGA
TAATGGAAATGCCTATCTACAATTTGCCTTCTAAGATCCTTTGCAGGGTGATGCATGTTCAG
CTTAAGGTTGAACCTGGCACAGATGAGGTCTTTGCACAAATAACACTGATTCCAGAAGCA
GAGCAAGATGAGGAAAGTTTGGAGCATAGAAATTATCAACCTTTACCCCAGAAAGCTTATC
CAGTGTTCTTTAGTAAGAAACTCACTCCATCAGATACAAGCACACATGGCGGATTCTCTAT
CCCAAAGCGACATGTTGATGATGGGTGGATCCCACCCCTGGACATGTCTCAGCAAACCC
CACAACAGGAATTGGTTGCAATAGACTTGCACGGTTTTGAGTGGCGCTTTCGACATATTTA
TCGAGGTCAGCCAAAAAGGCACTTGCTTACAAGCGGCTGGAGTACATTTCTGACTTCAAA
GAAGCTTCTTGCTGGGGATGAATGTATCTTTCTTAGAGGGGAAAAAGGAGAGCTCCGTCT
TGGAATTCGGCGAGCAACGACTGTTCTGAAATATACATCAACATCCGTCATATCTGGTCAT
AGCATGCGTCATGGCATACTAGCAAGTGCTTTTCATGCCTTCTCTACCAGAAGCATGTTTA
ATGTCAACTACCGTCCTTGGTCTACTTCTTCTGAATTTATCATCTCATTAGATCGGTATATGA
AGTCGGCTCAAATTGACTATTGCATCGGGACAAGATTTAGAATGCGATTAGAAGGCGAAG
AATGTGCGGAACAAAGACCTTCTGGCACTATCATAAGCATTGAAGATGTGGATCATACTAG
GTGGCCTAATTCTGAATGGAGATGTCTGAAGGTGAAATGGGATCTCACAGCAGGTGAAAA
TTTTCATCCTGAAAGAGTTTGTCCTTGGAACATTGAACCAACGGAATTTAGTATCAAGAAG
AGACCCTCCATTCTACGTAACCAAAAGAAGGCTCGTACTGATGATGTATCATCCCCTGGGT
TTTCTACCCTGCTAATGGATGGCATGTGGAGTGGTTCCATTAAATACGAATCTCAAAGTAG
CTCAGGGGTCTTGCAAGGTCAAGAAGATAGTGACACAGATGGTAATCAACCCGATGCACT
AAGACAACCATTACCACATTGCCTCCCACTAAATCACAGTTGGGATTCAATGCAACAGCCA
ATACAGAATCAACGAGAGATTGGCGCAGCACCCTTTTCTGGTGGACAAGTAGAAAGTTTG
GGTCTTCATAATAGTTGGTCCACAACATTCTCCTCCTCAAATGGAGTACATGAAGATGCTAT
TGCTAGCAGAAAAATTTCAGTTCCAAATGTTAATTCTCAGGAATGGAGTATTTCAGAACCAA
GGAATGAAAATGAAACATCATGGTGCGAACCAAAGGAGGGACATGCATGCATGCTTTTTG
GAGTAAATTTAGTTACCGGTCCACTGGAGCCCCCTTCACCCCAACTTGTCACTTCTAGCG
AGCTTGAAAGTCATTGTTCTATTCCTCCAACTTCTCAGTCAACTGTTTCGAAACCTTCTAA
GGGTACATCTAGCAAGCAGTGTGACAACTGCTGTTCTGCTAGCAATTGGAGTTGCACCAA
GGTACTCAAGCATGGGACTGCTCTTGGAAGATCAGTTGATATCACTAGATTTGACGGATAT
AAAGACCTCATCTCTGAGCTTGATCGCATGTTTGATTTTAATGGACGATTGATTGATGGAA
GCAGCGGCTGGCATGTAACCTTTACTGATGATATAGGGGAGATGAGGATGATTGGAGATC
ATTACCCATGGCAGAAATTTCAGAATGAGGTCCGAAGGATGCTGATCAACCCGAAGGAAG
AAATTGGAAGGGTGAATCAGAGCTCACTACTGGCGCCTTATGATGAAGTGTTTTAA

>GaARF24-3
ATGGATGGTGAAGGGCATGGTTCAAAAGCTAAGTTCCCTTTTCACTCCACTAGAGGAGAA
AACAATGATCTCTATAGTAAACTATGGCATGCATGCGCGGGTCCTTCTGTTTATGTTCCTCG
CTCTGGAGATAAAGTTTTGTACTTCCCTCAAGGTCACATGGAACAGGTTGAGGCATACAT
GAGTGAAGATGGCACTATGGAAATGCCCATCTACAATTTACCTTGGAAGATCCTCTGCAG
GGTTTTGCATGTTGAGCTTAAGGTTGAACCTGACACAGATGAGATCTTTGCAGAAATTATT
TTGCTTCCAGAGGCAGAGCAAGATGAGCAAAGCATGGAGCATAGATATTATCGAGCGTCG
CCTCGGGAAAATTATTCTCGTTACTTTAGTAAGAAGCTAACTCCATCGGATATAAAGACACA
CGGTGGATTCTCTATCCCAAAGCGGCATGCCAATGATGGGTGTCTTCCGCTCTTGGACAT
GTCTCAGGAAATCCCCCAGCAGGAACTGCTCGCAACTGACTTGCATGGTCATCCATGGTA
CTTTCGACATGTTTTTCGTGGCTATCCAAAAAGAAATTTGCTTACCACCGGTTGGAGTACC
TTTGTCACCTCGAAGAAGCTTGCTGCTGGGGATTCATTTATCTTTCTAAGAGGGGAAAATG
GAGAGTTCGGTGTTGGAGTTCGCCAATCAATGACAAAGCTACTGAACAGTCCATCTCCAT
CGATCATATCCGCTCACAGTGTGCGACATGGAATACTTGCCAGTGCTTTCCATGCCTTTGC
AACCAGAAGCATCTTTAACGTCTACTACCGTCCTTGGACAAGGTCTTCTGAATTTATCACT
CCACTTGATCAGTATATAAAGGCGGTTCAATTCGACTACTGCTTCGGGACAAGATGTAGAA
TGCGAGTTGAAGGTGGAGAATCTGGGGAACAGAGATCCCTTGGCACTATCATTGGCACT
GAAGATCTCGATCCTATTAGATGGCAGAATTCCAAATGGAGATGTGTGAAGGTGAAATGG
GATCCCGCAGCGAGTTCGGTTTTGCTTCCCGAAAGAGTTTGTCCTTGGAGCATCGATCTC
ACGGAATTCACCAAGAAAAAGAAAACTTCCACTCTGCATCATCAGAAGAGGGCTCGCCCC
AACAACGCATCATCCCCTGAGTTTTCTAGCTTGCTTATGGATGGCATGTTCCATGGTACAG

CTAAAAATCAATCTCAAAGTAGTTCAGGGGTCTTGCAAGGTCAAGAAGACAGTGACACAT
GCGTGAATCAATCCAGTGTACTGCGACAATCATTACCGCATCTTCTTCCGCAAGATCCTGG
TTGTGCCTCAATGCAACAGCAGATGCATAAGCAACTACAAATTCAGATTCCGACCTGTGAC
ACATTTTATCAATGTTCCAGCAGCACAGCACACTTTTCTGGTAGGAAAGTACCCGGTTTGT
GTAATGGGCTCTCAGCATTCTCCTCTAACAGAGTTCATGATGATGCTCGTGCTACCAAAAA
CGGAACTTCTTTGTCCAGACCAAATGGCAGTCACAGATGCATGGTTTTTGGAGTAAATTTA
TTTAATGGCTCACCCGAGCTCCCTTCACCACAAGTTCTCACTTCTAGTGAGGTTCAACGT
CTTTGTTCGACTCCTCTCACTTCTCAGTCGAGTGTTTCCATTGCTTCCAAGGGTATATCTA
GCAAGCAATGCAACAACTGTTGCTCCATCGGCGATCAAACTTGCACCAAAGTGCTCAAGT
ATGGAACTAATCTTGGAAGATCGGTTGATCTCTATCGATTCAATGGATACAAAGGCCTCAT
CCTTGAGCTTGATCATATGTTTGATTTCAATGGAAAGTTGATCGATGGAAGCAGCGGCTGG
CACATAACCTATACCGATGAAGATGGGGATATGATGCTGATTGGAGATCCTTATCCATGGC
AGAAATTTCAGCATGAAGTCCGAAGGATGGTTATCCACCCAAAGGAAGAAATCAACAGGC
TGAATCCGAGCTCACCGAGTTCAGCATCTTACTGA

>GrARF1-2
ATGGCTTTTCCGGCATCAGATATTCCATCTGCTGAACAACAAGCAGATGATCCTTTATATCG
TGAACTATGGCATGCCTGTGCTGGACCTCTTGTCACACTTCCTCGTGTAGGGGAGCGTG
TTTATTACTTCCCACAAGGTCACATGGAACAACTTGAGGCGTCAATGCATCAAGGGTTAGA
ACACCAAATGCCTTCATTCGATCTGCCATCTAAAATACTTTGCAAAGTGGCTTCTGTTCAG
CGTAAGGCTGAACCTGATACAGATGAAGTTTATGCCCAAATAACTCTGGTACCTGAAGTAG
ATCAAAGTGAGGTTATGAGCCCAGATGATCCACTTCAAGAACCTGAAAGGTGCATAGTCC
ATTCATTTTGCAAGACTCTTACTGCTTCTGACACAAGCACCCATGGTGGATTCTCAGTTTT
GCGCCGGCATGCAGATGATTGTCTGCCCCCGCTGGACATGACACAGCAGCCACCATGGC
AGGAACTGATTGCAACTGATCTGCATGGAAATGAGTGGCATTTTCGGCATATTTTTCGAGG
ACAACCTAGGCGCCACTTGCTCACTACTGGGGGGGCTAATGGGGATCTGCGTGTTGGAG
TGAGGAGGCTGATGAGACAACAGGCAAATATGCCTTCTTCTGTTATATCTAGTCATAGCAT
GCATCTTGGGGTGCTTGCCACTGCATCTCATGCCCTCTCTACGCGAAGTATGTTTTCCAT
CTTCTACAAGCCCAGAACAAGTTTGTCTGAGTTCATAGTGAGTGTAAACAAATATCTTGAA
GCTCGAAGCCATAAGCTATCAGTTGGGATGAGGTTCAAAATGAGATTTGAGGGTGAAGAG
GTACCTGAAAGAAGATTCAGTGGCACAATTGTTGGTGTTGAGGATAATAAATCATCTGCAT
GGGCTGATTCTGAGTGGAGATCTCTCAAGGTTCAATGGGATGAACCTTCATCCATCATAC
GTCCTGATAGGGTGTCCCCATGGGAATTAGAGCCTCTTGTTGCAACTAGTAACTCTTCCAT
CTCACAACCTGCACAAAGGAACAAGCGGGCTAGGCCACCCGTTCTACCTTCACCATCTT
CAGATCTTTCTTCACTTGGTATATGGAAATCACCAGTTGAATCTCCTTTCTCGTATTGTGAT
GCACAACGTGGGCAACCATCACCTAAATTATCCTCCACTGCAAAGCCTAATTCTGTTGGCT
TTAGTGGGAATAGCTCCCTGGCTGCAGTTTCTAGCAGCTCTAAGTATTGGCCTAACCGAG
TTGAGAATGTTACAGAATCTGTTGCACCAGTTGTGAACAAAGAATCTAGTGAAAGAAAGCA
GGGAACTGGGAATGGCTGCAGACTTTTTGGTATTCAGTTACTTGACAATATAAACATGGAA
GAAAATTCACCTTTGGCTACAATTTCTGGGACTGGTGTTAATGACCAGCCACTTCATTCAC
TAGATGCTAACTCTGACCAGCAGTCTGATCCATCAAATCTTAATCAGTCTGATCTTCCTTCT
ATAAGTTGTGAACCTGAGAAGTGCCTGAGATCTCCTCAGGAGTCACAGAGCAAGCAAATT
CGGAGCTGCACAAAGGTTCACATGCAAGGTATGGCAGTTGGAAGGGCTGTTGATTTGAC
ACGATTTGACTGTTATGAGGATTTGCTAAAGAAGCTGGAATACATGTTTGACATTAAAGGT
CAGCTTTGTGGATCAACAAAGAATTGGCAGGTTGTCTACACCGATGATGAAGATGACATG
ATGATGGTTGGGGATGATCCCTGGAATGAGTTTTGCAGCATGGTGAGGAAAATTTTTATCT
ATACATCAGAGGAAGTCCGGAAGTTATCACCTAAGATAAAACTTCCAGTGAATGATGATGA
TGATGATAGCAAAGCAACCAAGGCTGGGGTTGACACAGTTATCAACCCTGAAGATCGTTC
ATCAATCGTTGGTCAAGGGTGCTAA
>GrARF2-1
ATGACGAATACGGAGGTAGCTATGAAAGGGAATTGTGTGAACGGAAGAGGAGGAGGAGA
GAGCTTTTCTTCTGGTTATAGTGAACCAAAAGATGGTAGGAACACCGTGGAAGGGCAAAA
TGGTCATTCGACTAATCAAGCTCCGGCTATAGACCCCGAAACGGCGTTGTATAATGAACTA
TGGCATGCATGTGCTGGACCTTTGGTCACTGTTCCTCGTGAACAAGATCGTGTGTTTTAC
TTTCCTCAAGGTCACATAGAACAGGTTGAGGCGTCTACTAGTCAAGTAGCAGACGAGCAG
ATGCCAGTGTATAATCTTCCATCAAAGATCCTTTGTCGTGTGATTAACGTTCAGTTAAAGGC
TGAACCAGATACAGATGAAGTCTTTGCTCAAGTGACTTTACTTCCTGAGCCTAATCAAGAT
GAGAACACTGTGAACAAGGAGCCTCCTGCGCCTCAACCACCACGGTTCCATGTGCATTC
CTTTTGCAAGACCCTCACTGCCTCAGATACAAGCACCCATGGTGGGTTTTCGGTGCTCAG
GCGGCACGCAGATGAATGTCTTCCACCATTGGACATGTCACGGCAACCTCCAACCCAGG

AGTTGGTTGCGAAGGATTTGCATGGAAATGAGTGGCGATTCCGGCATATTTTCAGGGGTC
AACCACGAAGGCACTTGCTTCAAAGTGGATGGAGTGTTTTTGTTAGCTCCAAGAGGCTTG
TTGCAGGGGATGCTTTTATATTTTTAAGAGGCGAGAATGGAGAATTACGTGTTGGTGTACG
ACGTGCAATGAGGCAGCAGGGCAATGTTCCTTCATCAGTAATATCAAGTCACAGCATGCA
TCTTGGGGTGCTTGCAACAGCATGGCATGCCTACACGACAAAAACAATATTCACTGTGTA
CTACAAACCTAGGACAAGTCCAGCTGAGTTTATTGTTCCTTTTGATCAGTACATGGAGTCA
ATGAAGAATAATTACTCCATAGGGATGAGGTTCAAAATGAGATTTGAAGGTGAAGAGGCTC
CCGAACAGAGGTTTACTGGAACAATAGTTGGAATTGAAGATGCTGATCCCAAAAAGTGGA
AGGATTCCAAATGGAGATGCCTCAAGGTGCGATGGGATGAGACTTCTACCATACCTCGTC
CAGAGAGAGTTTCTCCTTGGAAAATTGAACCTGCTTTGGCTCCTCCTGCGCTGAATCCCC
TTCCAATGCCCAGGCCAAAAAGGCCCCGATCTAATGCAGTCCCTTCATCTCCTGATTCCT
CTGTACTTACTAGGGAAGGTTCATCAAAAGCTATTGTAGACCCTTCACCAGCTACTGGGTT
TTCAAGGGTCTTGCAAGGTCAAGAATTCTCGACCTTGCGAGGCAACTTTGGTGAGAGTC
ACGAGTCTGACACTGCTGAAAAGTCGGTGATATGGCGACCTACGGTAGATGACGAGAAG
ATTGATGTGGTTCCCACTTCAAGAAGATTTGGGTCAGAGAATTGGATGTCCTCTGGGAGG
CACGAACCAGCAGCATATGCAGATCTGCTCTCAGGTTTTAGGTCAAATGCTGATTCCTCC
CTTGGGTATTGTCCACCGTTGGTTGATCAAACTTCATTAGCTGGTAATCCAATGAGAAGAC
AATTACTAGATCAAGAAGGGAAGCTTGGCTCTTGGTCCCTCATGTCCTCTGGTCTCTCAC
TCAAGTTGGTTGACAGTAATGCTAAGCCTTCTGTGCAAGGTTCTGAGGTTCCTTATCAAGC
TCGAGGAAATGGTAGATTTAGTGGTTTTGGTGAGTATCCTGTGCTTCAAGGTCATAGGATC
GAGCACCCACATGGAAACTGGTTGATGCCTCCCCCAACATCATCTAATTACGAGAATCCTA
TCCAATCAAGAGATTTAATGCCAAAAGCTTCATTGGGACAAGATCATGAGAACGGAAAATC
TAGAGAAGGAAGTTGCAAGCTCTTTGGTATTCCTCTCATTAGTAATTCTGTTGCATCAGAG
CCCACCGTCTCTCCTATTAATGCCACGAACAAGGCAGCAAGTCATGTGGAAGCTGCACCA
AACCAAGCTCGTACATTTACGTTTGATCAAAAGTCCGAGCAGCCAAAATTCTCACCGTTG
GCAGAGAATCTGTCTATTTTTAATGAGCAGGAGAAATCGTTTCAGCCGGGTCAGCCTCATA
CAAGAGAGGTTCAAAGCAAATCTCCTAGTGCTTCAACTAGGAGTTGTACAAAGGTCCTAAT
GCAAGGGAGTGCTCTTGGTAGGTCCGTGGACCTTACTAAGTTCAACAACTACGATGAACT
GATTGCTGAATTGGATCAATTATTTGAATTTGGTGGTGAATTAATGGCCCCTAAAAAGAATT
GGCTTGTCGTTTATACTGATGATGAGGGTGATATGATGCTTGTTGGAGATGATCCCTGGCA
GGAATTTTGCACCATGGTTCGCAAGATCGGTATCTACACTAGGGAAGAGGTTCAGAAGAT
GAAGCCGGGGTCATTGAATTCAAAGGGCGAGGACAATCTGGTTTCTGCGGAAGGGCTAG
ATGCAAAAGACGTGAAATGTACATCAGCATCTAGTACAGAGAATTGTTAA
>GrARF2-2
ATGACTACGTCGGAGATATCGATAAAAGGAAATTGTGTCAACGGAAGAGGAGATGGTTTTT
CTTCCGGTTATACTGAGCCACGAGATACTAGGAACGCCATGGAAGGGCAGAACGGTCATT
CCGCTCGTACAGCTGCCGTCAGAGAAACCGTAGACCACGAAAGGGCGCTGTATACGGAG
CTATGGCATGCATGTGCTGGACCTCTGGTGACGGTCCCTCGCGAATTAGAGCGCGTGTT
CTACTTTCCTCAAGGTCACATAGAACAGGTTGAGGCGTCTACTCATCAGGTATCAGAACA
GCAGATGCCGGTGTATGACCTTCCACCAAAGATCCTTTGTCGTGTGATTAACGTACAACTA
AAGGCTGAACCGGATACTGATGAGGTTTTTGCTCAAGTGACTTTGCTTCCTGAACATAATC
AAGATGAGAACATGGTGGACAAGGAGCCTCCCATTCATGAACCCCCTCGGTTCCAAGTG
CATTCGTTTTGCAAAACCCTGACTGCTTCAGATACGAGTACCCATGGTGGATTTTCAGTGC
TCAGGCGGCATGCCGATGAATGTCTTCCACCACTGGATATGTCGCTGCAACCTCCAACAC
AGGAGCTGGTTTCTAAGGATTTGCATGGAAATGAGTGGCGATTCCGGCATATCTTCAGGG
GTCAGCCACGAAGACACTTGCTTCAAAGCGGTTGGAGTGTTTTTGTTAGCTCCAAGAAG
CTTGTTGCTGGGGATGCATTTATATTTTTAAGAGGCGAGAATGGAGAATTACGCGTTGGTG
TACGGCGAGCATTGAGACAACAGGGCAATGTTCCTTCATCGGTTATATCAAGTCATAGCAT
GCATCTTGGTGTGCTAGCGACAGCATGGCATGCCTACACTACCAGAACCATATTCACTGT
GTATTACAAACCCAGGACAAGTCCAGCTGAGTTCATTGTTCCATTTAATCAGTACATGGAG
TCGGTAAAGAACAATTACTCAATAGGGATGAGGTTCAAAATGCGATTTGAAGGTGAAGAAG
CTCCTGAACAGAGGTTTACTGGAACAATAGTTGGCATCGAAGATGCTGATCCAAAAAGGT
GGCAGGGTTCCAAATGGAGATGCCTGAAGGTGCGATGGGATGAAACGTCTACAATACCT
CGTCCGGAGAGAGTTTCTCCTTGGAAAATTGAACATGCTTTGTCTCCTCCTGCCCTTAAT
CCCCTTCCAATGCCCCGGCCAAAAAGGCCTCGAACTAATGCTGTATCTTCATCCCCTGAT
TCCTCTGTACTTAGTAGGGAAGGTTCTTCCAAAGTTACTGTAGACCCTTTGCCGGCCAGT
TCATTTTCAAGGGTCTTGCAAGGTCAAGAATTCTCGACCTTGAGAGGCACATTTGCTGAG
AGTAATGATTCTGACACTGCTGATAGGTCAGTGATGTGGCCACCTTCAATAGATGATGAGA
AGATTGATGTAGCTCATGGTGAAAGAAAATTTGGGCCAGAGAATTGGATGCCCTCTAGGA
GGCATGAACCAACTTACACAGATTTGCTCTCAGGTTTTGGGTCGAATGCTGATACATCGC

GCGGATATTATCCTTCCTTTGTTGATCAAACTTCAGTAGCTGGTAATTCGGGGAAAAAACA
ATTACTAGGTCAAGAAGGGAAGCTTGGCTCTTGGTCCCTCTTGCCATCTGGTCTCTCACT
CAAGTTGTCTGACAGTAGTACAGACCCTCCTTTGCAAGGTTCTGATGTGCCTTATCAGGC
GCGGGGAAATGGTAGATTTAGTGGTTTTGGTGACTACCCTATACTTGAAGGTCATAGGATT
GAATGCTCACGTGGTAATTGGTTGATGCCTCCCCCAACCACTTCTTGTTATGATAATTCAAT
CCATTCAAGAGATTTAATGCCGAAAACATCATTGGCTCAAGAGCATAAGAATGGAAAATCT
AGAGAAGGGAACTGCAAGCTCTTTGGTATTCCTCTCATAAGTACCTCTAGCGCATCAGAG
CCTGCAGTCTCTCATATTAGTGCTTTCGCCAAGCCTGTAGGACATATGCAAGCTGCATTGC
ACCAGTTTCATGCACTTGAATCTGATAAAAGGTCTGAAAATTCAAACGCCTCCCAGATGGC
AGAGGATGTTTCTGCTTTTAATGAGCAGGAGAAAATAGTGAAGCTGGGTCAGCCCCATGC
ACGGGAGTTTCAAAGCAAACTGTCTACTGCTTCAACTAGGAGTTGTACTAAGGTTCTCATG
CAGGGGACTGCTCTTGGAAGGTCTGTGGACCTTACTAAGTTCAACAACTATGATGAGTTG
ATCGCTGAATTGGATCAATTATTTGAGTTTGGAGGTGAATTAATGGCCCCTCAAAAGAACT
GGCTTGTTGTTTATACTGATGATGAGGGTGATATGATGCTTGTTGGAGATGATCCTTGGCA
GGAATTTTGTGCCATGGTCCGCAAGATTGGTATCTACACTAGGGAAGAGGTCCAGAAGAT
GAAGCCAGGGTCGTTGGGTTCAAAGTTTGAGGACATTGCAATTCCCACAGAAGGTACAG
TTGCAAAAGAAGTGAACTGTCCATCAGCATCTAGTGCAAAGAATTGTTCAGGGTAA
>GrARF2-3
ATGAAAGGAAATTGTGTTAACGTAAGAGGAGGAGAAAGCTGTTCTTCTGGTTACAGTGAG
TCAATGGATGCTAGGAAGACCATGGAAGGGCAAAATGGTCATTCCACTCATCAAGCTGCC
ATCAGAGACCCTGAAACAGCGCTGTATACGGAGCTATGGCATGCATGTGCGGGACCGCT
GGTGACCGTCCCTCGCGAAGGAGAGCGCGTGTTCTACTTTCCTCAAGGTCACATAGAAC
AGGTTGAGGCGTCTACTAATCAGGTTCCAGACCAGCATATGCCAGTGTATAATCTTCCATC
TAAGATCCTTTGTCGGGTGATTAACGTTCAACTAAAGGCTGAACCAGATACCGATGAGGTT
TTTGCACAAGTGACTCTACTTCCTGAACCTACTCAAGATGAGAATACCGTGGACAAGGAG
TCTCCCGTTCCTCAACCACCAAGGTTCCATGTTCATTCCTTTTGCAAGATCTTGACTGCCT
CAGATACAAGCACCCATGGTGGGTTTTCCGTTCTCAGGCGGCATGCGGATGAATGTCTTC
CACCATTGGACATGTCACGGCAACCTCCAACCCAGGAGTTGGTTGCTAAGGATTTGCATG
GAAATGAGTGGCGATTCCGACATATCTTCAGAGGTCAACCACGAAGGCACTTGCTTCAAA
GCGGCTGGAGTGTATTTGTTAGCTCCAAGAGGCTTGTTGCAGGGGATGCGTTTATATTTTT
AAGAGGTGAGAATGGAGAATTGCGTGTTGGTGTACGACGCGCAATGAGGCAGCAGGGC
AATGTTCCTTCATCAGTAATATCAAGTCACAGCATGCATCTTGGTGTGCTTGCAACAGCAT
GGCATGCCTTCACAACAAAAACCATTTTCACAGTGTATTACAAACCTAGGACAAGTCCGG
CTGAGTTTATTGTTCCATTTGGTCAGTACATGGAGTCATTAAAGAATAATTACTCAATAGGTA
TGAGGTTTAAAATGAGGTTTGAAGGGGAAGAAGCTCCTGAACAGAGGTTTACTGGAACA
GTGGTCGGGATTGAGGATGCTGATCCCAAAAGGTGGCAGGATTCCAAGTGGAGATGTCT
CAAAGTGCGATGGGATGAGACGTCTTCCATACCTCGTCCGGAGAGAGTTTCTCCTTGGA
AAATTGAACCTCCTTTAGCTCCTCCTGCCTTAAATCCCCTTCCAATGCCCAGGACCAAAAG
GCCTCGATCCCATGCAGTACCTTCATCCCCTGATTCCTCTGTGCTGACTAGGGAAGGTTC
ATCCAAAGTTACTATAGACCCATCGTCAGCTAGTGGGTTGTCAAGGGTTTTGCAAGGCCA
AGAATTCTCGACCTTGAGAGGCAACTTTGCTGAGAGTATTGAGTCGGACACTGCTGAAAA
GTCAGTGATGTGGCCTCCTTCTGTAGATGATAAGAACAACGATGTTGTTTCTGCTTCAAGA
AGATTTCCATCAGAGAATTGGATGTCCTCTGGGAGGCATGAACGAACATGCACAGATCTG
CTCACGGGGTTTGGGTCAAATGCTGAGTCCTTGCATGGCTACTGTTCATCCTTGGTTGAT
CAAACTTTAGTGGCTGGTAATTCAACGAGAAAACAATCACTACATCAAGAAGGAAAGCTTG
GCTCTTGGTCGCTCATGCCCTCTGGTCTGTCACTCAAGTTGGCAGACACCAATGCAAAGT
CTCCACTGCAAGGTTCTGATGTGCCTTATCAAATGCGAGGAAATGGTAGATGTAGTGGTTT
TGATGACTATCCTATACTTCAAGGTCATAGGATTGAACACTCGCATGGAAACTGGTTGATG
CCTCCCCCCACTTCATCTCATCATGAAAGCAATCCAGTCCAATCAAGAGATTTAATGCCCA
AAACATCATTTGTACAAGGGCATGAGAATGGAAAATATAGAGAAGGAAATTGTAAACTCTTT
GGTATTCCTCTCATTAGAAATTCTGTTGCATCAGAGCCGACCGTCTCTCATACTAATGCCTT
GAGTAAGCCTGTGGCTCATATGCAAGCTGCATCCCACCAAGTTCTTGAATTTGTGTCTGC
TCAAAATTCTGAAAAGTTGAAGGTCTTGCAATTGGCAGAGGTGGAGGACCTGTCTAATTAT
AACGAGCAGGAGAAACAGCCTCGTACACGAGAGATTCAAAGCAAACCTTCAAGTGCCTC
AACTAGGAGTTGTACCAAGGTTCACAAGCAGGGGATTGCTCTTTGTAGGTCGGTGGACC
TTACTAAGTTCAATGACTATGATGAACTGATTGCTGAGTTGGATGGATTATTTGAATTTAAA
GGCGAGTTAATGGCCCCTAAGAAGAATTGGCTCGTTGTTTATACTGATGATGAGGGTGATA
TGATGCTTGTTGGAGATGATCCCTGGCAGGAATTTTGTGCCATGGTTCGCAAGATTGGTAT
CTACACTAGGGAGGAGGTCCAGAAAATGAAGCCAGGATCATTGAGTTCAAAGGGTGAGG
ACAATTCAGTTTCCACAGGAGGCGTAGATGGAAAGGAAATGATGTGTCCATCAGCATCTA

GTGCAGAGAATTGTTAG
>GrARF3-1
ATGGGGGGATTAATCGATCTGAACTCTACGGAAGACGATGAAACTCCATTATCTGGTTCTT
TGTCTCCATCTTCATCTTCAGCTTCAGTGTTAAGTGCCCCTGGTTCTGGTTCTTCTGTTTG
TTTAGAACTTTGGCATGCGTGTGCTGGTCCCCTTATATCTTTGCCAAAAAGAGGAAATGTA
GTGGTGTACTTCCCTCAAGGCCACTTGGAACAAGTTTCCGATTTTTCCGGTGTAGCTGCA
GCTTATGATCTCCCTTCCCACGTGTTTTGTCGGGTTGTTGATGTCAAGCTCCATGCTGAG
GGTGCCACAGATGAGGTTTACGCCCAAGTTTCATTGGTTCCTGAAACTGAGCAATCTGAG
CAAAAGTTGGAGGGAGGGAAGACTGAGGCAGATGGGGAAGAGGAGGATGCTGAAACCA
ATATCAAGTCAACCACGCCCCATATGTTCTGCAAGACACTAACCGCTTCCGATACCAGCAC
GCATGGTGGCTTCTCTGTTCCTCGTCGAGCTGCCGAGGACTGCTTTCCTCCCTTGGACT
ATAATCAGCAAAGGCCCTCACAAGAGCTTGTTGCAAAAGACCTGCATGGTTCTGAATGGA
GATTTCGACACATCTATAGGGGTCAACCACGGAGACATTTGCTGACTTCCGGATGGAGTG
CTTTTGTAAATAAGAAGAAGCTTGTCTCTGGAGATGCGGTGCTTTTTCTGAGGGGTGAAG
ATGGAGAACTGAGGCTTGGAGTCCGAAGAGCTGCTCAAATTAAAGATGGCTCTTCTTTTC
CATCTTCTTGCAGCCAGCAGTTGAATTGCAGCAATTTTGCAGATGTGGTTCATGCTGTTTC
TATGAAAAGTGTATTCAGCATTTACTACAATCCAAGGGCCAGTTCATCGGACTTCGTAATAC
CCGTGCATAAATTCTGGAAGTGTCTTGATCCCTCATTTTCTATTGGAATGAGGTTCAAAATG
CAATTTGAAGCTGAAGATGCAGCAGAAAGAAGGTACAACCTTATTGAATCTCCAACTCTTG
AGTTCAAATTATTTGATTTTCAGCCTAAAGAGGAGAGCAAAGACCTACTCTACCATGTGAT
GAAGGCCTTAATTGACTTTTCTTGCAGACACTCAGGAGTAATAACTGGAATTAGTGATATAA
ATCCTGTTCGATGGCCTGGTTCAAAATGGAGATGTTTGATGGTAAGGTGGAATGATATTGA
TGCCAACAGGCATGGTAGGGTTTCTCCCTGGGAAATTGAGCCATCTGGTTCAGTTTCCG
GTTCTAACAGCTTGATCTCTCCTGGTTCAAAAAGGAACCGAGTTGGATTTCCTTCAGGAA
ATTCTGAATTTATGGTTCCTGATGGAATTAGAGCATCAGACTTCGGGGAGTTTTTGTGGTC
CCAGGTATTGCAAGTTCAAGAAAATCTGGGTTTTAACACTCTTTATGATGGTTCTGATAGTC
TGAATATGCATTGGTCTGAAATAAGGCGTTGCATTCCTGGTTCTATTGGTTCTGATTTTTCT
GCAATAGGAAATATTGGTAGAGGCTCACTGGTGAGTCCTGATATTTCCCGTAAAAGTGTAG
GCTTTGGGGAATCTTTCCGATTCCGTAAGGTCTTGCAAGGTCAAGAAATTTTTGTGTCCC
CTCCATATAGAAACGGTTCAACTGCAGATGAAAATGAAGAAAATGACGCTTTTGGTCTCCC
TGATGTTGGTCAGTTGTCGGGAACTAGAAGTGGATGGTCTTCCTTGATGCAGAGGTATAAT
ACTCATAGTCGTACACGACCATTTGCACCATCTACACAAACGTCCTCACCATCTTCAGTGT
TAACGTTCCTACAAGTGAGCAATCCAATTCTGAATTTCAGTCCTATCTATAATTCTAACAAC
CAAAAAAGGAAACAGGGAGTTAACAAACAGAGTTCTTTTCATGCACCTGAAATGTATGAG
GGAAAGCTATTTCCATCTTCAGCCAGTGAACATGATTCCCGTGCGAGGGATCTTGGAAGC
TCAGATTTATTCGGTCATTCTATTGGTTCTGTTCAACTTGGTGATGCTTCACCTCTAGCAGC
TCAACCAGTATTCAGGACTAGTCAAGAATTAGATTCCTCCTGTAAAAGTAGCTGCAGACTT
TTCGGTTTCTCCTTGACTGAGGGAAGACATGATGCTAGCAAGGAAGAAGACGTGGTACAA
GCAACCTCATCATTGGCCGCTGGAGCGTTTTTACCTTGTGTTAGGGAAGAGTTTCACCCC
AAGCCTTCGTCGGTGACGGACACAGTTGGAAGCAATTATACTGAAGCAAGCAATCTCTAT
TCTGTCAGAGATATGGTTTTAGATATTGCATTGTAG
>GrARF3-3
ATGGGGGGTTTAATCGATCTGAACACAACAGAAGACGAGGAAACGCCATCATCTGGTTCT
TTATCCCCTTCTTCATCTTCAGCTTCCGTGTTAAGTGCTTCTGGCTCTGCTTCAAGTTCTC
CCGTTTGTTTAGAACTCTGGCATGCGTGTGCCGGTCCACTTATATCTTTGCCCAAGAGAG
GAAGTGTAGTGGTTTACTTCCCTCAGGGCCACTTGCAACAAGTGTCCGATTTTTCCGGCG
TAGCTCCAGCACATGATCTCCCTCCTCACGTGTTTTGTCGGGTTGTTGATGTCAAGCTCC
ATGCTGAGGGTGCCACAGATGAGGTTTACGCTCAAGTTTCACTTGTTCCTGAAAATGAGA
AGTTGAAAGAAGGGAACATAGAAGTAGATGGTGAAGAGGATGCCGAAGCAGATATCAAGT
CCACCACGCCCCACATGTTCTGCAAGACTCTTACGGCTTCTGATACCAGTACACATGGAG
GCTTCTCTGTTCCTCGTCGAGCTGCTGAGGACTGCTTCCCTCCCTTGGATTATAATCAGC
AGCGGCCCTCCCAAGAGCTTGTTGCTAAAGACCTGCATGGCCTGGAATGGAGATTTCGA
CACATCTATAGGGGACAACCACGGAGGCATTTGCTTACAACTGGATGGAGTGCTTTTGTA
AATAAGAAGAAGCTTGTCTCTGGAGACGCTGTGCTTTTTCTTAGGGGTGAGAATGGAGAA
CTGAGGCTGGGAATCCGAAGAGCTGCTCACATTAAAAATGGCACTTCTTTTCATTCTTTGT
GCACCCAGCAGTTGAACCGCAGCAATTTCGCAGATGTGGTCCATGCTATATCTATGAAAA
GTGTGTTCAGCATTTACTACAATCCAAGGGCCAGTTCATCAGAGTTCATAATACCAGTGCA
TAAGTTCTGGAAGAGTCTTGATCATTCTTTTTCCGTTGGAATGAGATTTAAGATGCGGTTT
GAATCTGAAGATGCAGCAGAAGGAAGATACACAGGAGTTGTAACCGGAATTAGTGAGATG
GATCCTGTTAGATGGTCTGGTTCAAAATGGAGATGCCTGCTGGTAAGGTGGGATGATATT

GATGCCAATAGGCATAATAGGGTCTCTCCCTGGGAAATTGAACCATCCAGTTCAATATCTA
GTTCTAACAGCTTGCTCTCTCCTGGTTCTAAAAGGAACAGGGTTGGACTGCCTTCAGGGA
AACCTGAATTTATGGTTCCTGAAGGAATTGGAGCATCAGACTTTGGGGAATCTTTGCGGTT
CCAGAAGGTCTTGCAAGGTCAAGAAATTTTGGGTTTTAACACTCATCATGATGGTGCTAAT
AGTCAGATAATGCACCGGTCTGAAATAAGTCGGCGCTTTCCTTGTTCTAATGGTTCTGGTA
TTGCTGCTATACGAAATATTGGTAGAGACACATTGGTGAATCCTGATATTTCTTATAAAGGT
GTAGGCTTTGAGGAATCTTTCAGATTCCAAAAGGTCTTGCAAGGTCAAGAAACTTCTGTAA
GCCCTCCATGTAGAAGAGGTCCAACTGCAGATGACACTCGAGAAAGTGACAGTCCTGGT
GCTCCTGATGTTGGTCAGTTGTCAGGAACTAGAAGTGGATGGTCTTCTTTGATGCAGAGC
TATAATACTCATAGTCATATAGGACCATCTGCACAAGTGTCCTCACCGTCTTCAGTGCTAAA
GTTCCAACATGCAAGCAATCCATTTGCAAACGTCAATCCTATACATAACTTGAATAGCCAG
GAAAAGGAGCGAAGAGTTCATAAAAGTAGTTCTTTTCATGCTCCCGAAACATATGGGGATA
GGATACCATCTTCAACTGGTGGACATGGTTCCCGTAGGAGGCATCTCGGAAGCCTGGATT
CATTTGGTCCTTCAGCTGATACTGTTCAGCTTGGTGATTCTCAACCTCTGTCAGCACAACC
AACATTTAGGACAAGTCAAGAACTAGCTTCTTCATGTAAAAGTAGCTGCAGGCTTTTTGGT
TTCTCCTTGACTGAGGGAGGACACGATGCTGCCAAGGAGGACAACATGGTGCAAGCAAC
CTCGTCATTGGGTGCTGGAGCTTTCTTACCTCGCATTGGGGAACAGTTTAACACACAACC
TCCTGCAGTGACAAACACAGTTGGAAGCAGTTATACCAAAGTAAGCAACCTCTACGCTGT
CAGAGATATTGTTTATGATATTGCATTATAG
>GrARF3-4
ATGGGGTGTTTAATCGATCTGAACGCTACTGAAGAAGATGAAACGCCGTCGTCTTGTTCT
TTATCCCCATCTTCTGTATTGAGTGCTTCTGGTTTTAGTTCTTCAGTTTGTTTAGAGCTTTT
GCATGCTTGTGTTGGTCCACTTATATCTTTACCAAAGAAAGGAAGTGTAGTGGTCTACTTC
CCTCAAGGCCACTTGGAACAAGCTCCCGAATTTTCCGGCTTAGCTTCAGTTTATGATCTTC
CTCCTCATGTGTTTTGTCGGGTTCTTGATGTTAAGCTCCATGCTGAGGGTGCTACAGATG
AGGTTTATGCTCAAGTTTCACTGGTTCCTGATAATGAGCAAACTGAGCAGAAGTTGCAAG
AGGTAGACGGTGAGGATGAGGATGCCGAAGCAGATATGAAGTTAGCCACGCCGCACATG
TTCTGTAAGACCCTTACGGCTTCCGATACCAGTACTCATGGTGGTTTCTCCGTTCCACGTC
GAGCTGCGGAGGATTGCTTCCCTCCCTTGGATTATGATCAGCAGAGGCCATCACAAGAG
CTCGTTGCAAAAGATCTACATGGTGTAGAATGGAGATTTCGACACATTTATAGAGGGCAAC
CACGGAGGCATTTGCTCACTACTGGGTGGAGTGCTTTCGTAAACAAGAAGAAACTCGTAT
CCGGAGATGCTGTGCTTTTTCTTAGAGGCGAGGATGGAGAACTGAGGCTGGGAATCCGA
AGAGCTGCCCAAATTAAAAACGGGGCTTCTTTTCTTTCTTTGTGCAGCAAGCAGTTGAAC
CGCAGCACTTTTGCAGATGCGGTCCATGCTATATCTATGAAAAGCGTTTTCAGCATTTACTA
CAATCCAAGGGCTAGTTCGTCGGAGTTCATAGTTCCGGTCTGTAAATTCAGGAAGAGTCT
TGATCGTTCATTTTCTGTTGGAATGAGGTTCAAAATGCGGTTTGAAACTGAAAATGCACCA
GAACGAAGATCCTCGGGACTCATAACTGGAACTAGTGATTTGGATCCTGTTAGATGGCCT
GGTTCAAAATGGAAATGCTTGTCGGTAAGGTGGGATGATATTGACGCCAACAAGCACGGG
AGGGTTTCTCACTGGGAAATTGAACTTTCCGGTTCTATTTCTAGTTCTAACTGCTTGCTCT
CACCCTGTTCGAAAAGGAACAGGGTTGGATTGCCTTCAGGAAAACCGGAACTTATGGTTC
CTGATGGAATTGGAGCACCCGACTTTGGGGAACCTTTGCGGTTCCAAAAGGTCTTGCAA
GGTCAAGAAATATTGGGTTTTAGCACTCTTTATAATAGTGCTGATAGTCACAACATGCATTG
GTCTGAAATACGACGGTGCTTTCCTGGATCTAATGGTTCTGGCATTGCTACAATTGGAAAT
GTTGGTAGAGACCCTCCGTTGAATCCCGTTATTTCCTATAAAGGTGTAGGCTTTGGGGAAT
CTTTTGGATTCCATAAGGTCTTGCAAGGTCAAGAAATTTTTCTAAGCTCTCTGTATAGAAGA
GGCTCAACTATGGAAGAGACTCGAGGAAATGACAGTGCTGGTCTCCCTAATGTTGGGCA
AATGTCGGGAACTAGAAGTGGATGGTCTTCCTCGATGCAGGGATATAATACACATAGTCCT
GTACAACCATCTGCGCAAGTTTCCTCACCATCTTCTGTGTTGATGTTCCAACAAGCAAGC
AATCCAGTTCCGAACTTCAATCCTACTCGTAACTTCAACCAGGAAATGGAGCGGGGAGTG
AGTTCTTTTCGTGTGCCTGAAACTTATGGGGCCAAGTTACTGTCATCGTCAATCAGCGAA
CATGATTCACAACCTCTGGCAGCTCAACCTTCTTTTGGGACCAATCAAGAATTAGCCTCGT
GTAAAAGTAGCTGCAGGCTTTTTGGTTTCTCCTTAACTGAAGGAGATCTTGATGCAACTAA
GGAAGACAACATGGTACATGCAACATTATCATTGGGTCGTGGTTCGCTTTTACCTTGTATC
GGAGAAAATTTCCATCCAAATCCTCCAGCAGTGGCAAGCACAGTTTGGAAGCAATTGTAC
TAA
>GrARF4-1
ATGGTGGGTCTCCCTTCTGAGGATTCCTCAGATTCCACTCACCGAGTCGACTCAGACGA
CAATGGCAACAACAACAACGACATCAGTAACAACGATGACCAGCGAGTTTACTTTGTGCC
CTTCAGGTGGTGGAAGGATGCACAGGATTCAACTTCAAGCGAATCTGATTCGAAGAAGG
GGATTTTGTATACAGCTACACCAGGAACCTCGTATGCGGGTCCCATGAAGTTAATCAACAA

CATTTTCAACTCAGATCTTGCTTTCAATTTAAGGAAGGAAGAGGACAGTTCTTTGCACAAT
GGTGAAAATGGAGAGGTTGGTGTTTCAGGAAGGGACTACGCATTGGTTCCTGGTGAAAT
GTGGTTGCAGGCTCTTAAGTGGCATAGTGATGCTAAAGTTGCAGCAAAAGGTGGAAAAAG
CTTTTCAGCTGCTGAAGATGACATGGCGGATGTGTATCCCTTACATCTAAGACTTTCTATTC
CAAGGGAAACAAATTCATTGGCAGTAAAAATTAGCAAAAAGGACAATGCAGTGGAACTTTT
CAGAAGAGCCTGCAAAATCTTTAGTATAGATTCTGAGCAGTTACGCATTTGGGACTTTTCC
GGGCAGACAGCTCTATATTTTGTGAATGAGAGAGATAGATTTCTCAAAGATTCTCAACGCC
AATCAGATCAGGAGATTCTCCTGGAGTTGCAAGTCTATGGGTTGTCAGATTCCATGAAATG
CAGGGAGGGGAGAAAAGATGAAATGTTAGGACATCCAGCAAATTGCTCTTCTGGTGCTCC
AGTCTTGTCAAATGGTTGCAATGGCACTGCAAATTCCAATTTTGGCAGCACAAATGCTTCA
ATATTCTGTGGACGATCTGGAGAAGCTGGTTCTTTGGGATTAACTGGATTACAAAATCTCG
GGAACACTTGTTTCATGAACAGTGCCATTCAGTGCTTGGCTCATACACCAAAGGTGGTTG
ATTATTTTCTTGGAGATTATACTAGAGAAATAAATCCCGACAATCCTTTGGGAATGAAAGGT
GAGATTGCTTCAGCCTTCGGGGATCTGTTGAGGAAGTTATGGGCCCCTGGAGCAACCCC
TGTGGCACCGAGAACATTTAAGTCAAAGCTTGCTCGTTTTGCTCCTCAGTTTAGTGGCTT
CAATCAGCATGATTCTCAAGAACTTCTTGCATTTTTGTTGGATGGACTTCATGAGGATCTC
AACCGTGTGAAAAGTAAGCCTTATGTTGAAATGAAGGACAGCGAAGGTCGCCAAGATGAA
GAAGTAGCTGATGAATATTGGCAAAATCACATCGCTCGCAATGATTCTATCATCGTTGATGT
GTGCCAAGGTCAGTATAAATCAACTTTAGTTTGTCCACAATGCAAAAAGGTTTCTGTAACG
TTTGATCCATTCATGTACCTGTCACTACCTCTACCATCAACAACTTCGCGAACAATGACTGT
AACTGTCATCAGTACTGATGGAACCTTTCAACCATCTCCATTTACAATTACTGTCCCAAAGA
GTGGGAAACTTGAAGACCTTATACAGGCTCTCAGTTTTGCTTGTTCTTTGGGGGCTGATG
AGACCCTTCTGGTGGCAGAGATATACAACCACCGAATTATACGTTATCTTGAAGAGCTTTC
TGATTCGCTATCCTTGATTAGAGACGATGACACGCTGGTTGCTTATCGATTAAATAAAGACA
TTGAGAAAGCTCCTTTAGTTGTGTTTATGCATCAGCAGATGGAGGAGCAGTATATGCTTGG
GAAACTGACTTCAAGTTGGAAAACATTTGGAATTCCTCTTGTAGCAAGGCTTTCTAATGTT
GAAACTGGATCTGACATCCATGGTCTATATCTCAAATTACTAAATTCATTTCAGACACAGGC
TGAAGATGTCTTAGAAGACCATGATACCTCTGAGAGTACTGCAGTTGAAGACATTTCTCTG
AAAGAGCATGATGCATCATCTCCTATTTCAAACGGCATTGAAAAGCCTCCCGATGCAAATG
GGGTTTCTGCACTCTCAGAGGCTGAATTACAACTTTACATAACAGATGACAAGGGAATAGT
CAAGGAATCCCAGATACTTATGGCTGAAGCAATACCAGCTGCTGGCATGTCTAGAAGGTT
ACATGTGCTCGTTCCTTGGCCTGAAAAATATGTTAAGCAGTATGATACACAGCTTCTTGGC
TCTTTACCGCAGATTTTTAAGTCCTGCTTTTTTGCTAAGCGACCTCAGGAATCTGTTTCTCT
TTATAAATGTCTTCAAGCATTCTTAATGGAGGAGCCACTTGGACCAGAAGATATGTGGTAC
TGTCCGCGCTGCAAAGAGCACCGTCAAGCCAGTAAAAAGTTGGATCTTTGGAGGCTGCC
TGAGATTGTGGTTATTCATTTAAAACGATTTTCTTATAATCGATTCCTGAAAAACAAGCTGG
AGACATATGTTGACTTCCCCATTGACAATCTTGATTTGTCAAATTATATTGGCTCTAGGAAT
GGCGAGTTATCTAATCGTTACATGCTTTATGCTGTCAGTAACCACTATGGAAGCATGGGAG
GTGGTCATTATACTGCATTTGTCCATCATGGGGGTGGACAATGGTATGAGTTCGATGATAG
CCATGTTTTTCCCATCAGCCTGGAGAAGATAAAGACATCTGCTGCTTATCTTCTTTTCTACA
GAAGAGTTGTAGAATAA
>GrARF4-2
ATGGAAATTGATCTGAACCATGCAGTAAACGAGGTGGAGAAGACTGTACTTTGCAATGGG
AGCTGTGAAAAAGCTAGTGCTTGCGTTTACTGTTTGTCGTCGTCGTCGTCGTCATCATCAT
CTTCTTCTTCTTCTTCTTCATGTTCTTCAAACTCAGGTTCACCTCCTTGTTCATCTTCCATT
GACTTGGAGCTTTGGCATGCTTGTGCTGGTCCTCTCACATCACTTCCAAAGAAGGGAAAT
GTGGTTGTATACTTCCCTCAAGGTCACTTGGAACAACTTGCCCTTGCTTTTCCTTTCTCTC
CCTTGGACACCTTTGATCTTCCTCCCCAAATCTTTTGCAAAGTGATGAATGTCCAGCTTCT
GGCCAATAAGGAGAATGATGAGGTCTATACGCAAGTCACTTTACTTCCTCAACCGGAGTT
GGGAGGGCATAATTTGGGGAGCAAGCAGCTGGATGAGGTAGGGGCGGATGAGCATGCC
GATGGGTCGCCTTCGAAGCCAACCTCTCACATGTTTTGCAAGACACTAACAGCTTCTGAC
ACTAGCATCCATGGAGGGTTCTCTGCTCCTCGTAGAGCTGCTGAAGACTGTTTTCCTCGG
CTGGATTATAAACAGACAAGGCCCTCACAGGAGCTTGTTGCTAAAGACCTCCATGGAGTT
GAGTGGAGGTTTCGCCATATATTTAGGGGTCAACCGAGGCGACATCTCCTTACTACCGGC
TGGAGTGTTTTTGTGAGTCAAAAGAATCTTATTGCTGGGGATGCAGTGCTCTTTCTGAGG
GGCGAAGATGGGGAGCTGAGGTTGGGAATTAGAAGAGCCGTTCGACCAAGAACTGGTC
TTCCAGATTCAGTTATTGCGAAACATAATTCATATCCAAAGGTTCTTTCTGCAGTGTCTAAT
GCATTATCCACCAAAAGAGTGTTTCAAGTCTGCTACTGTCCAAGGGCCAGTCATGCCGAG
TTCGTCATACCCTTCCAAAAGTATATTAAAAGCATCACAAATCCAATGTGCACTGGGGCAA
GATTCAGAATGAAATTTGAAATGGATGATTTGCCGGAGAGAAGGTGTAATGGTGTAGTGAC

AGGAATAGGGGACTCAGATCCTTATAAATGGCCTAACTCAAAGTGGAGATGCGTAATGGTT
AGGTGGGATGATGATATCGCAAGTGATCACCAAGAGAGAGTTTCACCTTGGGAAATTGAT
CCTCCTGTTTTTCCCCCACCCTTAAGTATTCCATCTCCATTCAGGTTAAAGAAACTGCGGA
CAGGTCTGCAGACTGCTGCACTTGACACCCCTATCACTGGAGGGGTTGGATATTTGGACT
TTGAGGAATCGACAAGATCCTCTAAGGTCTTGCAAGGTCAAGAAAATGTAGGTTTCATATC
ACCCGTATATGGATGTGGTACAGTGAACCATCCCCTCAATTCCGAGATGCAATCTATGGCA
CATCAAAGTCTTGCATCGACAGGAATTGGAAAGAGTAACATTAGTGATTTTATGAGGGCTC
ACTCCTCCTCTTACACAGGCTTTGCGGAAACTAATCGGTTTCCGAAGGTCTTGCAAGGTC
AAGAAATTTGCTCATTGAGGTCCCTCACACACAAGGCTGATCTCAACCTTGGTGTTTGGG
CAAAAACCAATCATGGTTGCAATTCTTTCAACATGAATCAAGCACCCAACACCAACTGCTA
TCCAGAAGGACTTCGAAATATGTATTTTCCTTATAATGAGTTTTACAAAGCTGGCCAGGAAC
CAAAAATGTTTTCTTACGCATCTAAACTTCCAAGGGGCAATGTTTTGTTCAATGCTTCCTCA
ATTAAGCCGGGGGTTAGTGTGGACGACATCAGGAAACCAAACCCTCCGAATGACCATAAG
CCAATGGAGAATATCCCTAGTCCCGGTTTTGGAAAAAAATTGAGGAACCAACAGAATGAG
TGCTACAAAGGAAATGTGGCTGGATGTAAACTCTTTGGATTCTCCTTGACTGCGGAATCA
CCTACTCTGAACTCACAAAATTCTGGTAAGCGGAGTTGTACAAAGGTTCACAAGCAAGGC
AGCTTGGTTGGAAGAGCCATTGATCTCTCAAGACTTTATGGATATAAAGACTTGATGACTG
AACTAGAACATCTTTTCGGTATGGAAGGTCTTTTAAATGATCCTGATAAAGGGTGGCGGGT
TTTGTACACTGACAGGGACAATGATGTAATGGTAGTTGGAGACGACCCGTGGCATGAATT
TTGTGATGTGGTATCCAAGATCCATATATACACAGGAGAAGAAGTGGAGAAGATGACCGTA
GGAATGGGTAGTGATGAGACTCAAAGCTGTTTGGAAGAAGCAGCAGTGATAATGGAAGCA
TCAAAGTCGTCCTCGGTGGGGCAGCCAGATTCCTCTCCCACTGTAATGAGGGGTATGAA
GATCAGTGTTTGCTTCTAG
>GrARF5-1
ATGGGTTCCTTTGTTGAAGAGAAGATGAAAACAGGAGTGTTCGGTAATGGATGGGCAGAG
GCTACTTTGCTCGAGGAGATGAAGTTACTGAAAGAAATGCAACATCAGTCTGGGAGCAGG
AAGGCAATAAATTCGGAGTTATGGCATGCCTGCGCAGGTCCACTTGTTTCGTTGCCTCAG
GTGGGGAGTCTAGTTTACTACTTCCCTCAAGGACACAGTGAACAGGTATCAGTGTCCACA
AAAAGAATGATGACCTCTCAAGTTCCCAACTACCCGAATCTTCAATCTCAGTTGATGTGCC
AAGTTCACAATGTTACACTACATGCAGACACAGAAACAGATGAAGTTTATGCTCAAATGAG
TCTTCAACCAGTGAACTCTGAAAAAGATGTGTTTAATATACCAGACTTCGGAGTGAACCCG
AACAAGCATCCAAATGAAATTTTCTGCAAAACTTTGACTGCAAGTGATACAAGTACACACG
GTGGCTTTTCAGTGCCACGTAGGGCAGCAGAGAAGCTCTTTCCTCAGCTGGATTACACAA
TGCAGCCCCCATCTCAAGAGCTTGTTATGCGAGACATACATGATAATACTTGGACGTTTCG
CCACATATACCGCGGGCAACCAAAGCGACATCTTCTTACAACTGGGTGGAGTTCGTTTGT
TGGATCCAAAAGGCTTAGGGCAGGCGATTCCATTCTCTTTATCAGGCATGCTATAGTCACA
CACTGCATTGCTTTGAGCTGTAGACTTGCATTTGTTTGGCATGCCAATCGTCATCAAGCAA
CTTTGCCATCATCAGTTTTATCTGCAGATAGTATGCACATTGGTGTCCTTGCTGCTGCTGC
TCAAGCTGCTGCAAATAGAACCCCATTCACAATCTTCTACAATCCAAGATCCTGTCCTTCT
GAATTTGTCATCCCCATGGCTAAATACTGTAAATCTGTTTACGCAACTCAAGTCTCAGCTG
GTATGAGGTTTGGAATGATGTTTGAAACAGAAGAATCCGGAAAACGTAGATATATGGGTAC
AATAGTTGGCGTTAGTGACTTGGATCCTCTGAGATGGCCTGGCTCGAAGTGGCGAAACCT
TCAGGTTGAGTGGGATGAACCTGGATGTCATGATAAACAGAATAGGGTCAGCCCATGGGA
AGTTGAAACTCCTGAAAGCCTCTTTATTTTTCCTTCTATAAATTCAGGACTCAAGCGACCAT
ATCCTTGGATTTTGGGAGCAGAATCTGAATGGGGAAACTTGATCAATAGGCCTCTTCTCCA
GTTTCCTGAAAATGGAAGTGGGAATCTTCCTTACCCAATCTCAAACTCATATTCCAAGCAA
CTGATGAAGATGATGCTGAAGCCTCATCTTGTTAACCATCCTGGAACTTTTTCATCCACTT
CACAACAAATCTCTGCTGCAAAGGGATCTCCATTAAAGGAGATGAAGAATGTGCAATATAC
AAGCAATCAGGAACCTCAGCTTATGCAATCAGAAAATTTGTTGATAGAAAAACCAAATCCT
TCCCAGTTAGCCCTTGACCAACCTGATCCCATCAACTCAAATTTGCTGAAAATAAATGCTA
ACGGGAATCCACATCCCGCAAACGAGTTTGAAGGCCAAACACAAGCCAGGTGTAATAACA
AAAAATTAAAGTCGGAACCAGAACATTCAACTGATCAGTTAAGTCAGTTGAACTCGACATT
GGAATGCACTGAGGATAAATTGGTGGCAAATACTATAAGTCCAACTATTTGCAACAAACTTT
CTTTCCCTAACAACAACCAGACTCCATCCCAATTGCAAAACAATCCATGTCCAATTCAGTC
ACAGTTTGAACCATCACTCCTCCAAGCTCATCAGATGCAGGTTTCTCAAGCTGATATTAGT
AGTTTAAGCAGCTATCTTCCTTTCTTCAACACCGATGAATGGGCAACAATCCTTCCTTCTT
GCCAATCTATTGCCGGGGTTTATGGATCACCTGGTCCCATACCGCTAGTCGGGTTACAAG
ACTCCTCAGCTGCTTTTCCTGAGGCAACTAATCCTACTTTAACTACTAGGAGTGAAGTCAC
ATGGGATAGTCAGCTGTACAATTGTAGGTCTTCATCCCAAGCCAACGAATTATGTTCATTC
GCTCAGCAAGACCCATGTTCTTTTAATTCTGGTAGTATAAGAGATTTGTCCGATGACAGCA

ATAGTAAGAGTGGGATATACAGCTGTTTTAGTAATAATGGTAGTACTGTGATTGATCCTTCT
GTTTCCAACGTCATTCTGGAAGAGTTGTGTTCATTGAAGGATGCTAAGTTTCAAAAACCTT
CTGATAGCTTGGTTGGAAACTTTAGTTGTAGTCAGGATGTTCAGTCTCAGATTACCTCTGC
TAGCCTTGCAGATTCTCGGGTTTTCTCTCGACAAGACATACCAGACAGCTCGGGTGTCAC
GTCTTCGAGCAATGTTGATTTTGATGAGAATGGCCTTCTGCAGAAGACTTCATGGCAGCA
AATCACCCCGCGACTAAGAACATATACAAAGGTTCAAAAGGCAGGATCCGTTGGAAGGTC
AATTGATGTCACTAGTTATAAGAACTATGATAAATTACTCTCTGCAATTGAATGCATGTTTGG
ACTTAAGGGGCTGCTTAAGGATCCGAGAGGTTCAGGGTGGAAATTGGTATACGTGGATCA
TGAGAATGATGTTCTACTTGTTGGGGATGATCCTTGGGAACAATTTGTTGGGTGTGTCCG
CTGCATCAGAATTCTATCCCCTACAGAAGTACAGCAGATGAGTGAGGAAGGGATGATGCT
TCTCAACCGTGTCGGTTCCACAGTGCAAGGCCCCATTTCTGAAGTTTGCAATGCTCCACC
TAAGAATGAACCGTAG
>GrARF5-3
ATGGGTTCTGTCGTTGAAGAGAAGATCAAACAAGGAGGTTTGGTTAATGTAGGTGCACAG
TCCACTCTGCTTGAGGAAATGAAGCTATTGAAAGAAATGCAAGATCAATCTGGTACCCGTA
AGGCTATAAATTCCGAGTTATGGCATGCCTGTGCTGGTCCACTTGTTTCCTTGCCTCAGGT
GGGAAGTCTTGTGTATTACTTTCCTCAAGGACATAGCGAACAGGTAGCAGTGTCCACTAA
AAGAATGGCGACTTCTCAAATTCCCAACTACCCAAATCTTCCATCTCAGTTAATGTGCCAA
GTTCATAACGTTACATTACATGCAGACAGAGACACCGACGAAATATATGCCCAAATGAGTC
TTCAACCAGTGAACTCTGAAAAAGATGTGTTCCCTATACCAGACTTTGGATTGAAGCTGAG
CAAGCATCCTAATGAATTTTTCTGCAAAACTTTGACTGCAAGTGATACAAGTACACACGGT
GGTTTTTCAGTGCCACGTAGAGCAGCTGAGAAGCTCTTTCCTTCATTGGATTATTCCATGC
AACCTCCAACGCAAGAGCTTGTTGTGAGAGATTTGCATGATAACACCTGGACGTTTCGTC
ATATATACCGTGGGCAGCCGAAGCGACACCTTCTTACTACGGGGTGGAGTTTGTTTGTAG
GATCAAAAAGACTTAGAGCTGGTGATTCCGTTCTCTTTATCAGGGATGAGAAATCACAGTT
ATTGGTGGGTGTAAGGCGTGCTAATCGTCAACAAACCACATTGCCATCATCTGTTCTATCT
GCTGATAGTATGCACATTGGTGTCCTTGCTGCCGCCGCTCATGCTGCTGCCAATAGAAGT
CCATTCACAATTTTCTACAATCCAAGAGCATGCCCTTCAGAATTTGTCATCCCTTTGCCTAG
ATACCGTAAATCTGTATATGGGTCTCAAGTCTCAGTCGGTATGAGGTTTGGAATGATGTTT
GAAACGGAGGAGTCCGGGAAACGTAGATATATGGGTACAATAGTTGGTATTAGCGACTTG
GATCCTCTAAGATGGCCTGGCTCGAAGTGGCGAAACCTTCAGGTTGAATGGGATGAACC
TGGATGTAATGATAAACAGAATAGGGTGAGCGCATGGGAAATCGAAACTCCTGAAAGCCT
CTTTATTTTTCCTTCGTTAACTTCAAGTCTAAAGCGACCATTGTATCCTGGATTTTCAGGAG
CAGAATCTGAATGGGGAAGCTTGATGAAAAGGCCCCTACTCCAGTTTCCTGAAAATGGAA
ACGGGAATCTTCCCTATTCAATGTCGAATTTATGTTCTGAACAATTAATGAAGATGATGTTG
AAGCCTCAGCTTGTTAACCATCCTGGAATTTTTGCTTCCCCCTTACACCAAATCGCTGATG
TAAAGGTACCTCCATTAGAAGAAATGAAGAACTTGCAGTCTAAAAGCCACACAAAACCCCA
GGTTATCCAATCAGAAAATATGTTGATAGAGAACCGAAATCTTTCCCACCCAGTCCCTGAC
CAACCTGATCCCATAACTTCAAATATGTCCAAAATCAATGCTAATGGGAACCCACATCCTG
CAAATATTCTAACACAAGCTGGGACTGGGAGCAGTAATGAAAAACTAAAGTTGGAATCAAA
GCATTCAGCCGAGCAGCTGACTTCGACATCGGAATGCAATGAGGAAAAATTGGTGGCAA
GTACTGTAAACACAACTATGTCGAACCAACTTTCTTTCCCTACCCAGCCCCATATTCCACT
CCAAGTGCAAAATAATCCCTGGTCGATTCAGTCACAGTTGGATTCGTCAGTCCTCCAAGC
TCATCAAATGCTTGTATCCCAAGCTGATATTAGTACTTTAAACAGCTTTCTTCCTTTCTCAG
ACACTGATGAGTGGACGTCAAATCTTTCTTCTTGCCAACCTCTTTCTGGGGCATACAAATC
ACCTGGTCCGATACCAATGGTTGGGTTACAGGACTCTTCAGCTGTCTTTCCAGTTGAAAC
TGATGATTCATTAACTACGGTGGGTGAGGAAATATGGGATCAAAAGCTGAATAATTGCAGA
GTTTCATCCCAAGCAGACCAATTGGCTTCATTCACTCAGCAAGATCCATGCAGTCTTAATT
CTGGTGGGGTGAGGGATTTGTCTGATGACAGCAACAATCAAAGTGGGATATATAGTAGCT
GTCTTAACATTGATGTTAGCAATGGTTGCAGCACCGTGATTGATCCTTTTGTTTCCAGTGC
CATTCTAGATGAGTTTTGCTCATTGAAAGATGCTGATTTTCAAAACCCTTCAGATTGTTTGG
TTGGGAACTTTAGTTCTTGTCAGGATGTTCAGTCTCAGATTACCTCTGCTAGCCTTGCAGA
TTCTCAAGCTTTCTCTCGACAAGACTTGCCTGACAGCTCCGGTGGCAATATCGATTTTGAT
GACAGTGGCCTTCTGCAAAACAATTCCTGGAAGCAAACAGGTCCACGCGTTAGAACATAT
ACAAAGGTTCAGAAGGCAGGATCTGTAGGAAGGTCGATTGATGTCACGAGTTTTAAGAAT
TATGATGAACTAATCTCTGCAATAGAATGCATGTTTGGACTCAAGGGTCTGCTGGATGATC
CCAGAGGTTCAGGCTGGAAATTGGTGTATGTGGATTATGAGAATGATGTTCTTCTCGTTGG
AGATGATCCTTGGGAGGAATTTGTTGGGTGTGTTCGTTGCATCCGAATTCTATCCCCTACA
GAAGTACAACAGATGAGTGAAGAAGGAATGAAGCTTCTCAACAGTGCTGCCACAGTGCA
AGGCATCAATGGCTCTAACTCAGAAGGTTCCAATGCAAATGCTTAA

>GrARF6-2
ATGAGGCTTTCTTCAGCTGGTTTCAGTCCACAAGCTCCGGAAGGAGAGAAGAGAGTTCT
GAACTCTGAACTTTGGCATGCATGTGCGGGTCCTCTTGTTTCTCTACCTCCAGTTGGAAG
TAGGGTTGTTTACTTCCCACAGGGTCATAGCGAACAGGTAGCTGCATCAACCAACAAGGA
GGAAGTGGATGTCCACGTACCTAACTACCCAAGCTTACCTCCACAACTTATATGTCAGCTC
CATAATGTCACCATGCATGCAGATGTTGAGACAGATGAAGTATATGCACAAATGACATTGC
AACCACTGAATCCGCAAGAACAGAAGGAGGCTTACCTTCCAGCAGAATTGGGCACTTCC
AGCAGACAGCCAACAAATTATTTCTGTAAAACATTAACAGCCAGTGACACAAGCACTCATG
GAGGGTTCTCTGTTCCTCGCCGAGCTGCTGAAAAAGTGTTTCCCCCACTGGACTTCTCC
CAGCAGCCTCCTGCTCAAGAGTTGATTGCAAGGGACTTACACGATAATGAATGGAAATTTA
GGCATATATTTCGGGGTCAGCCCAAAAGGCACCTCTTGACAACAGGATGGAGCGTATTTG
TAAGTGCTAAAAGACTAGTTGCAGGTGATTCAGTTCTTTTTATCTGGAATGAAAAAAATCAA
TTACTTCTTGGCATCCGACGAGCTAATCGACCTCAAACTGTAATGCCTTCATCTGTTTTATC
GAGTGATAGCATGCACTTAGGGCTTCTTGCTGCCGCTGCTCATGCAGCTTCAACAAATAG
CCGCTTCACTATATTTTATAACCCAAGGGCTAGTCCATCAGAATTTGTAATACCTTTAACAAA
ATATATCAAAGCTGTCTATCATACTCGAGTTTCTGTTGGTATGCGCTTTAGAATGCTGTTTG
AAACAGAAGAATCAAGTGTTCGCCGATACATGGGTACAATCACTGGCATAAGTGACTTGG
ATCCTGTCCGGTGGCCAAATTCACATTGGCGATCAGTTGGCTGGGATGAATCAACAGCTG
GAGACAGGCAGCCTAGAGTCTCCTTGTGGGAGATTGAACCATTGACAACATTCCCTATGT
ATCCATCACCATTTCCATTAAGACTTAAGCGACCATGGCCAGCAGGATTACCTTCTTTCCA
CGGCATCAAGGATGATGGTCTAGGCATGAATTCTCCACTTATGTGGCTACAAGGAGATGC
AGGTAGAGGAATGCCATCTCTGAATTTTCCAGGGATTGGAGTTACACCGTGGATGCAGCC
AAGGCTGGATGCTGCTTCCATGCTGGGTTTGCAGACTGACATTCACCAAGCTATGGCTGC
TGCTGCGCTGCAAGACATGAGAGCAGTGGATCCCTCCAAATCAGCAACTACTACCCTTCT
GCAATTCCAGCAACCTCAAAATCTATCCTGCAGGCCTGCTGCTTTAATGCAGTCCCAGAT
GTTGCAGCAGTCTCAGCCTCAGGCTTTTCTTCAGGGTGTTGAAGATAACCAACATCAGTC
TCAGACTCAGGCTCAAACCCAACCGCCTCTTGTTCAGCAACAATTGCAGCAGCAGAATTC
ATTTAATAACCATCAACACCAACAGCAGTTACAGCATCCGCTGTCACAGCAACACCAGCAA
CTGGTCGATCATCAGCATATTTCTACTGGAGTGTCTGCCATGTCACAGTATACTCCGGCCT
CACAGTCCCGGTCGTCACCTTTCCAAGCCATACCTTCACTATGCCAACAACAGAGTTTTT
CTGACTCAAATGGGCACACCATGACCAGCCCTATTGTATCTCCCTTGCATGGTCTTTTGG
GATCCTTTCCCCAAGATGAATCGTCCGGTCTGCTCAACTTGCCTAGATCCAACCCAGTAAT
AACATCTGCTGCATGGCCATCTAAGCGGGCTGCTGTTGAAGTTCTGTCATCTGGATCTCC
ACAATGTGTTCTGCCCCAGGTGGAACAGTTGGGGCCCACCCAAACAAACATTTCTCATAA
TTCTATTTCGTTGCCACCCTTTCCTGGCAGGGAGTGCTCGATAGACCAAGCAGGGGGTAC
TGATCCACAGAGCCATCTCTTATTTGGTGTTAATATAGAGCCTTCATCTCTTCTATTGCAAA
ACGGGATGTCAAGCCTTAGGGGAGTTGGCAGCGAGAGTGACTCCACTACCATACCCTTC
TCTTCTAATTATGCGAGTACTGCAGGCACTGATTTTTCAGTTAATCCAGCAATGACACCTTC
CAGTTGCATTGAAGAATTGGGATTCTTGCAGTCTCCAGAGAATGTGGGCCAAGAAAACCC
ACAAACCAGAACCTTTGTTAAGGTTTATAAATCAGGGTCCTTCGGGAGGTCGTTGGATATC
TCCAAATTTAGCAGCTACAATGAGCTGCGCAGTGAACTCGCACGCATGTTTGGCCTTGAA
GGCCAGTTGGAGGACCCTTTGAGATCAGGCTGGCAGCTTGTATTTGTTGATCGGGAGAA
TGATGTTCTTCTCCTTGGTGATGATCCTTGGCCGGAGTTTGTGAACAGTGTGTGGTGCAT
CAAGATACTTTCACCGCAAGAAGTGCAGCAAATGGGCAAACGAGGCCTGGAGCTTCTAA
ACTCTGTTCCAGTTCAGAGGCTCTTGAATGGCAGTTGTGACGACTATGCGAGCCAGCAG
GACTCGAGAAATTTGAGCTCTGGTATTGCCTCTGTGGGGTCATTGGACTACTGA
>GrARF6-4
ATGAGACTTTCTTCTGCTGGTTTCAATCCACCAAACCAGGAAGACACTGCAGGAGAAAAG
AGGGTCCTCAACTCTGAACTGTGGCATGCGTGTGCTGGACCTCTTGTTTCTCTACCACAT
GTTGGAAGTAGGGTTGTTTATTTCCCACAGGGTCATAGTGAGCAGGTAGCTGCAACAACC
AACAAGGAAGTGGATGCCCACATACCTAACTACCCAAGCTTACCTCCACAACTTATTTGTC
AGCTTCATAATGTTACCATGCATGCTGATGTCGAAACAGATGAAGTATATGCACAGATGAC
CTTGCAACCATTGAGTCCGCAAGAACAAAAGGAGGCTTATCTACCTGCGGAATTGGGCAC
TCCCGGCAAACAGCCAACAAACTATTTCTGCAAAACATTAACAGCCAGTGACACAAGCAC
TCATGGAGGGTTTTCTGTTCCTCGCCGAGCAGCTGAAAAAGTGTTTCCTCCACTGGACTT
CACCCAGCAGCCTCCAGCTCAAGAGTTAATCGCAAGAGACTTGCATGATAATGAATGGAA
ATTTAGGCATATATTTCGGGGCCAGCCCAAAAGGCACCTCTTGACGACTGGATGGAGTGT
CTTTGTAAGTGCTAAAAGACTAGTTGCAGGTGATTCAGTGCTTTTTATATGGAATGATAAAA
ATCAATTACTTCTTGGCATCCGGCGAGCTAATCGACCTCAAACTGTAATGCCTTCATCAGT
GCTATCAAGTGATAGCATGCATTTAGGGCTTCTTGCTGCCGCTGCTCATGCAGCTGCAAC

AAATAGTCGGTTCACTATATTTTATAATCCAAGGGCTAGTCCATCAGAATTTGTCATAACTCT
CTCAAAATATGTCAAAGCTGTCTATCATACTCGAGTTTCTGTTGGAATGCGCTTTAGGATG
CTGTTTGAAACAGAAGAATCCAGTGTTCGTCGATACATGGGGACGATAACTGGCATAAGT
GACTTAGATCCTGTTCGGTGGCCAAATTCACATTGGAGATCAGTCAAGGTTGGCTGGGAT
GAATCAACAGCTGGTGAAAGGCAGCCAAGAGTTTCCTTATGGGAAATTGAACCATTGACA
ACATTCCCAATGTATCCATCGCCATTTCCTTTAAGGCTCAAGCGCCCATGGCCTCCAGGAT
TACCTTCTTTCCATGGCCTCAAGGATGATGATCTAGGCATGAGTTCACTTATGTGGCTACA
AGGAGATGCTGGTCGAGGAATGCAGCATCTAAATTTTCAGGGTATTGGAGTTTCACCATG
GATGCAGTCGAGGCTAGATGCCTCCATGCTTGGTTTGCAGACTGACATGTACCAAGCTAT
GGCTGCTGCAGCATTGCAAGAGATGAGAACTGTGGATCCTTCCAGATCTGGAACTGCTT
CCCTTCAATTCCAGCAACCCCAAAATGGCCCTTGCAGGCCTGCTGCTCTAGTGCAACCC
CAGATGTTGCAGCAGACTCTGCCTCAGGCCATTCTTCAGGGTGTTGAAGACAACCAGCA
TCAGTCTCAGCCACATCTACTTCAGCAACAATTGCAGCACCAGAATTCATTTAATAACCAA
CAGCAACACCAGCAGCAGCCTTTGTTTAACAACCAACAGCAACAGCAGCCACAGCATTC
AATGTCCCAGCAGCACCAGCAACTGGTTGATCATCAGCAGATTCCTAGTCCAGTGTCTGC
CATGTCACAGTATGCTTCAGCCTCTCAATCTCAGTCATCACCGTTGCAATCCATACCTTCA
CTAGGCCAACAACAGAGTTTTTCTGATTCAAACGGGAACCCTGTGACCAGCCCTGTTGTT
TCTCCTTTACATAGTCTTTTGGGTTCTTTCCCCCAAGATGAGTCTTCCAATCTGCTCAACTT
GCCTAGAACTAACCCCATAATGACTACTTCTGCATGGCCATCTAAGCGGGTTGCCGTTGA
CGTTCTCTCATCTGGATCTCCACAGTGTGTTCTACCACAGGTGGAACAGTTGGGGCCTTC
CCAGACAAACATGTCTCAAAATTCTATTTCGTTGCCACCTTTTCCTGGTAGGGAGTGCTCG
ATAGACCAGGGGGGTACCGACTCACAGAGCCATCTCTTATTTGGTGTTAATATAGAGCCTT
CATCTGTTCTAATGCAAAATGGAATGTCAGGACTTAGGGGAGTTGGCACTGATAGTGATTC
CACTTCTATACCCTTCTTTTCTAATTATATGAGTACTGCAGGGAATGATTTTTCGGTTAATCC
AGCAATGACACCTTCCAGTTGCATTGATGAATCTGGATTCTTGCAGTCTCAGGAAAATGTG
GGCCAATCAAACCCACAAACTAGAACCTTTGTTAAGGTTTATAAATCAGGGTCCTTCGGGA
GATCATTGGATATTTCTGCATTCAGCAACTACAATGAACTTCGCAGTGAACTGGCACATAT
GTTTGGCCTTGAAGGCCAGTTGGAGGACCCTCTGAGATCAGGCTGGCAGCTTGTATTTG
TTGACCGGGAGAATGATGTTCTTCTCCTTGGCGATGACCCCTGGCCGGAGTTCGTGAAC
AGTGTCTGGTGTATCAAGATACTTTCCCCACAGGAAGTGCAGCAAATGGGCAAACGAGG
CCTGGAGCTTCTAAACTCTGTGCCAGTTCAGAGGCTCTCTAATGGCAGTTGTGATGACTA
TGTGAGCCGGCAGGACTCGAGAAATCTTAGCTCTGGTATCGCCTCCGTGGGTTCTTTGG
ACTACTGA
>GrARF6-6
ATGGCACGGGCCCAACAGCAGCACGCTGAATATTGGGCTTATGCAAAGAGTAGGGATTTG
GCTCTAAGGAAATCCCTGCAAAATAATTTTACGAAGCCAATTCCAAAATTTCTTATCTTTCC
AGCCACCTTGCTACCATTTGTTGATGCTGCCGAGACACTCACTCAAGCTGCTACTAAAGC
AACTACTCAACCTGCTACCACAACACCTACTCAAAATGTTGTTGAAGAGATCGAAAAGACG
ATATCCCTAAATGCTGAGAAGGAAGAAGAAGGAGGAAAAAATGATACTACTACCACCACCA
CCACCACCACCAAGGGTAGAGAACCTACTCCACCAGCTCTCGTCACCATATAG
>GrARF6-7
ATGAGACTCTCTTCATCAGGGTTTAATCAGCAAACTGAGGAAGGGGAGAAGAAATGTTTG
AATTCTGAACTTTGGCATGCATGTGCGGGTCCTCTGGTGTCCCTGCCGCCTGTTGGAAG
CCGTGTTGTATATTTTCCCCAAGGTCATAGTGAACAGGTTGCTGCTTCAACCAACAAGGAA
GTAGATGCTCACATACCAAACTATCCGAGCTTACCCCCTCAGCTTATCTGTCAGCTTCACA
ATGTGACCATGCATGCAGATGTAGAAACAGATGAAGTGTATGCTCAAATGACCTTGCAGC
CACTGAGCCCGCAAGAGCAAAAGAATGTGTTCCTCCTGCCTGCTGAACTGGGTACTCCC
AGTAAACAACCAACCAATTATTTTTGCAAAACACTGACGGCAAGTGACACAAGCACTCATG
GAGGATTTTCTGTGCCTCGTCGGGCTGCTGAAAAAGTCTTTCCTCCTCTTGATTACTCCC
TGCAACCTCCAGCTCAAGAGTTAGTTGCCAGGGATCTTCATGAAAATGAATGGAAATTTAG
GCATGTATTTCGCGGGCAGCCAAAGAGGCATCTTCTTACAACTGGGTGGAGTGTTTTTGT
TAGTGCTAAGAGACTTGTTGCTGGTGACTCAGTCCTTTTTATTTGGAATGAGAAGAATCAG
TTGTTGCTGGGTATCCGGAGAGCAAATCGTCCTCAGACTGTTATGCCATCTTCTGTTTTAT
CAAGTGACAGCATGCACATTGGTCTTCTTGCTGCAGCCGCTCATGCGGCTGCAACCAATA
GCCGTTTTACTATCTTTTATAACCCAAGGGCCAGTCCTTCAGAGTTTGTCATTCCTCTCGC
CAAGTATGTTAAAGCAGTCTATTACACCCGGGTTTCTGTAGGCATGCGGTTTAGAATGCTG
TTTGAGACAGAAGAGTCAAGTGTCCGCCGATATATGGGTACGATAACTGGCATTAGTGACT
TAGATCCTGGTCGCTGGCCAAATTCGCATTGGCGCTCTGTTAAGGTTGGCTGGGATGAAT
CCACTGCAGGAGAGAAGCAGCCAAGGGTGTCCTTGTGGGAGATTGAACCATTAACAACC
TTCCCTATGTATTCATCACCCTTCCCCCTGCGATTGAAACGACCATGGCCATCTGCGCTAC

CCTCTTTCCATGCTTTCAAAGATGGTGATATGAACATCAATTCCCAAATGATGTGGCTTCAA
GGTGGGATTGGAGATCAAGGACTTCAGTCTTTAAACTTCCAAGGTTTTGGAGTTGCACCC
TGGATGCAGCCAAGGCTTGATACTTCTTCAATACAAGGTGTCCAACCTGATCTTTACCAAG
CAATGGCTGCTGCTGCATTTCAGGAAATGAGGACTGTTGATTCATCCAAATTAGGCTCTCA
GTCTCTCTTGCAATTCCAGCAACCACAGAGCATGTCCAATGGGTCGCCTGCTATAATTCAA
AGGCAGATGCTACAGCAGTCTCAAACACAAAATGCCTTTCTTCAGGGATTTCAGGAGAAC
CAGACTACTTCCCAGGTTCAGCTTCTGCAGCAGTTGCAGCGTTCCAATTCATACAATAATC
ACCGACAACAACAACAACAACAACAGCAGCAGCAGCGGCAACAACAGCAACAACAGCAA
CAATCTCAGCAAATGCAACAAATACCACAGTTTTCTGATCAACAGCAGATTTCTAATCTAAT
CCCTGCTTTTCCTAAAGCCTCCGGCAGCCAGGCCCAGTCATCATCTCTGCCAACTGCTG
CTTCAGAATGCCAGCAGCCGACATTTTCTGATCCTCTTGGGAACTCTCTAGCCATATCTAA
TGCTTCGTCAATGCAAAGTATCTTAGGTTCATTGTCCCATGCTGGAGCTTCCCATTTACAC
AACTTGAAGGGATCAAACCCAATTGTCTCTTCTTCTTTGTTGTCCAAGCCAGTAGCTATAG
AACCACAGCTTTCGTCTGAAACTGCTAACTACATACTGCCCCAGGTTGAACATCTGGGAA
TGGTACAGTCAAATGTACTCTCTAACGTATTACCTCCATTTCCAGGAAGAGAGTATTCTGC
GTACCAGAGTTCAACTGATACACAAAACAATTTTCTCTTTGGGGTTAGCATTGATTCATCAT
CTCTTGTGCTGCAGCATGGGATGACCAACCTGAAAAATATTGGAAATGAAAATGATTCATT
GTCTCTGCCATATGCCGCTTCAAATTTCACAAGTGCTTCTGGCACAGATTTCCCTCTTAAT
TCAGATATGACTACCTCAAGTTGTGTGGATGAATCAGGTTACTTGCAGTCTTCTGAATATGT
GGACCAAGTAAACCCTCCGACTGGAACCTTTGTTAAGGTTCACAAGTTGGGGTCCTTCG
GGCGCTCATTGGATATTTCCAAGTTTAGCAGCTATAATGAGCTGCGTTGTGAGCTTGCTCG
AATGTTTGGTCTGGAAGGCCAACTAGAGGACCCTCAGAGATCAGGCTGGCAGCTTGTATT
TGTTGACAGGGAGAATGATATTCTTCTCCTTGGTGATGACCCTTGGCAGTAA
>GrARF7-1
ATGAAGGCTCCACCAAATGGATTTATGGCAAATTCTGCAGAAGGAGAAAGGAAGAGTATC
AATTCAGAATTATGGCATGCTTGTGCTGGACCACTTGTTTCTTTGCCTCCAGTTGGAAGTT
TGGTTATTTACTTTCCTCAAGGTCACAGCGAGCAGGTTGCGGCGTCGATGCAAAAGGAG
ACTGATTTCATACCAAGCTACCCTAACCTTCCTTCCAAGTTGATATGCATGCTCCATAATGT
CACATTGCATGCCGATCCGGAAACAGATGAGGTGTATGCCCAGATGACACTTCAACCTGT
GAACAAATATGACAAGGAAGCGTTACTGGCATCTGATATTGGCCTCAAGCAAAGCCGGCA
ACCTGCTGAGTTCTTTTGCAAGACTCTTACAGCAAGTGACACTAGCACTCATGGTGGATT
TTCTGTGCCTCGACGAGCAGCTGAGAAAATCTTCCCTCCCCTGGATTTCTCGATGCAACC
ACCTGCTCAGGAGCTAGTAGCTAAAGATTTACATGACAATACATGGACATTTAGACATATTT
ATCGAGGTCAACCAAAGAGGCATCTTCTGACTACTGGTTGGAGTGTCTTTGTTAGCACAA
AAAGACTCTTTGCCGGTGATTCTGTTCTTTTCATAAGGGATGAGAAGTCTCAACTTCTGTT
GGGTACAAGGCGTGCAAATAGACAGCAGCCAGCTCTTTCTTCATCAGTGATTTCTAGTGAT
AGCATGCATATAGGGATCCTGGCTGCTGCAGCGCATGCTGCAGCAAATAACAGCCCATTT
ACTATATTCTATAATCCAAGGGCAAGCCCTTCTGAGTTTGTGATACCCTTGGCAAAGTTTAA
CAAAGCCATGTATACCCAAGTTTCCCTTGGCATGCGGTTTAGAATGATGTTTGAAACCGAG
GAGTCTGGAGTACGCAGGTACATGGGTACAATTACTGGTATCAGTGACTTGGATCCTGTG
CGATGGAAAAATTCACAGTGGCGCAATCTTCAGGTTGGATGGGATGAATCTACAGCTGGT
GAGCGGCCCAGCCGAGTTTCAATTTGGGACATTGAGCCTGTTATAACTCCTTTTTACATCT
GTCCACCTCCGTTTTTCAGGCCCAGGTTTCCAAAGCAACCGGGGATGCCAGATGATGAG
TCTGACATTGAGAATGCCTTCAAGAGAGCTATGCCCTGGCTAGGAGATGACTTTGGTATG
AAAGATGCTCCTAGTTCAATCTTTCCTGGTTTGAGTCTAGTCCAGTGGATGAGCATGCAAC
AAAATAATCAGTTTCCAGCTGCTCAATCAGGATTCTTTCCATCAATGGTTTCTTCGAATCTG
CTCCATAATAGCCTTGGCACCGATGATCCTGCCAAATTATTGAACTTTCAAGCTCCTGCGC
TTCCAGCATCAAATATGCAATTTAACAAAGCAAATACGAACCAAATCAATCAGTTGACTCAG
GCACCTATGACATGGCCCCAACAGCAGCAACTTCAACAGTTATTGCAGGCTCCTTTAAAT
CAGCAGCAGCAATCCCTACAGCAATTGCAGCGACAACTGCCACAACCACCACAGCAGCA
GCCTCAGCCGCATCTTTTTCACCAGCAGCAGCCTCAGCCTCAGCCTCAGCCTCAGCCTC
AGTCTCAGTCTCAGTCTCAGTCTCAGTCTCAGTCACAGTCGCAGTCGCAGTCACAGTCA
CAGCCACAGCCACAGCCACAGCCACAGCCACAGCCACAGCCACAGCCACAGCCACAGC
CACAGCCACAGCCACAGCCACAGCCACAGCCACAGCCACAGCCACAGCCACAGCCACA
GCCACAGCCACCTCCACTGCAGCAGCAGCAGCAGCAGCTGCAGCAACAACATCAAAAAA
GACAACAGACGCAATCACAGCAGCAGCTGCAACAAGCATTCCTCCCTTCTCAAGTAAATA
ATGGCATCATTGCCTCTAACCAGATCTCAAATCAAAATTTGCACCAGCCAGCTGTTTACTC
ACAGCTTCAGCAGCAACAATTGATGACAAGCAGTAGCCAATCTGCCCAGACTACCGCCTC
TGCCAATAAAACTTCATATCCTTTGACATCATTACCGCAAGATACACAGATTCAGCAACAGA
TGGAACAGCAACCTAACCTCATGCAGAGGCAGCTGCAACAGACACAGTTGCAGCAGTCA

CAACTACAATTATTGCAACAAAGCCTGTCCCAGAGGACACAGCAGCAGCCACAGCTTCTG
CAATTGTCACAGCAGGGCTTCTCGGAGCAACAGCTTCAGTTGCAACTGTTACAGAAATTG
CAGCAACAGCAGCAGCAGCAGCAGCAATCATCTCAACAATTACTCTCCCCAGCTGGATCA
CTGTTGCAGCCTCAAATGTTGCAGCAGCAGCCGACCCATCAACAGAGCCAACCATTGCA
GCAATTGCCTCTTTCACAAAGCCAATCACAACCTCTTGGCAGCAATGGCTTCTCAACATC
AATGCCTGTTCAACCTCAACAGGTTTCAGTGAACCAACCCCAAATTCACAAGCCATTTACT
GCTATGAGAACTCATTCTGGTCTTACTGACGGAGATGCTCCATCATGTTCAACCTCACCTT
CTACCAATAACTGTCAGGTTTCCTCGTTGAACTTTTTAAACAAAAATCAGCAAGTTCCATCC
TTATTGGTGTCAGATCCTCTTGTTGAGCCTGCAAGTACCCTGGTTCAAGAGCTTCAGAGC
AAGCCTGATATTAGAATCAAACATGAACTACCCACCTCTAAAGGACCAGAACTATCAAAGT
ACAAAAGTACTGTGACAGATCAATTAGAAGCATCCTCTTCTGGAACATCATACTGCCTGGA
TGCGGGCACCAACCAGCATAATTTCTCCCTCCCCACCTTTTTGGAAGGTGATCTTCAATC
ACACTCCCGGAACAATCTTCCTTTTACAGCAAATATTGATGGATTGGCACCTGACACTTTG
TTATCAAGGGGATATGACTCTCAAAAGGATCTTCAAAACATGTTGTCGAACTATGGTGGCA
ACCCAAGAGATATTGATGCGGAGTTGTCTACTCCTGCGATAAGCTCTCAGTCGTTTGGTG
TGCCAAACATACCTTTCAAGACTGGGTGCTCAAATGATGTTGCTATCGGTGACACAGGAG
TCCTAAATGGTGGACTGTGGGCCAACCAAACTCAACGCATGCGAACATATTCAAAGGTGC
AAAAGCGTGGGTCTGTAGGAAGGTCAATTGATGTGACCCGCTACAAAGGGTATGATGAGC
TCAGGCATGATCTAGCGCGCATGTTTGGTATAGAGGGGCAACTGGAAGATCCGCAAAGTT
CTGACTGGAAATTAGTTTACGTGGATCATGAAAATGACATATTACTTGTTGGTGATGATCCT
TGGGAAGAATTTGTAAGTTGTGTTCAGAGCATAAAGATACTGTCATCTGGGGAAGTACAGC
AGATGAGCTTGGAAGGTGATCTTGGAAATGTGGCAGTTCCGAATCAAGCTTGCAGTGGG
ACTGACAGTGGAAATGCATGGAGAGGACAATATGATGATACCTCAGCAGCGTCATTTAACA
GATAA
>GrARF7-2
ATGAAGGCTCCACCCAATGGATTTTTGGCAAATCCCGCAGAAGGAGAAAGGAAGAGTATC
AATTCAGAATTATGGCATGCTTGTGCTGGACCACTTGTTTCTTTACCACCAGTTGGAAGTT
TGGTGGTTTACTTTCCTCAAGGCCATAGTGAACAAGTTGCAGCATCGATGCAGAAGGAGA
CTGATTTCGTACCAAGCTACCCTAATCTTCCTTCCAAGTTAATTTGCATGCTCCATAATGTC
ACATTGCATGCTGACCTGGAAACCGACGAGGTCTATGCTCAGATGACTCTTCAACCTGTA
AACAAATATGACAGAGAAGCATTACTAGCATCTGATATGGGCCTCAAGCAAAGCAGACAAC
CTGCTGAGTTCTTTTGCAAGACTCTTACAGCTAGTGACACTAGCACTCATGGTGGCTTTTC
AGTGCCTCGTCGAGCAGCTGAGAAGATCTTCCCTCCTTTGGATTTTTCGATGCAACCGCC
CGCTCAAGAGCTAGCAGCTAGAGATTTACATGAGAATACATGGACATTTAGACATATCTATC
GAGGTCAACCAAAGAGGCATCTTCTAACTACTGGTTGGAGTGTCTTTGTTAGCAACAAAA
GACTTTTTGCTGGTGATTCCGTTCTTTTCATAAGAGACGAGAAGTCACAGTTTCTCTTAGG
TATAAGGCGGGCCAATAGACAGCAGCCAGCTCTCTCTTCATCAGTAATTTCTAGTGATAGC
ATGCATATAGGGATCCTTGCTGCTGCAGCTCATGCTGCTGCAAATAACAGCCCATTTACTAT
ATTCTACAATCCAAGGGCAAGCCCTTCCGAGTTTGTGGTACCCTTGGCGAAGTATAACAA
AGCCATGTATACCCAAGTTTCTCTTGGAATGCGGTTTAGAATGATGTTTGAAACCGAGGAG
TGCGGAGTACGTAGATACATGGGTACAATTACTGGTATCAGTGACCTGGATCCTGTACGAT
GGAAGAATTCACAGTGGCGCAATCTTCAGGTAGGATGGGATGAATCTACTGCTGGTGAGC
GACCAACCCGAGTTTCAATTTGGGACATTGAGCCTGTTGTAACTCCTTTCTACATATGCCC
ACCACCGTTTTTCAGGCCTAGATTCCCCAAGCAACCGGGAATGCCGGATAATGATAATGAT
GTTGAGAATGCTTTTAAGAGAGCTATGCCTTGGCTTGGAGATGACTTTGGCATGAAAGATT
CCCCCAATTCAATCTTCCCTGGTTTGAGTTTAGTTCAGTGGATGAACATGCAACAAAATAA
TCAGTTGGCAGCTGCTCAATCTGGACTCTTTCCTTCAATGGTTTCTTCGAATCCGTTGCAC
AATAGCCTTGGCACTGATGATCCATCCAAGTTATTGAATTTTCAAGCTCCGGTGTTACCTG
CGTCAAATATGCAATTTAACAAAGCAAATCCAAACCAGGTCAACCAGTTGTCTCACGCACC
TATGACCTGGCCCCAGCAACAGCAACTCCAGCAGTTGTTGCAGACTCCTCTGAATCAAAA
TCCACTACAGCAACAATCCCAACAGCAGTTACAGCAAAGACAGTCACAGCTGCAGCCAC
AGTCACATCTTCAACAGCAGCCTCAGCCACCTCTACAGCAGCAGCAGCAGCAGCAGCAA
CAACAAAGACAACAGGCACAGCCACAACCACAGCAGCAGCAGCCACCTCTACAGCAACT
GCAACAACAACAAAGACAACAGGCACAACCGCAGCTGCTGCAACAACAATTTCTACCAG
CTCAAGTAAATAATGGCATCATTGCCTCAAACCAGATGCCAAATCGAAATTTGCATCAGCC
TGGTGTTTATTCTCAGGTTCAGCAACAGCAGCAACAACAACAAATATTGACAAGCAATGGC
CAGTCAACTCAAACCACCCTTCCTGCTAATAAAGCTTCATATCCTTTGACGTCGTTAGCTC
AAGATACACAGATTCAACAACAGGTGGAACAGCAACCTAACCTCGTACAGAGCCAACAGC
AACAGACACAGTTGCAGCAAAATCTGTCCCAGAGGTCACAACAGCAGCCGCAAATTCAA
CAACTGGCACAGCAGGGCCTCCCAGAGCAACAATTCCAATTATTACAGAAATTGCAGCAA

CAGCAGCAGCAGCAGCAGTCATCTCAACAATTACTCTCGCCTACTGGATCACTGTTGCAG
TCTCCAGTGGTGCAACAACAGCCAATGCATCAACAAAACCAACCAATGCAGCAGGTGCC
TCTTTCTCAGAGTCAACAGACACTTGGCAGCAATGGTTTCTCAACATCAATGCTAATGCAA
CCACAACAGCTAGTGGTGAGCCAATCCCAAAATCAGAACAAGCCACTTATGGCAATGAGA
ACCCATTCTGGTCTTACTGATGGAGATGCTCCATCATGTTCAACCTCACCTTCTACCAATAA
TTGTCAGGTTTCACCATCAAGCTTCCTAAGCAGAAGTCAGCAAGTACCATCCATGGTGGT
GACAGATCCAGTTGTTGAGCCTGCAAGTACACTGGTTCAGGAGCTTCAGAGCAAGCCTG
ATATCCGAATCAAACATGAGTTGCTTGCCTCTAAAGGTCCAGATCAATCAAAGTACAAAAG
TACTGTGACAGACCAGTTAGAAGCATCCTCTTCTGGAACATCATACTGCTTGGATGCAGG
CACCATCCAGCATAATTTCTCTCTCCCCACTTTTTTGGAAGGTGATGTCCAATCACATCCT
CGGAACAATCTTCCATTTTCAGCTAATATTGATGGACTGGCACCCGACACCTTGTTATCAA
GGGGATATGACTCTCAAAAGGATCTTCAAAACCTGCTTTCTAATTATGGTAGCAATCCAAG
AGATATTGACACAGAGTTGTCTACTGCTGCAATAAGTCCCCAGTCATTTGGTGTGCCAAAC
ATACCTTTCAAAACTGGGTGTTCAAATGATGTTGCCATAAACGATGCAGGAGTTTTAAATG
GTGGATTGTGGGCCAACCAAACTCAACGGATGCGAACATATACAAAGGTACAAAAGCGTG
GCTCTGTGGGAAGATCAATCGATGTGACCCGCTACAAAGGGTATGATGAACTCAGGCATG
ATCTAGCCCGCATGTTTGGTATCGAAGGACAGATGGAAGATCCACAAAGTTCTGACTGGA
AATTAGTATATGTTGACCATGAAAATGACATATTACTTGTTGGTGATGATCCTTGGGAAGAA
TTTGTAAGTTGTGTTCAGAGCATTAAAATACTGTCATCCCTTGAAGTACAACAAATGAGCTT
GGATGGTGACCTTGGAAATGTGCCAGTTCCCAATCAAGCTTGTAGTGAGATCGACAGTGG
AAATGCATGGAGAGGACATTATGATGATACTTCAGCTGCCTCCTTTAACAGATAA
>GrARF8-1
ATGAAGCTTTCATCATCAGGCCAGGGTCAGCAGGCTCATGAAGGGGAAAACAAGTGCTT
GAACTCAGAGCTATGGCATGCCTGCGCTGGCCCACTGGTTTGTCTACCAACTGTGGGGA
CCCGTGTGGTCTACTTTCCTCAGGGTCATAGCGAGCAGGTAGCAGCCACCACTAACAAG
GAAGTTGACACCCACATTCCGAATTACCCCGACTTGCCTCCTCAGTTGATATGTCAGCTC
CAAAATGTTACCATGCACGCTGATGTTGAGACAGATGAAGTATATGCCCAAATGTCGTTGC
AGCCTTTGACGCCTGAAGAGCAGAAGGATACATTTCTTCCTATGGAGTTGGGAATTCCAA
GCAAGCAGCCAACCAATTATTTCTACAAGACACTGACAGCAAGTGACACTAGTACCCATG
GTGGTTTTTCTGTTCCTCGTCGTGCTGCTGAGAAAGTCTTTCCGCCACTGGACTTCTCAC
AGCAGCCTCCAGCTCAGGAACTTATTGCAAGGGATCTTCATGACATTGAGTGGAAATTTA
GACATATATTTAGAGGACAGCCTAAACGCCACCTTCTTACTACAGGCTGGAGTGTGTTTGT
TAGTGCCAAGAGACTTGTTGCAGGGGATTCTGTTCTTTTTATTTGGAATGAAAAGAACCAG
CTTCTTTTGGGAATTCGCCGTGCCACTCGCCCACAAACTGTAATACCATCATCTGTTTTAT
CTAGTGATAGCATGCTCATTGGACTACTTGCTGCTGCAGCTCATGCTGCGGCAACTAATAG
CTGCTTTACAGTATTTTATAATCCAAGGGCTAGTCCATCTGATTTTGTAATACCCCTTTCAAA
ATATGTCAAAGCTGTTTTCCACACACGTGTCTCGGTTGGGATGCGTTTTCGGATGCTTTTT
GAGACAGAAGAATCAAGTGTTCGTAGGTATATGGGTACAATAACTGGTATAAGTAGTTTGG
ATCCTGTTCGTTGGCCAAATTCCCATTGGCGGTCTGTGAAGGTTGGTTGGGATGAGTCAA
CAACTGGTGAGAGGCAGCCAAGGGTATCATTGTGGGAGATTGAGCCTTTAACAACATTTC
CAATGTATCCATCTCTATTTCCCCTCAGATTGAAACGCCCTTGGCATCCGGGTTCCTCATC
TTTGTTTGATAACAGAGATGATATATCTAATGGGTTATCGTGGCTAAGGGGAGGATCTGGA
GAGCAAGGACTACAATCTCTGAATCTTCAGTCCATCTGCTCACATCCATGGGTGCAGCAG
AAACTAGATTTGAGTTTCCCTGGAAATGATTATAATCTGCAGTACCAACATATGCTGGCCAA
TGGGTTGCAGAACTTGGGAAGTGGGGATCTGCTGAGACAGCAGTTGCAGCAATCTTTTC
AATATGTTCAACAGCCAGGCAGCCATAATTTACTGTTGCAGCAGCAGCAGCAGCAGCAGC
AGCAGTCTGTTTCACAGTTAGTTCCACACAACATTGTGCAGGCACAGCAATCCCAAATTCT
AATCGAGGGTTTTCTCCCAGTCCCTGGACGGGAACAAGTAGGCAATCAATCGGAGGAAC
TGGCTCAGCAACAGCATAACATGACTCAAAGTGATCAGCACCAGCAGAGGCGGCCGGTG
AATGTGCCTTCATTTCTGAAACCAGATTTTATTGACTCAGGATCTGGTCCCCCTGTTCTGG
ATATGCTGGGTTCATTGTGTCCTGAAAGCAGTGCAAATCTTTTGAACTTCTCCACAACTGG
TCAGTCAATGCTAGCTGACCAGTTACCCCAACTGTCCTGGGCTCCGAAGTATGCTCATTC
AGATGTAAATGCCTTTGCTGGCTCAACATCAGGTCCACAAGTTTCTCCTGGAAAAGATGCT
ACGATAGAGCTAGATATTGGTACCTCTGATGCTCAGAACTCTACTGTTTTTGGTGTTAACG
ACGATTCATTTGGCCTTCTACTGCCCACCTCAATGCATGGTTTCACTACATCTTCAAGTGA
AGCTGATATGCCCTCAATTCCATTAGGGGATCCCTCATTCCAGAATCCTTTGTATGGTTGC
ATGCAATACTCTTCAGAGTTGCAGAGCACAGGGCAAGTTGACCCACCAACTTCATCTCAA
ACATTTATCAAGGTTTATAAATCAGGGTCGGTGGGGCGCTCATTGGACATCTCCCGGTTCA
GCAGCTACCATGAGCTGCGAGAGGAGCTGGCTCAGATGTTTGGAATTGAGGGGAAGTTG
GAAGACCCTCTTAGATCAGGCTGGCAGCTTGTATTTGTCGACAGGGAGAATGATATTCTTC

TCCTTGGAGACGACCCATGGGAGGCATTTGTGAACAATGTTTGGTACATAAAGATACTTTC
ACCAGAGGATGTGCAGAAGATGGGATAG
>GrARF10-1
ATGAGAAAAGAGGCAGAAAAGAGCTTGGATCCTCAGCTATGGCATGCCTGTGCTGGATC
CATGGTTCAAATTCCACCAGTGAACTCCAAAGTCTTCTACTTTCCTCAAGGCCATGCCGA
ACACTCTTTATCTCCGGTCGATTTTTCATCTTCTCCTCCAATCCCGGCTCTAGTTCTTTGCC
GGGTGGCTTCTGTCAAATTCTTGGCTGATGCTGAAACCGATGAAGTGTATGCCAAGATCA
TGCTTGTACCATTGCCCAACACTGAGCCTGATCTCGAGAACGTTGCCGTTTTGGGTGGTG
GGTCTGATAATGTGGAAAAGCCTGCTTCTTTTGCTAAGACATTGACTCAATCCGATGCAAA
CAACGGGGGTGGTTTTTCGGTTCCCAGATATTGTGCTGAAACCATATTTCCGAGACTGGA
TTACACTGCCGATCCTCCGGTTCAAACTGTGATTGCCAGGGATGTTCATGGTGAGATTTG
GAAGTTTAGGCATATCTATAGGGGAACACCTAGGAGACATTTATTGACTACCGGCTGGAGT
AGTTTCGTTAACCATAAGAAACTTGTTGCTGGGGACTCCATTGTGTTCTTGAGAGCCGAA
AACGGTGAGCTTTGTGTTGGGATAAGGCGGGCCAAGCGGGGGAATGATACTGGAGCTGA
ATCTGGTCTTGGAAATGGAAACTACGTTAGTCCTTATGGAGGGTTTTCAGGTTTCTTGAAG
GAGGATGAGAGTAAGATAACCCGAAAAGGGAATCCGAGAGGGAAGGGAAAAGTGAGAG
CAGAGGCTGTTGTGGAGGCAGTGGGGCTTGCCGCTAATGGCCAGCCTTTTGAGATAGTT
TACTATCCGAGAGCAAGCACACCGGAGTTTTGTGTTAAGGCATCTGTAGTAAGGGCGGCA
ATGAGGGTTCCTTGGTGTTCTTTGATGAGGTTCAAGATGGCTTTCGAGACCGAGGATTGT
TCTAGGATTAGTTGGTTCATGGGGACTGTATCTTCTGTTCATATTGCAGACCCCCTTCGAT
GGCCTAATTCCCCGTGGCGCCTTCTTCAGGTAACATGGGATGAACCAGATTTGCTGCAAA
ATGTTGAACGCGTGAGTCCCTGGTTGGTTGAATTGGTACCTAACATGCTTCCCGTCCACC
TGTCACCCTTCTCAACAGTGACCCCCAGAAAGAAGTTGAGGCTTCCTGAGCACCTCGAT
TTCCCCCTTGTTGAACAATTTCCGATGCCATCGTTTTCCGGTCATCCCCTCAGGTCAAGC
AACCCATTATGTTGTTTATCTGATAATGCTCCTGCAGGCATACAGGGAGCCAGGCATGCTC
AAGTCAGATTATCTTCATCAGATCCCCATCTTAATAAACTGAAGTCCGGACTGTTTCCGTCT
GGCTTCCAGCTGTTTGATCCGCAAGCTCGAGTTCCTAATGGCATCTCAATGACAAAGCAC
ACAGACAGTAACGATGATAATCTATCGTGCTTATTAACAGTAGGGAATTCTAGTCCAAAGAA
GAAATCTGAAAACGGAAAGAGACACCAGTTTTTACTCTTTGGTCAGCCAATACTTACCGAG
CAGCAGCTCTCTCGGAGCTGTTCAACTGGAGTAAAAACTGCTCTCGAGAATGAAGACAAA
AGAAAAGATTATTCCAATGGTTCAGAATCTGCTCTCGAGAATCAATTGTCTCCAGAGAAGT
CATTTACTACCAGATTATTGTGGCAGCAGGACTATCAAGCCCCAGAACCTGGCTCAGCCA
CTGGTCATTGCAAGGTATTCTTGGAATCTGAGGATGTGGGACGAACTCTTGACCTCACAG
TTCTCGGTTCTTATGAAGATCTATACATGAGGTTGGCTAACATGTTTGGAAGAGAAAGATC
AGAGATGTTGGGCCATGTCTTGTATCGAGATGCAACAGGTGCTGTCAAACAAACTGGAGA
TGAACCATTCAGTACTTTTATGAAGACGGTCAAAAGATTGAACATAAGGATGGATTCACGC
AATGATACCGATGGAAGGTCATGGCTCACAGGGATTCGAACTGCCAAAAACAGACTAGAA
GGGCCAAATAAGAGAGGTCCCTTGAGCATATTTGCATGA
Cotton_D_gene_10017685 locus=scaffold167:15265:18856:- >GrARF11-1
ATGTCTCCTCTGAAGTTGGAAGCATCCACGAATCAGACGTTGAATCAGAGGATTCCGTTG
TTTAATCTTCCATCGAAGATTCTTTGCCGTGTCGTTCATATTCAGTTGCTGGCTGAAAAAG
AAACAGATGAAGTTTATGCACAAGTGACTTTGTTGCCGGAACCAAGTCAACCTGAGCCAA
CAACCCCTGATCTGTGCCCCCCTGAGTCTCAAAGACCTACAGTCCACTCATTCTGCAAGG
TTTTAACTGCGTCAGATACAAGCACTCATGGAGGATTTTCTGTTCTTCGCAAACATGCTAC
TGATTGCCTTCCTAAACTGGACATGAATGATGCAACACCAACCCAGGAATTGGTTGCAAA
GGATCTTCATGGTTATGAGTGGCGCTTTAAGCATATTTTCAGAGGCCAACCAAGGAGACAT
TTGCTCACAACAGGATGGAGTACGTTTGTTACTTCAAAGAGATTAGTAGCTGGTGACTCCT
TTGTGTTTCTGAGAGGGGAAAATGGCGAGCTACGTGTTGGAGTGAGGCGTGTTGTTCGC
CAACATAGCATCATGCCATCGTCAGTGATTTCAAGTCAGAGCATGCATGTAGGAGTGCTTG
CAACTGCATCTCATGCTGTTTCAACACAAACCCTCTTTGTTGTCTACTATAAGCCAAGGAC
AAGTCAGTTCATCATTGGGTTGAACAAATATTTAGAGGCTCTTAGTAATAAGTTTGCTGTTG
GCATGAGATTTAAGATGAAGTTTGAAGGGGAGGATTCTCCTGAGAGAAGGTTTTCTGGCA
CGATTGTGGGGGTTGAAGATTTTTCTCCTCTTTGGAAAGATTCAAAATGGCGATCATTGAA
GGTACAATGGGATGAACCTGCCTCTATCCCAAGGCCTGATAGGGTTTCACCGTGGGAGAT
AGAACCCTTTGCTGCTCCCATTCCACCATCTTTGACTCAACCAGTTGCAGCTAAGAACAA
AAGGCCTCGACCACCTGCTGAAATTCCTGCCCCAGATTTATCTTCAACAACATCAGCTCC
TTATTCTGGAGTTACGCATTGCCATGATCTAACACCACAAAACATTGCTGCCGAAGCAAAA
GGAAATGAAAATCCTGTTATTGGGCACCACATGTGGACAGAGATGAGCAGTGGTTGCAGC
TCTGTCGCAAAGGCTCTGAATAAAGGAAGTTGGCTATCTTCTCCTGGCACATGTGTTCCT
CATCATCTGTTTCCTGATGCAAGAGAGGATAAAACGAGATTCTCTGCTTGGCCTGTTCCTT

CGGGTTTTTCAAACCCACAGCTCAACGAAGATTCAACCTTTGATTCAACTGAAAAGGCTA
AGAGATCTGAGACAGCTGCTAGCTGCCGATTGTTCGGTATTGATCTGATAAACCATTCTAC
AAGCTCAACACCGTTGGAGAGAACACCTGCACAACTTTCTACTATGATAATGGGTACTGAA
GTACCAGGCCCAAGTACTCTATCATCCACTTATTCTGATCAGAAGTCTGAAATTTCAAAAGA
TTCTAAAGAGAAGAAGCTGGAACAATTACAGTTATCAGCAAAAGAGAACCAGAGCATGCA
AAGTTGTTCTAGTTTTACAAGAAGTCGTACAAAGGTTCAAATGCAGGGGATAGCCGTAGG
TCGTGCTGTGGATTTGGCCATGTTTGAAGGGTATGGTCAGCTTATAGATGAACTGGAGGA
GATGTTTGATATTAAGGGAGAGCTTCGCCCTAGGAATAAGTGGGAGATTGTCTACACTGAT
GATGAAGGTGATATGATGCTTGTAGGCGATGATCCATGGCCGGAATTTTGCAACATGGTAA
GAAGAATATTTATTTGTTCAAGCCAAGATGTGAAAAAGCTGAAGACAGGAAGCAAACTTCC
CTTGGCTTCTATCGAAGGTGAAGGGACGGTAATAAGTTCAGACTCATCTGAAAACTAA
>GrARF11-4
ATGGCGAATCGGGGAGGGGTGTTTTCACAGACAAATAATGTTTCCTCCGAAGGAAATGGA
GGAGATGATCTATACATGGAGTTATGGAAGTTATGTGCGGGACCCCTGGTTGAGGCTCCT
CGAGCTAGAGAGAGAGTCTATTATTTTCCTCAAGGACATATGGAACAGTTGGAAGCGTCG
ACGAATCAGGAGTTGAATCAGAGGATTCCTTTGTTTAATCTTCCATCGAAGATACTTTGTAG
TGTTGTTCACATTCAGTTTCTGGCTGAACAAGAAACAGATGAAGTTTATGCACAAATAACT
TTGATGCCGGAGCCAAATCAACCTGAGCCAACAACTCCTGATGCATGTCCTCCGGAGCC
TCCAAAACCTACTGTCTGTTCTTTCTGCAAGGTTTTAACAGCCTCTGATACCAGCACTCAT
GGAGGTTTTTCTGTTCTTCGCAAACATGCCACTGAATGCCTTCCTCCACTGGACATGAAC
CAGGCAACGCCAACCCAGGAATTGGTTGCCAAGGATCTTCATGGCTATGAGTGGCGCTTT
AAGCATATTTTTAGAGGCCAGCCAAGGAGGCATTTGCTCACAACGGGATGGAGTACATTT
GTTACTTCCAAGAGATTAGTAGCTGGTGATTCCTTTGTGTTCTTGAGAGGGGAAAGTGGG
GAGCTGCGCGTTGGAGTGAGGCGTGTTGCTCGTCAACAGAGCAGCATGCCTACATCAGT
GATTTCAAGTCAGAGCATGCATTTAGGAGTCCTAGCAACTGCATCTCATGCCGTTTCAACA
CAAACCCTCTTTGTTGTCTACTATAAACCAAGGACAAGTCAGTTCATTATTGGGTTGAACA
AATATTTAGAGGCTCTTAACAATAAGTTTACAGTTGGCATGAGGTTTAAGATGAGATTTGAA
GGGGAGGATTCTCCTGAGAGAAGGTTTTCTGGCACAATCGTTGGGGTTGAAGACTTTTCT
CCTTACTGGAAAGATTCAAAATGGCGTTCATTGAAGGTACAATGGGATGAACCTGCCTCTA
TACCAAGACCTGACAGGGTTTCACCATGGGAGATAGAACCCTTCGCTGCTCCTATTCCAC
CAACTCTGGGTCAACCTGTTGCTGCTAAGAACAAAAGGCCTAGACCAACTGCTGAAATTC
CTGCTCTAGAATTACCTTCAACAGCATCAGCTCCTTGGAATTCTGGAGTTATGCATACCCA
TGATCTAATGCGGCGTAACATTACTGCCGAAGCAAACAGAAATGAAAATCATGTCATATGG
CATATGCAGACTGAAATGAGCAGCAGTTGCAGCTCTGTCTTGAAGACTCAGAACGAAGG
GAGCTGGCTATCTTCTCCTTGCATGAGTGTTTCTAAGCATCGATTTCCTGATGCAAGAGAC
GATAGCAAATGTGCATCTGGTTGGCCTGTTCTTTCAGGATTGTCAAACCCACAAGTGAAC
AATGATTCAACCTTTGATCCAATAGAAAAGGTAAAGAAATCTGAGACAGCTTCTAGCTGCC
GATTGTTTGGCATTGAGTTGATAAACCACTCTGCTAGCTCAAGGCAATTGGAGAGGACAC
CTACCCAACTTTCTACTATGACTGCCAGTACAGCTGAAGGACATCATACCTTGTCACCTAAT
AATTCTTGCCAGAAGTCTGAAATTTCAAAAGATTCTAATGAGAAGCAGGAACAGTTACAATT
ACAAGCAAAAGAGATCCAGAGCAGGCAAAGTTGCCCTAGTTCTACAAGAAGTCGTACCAA
GGTCCAGATGCAGGGGGTAGCCGTCGGTCGCGCTGTGGACCTGGCCATGTTGGAAGGG
TACGATCAGCTTATAGATGAACTAGAGGAGATGTTTGATATTAAGGGAGAGCTTCGCCCTA
GGAATAAGTGGGAGATTGTCTACACTGATGATGAAGGGGATATGATGCTTGTAGGCGATG
ACCCATGGCAGGAATTCTGTAGCATGGTAAGAAGAATATTTATCTGTTTAAGTCAGGATGT
GAAAAAGCTGAGTACAGGAAGCAAACTTCCCATGGCTTGTATTGAAGGTGGTGAAGGAA
CTGTGATAAGCTCAGAGTCAATTGAAAATTAA
>GrARF16-1
ATGAAGGAATCAGATAAGAGCTTAGATCCACAGTTATGGCATGCATGTGCTGGGCCCATG
GTTCAAATCCCACCATTAAACTCCAAGGTCTTTTACTTCCCTCAAGGCCACGCCGAGCAC
TCACTCGCCGCCGTCGATTTCCCCTCGTCTCCGCCGGTCCCGGCTCTAGTTCTTTGCCG
AGTGGCTTCGCTCAAATTCATGGCGGATACCGAAACCGACGAGGTATACGCCAAGATCCT
CCTCATGCCGTTACCCAACACAGAGCTCGACCTAGAACACGTCGCCGTTTTCGGCTCCG
ATAACGCGGAGAAGCCTGCTTCTTTCGCTAAGACATTGACCCAATCCGACGCCAACAACG
GCGGTGGGTTTTCGGTCCCGAGGTACTGCGCCGAAACCATTTTCCCGCCGTTGGATTAC
ACGGAGGACCCTCCGGTTCAAACCGTCGTCGCCGTGGATGTCCACGGTGAAACGTGGA
AGTTCAGGCATATTTATAGAGGGACCCCGAGGAGGCATTTATTGACTACGGGATGGAGCA
CTTTCGTGAACCATAAGAAGCTCGTTGCCGGCGATTCCATCGTGTTCTTAAGGTCCGAAA
ACGGCGGCCTGTGCGTCGGGATCCGACGAGCCAAGCGCGGGACTGGAAACGGACCCG
AAGCTGGGTCTCCATTTTTGTCTTTCTTAAGGGAGGACGAGAGTAAGATGATGATGATGAA

CCGAAATGGGGATTGGAGAGGAAAGGGGAAACTGAAGGCGGAAGCTGTTTTACAGGCG
GCTACGTTGGCGGCCAGCGGTCAGCCGTTCGAGGTTGTTTATTACCCAAGAGCGAGCAC
ACCGGAGTTTTGCGTTAAGGCGTCGTCGGTGAAGGCGGCAATGAGGGTTCCTTGGTGTT
GTGGGATGAGGTTTAAGATGGCTTTCGAGACAGAAGATTCTTCAAGGATTAGTTGGTTCAT
GGGGACTGTGTCTTCTGTTCAAGTTGTCGATCCCATTCGGTGGCCTAATTCCCCATGGCG
ACTTCTTCAGGTAACATGGGATGAACCGGATTTGCTTCAAAACGTTAAACGTGTTAGTCCC
TGGTTGGTTGAATTGGTATCGAATGTGCCTGCCGTCCACTTGTCGCCGTTCTCCCCACCG
AGAAAGAAGTCTAGGTTTCCCCAACATCTCGATTTTCCTCTCGACGGACAATTCCCTATGT
CGGCATTTTCAAGCAATGGCAATCCCCATGGGACCGGCTGTCCGTTAGTTTTATCTGATAA
TGCTCCTGCAGGCATACAGGGAGCCAGGCATGCTCAATTTGGGTTATCTTTATCCGATCT
CCATCTTAATAATAAACTGCAGTCCGGACTTTTTCTGCCCGGTTTCCAGCGGTTCGATCCC
CACTCTAGAATTTCCGATGGCATCATGATGGCAAGGCGCCCTAATGGTACTGATAATCTTT
CTTGCTTGTTAACAATCGGGAATTCTAATGTGAATGAGAAATCTGGCAACACAAAAAGACA
CCAGTTTTTACTCTTTGGTCAGCCGATACTTACTGAGCAGCAGCTCTCTCGTAGCTGTTCA
AGTGAAGTTGTCTCACAAGTTATTAACGGAAATAGTTCGTTAGATGGCAGTGCCGAAAAAA
CAAAAGATACTTCTGATGGTTCTCGATCTTCTCTTGAGAAGTCATCTACTGCTGGATTTTTG
TGGCACCAGGATTATAGAAGCACGGAAACCGGCCTCGATATTGGCCATTGCAAGGTATTC
TTGGACTCGGAGGATGTCGGACGAACCCTTGACCTCTCAGTTCTTGGCTCTTATGAAGAG
CTGTACAGGAGATTGGCCAACATGTTTGGAATAGAAAGATCCAAGATGTTGGGCCATGTG
TTGTATCGAGATGCAACAGGTGCTGTCAAACAAACTGGAGAAGAACCATTCAGTGCATTTA
TGAAAACAGCAAAAAGATTGACAATAAGGATGGATTCAAGCAATGAAACTGTTGCAAGGT
CTTGGCTTACCGGGATTCGAACCGCCGAAAACGGGCTAGGAGGGCCGAGCAAAAGAGG
TCCCTTAAGCATATTTGCGTGA
>GrARF16-2
ATGATTACTTTTATGGAGTCGAAAGAGAAACCGAAAGAGATGGAGAAATGCTTGGATTCTC
AGCTATGGCATGCTTGTGCTGGTGGAATGGTTCAAATGCCTTCAGTTAACACCAAAGTCTT
TTACTTTCCTCAAGGCCATTCCGAGCACGCTTGTGGCACTGTTGATTTCAGGAACTGTCC
TCGAATACAAGCTTATATACTCTGCAGAGTCGCCGCCGTTAAGTTCATGGCCGACCCTGA
AACCGACGAGGTTTTCGCCAAAATCAGGCTGATCCCAGTTAACACCAATGACCCTGATTT
TGAAGATGATGGAATAGGAAGCATTAATGGGAATGAAACACAAGAAAAACCGGCTTCCTTT
GCAAAGACATTGACTCAATCAGATGCTAACAATGGTGGGGGATTTTCAGTTCCAAGGTAC
TGTGCTGAAACGATATTTCCCAGATTGGATTACTCTGCTGATCCCCCGGTTCAGACCATTC
TGGCTAAGGATGTCCATGGGGAAACTTGGAAGTTTAGGCATATTTACAGGGGGACCCCAA
GGAGACATCTTTTGACTACGGGCTGGAGTACTTTTGTCAACCATAAGAAGCTTGTAGCTG
GTGACTCGATTGTGTTTCTTAGGGCGGAAAATGGGGATCTCTGCATCGGAATTAGGAGGG
CGAAGAGGGGGATTGGTGGAGGACCCGAGTCTTCAACCGGGTGGAATGCTACTGCTGG
AAATTGTATGATCCCGTATGGGGGGTTTTCAGCATTTCTGAGGGAAGATGAGGGCAAGCT
GATGAGAAATGGAAGCAGTAATGGGGTTAGCTCAAATGGTAATTTGATGGGGAAGAGGAA
GGTTAGGACTGAACAAGTTATTGAAGCTGCAACACTTGCTTCCAATGGCCAACAATTCGA
GGTCGTTTACTACCCAAGGGCGAGTACTCCTGAGTTCTGCGTGAAGGCCTCTTTAGTGAA
GGCTGCATTGCAGATCCGGTGGTGCTCAGGAATGAGGTTCAAAATGGCATTCGAAACAG
AGGATTCTTCTCGGATTAGTTGGTTTATGGGCACTATATCTTCAGTTCAAGTCGCAGATCC
CCTCCACTGGCCTGACTCACCTTGGAGGCTTCTTCAGGTTACATGGGACGAGCCAGATT
TGCTTCAGAATGTAAAACGAGTTAGTCCTTGGCTGGTGGAATTGGTTTCGAACATGCCCG
CGATTCATCTATCTCCCTTTTCACCACCAAGGAAAAAGTTGAGACTGCCGCAGCACTCGG
ATTTTCACCTAGATGGTCAACTTCCAATGCCGACATTTTCAGGCAACCTCCTTGGGGCCA
GCAACCCCTTTGGGTGTTTACCCAACACTGCTCCTGCTGGCATGCAGGGAGCCAGGCAT
GCTCATTACGGTCTATCTTTATCAGATCTCCACCTCAATAAACTGCAGTCAGGTCTCTTTCC
GGCTAGTTTCCCACCACTTGATTACGCTCCTGTACCTAATAGGACCTCCAATGGTGGCCC
AAACATTCGAAAGCCTAGCATGAGTGAGAACGTTTCTTGTGTGCTAACCATGTCCCATCC
CTCACAGAATACCAAGAAAGCTGATGGTGCAAAGACACCTCAGCTTGTACTTTTCGGTCG
GCCGATTCTTACTGAACAGCAAATCTCTCTCAGCTGCTCTGGTGATACAATCTCACCAGTT
CTTACTGGAAATAGTTCTTCAGAATATCTGGATAAGGCAGCAAACTTTTCCGATGGTTCTG
GATCTGCTCTTCATCAACAAGGCCTTCCTGAGCGAGCATCCTGCGAAGGTCTCCCGTGG
TACAAAAACAGTCGCCAAGAAACTGAACCCAATTTAGAGACCGGTCACTGTAAAGTTTTCA
TGGAATCAGAGGATGTCGGTCGTACACTCGACCTTTCTTTGCTGGGGTCTTATGATGAAC
TGCAGAGAAAGTTGGCAGACATGTTTGGTATAGAAAACTCCGAAACTCTGAGCCATTTACT
CTATCGGGATGCTACGGGTGCTGTCAAACAAATTGGAGAAGAACCATTCAGTGACTTTAT
GAAAACCGCGAGGAGATTAACGATTCTAACGGATTCAAGCAGCGACAACGTAGGAGAATA
TAGAGGAAGAAGCAAACCTTGA

>GrARF17-1
ATGCCGCCTTCACGGTCTAGTCCTCCGGAGCTACGTCATGTTGATCCAAGGATCTGGCGA
GCTTGTGCCGGCTCTTCCGTTCAGATCCCCACCGTTAATTCTAGGGTTTATTACTTCCCTC
AAGGCCACCTTGAACAAGTTTGCGGTTCCACTCCCAAACTGTCCTCTCTCGTACTGTCTA
GGCCTCTCATCAATTGCGTTATCTCAGACGTTCATTATCTTGCCGATCCGAGAACCGATGA
GGTCTTCGCTAAGCTTTTACTCACCCCTGTTGAAACTTCTAGGCTTCCTAATCAATTTCTAA
ATGTGAATGGTGAAGTTGAGGATTCTGATAGGAATAAGATCGTGTCGTTTGCTAAGGTTTT
AACACCCTCTGATGCCAACAACGGCGGTGGATTCTCCGTCCCGCGGTTTTGTGCCGATT
CTGTTTTCCCGCCGCTCGACTACAATGCTGATCCGCCAGTTCAGACTCTCTCCGTCACCG
ACGTTCGCGGCGGTGTTTGGGAGTTTCGTCACATTTATCGAGGGACGCCACGTAGGCAT
CTGCTCACTACGGGATGGAGCAAGTTCGTTAATCAAAAGAAGCTTATCGCCGGCGATTCT
GTTGTTTTCATGAGGGACTGTAATTGGAAAATGTTTATTGGAGTCCGACGAGCGATGAAG
GCGGGAGAAGGCGGAGGGGATTCTGGGAGGTGGAGAGAGCCGAGTGATGGAGGAGCA
ATGAAAGGGGAAGGAAGGGGGAGGATGACGGCGGAGGCAGTGGCTGAAGCGGCGGAG
AAGGCAGCGAGGGGGTTCCCGTTCGAGGTTGTGTATTATCCTCGGGCCGGTTGGACTGA
TTTCGTGGCAAGGGCGGTGTTGGTGGAGGCTGGAATCAGTACTTACTGGGCTGCGGGG
ACCAGAGTGAAGATGGCAGTTGAGACGGAGGACTCCTCGCGATTGGCTTGGTTTCAAGG
GACAGTTATGTCTGCTGCTGTACCGGATTCTGGCCCCTGGATTGGCTCGCCTTGGCGAAT
GCTTCAGGTTGCTTGGGATGAACCTGAAGTTCTCCAGAATGCAAGGAAAGTGAACCCAT
GGCAAGTTCAAATTTCTTCCTCCTCACCGCTTCATTCCTCGTTCCCCTCGGAAAAGAGGC
TGAAGTTTTCTCAGGATTCAGGGCTGGCTGATGCAGAGGGTGAAATCTTTCCTATATCAG
GGTTAACTAATTCAACAATGGGGTATTTGAATCCATCACTGTTGAATTACAATTCTTTTCCT
GCTGGCATGCAGGGAGCCAGGCAATATCATTTTCACTTGCAAAGTTTAACCAACGATATGA
GTGAGAATACCCCAATGATGTCCACTGATAACTTCTCTGGCAACTATGTGGTACCCAAGCC
AACTAGGATATCCACTGAGCTCAATATTGGCAGTTCACAGTCTGACAACTTGTCACCTGAT
AGTCAGAGCAGCATGGTTTCCTTTGGCACGGAACTTATCGAACATGGGGGTTGCAACTCA
AGCAAAGTAGGTGTTGGTTCGTTTCGATTGTTTGGCAAGATAATTGCTTTGAAAGAGCCTG
TCAGAAGCAGATTTGATGATGTTGGTTGTATGGATGATGTTGGAGGTAAAAGGCATGATGA
AGCTGTGAGTGAAAAGAACTCCTTAGATCTGTCATTGACTTATGGCTGTTCGAAGCTGCTT
GACAGGCTTGATGTCCAATGCCAACGAGCCTCAACTTTTGAAGGCTTCTCCTTGTGA
>GrARF17-4
ATGCCGCCTCCACGGCCGCCGGCTGCGGAGGTTCGCCACGTCGATTTAAGGATCTGGC
GAGCTTGCGCTGGCTCTTCCGTTCAAATCCCCACTGTTAACTCTATTGTTTACTACTTCCC
TCAAGGACACGTGGAACAATCTTGCGGTTCGACTCCGTTACTCTCTTCTCTCGTGCTCTC
TAGGCCTCTCATCCCTTGCGTTGTCTCCGACGTTCATTGCCTCGCCGATCCCAGAACTGA
TGAAGTCTTCATCAAACTCTTCCTCGTCCCTGTCGAGCCTTCTAGACTACCCAATCAATTT
CTAGACGTTAATGGTGAAGTTGAAGATCCTGATAAGATCGTGTCCTTCGCCAAGATTTTAA
CGCCGTCCGATGCCAACAATGGCGGCGGATTCTCCGTCCCCCGGTTTTGTGCTGACTCT
ATTTTCCCGCCTCTTGACTACAATGCTGACCCGCCTGTTCAGACTCTCACCGTCACCGAC
ATTCGCGGCGGGGTTTGGGAGTTCCGCCACATTTACAGAGGAACGCCGCGTCGGCATCT
GCTCACTACGGGATGGAACAAATTTGTTAATCAAAAGAAGCTCATCGCCGGCGATTCTGT
TGTCTTCATGAGGGACTGTAATGGGAAAATGTTTATTGGAGTCCGTCGGGCTTTGAAGAG
AGGAGAAGGCGGTGGGGATTCCAGGAGGTGGAGAGAGCCAACTGGTGGAGGAGCAAC
GAAAGGGGACGGAAGGGGGAGGATGACGGCGGAGGTGGTTGTGGAAGTGGCGGAGAG
GGCCGCGAAGGGGTTGCCATTCGAGGTTGTGTACTATCCTCGGCCCGGTTGGACAGATT
TCGTGGTGAGAGCGGAGTTGGTGGAGGCTGGACTCAATATATACTGGGCTGGTGGGACC
AGAGTGAAGATGGCAGTTGAAACAGAGGATTCCTCGAGAATGACGTGCTTTCAAGGGAC
GGTTATTTCTGGTGCTTTGTCGGATTCCGGCCCCTGGATTGGCTCTCCTTGGCGAATGCT
TCTGGTTGCATGGGATGAACCTGATCTCCAGAATGTAAGGAGAGTGAACCCATGGCAAGT
TGAAATTGCTACTTCCTTACCGCTTCAGTCTTCATTTCCCTTGGCGAAGAAGTCCAAGTTT
TCTCAGGAATCTGGGCTGGCTGATGCAGAAGGAGAAATAATGTTCCCTATGACAGGGTTA
ACCAATTCAACAATGAGGTACATGAATCCATCACTGTTGAATTACAATTCTTTTCCTGCTGG
CATGCAGGGAGCCAGGCAAAATCATTTTCACGTGCAAGGTTTAACCAACCATGTGAGTGA
GAATACCCCAATGATGTCCACTGATGCTTCATCTAGCAACTATTGGGTGCCCAAGTTAAAG
AGGATATCCACTGAGCTTAACATTGGCAGTTCACAGTCTGACAACTTGTCCCCAGATAGC
CAGAGCAGCATGGTATCCTTTGGCACAGAATTTACTGAAAATGCAGGCTGCAACTTGAGC
AAAGTAGGTGTTAATTCGTTTCAACTGTTTGGCAAGACGATTCATATGAAGGAGCCTGGTG
GAAGCATGTTTGGTAATGTTGGTAGCATGGAAGATGATAGTGGTAAAAGATATGATGAAGC
TGTGAGAGAAAAGAACTCGTTAGATCTTTCATCCACTAACGACTATTCAAAGCTGCATGAC
AGGATAGATGTCAAAAGTGAGAGTGCTTCAGCTTTCAAAGGCTTTTCTTTGTGA

>GrARF17-5
ATGGAGCTTGAAAAAAGCTTAGACCCAAATCTATGGCATGCATGTGCTGGATCCATGGTTC
AAATCCCACCAATAAACTCCAAAGTCTATTATTTCCCTCAAGGCCATGCCGAGCACTCACT
TAACTCGGTGGATTTCCCGTTTCCTCGTCAAATCCCACCCCTTGTTCTTTGCCGAGTTGA
CTCAGTCAAATTCATGGCCGATGGTGAAACCGATGAAGTATATGCCAAGATCATGCTTACG
CCATTACAGAACAATGAGCCTGATGTTGGAGACGATGATGGTGTTTCAAATGGCGGCTGT
AATAATATTGAAAAAAAGGCTGCTTCTTTTGCTAAGACGTTGACTCAATCCGATGCTAACAA
CGGCGGTGGGTTTTCCGTTCCGAGATACTGTGCTGAAACCATTTTTCCCGCGTTGGATTA
TAGTGCCGATCCTCCGGTTCAAACCGTCGTCGCCGTTGATGTTCATGGTCAGCTATGGAA
GTTCAGGCATATTTATAGGGGAACTCCGAGGAGACATTTGTTGACTACAGGGTGGAGTGC
TTTCGTCAACCGGAAAAAACTGGTTGCCGGCGACTCAGTCGTGTTCTTGAGAGCTGAGA
ACGGCGGAATTCGTGTCGGGATCAGGCGAGCCAAGCTTGGGATTGGGGATGGTGATTGT
TACGGAAGTTTGGGTTTTTGTGGGAATTCGAAAGGGAACGGAAAAGTGAAGCCGGAGGA
GGTTTTGGAGGCGGCGAAGGCGGCTTCCGGTGGCCGGAAGCCGTTCGAGGTTGCTTAT
TATCCGAGAACAAGCGGGCCGGAGTTTTGTGTTAAGGCATCTGCAGTGAATACAGCAATG
AAGATCCCTTGGTGTTGTGGTATGAGGTTCAAAATGGCTTTTGAGACTGAAGATTCTTCTA
GAATTAGTTGGTTCGTCGGAACCATATCGTCCGTTCAAGTTGCCGATCCGGTCCGGTGGC
CGGACTCGCCGTGGCGGCTTCTCGAGGTGACATGGGATGAACCGGATTTGCTGCAAAAC
GTTAACCGTGTGAATCCGTGGTCTGTTGAATTAGCATCGAACTTGCCCGCTATCCACCTCT
CTCCCTTTTTACGACCAATGAAGAAGTTGCGGCTCCCTCAACACCTCGATGGACTTTTAG
TGCCGTCTTTTCCAAGCAAGCCCCTTATCCCGAGCAGCCCGTTATATCGTTTATCTGATAA
TGCTCCTGTAGGGATACAGGGAGCCAGGCAAGTTCAATTCGGGTTACCTTTATCAGATCT
CCATCTTAAAAATAACCCGCATTCGGGACTGTGCCTGTCCGGTTTGCAGCGGTTTGATCT
ACACGATAGAATTCCCGATACATTCACAAACAGTCGCAATAATCTATCTTGCTTTTTAACAAT
GGGGAACTCTAATAAATCCAAAAACCCGAAAAAATGCCGGTTTTTACTCTTTGGTCAGCCG
ATACTTACTGAGCAGCAACTGTCTCAGAGCTCTTCAAGTGAAGCTGTCTCGGAAGTTGTT
AGTGGAAACAGTTGCAAAACAAAAGATTCATCCGACGGTTCAGGATCTGTTATCGGGAAC
CAATTCTCTCCGGAGAAGTCATCTACTGCTCAATTTTTGTGGGATCGGGACAATCGAGCC
ATAGAACCCTGCATGGATACCGGTCATTGCAAGGTATTCATGGAATCAGAGGATGTCGGA
CGAACTCTTGACCTCTCGGTTCTCGATTCTTATGAAGATTTATACAGGAGATTGGCTAACAT
GTTTGGAATAGACAGATCGAAAATGTCCGAACATGTGCTGTATCGAGACGCAACTGGTGC
CATCAAACGAACTGGAGATGAACCATTCAGTGCATTTACGAAGATCGCCAAAAGATTAACA
ATAAGAATGGATTCAAGCAACAATACTATTGAAAGGTCCCGGCTCACCGGAACCCGAACT
GCGGAAAATGGATTTCGAGGATCAAACAAGACAGGTCCTTTAAGCATAGTTGCATGA
>GrARF18-2
ATGGCTCATTTAGAAGGTAATCAAAGGGGTTCCTCGACTGCTCATGCGGTTTCAGGTTCG
GCTGGTGATGATCTATATCAAGAATTATGGAAGCTATGTGCAGGCCCCTTGGTGGAGGTTC
CTCGGGTTCACGAGAGAGTTTTTTACTTCCCTCAGGGTCACATGGAACAATTAGAAGCAT
CGACAAATCAGGAACTTAGCAATCAAACCCCACTGTTTAATCTTCCTTCTAAGATCCTATGT
CGTGTTCTTCACGTGGAGTTACTGGCAGAACAAGAGACAGATGAAGTTTATGCTCAGATC
ACTTTGCAGCCTGAAGATCAAAGTGAGCCTACAAGTCTTGATCCCTTCCCAACCGAGGCT
CCAAAGAGGAAAGTTCATTCCTTTAGTAAGATTCTAACGGCATCAGACACTAGCACTCATG
GAGGGTTCTCTGTTCTCCGAAAGCATGCAACTGAGTGCCTTCCTCCTTTGGACATGAATC
TAGCAACTCCAACTCAGGAGTTGGTTGCCAAAGATCTTCACGGGTATGAGTGGCGGTTCA
AGCATATATTTAGAGGGCAACCACGGAGACATTTACTTACAACAGGGTGGAGCACTTTTGT
GACTTCGAAGAGATTAGTTGCTGGAGATGCATTTGTGTTTCTTAGAGGTGATAATGGAGAA
CTAAGAGTTGGGGTTCGGCGGCTTGCTCGTCAACAGAGCACAATGCCTTCGTCTGTGATA
TCCAGCCAGAGCATGCATTTAGGAGTGCTTGCTACTGCTGCTCATGCTGTTACAACTCATA
CCCTCTTTGTTGTGTATTACAAACCAAGGACAAGCCAATTCATAATTGGAGTAAACAAGTAT
CTGGAGGCTATTAACAATGGATTCTCTGTTGGCATGCGTTTCAAGATGAGATTTGAGGGA
GAAGACTCTCCTGAAAGAAGGTTCACAGGTACCATAGTTGGGGTTGGAGATATTTCCCCA
CATTGGTCAGAATCTAAATGGCGGTCCTTGAAGATTCAATGGGATGAACCTGCAGCAATAC
AAAGGCCAGAGAGAGTTTCTCCTTGGGAAATAGAGCCGTTTGTAGCTTCTGCTTCTACAA
ATGCACAACCTACTATAATGTGCAAAAGACCAAGACCTGTCGATATTCCAGCTTCTGAAATT
ACTACCAGTTCAGCTGGTTCAGCCTTCTGGTGTCGTGAGTCAATCCAGTCTCATGAACTA
ACACCAGTGGGAAGCACACTTGAGGTCCAGAGCAGTGAAAACCAGGTTATGTGGCCTAT
GAGGCAGAAAGAAGCTGATAATTGTCTTATCAATGGTAATGGAGGTTACAAATCAAGGACT
CCACCTGAAAATGCCTGGCCACCTTCTCCTCTTGTGAATGTATCTTTGAACCTTTTTCCTG
ACTCAATGGAGAACAACTACAAAACAGGAGCATTGCAAACCGCTCTCACTGGTTATGCCA
AGGGCCTAATGCATGATCAAGTTGAAAAAAGAAAAACTGAGACTTTCACAGGTTGCCGGT

TGTTTGGGTTTAATTTGACAGATAGCACTAGTGTAGCTGCCCCCCCTGACAAGGAACAAA
CGAGCACAAGTATTGACTACAATGGTGTGAGAGGGCATGTCCCTGCTGCGTTTCATGTTG
AACAGAAACCAGAAACTTCAAAGGAGCAGAAGCAAGTTGCATCGGAGACATCAACCCAG
GAGATGCAAGCTAAGCAGGGTTCTGCAACTTCCATGAGAAGTCGTACCAAGGTACATATG
CAAGGGATTGCAGTTGGCCGTGCTATTGACTTAACTGCACTGAAAGGATATAATGATCTCA
TAAATGAGCTGGAGAAAATGTTTGAGATCAAGGGAGAGCTTTGTCGTAGTGGTCAGTGGT
CCATTGTTTTTACTGATGATGAGGGTGATATGATGCTCGTGGGTGATGATCCCTGGGTTGA
ATTTTGTAAGATGGTGAGAAAGATCTTCATATATTCAAGCGAGGAGGTGAAGAAGATTAGT
ACAAGATGCAAATTTCCAGCATCATCTATGGAGTGTGAAGGGACGGTTGTAAGCTTGGAC
TCAGAGCATAGGTCTGTTTGA
>GrARF18-3
CTGTATCCAGAACTATGGAAATTATGTGCAGGCCCTTTAGTGGAGATTCCTCATGTTCAAG
AAAGAGTCTTTTATTTCCCTCAGGGTCACATTGAACAATTAGAAGCATCAACTAATCAAGAA
CTTAACTATGAAGCCCCACTGTTTAATCTTTCTTCAAAGATTCTATGTCGTGTTCTTCATGT
CCAGTTACTGGCTGAACAAGAAACAGATGAGGTTTATGCACAAATCACTTTACAGCCTGAA
CCAGATCAAAGTGAACTGACGAGTCCGGATCCATTCCCGACCGAGGTTCCGGAGAGGGA
AGTTCATTCCTTTTGTAAGATTTTAACTGCGTCAGATACAAGTACTCATGGAGGGTTTTCCG
TTCTACGGAAACATGCCACCGAGTGCTTGCCTCCATTGGATATGAATCAAGCTACCCCGA
CACAGGAATTAACCGCGAAAGATCTTCACGGATATGAGTGGCGGTTTAAGCATATATTTAG
AGGACAACCTCGGAGACATTTACTTACGACGGGGTGGAGCACGTTTGTGACTTCGAAAA
GATTGGTTGCGGGAGATGCATTCGTGTTTCTAAGAGGTGATAACGGAGAATTAAGAGTTG
GGGTTCGCCGGCTCGGTAATCAACAAAGCACAATGCCTTCGTCGGTCATATCTAGCCAAA
ACATGCATTTAGGAGTGCTCGCTACTGCTGCTCATGCCGTTATGACTCAGACCCTCTTTGT
TGTGTACTACAAGCCGAGGACGAGCCAATTCATAATAGGAGTAAGTAAGTATCTCGAAGCT
ATTAACAACAGATCCTCCGTTGGTATGCGTTTCAAGATGAGATTCGAGGGCGAAGATTCC
CCTGAGAGAAGGTTCACTGGTACCATAGTCGGGGTTGGAGATGCTTCCCCACATTGGTC
AGAATCTAAATGGCGGTCCTTGAAGATTCAATGGGATGAACCTGCAATGATACAAAGGCC
AGAACGAGTTTCCCCTTGGGAAATAGAGCCATTTTCGGCTTCTGCTTCTATAAACCTCATA
CAACCAGCTGTAAAGAGCAAAAGACCACGACCGGTTTATATTCTAGCTTCTGATACGACTA
CAAATTCAACCGGTTCAGCCTTTTGGTGTCACGGGTCAACCAAATCACATGAACTAGCTC
GAGCAGGAAGCATAGCTGAAGTCCAAAGCAGTGAAAGCAGCCAAGTTGTTTGGCCTATG
AGACAGAATGAAGCCGATGCAGGTTATAACACGAGGGCTCGGCTTGAAAATGCTTGGCC
ACCTTCCTCTCTTGTGAACGTCTCTCTAAACTTTTTCCGCAATCCAGCGGATGCCTCTCCT
GTTGAATTGAGGACAAGCAATGACGTTATGCGCGACCAAGTTGAAAAAGGAAAGAAACTC
GAGATTTCTACAGGCTGCCGTTTGTTCGGGTTCAATTTGACAAATAGTAATAGTGCAGTCA
GTGGAACCGTTATAGCTCCGTCCCATATTGATGAAAATCCCGAAACTTTTCAGTCACCAAA
GCAGCAAAAGCAAAATGCATCAGAGACATCAACCAAGGAGATAAAGGCTAAGCACGGTAC
CACTTCTTCTATGAGAACTCGTACTAAGGTTCAAATGCAAGGGATTGCCGTCGGTCGTGC
TATTGACTTAACCGTATTAAAAGGATACGATGACCTCATAAATGAGCTAGAGAAAATGTTCG
ATATTGAGGGAGAGCTTCGTCACCGTACTAAATGGTCTGTCGTTTTCACTGACAATGAAG
GTGATATGATGCTTGTGGGCGACGATCCTTGGGTGGGATTTTGTAAGATGGTGAGAAAGA
TATTCATATATTCGGTCGACGAGGTGAAGAAGATTAACGGGAGATGCAAATTTCAAGCCTC
GTCTTTAGAAGGCGAAGGCACTGTTGTAAGCTTGGGTTTAGAGCATAGGTCCGGAACATG
A
>GrARF18-5
ATGTTTGTTTTAATTGAATTTGATTTTGGTTGGATAGGTTCGAGTGATGATGATCTATACGCA
GAGCTATGGAAGTTATGCGCAGGCCCGTTGGTGGAGATTCCTCGGAATCACGAGAGAGT
GTTTTACTTCCCTCAGGGTCACATGGAACAATTAGAAGCATCCACTAATCAGGAACTTAAT
AATCAAGCCCCACTGTTCAATCTTCCTTCTAAGATCCTATGTCGTGTTATTCATGTCCAGTT
ACTGGCAGAACTAGAGACTGATGACGTTTATGCTCAGATCACTTTGCAGCCTGAAGCAGA
TCAAAGTGAACCTACAAGTCCTGATCCTTGCCCAGCTGAGGCTCCAAAGAGGACAGTTAA
TTCGTTTTGTAAGATTTTAACAGCATCAGATACGAGCACTCATGGAGGGTTCTCCGTTCTT
CGAAAGCATGCTACTGAGTGCCTGCCTCCTTTGGATATGAATCAGGCAACCCCTACACAG
GAGTTGGCTGCTAAAGATCTTCATGGATATGAATGGCGGTTCAAGCATATATTTAGAGGAC
AACCACGGAGACATTTGCTTACAACAGGGTGGAGCACCTTTGTCACTTCCAAGAGATTGG
TTGCGGGAGATGCATTTGTGTTTCTCAGAGGTGATAATGGAGAACTTAGAGTTGGGGCTC
GACGGCTTGCTCGCCAACAGACCACCATGCCTTCATCTGTGATTTCCAGCCAGAGCATG
CATTTAGGAGTGCTTGCTACTGCTGCTCATGCTGTTACAACGCAGACCCTCTTTGTTGTGT
ACTACAAGCCAAGGACGAGCCAATTCATAATAGGTGTTAATAAGTATTTGGAGGCCATTAA
AAATGGATTCTCAGTTGGTATGCGTTTTAAGATGAGATTTGAGGGAGAAGACACTCCTGAT

AGAAGGTTCACGGGTACCATAGTTGGGGTAGGAGATTTTTCCCCACATTGGTCGGAATCT
AAATGGCGAACCTTGAAGATTCAATGGGATGAACCTGCAACAATACAAAGGCCAGAGAGA
GTTTCACCATGGGAAATAGAGCCATTTGCGCCTTCTGCTTCCATAAATCTTGTTCAACCAG
CTGTAAAGAACAAAAGGCCACGGCCTGTTGATATTCCAGTTTCTGAAATTACTACAAATTC
AGCTGGTTCAACCTTCTGGGGTCGTGGTTCAACCCAATCTCATGAACTAACTCAAGTAGG
AAGCACACCTGAAATCCAAAGCAGTGAAAGCCAAGTTATGTGGGGTATGAGGCAGAAAG
AAGCTGATTACAGTCGAGGTTATAATTCGAACGCCTGGCCACATTCTCCTCTTGTGAATGT
CTCTTTAAACCTTTTTCCTAATTCAGTGAGTGACAAAAACAGAACTGAAAAACCACAAACC
ACTCTCACTGGTTATGCCTTGCCTTCTCTATCGAGGCCAAGTAAGGGCCTGATGCATGAC
CAAGTTGAGAAAGGGAAAAAATCTGAGACTTCCACAGGTTGCCGGTTGTTTGGGTTTAAT
TTGACAGACACCATTAGTGCAGTTATCCCCACTGACAAGGAACAAACGAACACAACGGTT
GATCACAATGGTGTTTGGGGGTCTCTTGCAGCTGCGTCTCATATTGATCAGAATCCAGAA
ACAGCTAAACAGAAGCATGTTGCAGCAGAGGCCTCGTCCAAGGAGATGCAGGCTAAGCA
GGGTGCATCCACCACTTCAACCAGAAGTCGTACTAAGGTACAAATGCAAGGGATTGCGGT
TGGTCGTGCTGTGGACTTAACTGTGTTGAAAGGATATGATGATCTCATTAATGAAGTGGAG
AAAATGTTTGATATCAAGGGAGAGCTTCGCCCCAGTGGTAAATGGTCCGTTGTTTTCACT
GATGATGAGGGTGATATGATGCTTGTTGGCGATGATCCATGGATGGATTTTTGCAAGATGG
TGAGAAAGATCTTCATATATTCAAGCGAAGAGGTGAAGAAGATTAGTCCAAGATGCAAATT
TGCAGTGTCGTCTTTGGAGGGTGAAGGGACTGCTGTAACCATTGATTCAGAGCATAAATC
TGAAACATGA
>GrARF19-2
ATGGGGGAACGCGAGTTGACAGATGGTTGCTTGAGTGATGATGCAGGTGAAGCGCCGG
AGAGAAGGCACATCAACCCGGAGCTATGGCAGGCGTGTGCTGGGCCGCTGGTGAACCT
GCCGGCTGCTGGGACCCATGTTGTCTACTTTCCTCAAGGTCACAGTGAACAGGTTGCAG
CATCTATGAAGAGAGATGTGGATGCTCAAATTCTGAACTACCCAAATCTTCCTTCTAAGCTA
TTATGTCTCCTTCATAATGTCACCTTGCATGCAGACCCAGAGACAGATGAAGTCTATGCTC
AGATGACCCTCCAGCCTGTTTCTTCTTTTGATAAGGAGGCGTTACTGAGATCAGATCTTTC
TCTGAAGTCAAATAAGCCACAACCGGAATTCTTCTGTAAGACGTTGACAGCAAGTGATAC
GAGCACTCATGGAGGTTTTTCTGTTCCTCGACGTGCCGCCGAAAAGATTTTCCCTCCTCT
TGATTTCTCGATGCAAAAACCTGCTCAAGAACTTGAGGCCAGGGATCTGCATGAAAATGT
CTGGAAGTTCCGCCATATCTATCGTGGAAAACCACAGCGCCACTTGCTTACAACAGGATG
GAGTCTATTTGTTAGTGGAAAGAGACTTTTTGCTGGTGACTCAGTTTTATTCATTAGAGATG
AAACACAGCAGCTTCTCTTGGGTATAAGGCGTGCTAACAGGCAACCTGCCAATCTATCAT
CATCAGTACTGTCTAGTGATAGCATGCATATTGGCATCCTCGCTGCAGCTGCTCATGCAGC
AGCAAATAATAGCCCCTTTACCGTGTTTTATAACCCAAGGGCTAGCCTATCTGAATTTGTTA
TTCCTTTAGCCAAGTACTATAAAGCTGTGTACAACCATCAAATATCACCTGGCATGCGCTTT
CGAATGATGTTTGAAACTGAGGAGTCAGGAACAAGAAGGTATATGGGTACAATTACAGGA
ATCAGCGATATTGACCCTGTAAGATGGAAAAACTCACAATGGCATAATTTGCAGGTTGGCT
GGGACGAGTCAACTGCTGGGGAAAGACGTAATCGAGTATCCATTTGGGAAATCGAACCA
GTTACAGCTCCATTTTTCATCTGTCCATCTCCATTGTTCAGATCTAAGCGCCCTAGGCAAC
CTGGAATGCCGGCTGATGAATACTCCGACTTAGATAATCTATTCAAGAGGCCAATGCCTTG
GCTTGGTGATGATATATGCCTGAAGGATTCCGATGCCCATCCAGGGCTTAGCTTGGTCCA
GTGGATGAACATGCAGCAAAATCCTCTGCTGGCAAACTCTATGCAGCCAAATTTCATGCA
GTCTCTGGCTGGGTCTACTATGCAAAACTTTGACGGAGCAGATCTTTCCCATCAAATGGG
CCTTTCAGCACCACAAATGCCTCAACCCAACAACTTACAGTTCAATGCTCATAGGCTACCT
CAGAAAGTGCAGCAACTTGATCAAGTTCCAAAGCTACCATCTACAATGAACTCACTGGGA
TCCATTATTCAGCCGCAACAGCTGAATGACATGACTCAGCAGTCGAGGCAAAATTTGGTT
GCTCAGCCTCTACCCTCTAGTCAAGTTTTGCAGCCTCAAGCCCTTGTCCAAAGTAACAATA
TCCTTCAGCAGCAGCAAACATCTAATCCAACTCATCAACTCCCTCTAAGTCTTCCTCAAAA
CCTGCAGCAGCATCTTGTGGGCCCAAATCATCTGCAAAACCTAATGCATTCCCAGCTGCC
TGATCCACTCAACCAGCATTTACAAGTGCCTGACAACCAGGTCCAGTTTCAACTGATGCA
GAAACTTCAGCAGCAACAACAGTTGCTTTTGGCACAGCAATCTGCACTTCAGCAGCCTG
GTCAACTTGCCCAACCCCAAGATCAACAAAGGCAGCTGTTAGATGCGTCTCAGAGCTTCT
CTAGTTCTGTGACAGCTAGCCAAGTGTTAGAGATGCCTCAAAACATACCTACCTTGCTACC
TCAATCTAATGTTGCCCCACAGCAGATGCCTAAAAATAACAGCCAGGCAAATGTTTGGTTC
TCTCAGCCGCCTCTGCAGTCAAAAGTTCAGCAACAACAAACTGGAATGCTACCTGAAGTT
CCTGGTCTTGTAGGTCCCTTCCAAACTACAGCAACAAATCAGTTCTCCACAGCTGTTAGTA
GTGTAATGATGTCTGCTGCTGTAGCCGCACCTTCTGTGATTACTGATGATAATCCATCATG
CTCCACTTCGCCATCTACAAACTGTCCAAGTGTTCTTCAACCAATGATAGACAGCAGAGTC
CACAGGAGTACTGGGTTAGGAGATGACATCAGTCAGTCTACTGCCACAGTATTGAATCCT

AATGCCGTGGAGACGATGTCAACTAAGGCTAATATGGTTAAAGAACAGCAGCAAAAGTCT
GTTAAACCCTTGTTGAATATCTCCAAGAGTCAAAACCAAGGCTCTTTTGCCCCGCAAAACT
GTATCAATGGTGCTACAGCACATGCAGATTGTTTGGACACATCATCTTCTACAACTTCAGT
TTGCCTTTCTCAAAGTGATGTTCATTTGCACCAAAACACACCGTCTTACAACCCTCAAACA
ATGTTGTTGAGAGATACAAGTCAAGACGGAGAAGTTCAGGCATATCCAAGGAATAGTGTTT
CATACGGCAATAATATGGATAGCCAAATTGAGATGTCCATGAATTCTGACACTTTGTCTGCG
AAAGGCATGATGGGACTGGGGAAGGATTTTTCAAATCACCTCTCTTCGGGAGGGGTACTT
GCCAGCTATGAAAACCCTAAAGATACTCAGCAAGAACTTTCTTCGTCAATGGTTTCCCAGT
CATATAGAGTTCCAGATATGGCGTTTAACTCCATTGATCCCACTATAAACCATAGCAGCTTC
ATAAACCGCAGTGGATGGACCCCACCATCACAATTTCAGAGATTGCGAACATATACCAAG
GTATACAAACGTGGAGCTGTTGGAAGATCAATAGATATAACTCGTTATTCAGGTTATGATGA
GCTCAAACAAGATTTGGCTCGTAGGTTTGGAATTGAGGGGCAGCTGGAAGACCGAGGGA
GAGTAGGCTGGAAACTAGTCTATGTGGATCACGAGAATGATGTTCTGCTAGTAGGAGATG
ACCCATGGGAGGAGTTTATCAACTGTGTCAGATGCATCAAAATACTCTCCCCTCAGGAAG
TCCAGCAGATGAGCGTGGATGGAGAGTTTGGGAACTCTGTCCTGCCTAATCAAGCCTGTA
GCAGCTCTGGCAATGGGAATGCATAA
>GrARF19-3
ATGAAGGCTCCACCAACTGGATTTTTGGCAAATCCTGCTGAAGGAGATAGGAAGAGTATC
AATTCAGAATTATGGCATGCTTGTGCTGGACCACTGGTTTCTTTGCCACCAATTGGAAGTC
TGGTGGTTTATTTTCCTCAAGGCCACAGCGAACAAGTTGCAGCATCAATGCAGAAGGAGA
CTGATTTCATACCAAGTTACCCTAACCTTCCTTCCAAGTTGATTTGTGTCCTCCATAATGTT
ACATTGCATGCTGATCCAGAAACTGATGAGGTCTATGCCCAGATGACTCTTCAACCTGTAA
ACAAAGTAGTGAAAGATGGGAAGAATCTTTATAGTACTTCTGCACAGTATGACAAGGAAGC
ATTACTGGCATCTGATATGGGCCTCAAGCACAGCAGGCAACCTGCTGAGTTCTTTTGCAA
GACTCTTACAGCTAGTGATACTAGCACTCACGGTGGATTTTCAGTCCCTCGTCGAGCAGC
TGAGAAGATCTTCCCTCCTCTGGATTTTTCCATGCAACCACCAGCTCAAGAGCTTGTAGC
AAGAGATTTGCATGAAAATGCATGGACTTTTAGACATATTTATCGAGGTCAACCAAAGAGG
CACCTTCTGACTACTGGTTGGAGCGTCTTTGTTAGCACAAAAAGACTCTTTGCTGGTGAT
TCTGTTCTTTTCATAAGAGATGTGAAGTCACAGCTTCTCTTGGGTCTAAGGCGCGCTAATA
GACAACAGCCGGCTCTCTCGTCATCAGTGATTTCTAGTGATAGCATGCATATAGGGATCCT
TGCTGCTGCAGCCCATGCTGCTGCAAATTTCAGCCCATTTACTATATTCTACAATCCAAGG
GCAAGCCCCTCTGAGTTTGTGGTACCTTTAGCGAAATATTACAAAGCCATGTACACCCAAG
TTTCTCTTGGCATGCGGTTTAGAATGATGTTTGAGACTGAGGAGTCTGGAGTACGAAGAT
ACATGGGTACAGTTACTGGTATCAGCGACCTGGATCCTGTGCGATGGAAAAACTCACAAT
GGCGCAATCTTCAGGTTGGTTGGGATGAATCTACAGCTGGAGAACGGCCCAGGCGAGTT
TCAATTTGGGAAATTGAGCCTGTTGTATCTCCTTTCTTCATATGTCCACCTCCTTTTTTCAG
ACCCAGGTTTCCAAAGCAACCAGGGATGCCAGATGATGATTCTGATGTCGAGAACGCTTT
CAAAAGAGCTATGCCTTGGCTTGGAGATGATTTTGGTATGAGAGGTACCCCTAGTTCAATC
TTCCCTGGTTTGAGTTTAGTTCAGTGGATGAATATGCAACAAAGTAATCAGTTTCCAGCTG
CTCAATCAGGATTCTTTCCATCAACGGTTTCTTCGAATCCACTGCATAATAACCTTAGCATT
GATGATCCTTCCAAATTATTGAATTTTCAAGCTCCTGTATTACCTGCACCGAATATGCAATTT
AATAAAGCTAACCCAAACCAAGTCAACCAGTTGCCTCAGGCACCTACGACTTGGCCCCAG
CAGATATTGCAGACTCCGCTAAATCAACATCAGCAGCAGCAACCCCAACAGCAATTGCAG
CAACAACAGCCACAACAACCAAAGCAGCAGCCACAGCCACAGTCACATCTTCTTCATCAA
CAGCAGCCTCAGCCACAGCCATCTCCAAAGCAACAGCAACACATCCACCAGGAACAAAG
ACAACAGCCACAACAGCAGCAGCCGCTGCAACAACAACCAACCCTACCGCCTCAAGTAA
TTAATGGCATTGTTGCTCCTAACCAGATCTCAAATCAAAATTTGCATCAGCCAGCTGTTTAC
TCTCACTTGCAGCAGCAACAATTGTTGACAAGCAATAGCCTGTCTACCCAAACTACCCTCT
CCGCTCATATGACTTCATATCCTTTGACGTCATTACCACAAGATACACGGGTTCAGCAGCA
GATGGAACAGCAACCTAACCTCATGCAGAGGCAGCAGCAACAGACACAACTGCAGCAAA
GCCTGTCCCAGAGGACACAGCAGCAGCCGCAGATTCTGCAACTATCACAGCAGGGCCTC
TCGGAGCAACTGCAATTACAACTTCTCCAAAAATTGCAGCAGCAGCAGCAGCAGCAGTC
GGCTCAACAATTACTCTCCGCAGCTGGGTCTCTGCTGCAGCCTCCAATGTTGCAGCAAC
AGCAAACTCATCAACAGAACCAACCATTGCAGCAGTTGCCTCTTTCTCAGAGCCAGGTGC
AACCACTGGGTGGCAATGGCTTCTCAACATCAATGTTTGTGCAACCTCAACAACTTTCAG
TGCATCAATCCCAAAGTCAGAACAAACAACTTATGGCAATGAGAAGCAATTCTGGTCTTAT
TGATGGAGATGCTCCACCGTGTTCGACCTTATCTTCTACCAATAATTGTCAGGTTTCCCCA
TCAAACCTTATAAACAGAAGTCACCATGTACCATCCATATTGATGACAGATCCAGTTGTTGA
GCCTCCAAGTACACTAGCTCAAGAGCTCCTGAGCAAGCCTGATATTCAAATCAAACATGA
GCCGCCCACCTCTAGAGGACTAGACCAATCAAAGTACAAAAGTTCTGTAACAGACCAATT

AGAAGCATCCTCTTCTGGAACATCATATTGCTTGGACGCAGGCACCCTCCAGCATAATGC
CTCCCTTGCCTTTCTGGAAGGTGATGTCCAATCACATTCTCGGAACAATCTTCCTTTTACA
GCCAATATTGATGGATTGGCACCTGAGACTTTGTTAACAAGGGAATATGACTCTCAAAAGG
ATCTTCAAAACATGCTTTCTAATTATGGTGGTAACCCTAGAGATATTGACACTGAGTTGTCT
ACTGCGGCAATAAGCTGTCAGTCATTTGGTGTGCCAAATATACCTTTCAAGACAGGATGCT
CAAATGATGTTGCCATAAATGAGACAGGAGTTTTAAATGGCGGATTGTGGACCAACCAAAC
TCAACGCATGCGAACATATACAAAGGTGCAAAAGCGTGGTTCTGTGGGAAGATCAATTGA
TGTGACCCGCTACAAAGGGTATGATGAACTCCGGCATGATCTAGCCCGCATGTTCGGTAT
CGAAGGGCAGCTGGAGGATCCACAAAGTTCTGACTGGAAATTAGTTTATGTGGATCATGA
AAATGACATATTACTTGTTGGTGACGATCCTTGGGAAGAATTTGTAAGTTGTGTTCAAAGC
ATAAAGATACTGTCGTCAGCAGAAGTACAGCAGATGAGCTTGGATGGTAATCTTGGAAATG
TGTCAGTTCCCAATCAAGCTTGCAGTGGGACTGAAAACGGAAATGCATGGAGAGGACATT
ATGATGATACCTCAGCAGTCTCATTTAACAGATGA
>GrARF23-1
ATGATAACAGTCATGGATTCTCGGAAAGAAGTTGTGAAAAATTCAGAAAAATGCTTAGATC
CTCAGCTATGGCATGCTTGTGCTGGTGGTATGGTACAAATGCCATCAGTGAACTCTAAGG
TGTTCTATTTCCCTCAGGGTCATGCTGAGCATGCCAATGGGAATGTAGATTTTGGGAATCT
CCCGATTCCGTCACTCGTCTTGTGTCGGGTTTCCGCCGTTAGGTTTATGGCGGATCCCGA
AACCGATGAGGTTTATGCTAAAATCATGTTGGTTCCTTTGAGAGAGAATAGTTTTGGGGTT
GAAGATGATGGTTTTGATGGGAATGTTGGGGTGGAGAACCCTGAAAAATCTGCTTCCTTT
GCTAAGACATTGACTCAGTCCGATGCTAACAACGGTGGGGGGTTCTCGGTTCCGCGGTA
TTGCGCGGAGACTATATTTCCTAGGTTGGATTATAATGCTGAACCCCCTGTTCAGACTATTC
TTGCTAAGGATGTTCATGGTGAGGTTTGGAAATTTAGGCATATATATAGGGGGACGCCTCG
TCGACATCTTTTGACGACGGGGTGGAGTAATTTCGTGAACCACAAGAAGCTTGTGGCTG
GTGATTCGATTGTGTTTCTTAGAGCTGATAATGGAGATCTTTGTGTGGGGATTCGTAGGGC
GAAAAGAGGACTTGGGGGTGGACATGAATTTCCGGGATGGAACTCTGCGAGTGGAACCT
CTGGTTCACAAATTGGGAGTTATTCTCCGTTTTTGAGGGAGGGGGAGAGTAAATTGATGA
GGAAGGATTGTAGTGGGGATCCAAGGGGAAGAGTAAGGGCTGACTCCGTGATTGAAGCT
GCGAGTCGTGCAGCTAGTGGGCAGCCCTTTGAGGTCGTTTACTATCCACGAACTAGCAC
TCCTGAGTTCTGCGTGAAGGCCTCATCCGTTAGAGCTGCAATGCAAATCCAGTGGTATCC
TGGGATGAGGTTCAAAATGCCTTTCGAAACTGAGGACTCTTCACGGATTAGCTGGTTCAT
GGGAACGATATCTACTGCTAAGGTTGTCGATCCCATCCGTTGGCCTAATTCTCCATGGCG
ACTACTGCAGGTAGCATGGGATGAGCCAGATTTACTCCACAATGTGAAGCGTGTTAGCCC
ATGGTTGGTTGAATTGGTCACAAACATACCAGCTATTAATCTTAATCCTTTCTCGCCACCAA
GGAAAAAGATGCGGCTTCCACAACACCCAGACTTTTCCTTTCTTAACCAAATTCCAATGCC
ATCATTTTCCGGGAACACTTTCAGATCCAGCAGCCCCATGCGTTGCATTACGGACAACATT
CCTGGAGGCATACAGGGAGCCAGGCATGAACCGTTCGGATTATCTTCATCAGATCTCCGC
TCCAACAAGCTGCATTCAGGTCTGTTTCCATCTGGTTTTCATCAACTTGATCGTACTGCCC
CACCTATTAGACTTTCCGGCGACAACTTTTGTAGCGACCACCCGAACAATACAAATATATCT
TCCTTGTTGACAATAGGAAATCCGACCCAGAGTTTGAAACAAAGCAATGATAGCAAGACA
CCCCATATTGTATTGTTCGGTCAACTCATTTTCTGTGAGCAGCGGGCTTCTCAGAGCTGCT
CAGGGGATACGGTTGGAAACAGTTCATCAGATGGGAATACAGAGAAGACTGCTATTTCCT
CTGATGGAACTGGATCTGTGTTACATCAAAATGTTCGGGAAAATTCTTCGGATGAAGGGTT
TCCTTGGTGCAAAGAGCATCAAAAAACTGATCTAGGATTGGAGACTGGTCATTGCAAAGT
GTTTATGGAATCAGAGAATGTAGGTAGAACCCTTGATCTTTCAGTTCTCGGATCATATGAA
GAGCTGTATGGGAAGCTGGCCAACATGTTTGGCATAGAAAGTTCAGAGATGCTGAGCAGT
GTGCTCTACCGCGATGCTGCCGGTTCAGTTAAACACACTGGAGATGAGCCCTTCAGCGA
GTTTATGAAGACAGCAAGGAGGCTAACGATTCTCATGGATTCAAGCAGTGACAACTTAGA
AAGATAG
>GrARF23-2
ATGATAACAGTAATGGATTGTAGGAAAGAAGTGGTTAAGACATCAGAAAAATGCTTAGATC
CTCAACTATGGCATGCTTGTGCTGGTAATATGGTGCAAATGCCAGCGGTGAACTCCAAGG
TGTTCTATTTCCCTCAAGGTCATGCTGAACATGCCAATAGGAATGTAGATTTTGGGAGTCT
TTTAATTCCTTCACTCATCCTATGTAGAATTTCGGCCATTAAGTTTATGGCAGATCCTGAAA
CTGATGAGGTTTATGCTAAAATTATGCTGGTTCCTTTGAGAGAGAATGATTTTGGGTATGAA
GATGGTTTTGATGGAAATATTGGGATGGAGAATCTTGAAAAACCACCTTCTTTTGCCAAGA
CATTGACTCAATCCGATGCTAATAATGGTGGGGGTTTCTCAGTTCCGCGGTATTGTGCAGA
GACTATATTTCCTAGGTTGGATTATAGTGCTGAACCCCCTGTTCAGACCATTCATGCAAAG
GATGTACATGGTAATGTTTGGAAATTTAGGCATATATATAGGGGAACACCTCGCCGGCATCT
TTTGACGACGGGGTGGAGTAATTTTGTGAATCATAAGAAGCTTGTGGCTGGTGATTCGAT

TGTCTTTCTTCGAGCAGAGAATGGAGATCTTTGTGTGGGAATCCGTAGGGTGAAGAGAG
GTATTGGAAGTGGACATGAGTATCCTTCTAGTTGGAATTTGGGGGATGGAAGCTCCGGTT
ATTCTCCACTCTTGAGGGAGGGGGAGAGTAAATCGATGAGGAATGATTCGAATGGGGATC
TGAGGGGAAGAATAAGGCCCGAGAATGTGATTGAAGCTGTGACTCGTGCTGCCAATGGG
CAACCCTTTGAGGTTGTTTACTATCCACGAGCAAGCACTCCTGAGTTCTGTGTGAAGGCC
TCATCAGTTAGAACTGCAACGCAAATCCACTGGTATCCTGGGATGAGGTTCAAAATGGCT
TTTGAAACCGAGGACTCCTCACGGATTAGCTGGTTTATGGGAACAATATCTATCGCTCAGG
TTGTTGATCCCATCCGTTGGCCTAATTCTCCATGGCGCCTTCTTCAGGTGGCATGGGATG
AGCCAGATTTACTACACGATGTGAAGCGTGTTAGCCCATGGTTGGTTGAATTGGTGACAA
ACATACCAGCCATCCATCTTAATCCTTTCTCGCCCCCGAGGAAAAGGATGCGGCTTCCGC
AACACCCTGATTTTTCTTTACTTGGCCAAATTCCAATGCCATCTTTTTCTGACAGCACTTTC
AGGTCCAGCAGCCCCGCATGTTGCATTACAAACAACATTCCTGGAGGCATACAGGGAGC
CAGGCATGCACCGTTTGGATTATCTTCATCAGATCTCCGTTCCAGCAAGCTGCAGTCGGG
CCTCTTTCCACATGGTTTTAATCAGCTTGATCATACTATCCCACCTATGGGACACTCCAGT
GACTATGGGAACAATAGGAATATTTCTTCCTCGCCCGCAATGGGAAATCTTACCCAAAGTT
TAAAAGAAAGCAATGAAATAAAGACGCCCCATATCTTATTGTTTGGTCAACTCATTTTCTGT
GAGCAGCAGGTTTCTCAGAGCTGCTCAGATACAATTGGAAACAGTTCATCCAACGGGAAT
ACAGAGAAGACTATGATTTCTTCCGATGCCTCTGGATCTGCATTACATCAAAATGCTCGTG
AAAATTCTTCAGATGAAGGGTCTCCTTGGTACAAAGAGCTCCCAAAAACCAACATGGGGT
TGGAGACTGGTCACTGCCAAGTCTTCACGGAATCAGAGAATGTGGGAAGAACCCTAGAT
CTTTCAGTTCTCAGATCATATGAAGAGCTGCACGGCAATCTAGCCAACATGTTTGGCGTAA
AAAGTTCAGATATGCTGAGCAATGTGTTCTACCACGATGCTGCTGCTTCTGTTAAGCACAC
AGGAGATGAGCCCTTCAGTGAGTTTTTGAAGACAGCAAGGAGGCTGACCGTTCTTACAG
ATTCAGGCAGTGACAACATGGGAAGATAG
>GrARF24-2
ATGGACGGTGAACGGAATGGTTTGAAGGCTAAGGTCCCATTTCACCCCAGAAGACGTATC
AACGTCTATGGCTTGCCGTTGCTTCCTCCGAACAATCAAGGAGAAAAAGATGATCTACAT
GTTGAACTATGGCATGCATGCGCTGGTCCTTCAGTTTATGTGCCGCGCGCTGGGGAAAA
GGTTCTGTATTTCCCTCAAGGTCACATAGAACAGGTGGATGCATGTATGAATCAAGATGGG
ATAATGGAAATGCCTATCTACAATTTGCCTTCTAAGATCCTTTGCAGGGTGATGCATGTTCA
GCTTAAGGTTGAACCTGGCACAGATGAGGTCTTTGCACAAATAACACTGATTCCAGAAGC
AGAGCAAGATGAGGAAAGTTTGGAGCATAGAAATTATCAACCTTTACCCCAGAAAGCCTAT
CCAGTGTTCTTTAGTAAGAAACTCACTCCATCAGATACAAGCACACATGGCGGATTCTCTA
TCCCAAAGCGACATGTTGATGATGGGTGTCTCCCACCCCTGGACATGTCTCAGCAAACC
CCACAACAGGAATTGGTCGCAATAGACTTGCACGGTTTTGAATGGCGCTTTCGACATATTT
ATCGAGGTCAGCCAAAAAGGCACTTGCTTACAAGCGGCTGGAGTACATTTCTGACTTCAA
AGAAGCTTCTTGCTGGGGATGAATTTATCTTTCTTAGAGGGGAAAAAGGAGAGCTCCGTC
TCGGAATTCGGCGAGCAACGACTGTTCTGAAATATACATCAACATCCATCATATCTGGTCAT
AGCATGCGTCATGGCATACTAGCAAGTGCTTTTCATGCGTTCTCTACTAGAAGCATGTTTA
ATGTCAACTACCGTCCTTGGTCTACTTCTTCTGAATTTATCATCTCATTAGATCGGTATATGA
AGTCGGCTCAAATTGACTATTGCATCGGGACAAGATTTAGGATGCGATTAGAAGGTGAAG
AATGTGCGGAACAAAGGCCTTCTGGCACTATCATAAGCATTGAAGATGTGGATCATACTAG
GTGGCCTAATTCTGAATGGAGATGTCTGAAGGTGAAATGGTATCCCACAGCAGGTGAAAA
TTTTCATCCTGAAAGAGTTTGTCCTTGGAACATTGAACCAACGGAATTCAGAATCAAGAAG
AGACCCTCCATTCTACATAACCAAAAGAAGGCTCGTACTGATGATGTATCATCCCCTGGGT
TTTCTACCCTGCTAATGGATGGCATGTGGAGTGGTTCCATTAAATACGAATCTCAAAGTAG
CTCAGGGGTCTTGCAAGGTCAAGAAGATAGTGACACAGATGGTAATCAACCCGATGCTCT
AAGACAACCATTACCACATTGCCTCCCACTAAATCACAGTTGGGACTCAATGCAACAGCC
AATACAGAACCAACGAGAGATTGGTGCAGCACCCTTTTCTGGTGGACAAGTAGAAAGTTT
GGGTCTTCATAATAGTTGGTCCACAACATTCTCCTCCTCAAATGGAGTACATGAAGATGCT
ATTGCTAGCAGAAAAATTTCAGTTCCAAATGTTAATTCTCAGGAAGGGAGTATTTCAGAAC
CAAGGAATGAAAATGAAACATCATGGTGCGAACCAAATGAGGGACATGCTTGCATGCTTT
TTGGAGTTAATTTAGTTAACGGTCCACTGGAGCCCCCTTCACCCCAACTTGTCACTTCTAG
CGAGCTCGAAAGTCATTGTTCTATTCCTCCAACTTCTCAGTCAACTGTTTCGAAACCTTCT
AAGGGTACATCTAGCAAGCAGTGTGACAACTGCTGTTCTGCTAGCAATTGGAGTTGCACC
AAGGTACTCAAGCATGGGACTGCTCTTGGAAGATCAGTTGATATCACTAGATTTGACGGAT
ATAAAGACCTCATCTCTGAGCTTGATCGCATGTTTGATTTTAATGGACGATTGATTGATGGA
AGCAGCGGGTGGCATGTAACCTTTACTGATGATGAAGGGGAGATGAGGATGATTGGAGAT
CATTACCCATGGCAGTTAAGTGCAAAACCAAACAAAAATTCCATCTAA
>GrARF24-4

ATGGATGGTGAAGGGCATGGTTCCAAAGCTAAGTTCCCTTTTCACTCCACTAGAGGAGAA
AACAATGATCTCTATACTAAACTATGGCATGCATGCGCGGGTCCTTCTGTTTATGTTCCTCG
CTCTGGAGATAAAGTCTTGTACTTCCCTCAAGGTCATATGGAACAGGTTGAGGCATACATG
AGTGAAGATGGCACTATGGAAATGCCCATCTACAATTTACCTTGGAAGATCCTTTGCAGGG
TTTTGCATGTTGAGCTTAAGGTTGAACCTGACACAGATGAGATCTTTGCAGAAATTATTTT
GCTTCCAGAGGCTGAGCAAGATGAGCAAAGAATGGAGCATAGATATTATCGAGCGTCGCC
TCGGGAAAATTATTCTCGTTACTTTAGTAAGAAGCTAACTCCATCGGATATAAAGACACACG
GTGGATTCTCTATCCCAAAGCGGCATGCCAATGATGGGTGTCTTCCGCTCTTGGACATGT
CTCAGGAAATCCCCCAGCAGGAACTGCTCGCAACTGACTTGCATGGTCATCCATGGTACT
TTCGACATGTTTTTCGTGGCTATCCAAAAAGAAATTTGCTTACCACCGGTTGGAGTACCTT
TGTCACCTCGAAGAAGCTTGCTGCTGGGGATTCATTTATCTTTCTAAGAGGGGAAAATGG
AGAGTTCGGTGTTGGAGTTCGCCGATCAATGACAAAGCTACTGAACAGTCCATCTCCATC
GATCATATCCGCTCACAGTGTGCGACATGGAATACTTGCCAGTGCTTTCCATGCCTTTGCA
ACCAGAAGCATCTTTAACGTCTACTACCGTCCTTGGGCAAGGTCTTCTCAATTTATCACTC
CACTTGATCAGTATATAAAGGCGGTTCAATTTGACTACTGCTTCGGGACAAGATGTAGAAT
GCGAGTTGAAGGTGGAGAATCTGGGGAACAGAGATCCCTTGGCACTATCATTGGCACTG
AAGATCTCGATCCTATTAGATGGCAGAATTCCAAATGGAGATGTGTGAAGGTGAAATGGGA
TCCCGCAGCAAGTTCGGTTTTGCTTCCCGAAAGAGTTTGTCCTTGGAGCATCGATCTCAC
GGAATTCACCAAGAAAAAGAAAGCTTCCACTCTGCATCATCAGAAGAGGGCTCGCCCCA
ACAACGCATCATCCCCTGAGTTTTCTAACTTGCTTATGGATGGCATGTTGCATGGTACAGC
TAAAAATCAATCTCAAAGTAGTTCAGGGGTCTTGCAAGGTCAAGAAGACAGTGACACATG
CGTGAATCAATCCAGTGTACTGCAACAATCATTACCGCATCTTCTCCCGCAAGATCCTGGC
TGTGCCTCAATGCAACAGCAGATGCATAAGCAACTAGAAATTCAGATTCCGACCTGTGAC
CCATTTTATCAATGTTCCAGCAACACAGCACACTTTTCTGGTAGGAAAGTACCAGGTTTGT
GTAATGGGCTCTCAACAATCTCCTCTAACAGAGTTCATGATGATGCTCGTGCTACCAAAAA
CGGAACTTCTTTGTCCAGACCAAATGGCAGTCACAGATGCATGGTTTTCGGAGTAAATTTA
TTTAATGGCTCACCTGAGCTCCCTTCACCACAAGTTCTCACTTCTAGTGAGGTTCAATGCT
CGACTCCTCTCACTTCTCAGTCGAGTGTTTCCATTGCTTCCAAGGGTATATCTAGCAAGCA
ATGCAACAACTGTTGCTCCGTCGGCGATCGAACTTGCACCAAAGTGCTCAAGTATGGAAC
TAATCTTGGAAGATCTGTTGATCTCTATCGATTCAACGGATACAAAGGCCTCATCCTTGAG
CTTGATCATATGTTTGATTTCAATGGAAAGTTGATCGATGGAAGCAGCGGCTGGCACATAA
CCTATACCAATGAAGATGGGGATATGATGCTGATTGGAGATCCTTATCCATGGCAGAAATTT
CAGCATGAAGTCCGAAGGATGGTTATCCGCCCAAAGGAAGAAATCAACAGGCTGAATCC
GAGCTCACCGAGTTCAGCATCTTACTGA
